# Bicyclo[1.1.1]pentane Ketones via Friedel–Crafts Acylation

**DOI:** 10.1021/acs.joc.5c01754

**Published:** 2025-12-22

**Authors:** Karolina Urbańska, Freya Ritterling, Brendan Twamley, Oliver Cseh, Vitalina Levchenko, Vasyl Ripenko, Pavel K. Mykhailiuk, Mathias O. Senge

**Affiliations:** † School of Chemistry, Trinity Biomedical Sciences Institute, Trinity College Dublin, 8809 The University of Dublin, 152−160 Pearse Street, Dublin D02 R590, Ireland; ‡ School of Chemistry, Trinity College Dublin, The University of Dublin, Dublin 2 D02 CX56, Ireland; § 376198 Enamine Ltd, Winston Churchill Str. 78, Kyiv 02094, Ukraine; ∥ Taras Shevchenko National University of Kyiv, Chemistry Department, Volodymyrska 64, Kyiv 01601, Ukraine; ⊥ Institute for Advanced Study (TUM-IAS), Technical University of Munich, Lichtenbergstr. 2a, Garching 85748, Germany; # Trinity Translational Medicine Institute, St. James’s Hospital, Trinity College Dublin, The University of Dublin, Dublin D08W9RT, Ireland

## Abstract

Bicyclo­[1.1.1]­pentane (BCP) is a rigid aliphatic hydrocarbon with a three-dimensional (3D), propeller-like shape and a molecular size that makes it a targeted bioisosteric replacement for phenylene and acetylene groups in medicinal chemistry. For the pharmaceutical application of BCP, simple, efficient, and cost-effective synthetic tools are required to enable the exploration of BCP’s potential as a bioisostere across a broad chemical space. With numerous sophisticated protocols for C­(sp^3^) functionalization of rigid aliphatic hydrocarbons reported in the literature, the synthesis of BCP mono- and diketones remains a challenging task, limited by both substrate scope and expensive photocatalysts. Here, we present a protocol for Friedel–Crafts acylation of (hetero)­aromatic hydrocarbons with BCP acyl chlorides; in particular, the first method to access diaromatic BCP 1,3-diketones. Reaction optimization, substrate scope, and reactivity of the products are discussed. A total of 35 mono- and diketones are reported, accompanied by 7 examples of postsynthetic modifications. The synthesis of a BCP analogue of fenofibrate is reported. Noncovalent interactions of the compounds in the solid state are discussed, including a Hirshfeld analysis.

## Graphical abstract

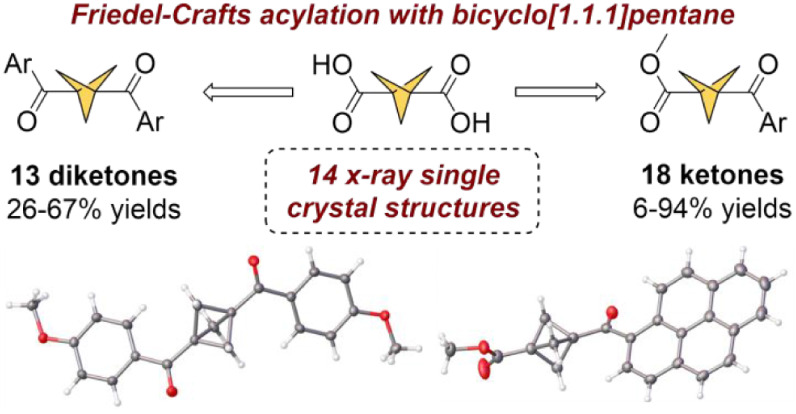

## Introduction

In recent years, bicyclo[1.1.1]­pentane (BCP) has become a medicinally relevant structural motif with several BCP derivatives reported as potentially bioactive.^1,2^ While the molecular size of bicyclo[1.1.1]­pentane finds a closer match with an internal acetylene motif than a phenyl ring,^3,4^ it is the latter that has been targeted for bioisosteric replacement with BCP in the past three decades.^5–7^ The presence of a sp^3^-rich three-dimensional (3D) motif is now known to improve the physical properties (such as solubility),^8^ bioavailability,^9^ metabolic stability,^2^ and other medicinally relevant features^1,10^ when compared to phenyl ring containing counterparts. In this context, the development of simple, inexpensive, and scalable synthetic processes targeting medicinally relevant BCP motifs is crucial for pharmaceutical applications.

A ubiquitous motif found in both medicinal chemistry and natural products is aryl ketones like benzophenones. Representative examples include applications in sun protection,^11^ nonsteroidal anti-inflammatory drug ketoprofen,^12,13^ cholesterol-reducing fenofibrate,^14^ and Parkinson’s treatment tolcapone (Figure 1).^15^ Thus, evaluation of the bioisostere containing equivalents presents the opportunity to modulate the pharmacokinetic and pharmacodynamic properties of this class of molecules. Therefore, developing synthetic methods to access this chemical space is essential for the drug design and development process.^16^

**Figure 1:**
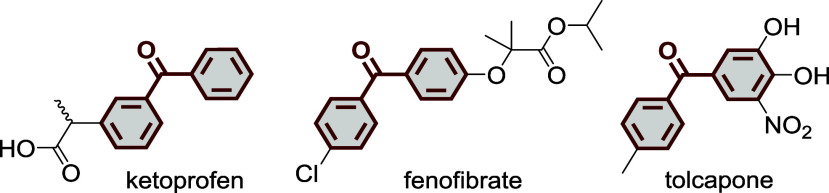
Chemical structures of benzophenone-containing drugs: ketoprofen, fenofibrate, and tolcapone.

To date, several approaches toward BCP ketones were reported (Scheme 1). Examples of installation of the carbonyl group in BCP bridgehead position involve both radical and anionic mechanisms, illustrating the omniphilic nature of bicyclo[1.1.1]­pentane’s precursor [1.1.1]­propellane.^17^ The first report on acylations of propellane was published by Wiberg in 1990,^18^ but the past five years have brought numerous synthetic approaches toward 1- and 1,3-(di)­substituted BCP ketones, involving strain release reactions of propellane with nucleophiles. 2-Aryl-1,3-dithianes^19^ and Grignard reagents^20,21^ react with BCP forming intermediates which can undergo transformation to BCP ketones (Scheme 1). Alternative to the stepwise protocols, radical acylations of [1.1.1]­propellane with aldehydes^22^ and hydrazides^23^ were reported to form 1-substituted derivatives. Multicomponent metallophotoredox reactions yield 1,3-disubstitution in one synthetic step.^24–26^ Another example is generation of a BCP methyl ketone from a corresponding ester using a traceless activating sulfinate group.^27^

**Scheme 1:**
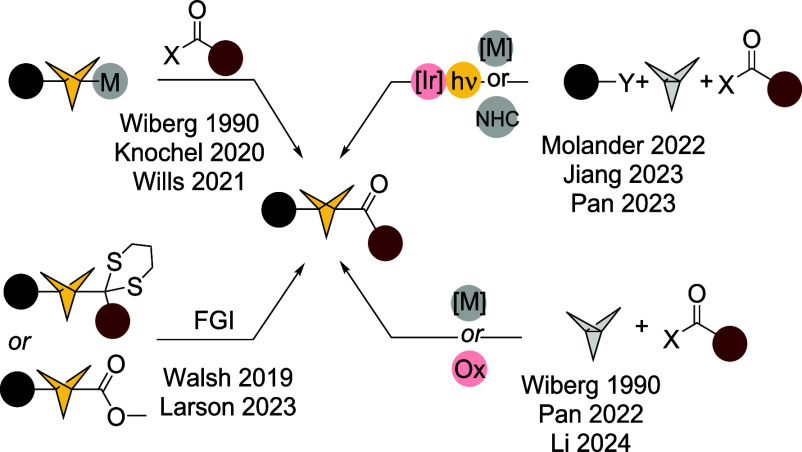
State of Art in BCP Ketone Synthesis^a^

a M = Li or MgBr, [M] = metal catalyst, [Ir] = iridium catalyst, NHC – *N*-heterocyclic carbene, FGI – functional group interconversion, Ox – oxidant, X = H or Cl, Y = BF_3_K.

These examples illustrate the prevalence of sophisticated methods in the development of BCP-carbonyl architectures. We aim to address the gap in the synthetic protocols by developing simple, scalable and inexpensive methods for BCP functionalization. Here we present access to BCP 1-ketones using bicyclo[1.1.1]­pentane-1,3-dicarboxylic acid **1** as a starting material.^28^ In addition, this method gives facile synthetic access to BCP 1,3-diketones, which, with few exceptions,^29,30^ remain synthetically underexplored.

Developed almost 150 years ago,^31^ Friedel–Crafts acylation finds widespread applications from total synthesis^32^ to industrial chemistry^33^ and undergraduate education.^34,35^ While variants of the Friedel–Crafts reaction have been reported,^36^ the original conditions provide an inexpensive, ubiquitous, and easily accessible route to carbonyl compounds. Here we present a protocol for Friedel–Crafts acylation of (hetero)­aromatic hydrocarbons with bicyclo[1.1.1]­pentane-derived acyl chlorides (Scheme 2). This simple, scalable procedure allows access to BCP mono- and diketones of (hetero)­aromatic hydrocarbons with moderate to excellent yields under mild conditions and using inexpensive reactants. Furthermore, we present the synthesis and subsequent reactivity of a library of bicyclo[1.1.1]­pentane 1,3-diketones. In addition, we examined the noncovalent interactions of both mono- and diketone BCP products, capturing the first report of hydrogen bonding interactions via the bridging methylene groups in the solid state.

**Scheme 2:**
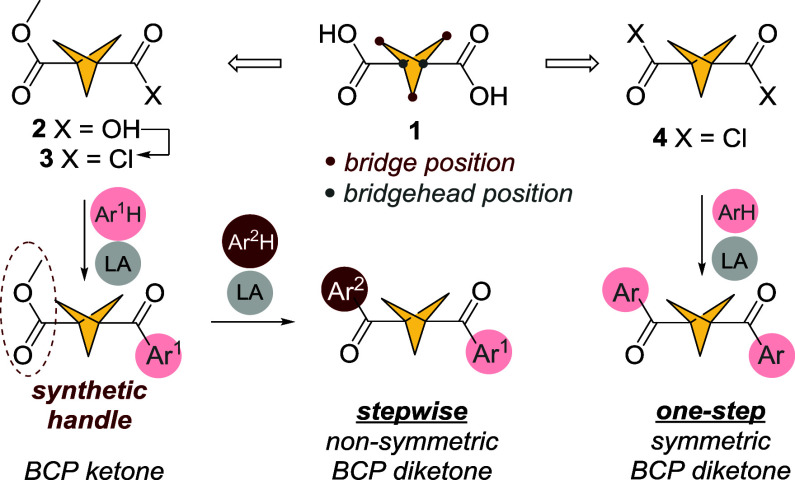
Carboxylic Group Transformations as Access to BCP Ketones Explored in This Work[Fn sch2-fn2]

a Ar, ArH, Ar^1^H, Ar^2^H – aromatic groups, LA – Lewis acid.

## Results and Discussion

### Optimization

We investigated the Friedel–Crafts acylation of anisole **5** with acyl chloride **3** (Scheme 3 and Table 1). The initial screening of reaction conditions showed that a slight excess of AlCl_3_ gave 0% conversion into ketone **6a** (Table 1 entry 1) and an increase to 10 equiv. resulted in full conversion and 88% isolated yield (Table 1 entry 2). Five equiv. AlCl_3_ yielded 88% conversion and 68% isolated yield (Table 1 entry 3). A screening of Lewis acids (FeCl_3_, ZnCl_2_, BBr_3_, Table 1 entries 4–6) revealed AlCl_3_ to be the most suitable for this transformation.

**Scheme 3:**
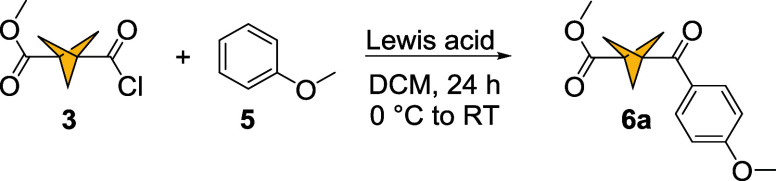
Friedel–Crafts Acylation of Anisole with BCP 3-Acyl Chloride

**Table 1:** Lewis Acid Screening

Entry	LA	eq	Conversion [%]^a^	Yield [%]
1	AlCl_3_	1.2	0	0
2	AlCl_3_	10	100	88
3	AlCl_3_	5	88	68
4	FeCl_3_	5	0	0
5	ZnCl_2_	5	0	0
6	BBr_3_	5	0	0

a Determined by ^1^H NMR. LA – Lewis acid.

Next, we carried out optimization of the model reaction for Lewis acid excess, reaction time, and temperature. We found that 2 equiv of AlCl_3_ was insufficient to drive the reaction to completion within 24 h, resulting in only 21% conversion—over 4 times lower than that obtained with 5 equiv of Lewis acid (Table 2 entries 1 and 2). Reducing the reaction time from 24 h to 4.5, 3, and 1 h caused significant decrease in conversion from 88% to 60%, 56% and 31%, respectively (Table 2 entries 3, 4, 5). Finally, when the temperature was decreased to 0 °C for 24 h, no product formation was observed (Table 2 entry 2). The isolated yield of the reaction was determined for entry 1, as the eentry with the highest conversion; the isolated yield was equal to 68%. Standard conditions for Friedel–Crafts acylation of (hetero)­aromatic hydrocarbons with BCP acyl chlorides were established as in Table 2 entry 1:5 eq. AlCl_3_ added at 0 °C, then stirring at RT for 24 h.

**Table 2:** Optimization of Friedel-Crafts Acylation with AlCl_3_

Entry	LA eq	*t* [h]	*T* [°C]	Conv. [%]^a^
1	5	24	0 to RT	88
2	2	24	0 to RT	21
3	5	4.5	0 to RT	60
4	5	3	0 to RT	56
5	5	1	0 to RT	31

a Determined by ^1^H NMR. LA – Lewis acid, Conv. – conversion.

### Scope and Limitations

#### BCP Ketones

With the standard conditions in hand, we proceeded to synthesize a library of mono- and diketones, from acyl chlorides **3** and **4**, respectively. Reactions of acyl chloride **3** yielded 18 ketones **6a**–**r** with moderate to excellent yields (Scheme 4).

**Scheme 4:**
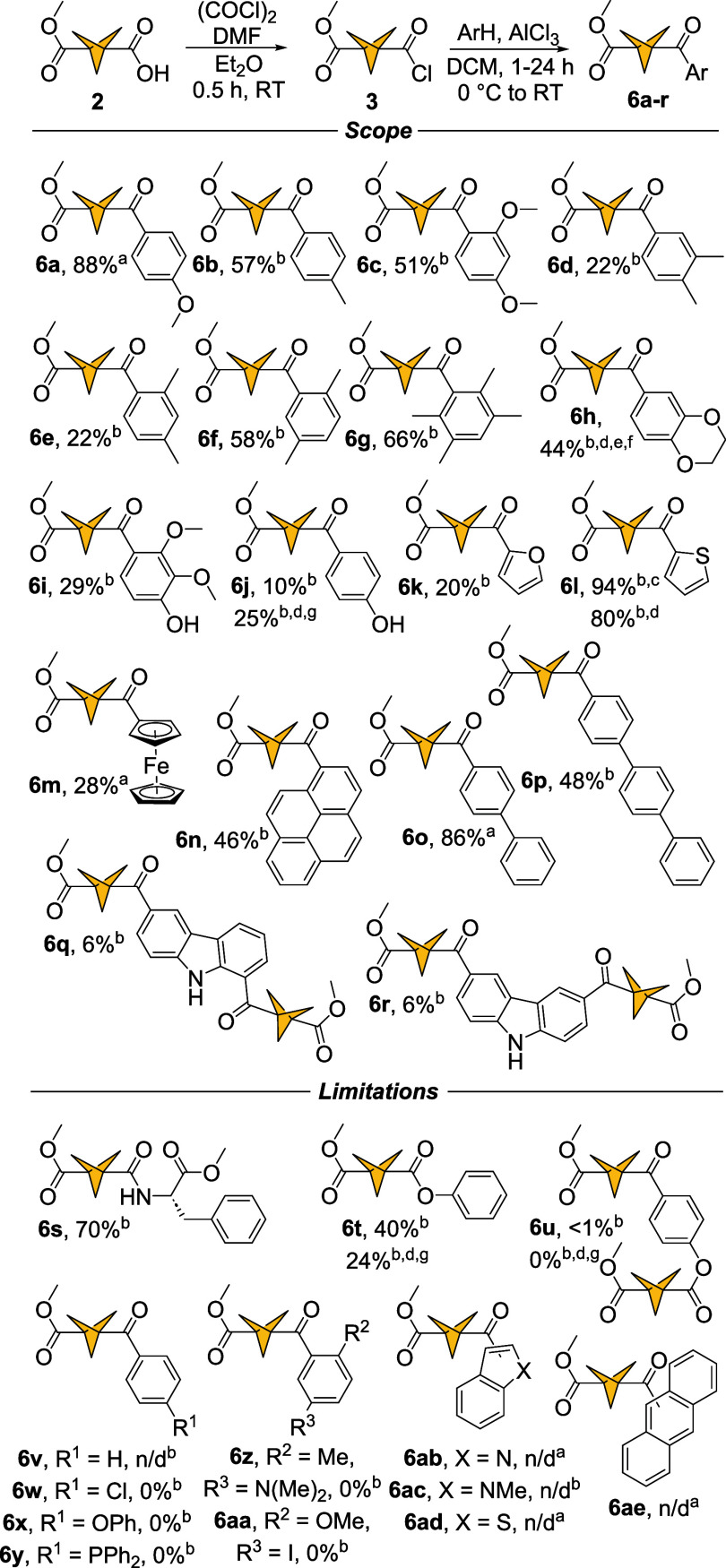
Friedel–Crafts Acylation of Hydrocarbons with BCP 3-acyl Chloride[Fn sch4-fn3]

a
Reaction time: 1 h, ^b^reaction time: 24 h, ^c^scale: 0.27 mmol, ^d^scale: 2.7 mmol, ^e^10 eq. AlCl_3_ used, ^f^reaction at 30 °C, ^g^reaction at 50 °C.

In the range of monosubstituted phenyl rings, anisole and toluene yielded ketones **6a** and **6b** with 68% and 87% yields, respectively. Substrates with multiple activating groups on the phenyl ring (1,3-dimethoxybenzene, *o*-xylene, *m*-xylene *p*-xylene, and 1,2,4,5-tetramethylbenzene) reacted with BCP **3** with low to good yields (**6c**, 51%; **6d**, 20%; **6e**, 20%; **6f**; 58%, **6g**, 66%). Acylation of 2,3-dihydro-1,4-benzodioxine produced **6h** in 44% yield. This experiment was carried out using 10 equiv of catalyst, on 2.7 mmol scale. A reaction with 1,2,3-trimethoxybenzene under standard conditions resulted in demethylation of the *para*-methoxy group. No *O*-acylation was observed and **6i** was isolated in 29% yield. However, *O*- and *N-*acylations were observed in reactions with phenol and *N*-Boc phenylalanine methyl ester, respectively. Under acidic conditions of Friedel–Crafts acylation the acid-labile Boc protecting group was cleaved and free primary amine was acylated to form product **6s** with 70% yield. No side chain acylation was observed in this case. On the other hand, acylation of phenol under optimized conditions yielded a mixture of monoacyl products **6j** and **6t** in 10 and 40% yield, respectively. Additionally, a small amount of diacyl compound **6u** was identified in the reaction mixture (see Figure S138 for HRMS). Increasing phenol excess to 1.5 equiv and the reaction temperature to 50 °C helped shift the reactivity toward the ketone **6j** isolated with 25% yields under the revised conditions (suppressing both *O*-acylation and double acylation, as no **6u** was detected and **6t** was isolated with 24% yield). Furan and thiophene underwent acylations to form heterocyclic ketones **6k** and **6l** with 20% and 94% yields, respectively. Ferrocene and pyrene ketones **6m** and **6n** formed with 28% and 46% yields. Oligophenylenes, such as biphenyl and *p*-terphenyl yielded ketones **6o** and **6p** in 86% and 48% yields, respectively. Carbazole underwent multiple acylations to yield 1,6- and 3,6-diacylated products **6q** and **6r**, each with 6% yield. Reactions with chlorobenzene, diphenyl ether, and triphenylphosphine resulted in no conversion into the desired products **6w**, **6x**, and **6y**. Similarly, disubstituted substrates 4-methyl-*N*,*N*-dimethylaniline and 4-iodoanisole did not produce ketones **6z** and **6aa**. Benzene, benzo­[*b*]­thiophene, indole, and anthracene produced complex inseparable mixtures of acylation. Formation of ketones **6u, 6v**, **6ab**, **6ac**, **6ad**, and **6ae** was confirmed by mass spectrometry only (see Supporting Information). A large-scale acylation of thiophene (2.7 mmol, 500 mg acyl chloride **3**) produced the corresponding ketone **6l** in 80% yield. Compounds **6a**, **6m**, and **6o** were isolated after 1 h in yields reported herein, as the starting material was fully consumed after this time as indicated by ^1^H NMR. In all other cases reactions were carried out for 24 h. The structures of compounds **6a**, **6b**, **6c**, **6k**, **6l**, **6m**, **6n**, **6o**, and **6r** were confirmed by X-ray diffraction analysis.

#### BCP Diketones

Reactions with diacyl chloride **4** under general conditions produced a library of 13 symmetric 1,3-BCP diketones **7a**–**m** (Scheme 5). The isolated yields of diketones are slightly lower than those of monoketones. Anisole and toluene produced diketones **7a** and **7b** in 51% and 45% yields, respectively. Xylene isomers (*o-*, *m-*, *p*-) yielded diketones **7c**, **7d**, and **7e** in 38%, 44%, and 55% yield, respectively. 1,2,4,5-Tetramethylbenzene produced diketone **7f** in 26% yield. Bis­(heteroaromatic) diketones **7g** and **7h** were obtained through acylation with furan and thiophene, in 36% and 54%, respectively. Reducing reaction time from 24 to 1 h for acylation of thiophene resulted in a significantly lower yield (17%). Both biphenyl and *p*-terphenyl products **7i** and **7j** were formed in 28% and 56% yield, respectively.

**Scheme 5:**
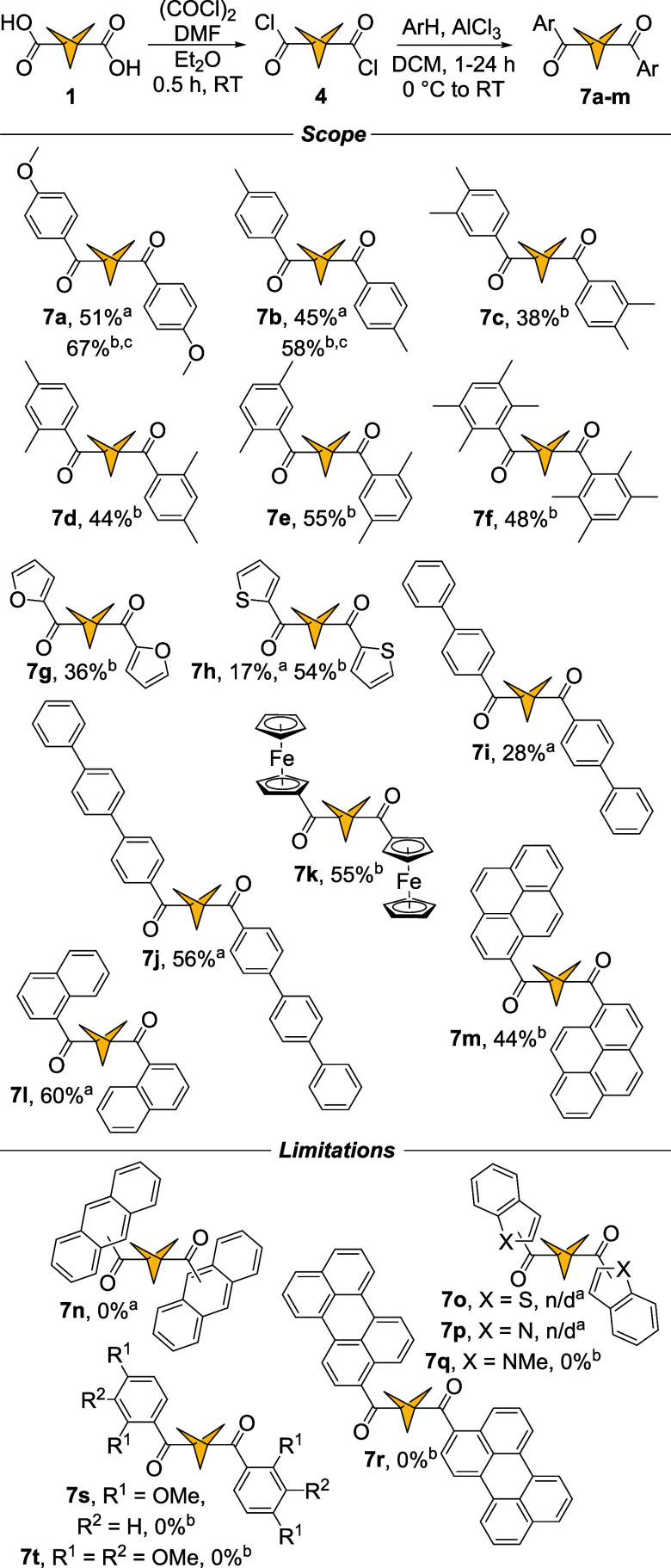
Friedel–Crafts Acylation of Hydrocarbons with BCP 1,3-Bis­(acyl chloride)[Fn sch5-fn4]

a
Reaction time: 1 h, ^b^reaction time: 24 h, ^c^scale: 10 mmol.

Acylation of ferrocene yielded diketone **7k** in 55% yield. In the range of polycyclic aromatic hydrocarbons, only naphthalene and pyrene produced the desired ketones **7l** and **7m** in 60% and 44% yields, while perylene failed to form ketone **7r**. Similarly to symmetric monoacylations of BCP **3**, attempts to generate diketones **7o**, **7p**, and **7q** from acyl chloride **4** and benzo­[*b*]­thiophene, indole, and *N*-methylindole produced complex inseparable mixtures of regioisomers. Surprisingly, neither 1,3-dimethoxybenzene nor 1,2,3-trimethoxybenzene gave the corresponding diketones **7s** and **7t**. However, 1 h reaction time was sufficient to isolate compounds **7a**, **7b**, **7h**, **7i**, **7j**, and **7l** in reported yields. In all other cases, acylations were carried out over 24 h. Large scale (10 mmol, 24 h) experiments with anisole and toluene produced compounds **7a** and **7b** in 67% and 58% yields, respectively. Similar to acylations with BCP **3**, attempts to generate diketones **7n**, **7p**, **7r**, and **7t** were carried out with 1 h reaction time, resulting again in inseparable mixtures of regioisomers. The structures of compounds **7a**, **7b**, and **7l** were confirmed by single crystal X-ray crystallography. Overall, yields obtained for diketones **7a**–**m** are lower than those for ketones **6a**–**r** with few exceptions: furan and ferrocene gave higher yields for compounds **7h** (36%) and **7l** (55%) than **6k** (20%) and **6m** (28%), respectively.

Having established the scope of hydrocarbons with BCP acyl chlorides **3** and **4**, we tested other BCP derivatives as acylating agents (Scheme 6). 1-Fluorobicyclo­[1.1.1]­pentane-3-carboxylic acid **8a** and 1-methylbicyclo[1.1.1]­pentane-3-carboxylic acid **8b** were selected as potential precursors to bioisosteres of benzophenone-containing medicinally relevant compounds (Figure 1). As a model substrate for acylation, we selected thiophene, taking advantage of the high yield of acylation with unsymmetrical acyl chloride **3** (**6l**, 94%). Due to the volatility of acyl chlorides prepared from acids **8a** and **8b**, a two-step one-pot protocol for generation of ketones **9a** and **9b** was utilized to avoid isolation of the intermediate acyl chlorides. Under these conditions, only **8b** produced the corresponding ketone **9b** with a good yield of 65%. The analysis of the crude reaction mixture of **8a** with thiophene revealed a lack of fluorinated species.

**Scheme 6:**
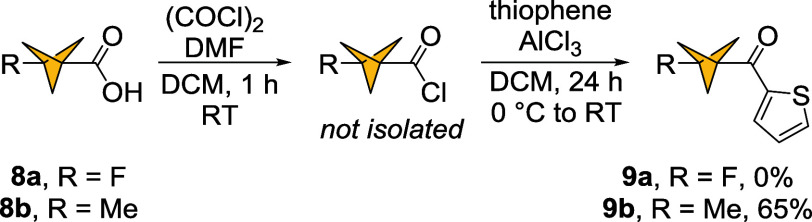
Friedel–Crafts Acylation of Thiophene with 1-Fluoro and 1-Methylbicyclo[1.1.1]­pentane Acyl Chlorides

#### Reactivity of BCP Ketones

Next, we investigated the reactivity of mono- and diketones **6a**–**u** and **7a**–**m** to demonstrate their broad synthetic applications. Reduction of ketones **6l** and **7b** with NaBH_4_ smoothly produced alcohol **10** and diol **15** with 80% and 86% yield, respectively (Scheme 7a,d). Alcohols **10** and **15** were isolated as mixtures of enantio- and diastereomers. As outlined in Scheme 2, our method can be used to generate unsymmetrical BCP diketones. Here, we applied a three-step synthetic sequence to transform ketone **6l** into unsymmetric diketone **13** (Scheme 7b).

**Scheme 7:**
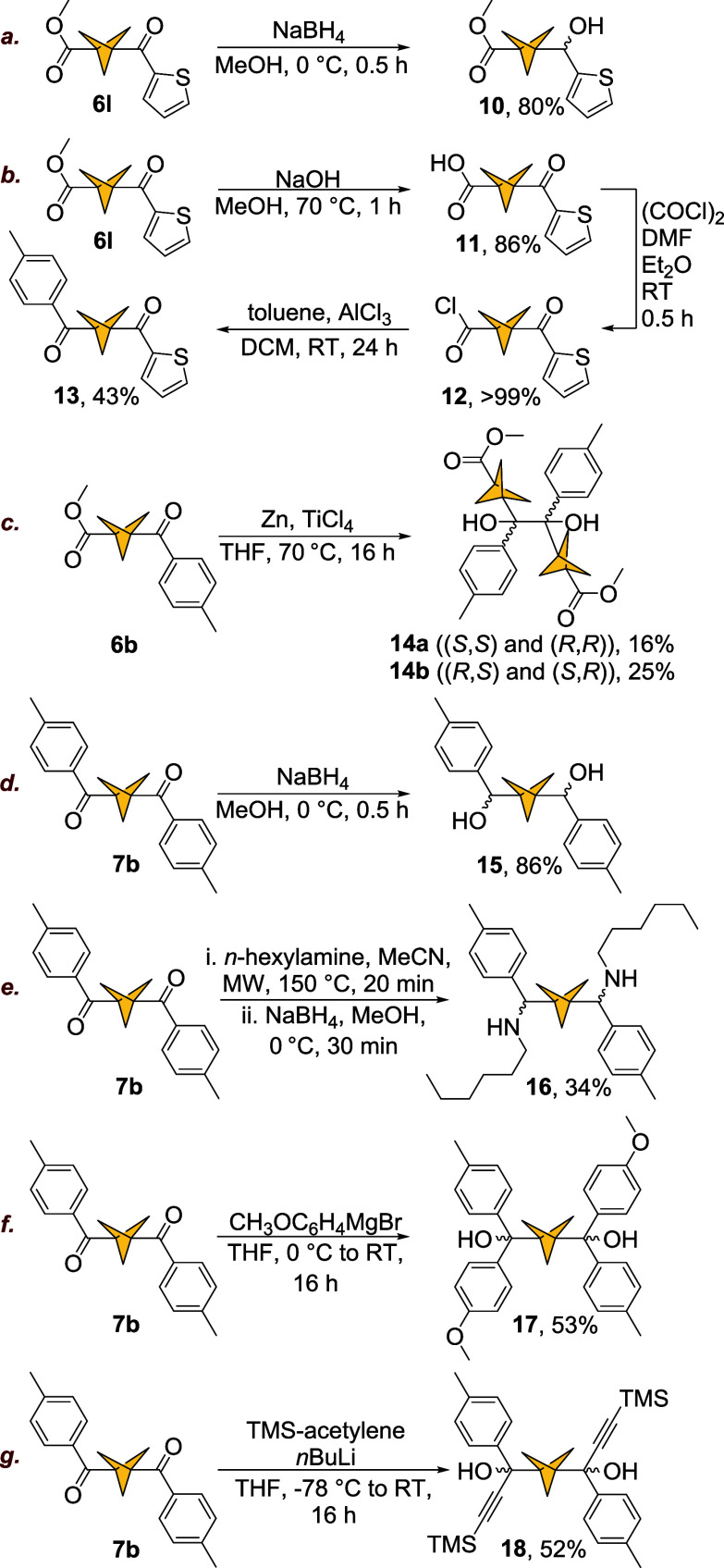
Reactions of BCP Ketones and Diketones[Fn sch7-fn5]

a a, d – reduction of ketone to alcohol; b – synthetic path toward unsymmetrical BCP diketones: methyl ester hydrolysis, acyl chloride generation and Friedel–Crafts acylation; c – McMurry coupling of BCP ketone **6b**; e – reductive amination of BCP diketone **7b** with *n*-hexylamine; f – addition of a Grignard reagent to diketone **7b**; g – addition of acetylide to diketone **7b**.

Hydrolysis of the methyl ester in ketone **6l** yielded carboxylic acid **11** (86%), which was subsequently transformed quantitatively into acyl chloride **12**. Compound **12** was subjected to Friedel–Crafts acylation with toluene to yield unsymmetric diketone **13** with 43% yield (Scheme 7b). Overall, this three-step procedure gave access to diketone **13** in 37% based on **6l.** Based on BCP carboxylic acid **2**, this 5-step procedure, involving one chromatographic column (isolation of final compound **13**) and one crystallization (isolation of ketone **6l**) resulted in a 35% yield. This example illustrates the simplicity of our Friedel–Crafts acylation-based approach, enabling access to complex structural targets involving bicyclo[1.1.1]­pentane.

Another modification of BCP ketones led to an unexpected result. McMurry coupling of ketone **6b** yielded pinacols **14a** and **14b** with 25% and 16% yields, respectively (Scheme 7c). Separation of the enantiomers was not attempted. Formation of the anticipated BCP alkenes was not observed. This result can be explained by the steric bulk of the BCP motif causing dissociation from the titanium center before elimination can occur, resulting in the isolation of the pinacol.^37^

Further examples of carbonyl reactivity in BCP ketones include the reactivity of diketone **7b**. Reaction with *n*-hexylamine under microwave irradiation and subsequent reduction with NaBH_4_ produced amine **16** in 34% yield (Scheme 7e). This two-step protocol was carried out without isolation of the intermediate imine. Addition of 4-methoxyphenylmagnesium bromide to diketone **7b** gave diol **17** in 53% yield (Scheme 7f). Treatment of diketone **7b** with TMS-protected acetylide resulted in formation of propargyl diol **18** in 52% yield (Scheme 7g).

#### Synthesis of a BCP-Fenofibrate Analogue

We envisioned the application of our Friedel–Crafts acylation protocol as late-stage functionalization method in the synthesis of BCP-containing isosteres of fenofibrate (Figure 1). However, our attempts at acylation of fibric acid esters **19a** (*iso*-propyl) and **19b** (methyl) were unsuccessful under optimized conditions and at an elevated temperature (50 °C, Scheme 8a). In the acidic environment of the reaction, the *iso*-propyl ester in **19a** was cleaved; the nonlabile methyl ester of fibric acid **19b** was unreactive under conditions listed in Scheme 8a (starting materials were recovered). This method failed to produce compounds **20a** and **20b**. In a revised approach, we subjected the phenol derivative **6j** to a Williamson ether synthesis with bromide **21** and isolated compound **20a** in 48% yield (Scheme 8b).

**Scheme 8:**
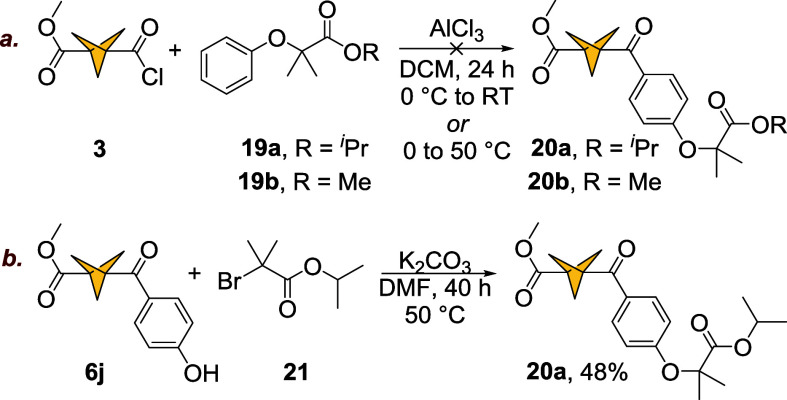
Synthesis of BCP Analogue of Fenofibrate

### Crystallography

The analysis of single crystal X-ray structures of BCP ketones revealed intriguing noncovalent interactions. Namely, we observed that BCP bridge (C2) hydrogen atoms can participate in weak noncovalent interactions in the solid state. The only examples reported to date by us^38^ and others^39^ involve CH···π interactions. Despite numerous BCP derivatives reported in the literature (∼300 structures) and the fast-growing scientific interest in solid state interactions of rigid aliphatic hydrocarbons,^40^ few examples of hydrogen bonding involving BCP bridge hydrogen atoms have been described to date. Given the importance in current medicinal chemistry^7,41^ and materials,^42–45^ there is a fundamental need to understand potential binding modes of BCP units in target environments. Here, we report 14 X-ray crystal structures determined for compounds synthesized in this project. We discuss the weak noncovalent interactions in solid state involving π–π stacking and, weak interactions involving BCP methylene hydrogen atoms.

Torsion angles between the BCP motif and the neighboring carbonyl groups were measured for all structures (Table S5). In the library of BCP ketones **6a**, **6b**, **6k**, **6l**, **6m**, **6o**, and **6r**, the ketone carbonyl group adopts a nearly coparallel conformation with respect to the nearest BCP bridgehead methylene group with the torsion angle values varying from −1.95(16)° for **6o** and 6.62(19)° for **6a**. A slight tilt of the carbonyl group with respect to the BCP bridge carbon was observed in **6k** (−16.0(2)°) and **6m** (19.0(6)°). An even more pronounced tilt is found in compounds **6c** (Figure 2a) and **6n**, where the torsion angles are −35.0(3)° and −27.20(17)°, respectively. In carbazole diketone **6r**, which contains two BCP ketone groups, a significant difference in the torsion angles determined for the two ketones was observed (−6.4(4)° and −22.6(5)°). In contrast, no similarity can be found in torsion angles measured between the same BCP methylene groups and the carbonyl oxygen atoms of the ester moieties within this range of compounds. These geometries are in line with those observed in BCP amides reported by us earlier.^46^

**Figure 2:**
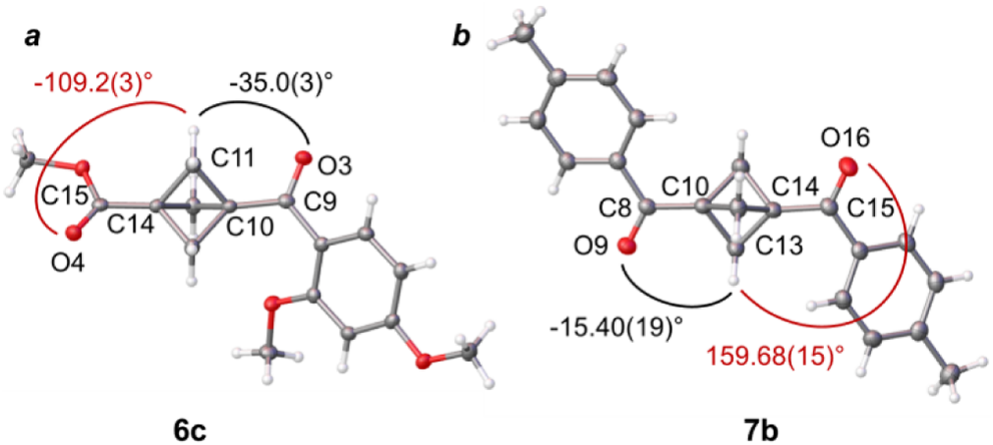
Torsion angles in ketones **6c** (a) and **7b** (b).

BCP diketones **7a**, **7b**, and **7l** show a slight tilt from a coparallel orientation between one of the carbonyl groups oxygen atom and the nearest BCP methylene group, equal −2.41(17)° (**7a**), −15.40(19)° (**7b**, Figure 2b) and 12.9(4)° (**7l**). As no clear trends could be determined for the dihedral angle data, these findings indicate that in the library of BCP mono- and diketones, the bicyclo[1.1.1]­pentane propeller motif allows unrestricted rotation of substituents at the bridgehead positions.

Interestingly, heterocyclic derivatives **6k** and **6l**, the heteroaromatic rings adopt opposite orientations with respect to the ketone motif and the BCP ring. The oxygen atom of the furan ring in **6k** is pointed toward the BCP propeller with a dihedral angle of −179.1277 (14)° (Figure 3a). In contrast, the sulfur atom in thiophene motif of **6l** adopts a coparallel orientation with the ketone oxygen atom, with a dihedral angle of 11.0679 (3)° (Figure 3b).

**Figure 3:**
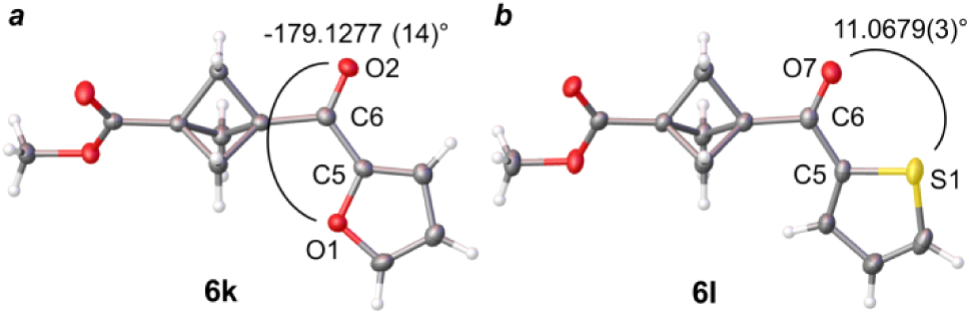
Heterocycle orientation in ketones **6k** (a) and **6l** (b).

Of the 14 crystal structures obtained, three exhibited strong hydrogen bonding networks. Etter’s graph-set notation was utilized to characterize crystal structures **6r**, **14a**, and **14b** (Table S6).^47^ All hydrogen bond networks found are arranged in either chain, rings, or combinations of both. Compound **6r** exhibits hydrogen bond chains with 8 member atoms, between the donor NH group of carbazole and the acceptor oxygen of a carbonyl. This same chain motif is seen in **14a**. **14a** also forms longer chains of 9 and 18 atoms with one and two donor and acceptor pairs, respectively. In addition to chains, both **14a** and **14b** form ring networks. A 34-membered ring with 4 hydrogen bond donor and acceptor pairs and a 52-membered ring with 6 hydrogen bond donor and acceptor pairs are observed in **14a**. **14b** displays a 16-membered ring with 2 hydrogen bond donor–acceptor pairs. **14a** and **14b** vary only by their stereochemistry, thus the more complex hydrogen bonding networks seen in **14a** are due to trans orientation of the hydroxyl groups with respect to each other. In both these compounds, strong hydrogen bonding occurs between hydroxy groups and ester carbonyls.

Examples of such interactions in compounds **6a** and **6o** are shown in Figure 4. In **6a** the donor–acceptor contact between bridge CH and ether O was observed with a distance of 3.423 Å (Figure 2a) and in **6o**, the distance between BCP bridge CH and the carbonyl oxygen atom of the ester group is 3.481 Å (Figure 2b). In **6n** the distances between each of the bridge CH to ketone carbonyl are 3.228 Å, 3.319 Å, and 3.363 Å (Figure S180), and in **6m** the same distance is 3.30 Å (Figure S182).

**Figure 4:**
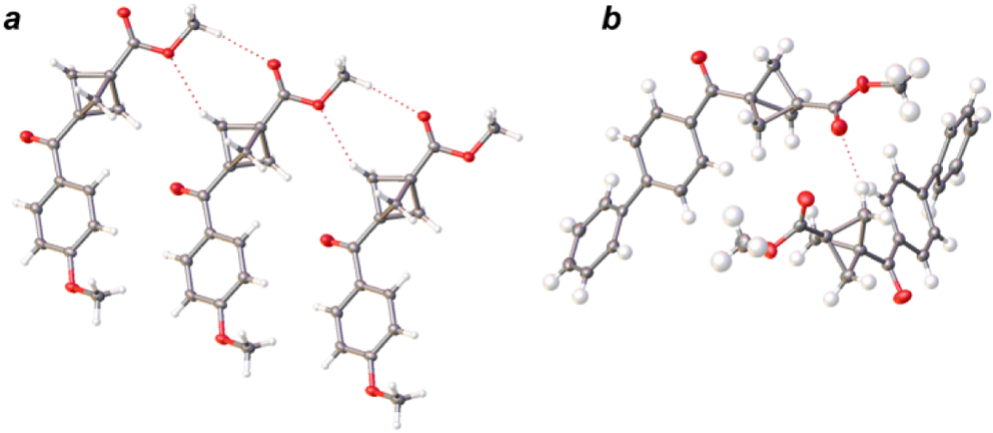
Noncovalent interactions involving BCP bridgehead positions in **6a** (a) and **6o** (b).

Additionally, in most cases, carbonyl oxygen atoms participate in H-bonding interactions with other donors. In **6a** the distance between methoxy group CH and the ester carbonyl O is 3.359 Å. In **6k,** the heterocyclic 2-H interacts with ester carbonyl O at 3.248 Å. In **6n** pyrene 5-H interacts with the ester carbonyl oxygen with distance 3.360 Å. In **7b** two *ortho*- hydrogen atoms, 2-H and 6-H, interact with carbonyl oxygen atoms with 3.273 Å distances. In **7a,** the methoxy CH interacts with the carbonyl oxygen at 3.247 Å. Likewise, in **6a** the methoxy CH_3_ interacts with the methoxy ether oxygen over 3.278 Å. Compound **6m** shows an intramolecular interaction of the cyclopentadienyl CH with ketone oxygen 3.290 Å. The parallel orientation of aromatic rings in **6b** and **6n**, and the respective distances (3.690 Å in **6b** and 3.532–3.774 Å in **6n**) indicate possible π–π interactions in the solid state (Figure 5).

**Figure 5:**
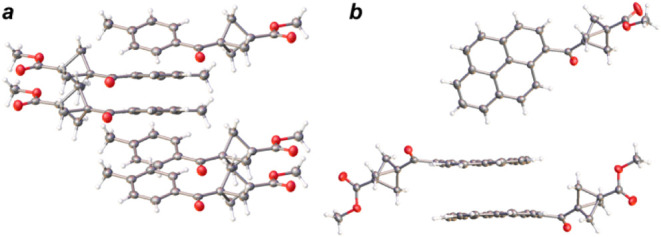
Parallel orientation of aromatic rings in BCP-aryl ketones indicating π–π stacking in **6b** (a) and **6n** (b).

Intermolecular interactions were analyzed using 2D fingerprint plots of the Hirshfeld surface for all crystal structures except for **6k**.^48–50^ The crystal structure of **6k** contains disorder in the methoxy groups which led to difficulty in calculating the Hirschfeld surface and impacts the fingerprint plots, rendering them inaccurate. However, the notable interactions for **6k** elucidated by the crystal structure are interactions via the carbazole nitrogen and carbonyl groups, previously categorized using Etter’s graph-set notation. For most of the compounds, the Hirshfeld surfaces around BCP only show van der Waals interactions or the lack of interactions: **6b**, **6c**, **6k**, **6l**, **6r**, **7a**, **7b**, **7l**, **14a**, and **14b**. However, surfaces for compounds **6a**, **6m**, **6n**, and **6o** indicate stronger interactions (Figure 6a–d). These interactions mainly consist of bicyclo[1.1.1]­pentane’s methylene groups with oxygen atoms in carbonyl moieties. This interaction corresponds to the weaker hydrogen bonds identified in the crystal structures of these compounds.

**Figure 6:**
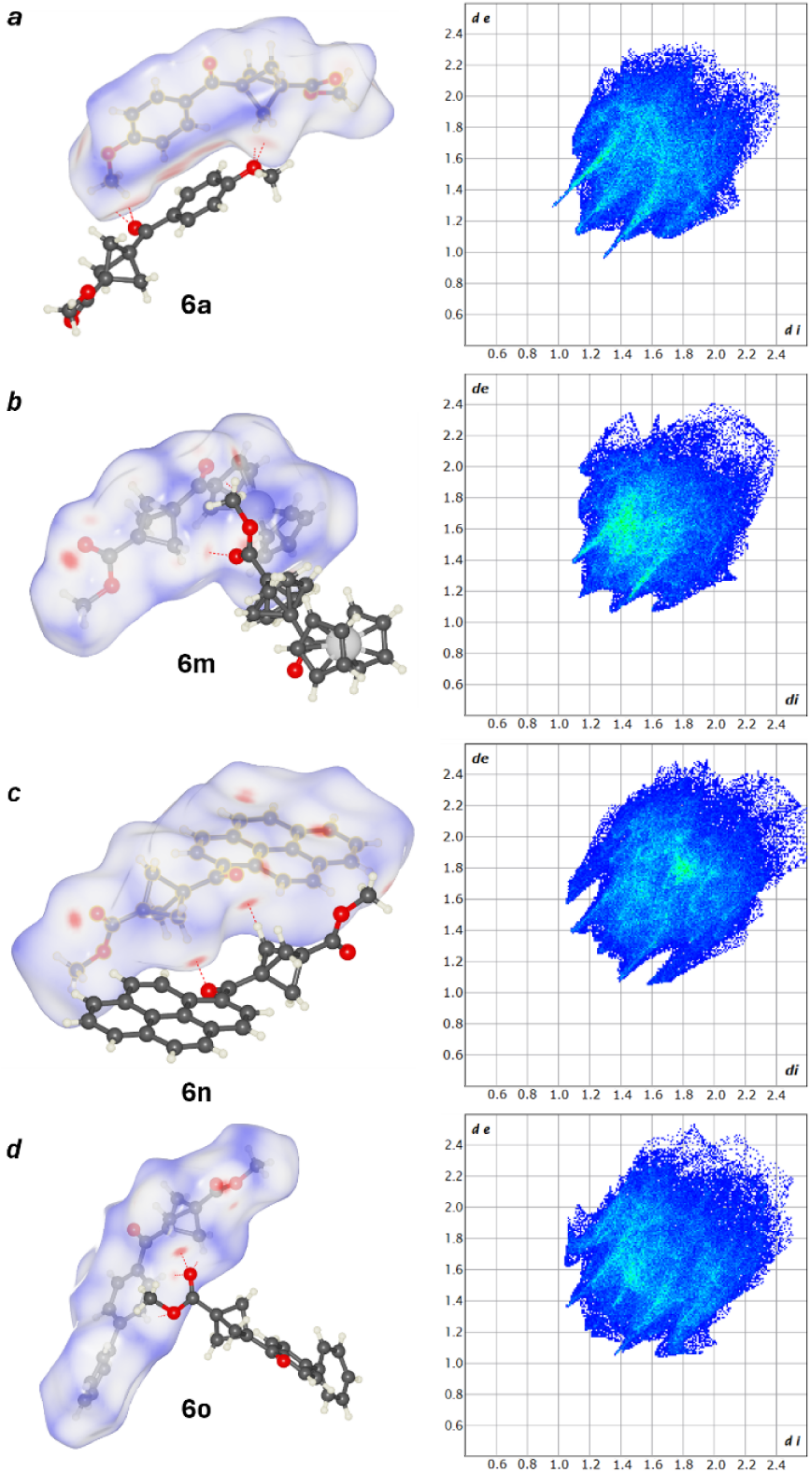
Hirshfeld surfaces with surface colors indicating contacts shorter than van der Waal radii (red), equal to van der Waal radii (white), longer than van der Waal radii (blue), and red dotted line representing intermolecular interactions (left) and two-dimensional fingerprint plots (right) (a) **6a**, (b) **6m**, (c) **6n**, and (d) **6o**. Crystal Explorer 25 ^51^ was used to calculate the Hirshfeld surface.

The two-dimensional fingerprint plots of the crystals reveal hydrogen bonding, π–π, CH−π, H–H, and van der Waals interactions (see Supporting Information). Structures **6a**, **6l**, **14a**, and **14b** contain two sharp peaks directed toward the bottom left of the plots indicating weaker hydrogen bonding (Figure 6a). Less intense versions of these peaks appear in the fingerprint plots for **6b**, **6k**, **6n**, **6m**, **6o**, **7b**, and **7l**, suggesting weaker hydrogen-based donor–acceptor based interactions (Figure 6b–d). No hydrogen bonding peaks appear in the plots of **6c** and **7a**. While the crystal structure of **6c** displays hydrogen bonding between ethereal oxygens and methoxy CH, this interaction is not seen in the fingerprint plots as it is an intramolecular interaction. π–π interactions are associated with color changes to the region around 1.8, 1.8 (di, de). This occurs in the plots for compounds **6n** and **7b** which agrees with the crystal structure data obtained. π–π interactions for **6n** are localized between planes consisting of (C15, C14, C3, C4, C5, C6) and its symmetry equivalent (1-X, 1-Y, 1-Z). Their centroid-centroid distances measure 3.532 Å with a shift distance of 1.106 Å. Compound **7b** exhibits π–π interactions between (C2 C7 C6 C5 C4 C3) and the symmetry equivalent (2-X,1-Y,2-Z) with a centroid-centroid distance of 3.690 Å and shift distance of 1.066 Å. CH−π interactions are visualized as wings protruding to the top left and bottom right of the fingerprint plots. Distinct interactions are present in compounds **6a**, **6b**, **6o**, **7b**, **7l**, and **14b** (Figure 6a,d). Weaker interactions appear in the plots of compounds **6c**, **6m**, and **14a**; and CH−π interactions are absent for compounds **6k**, **6l**, and **6n** (Figure 6b,c). The most prevalent interaction in the crystal structures is H–H interactions associated with clusters of points in the region of 1.2, 1.2 (di, de). This is observed for compounds **6a**, **6b**, **6c**, **6k**, **6l**, **6n**, **6m**, **6o**, **7b**, **7l**, **14a**, and **14b** (Figure 6a–d).

For comparison, the Hirshfeld surface and two-dimensional plots were obtained for an analogous cubane derivative reported previously in literature as a part of a study on hydrogen bond networks in saturated aliphatic hydrocarbons. 1-(4-*tert*-butyl)­phenyl) 4-methyl-cubane-1,4-dicarboxylate is the closest cubane mimic to the BCP based compound synthesized here.^40^ Like the BCP compounds, the cubane contains of two bridgehead carbonyls, one of which is attached to an aromatic group. While similar in many ways to the BCP compounds, the cubane crystal structure exhibits much stronger hydrogen bonds. Multiple bonds between the cubane methine hydrogens form to carbonyl oxygens with the shortest interaction observed with a contact of 3.638 (2) Å (D···A), and 134.4° (D-H···A). Similar to the bifurcated bonds seen in compound 6n, trifurcated bonds were measured with the ester carbonyl binding three cubane methine hydrogens. The Hirshfeld surfaces for this compound shows weak energy interactions above the cubane methine hydrogen (Figure 7a), most likely reflecting the weakening of the bond due to trifurcation. The closer contacts of the cubane-based hydrogen bonds are substantiated by the two-dimensional fingerprint plots where the characteristic hydrogen bonding peaks extend further to the bottom left (Figure 7b). In addition, the cubane equivalent exhibits similar H–H, CH−π, and π–π interactions as the BCP-based compounds. Overall, the Hirshfeld surface analysis and two-dimensional fingerprint plots of BCP and cubane containing compounds contain analogous interactions.

**Figure 7:**
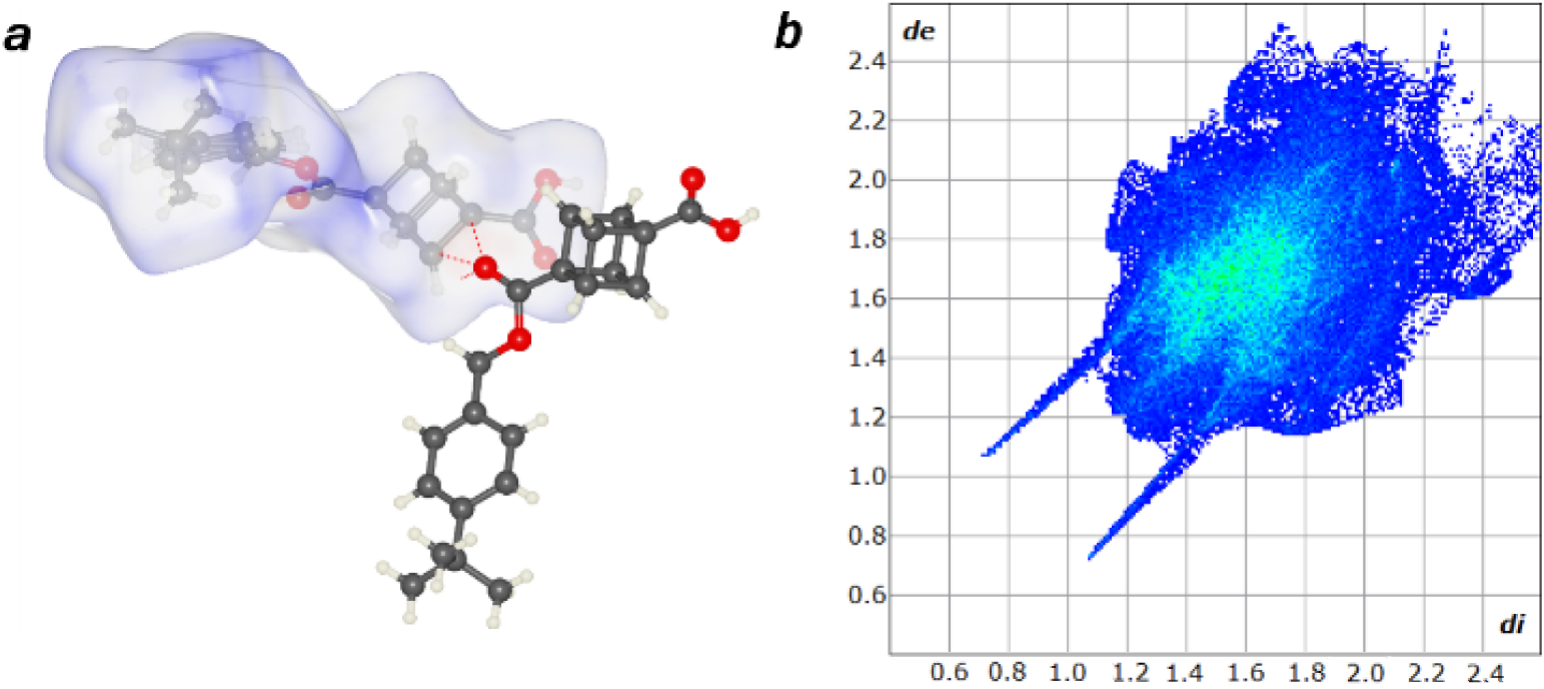
(a) Hirshfeld Surface Analysis for 1-(4-tert-butyl)­phenyl) 4-methyl-cubane-1,4-dicarboxylate with red dotted lines indicating intermolecular interactions. (b) Two-dimensional fingerprint plot. Crystal Explorer 25 ^51^ was used to calculate the Hirshfeld surface and the two-dimensional fingerprint plots.

The most apparent difference between the cubane compound and the BCP counterparts is the length of the hydrogen bonds. This difference in length and therefore strength of the bonds could be attributed to the different acidities of the strained aliphatic hydrocarbons. Cubane’s C–H acidity is well understood with numerous examples of cubane-based strong and weak hydrogen bonding reported in literature.^40,52–57^ In contrast, there is a lack of BCP based interactions reported; in particular, those approaching the length and directionality required for hydrogen bonding. This could be due to a potential decrease in methylene C–H acidity, compared to cubane.^38^ This is further substantiated with molecular modeling calculations which report BCP bridge-based radicals and carbocations as less stable than their bridgehead equivalents, supporting the stability of the BCP bridge C–H bond.^58,59^ In addition, the lack of mild methods for postsynthetic functionalization of BCP methylene hydrogens; this lack of reactivity further supports the stability of the bridge C–H bond.^59,60^

Overall, this suggests that the BCP methylene bonds are less acidic than the methine bonds in cubane, impacting their intermolecular interactions. This indicates that the two isosteres could potentially be utilized to modulate binding properties within chemical systems. This finding is in agreement with the results of Macreadie et al., whose studies indicate that cubane-based MOF (metal–organic framework) exhibits stronger intermolecular interactions than the corresponding BCP-based MOF.^42^ Therefore, as isosteres, cubane and bicyclo[1.1.1]­pentane might present the ability to selectively tune the hydrogen bonding strength of intermolecular interactions. As such, the choice of isostere has the potential to introduce binding specificity for chemical systems with bicyclo[1.1.1]­pentane presenting with the ability to serve as weak hydrogen bond donor.

## Conclusions

In conclusion, we reported an application of Friedel–Crafts acylation of (hetero)­aromatic hydrocarbons with BCP acyl chlorides as a simple, mild, and inexpensive method to access BCP mono- and diketones. We optimized the reaction conditions and synthesized a library of 33 BCP ketones. These BCP ketones can be subsequently transformed into amines, alcohols, alkenes, and include symmetric or unsymmetric diketones. We demonstrated the applicability of this method for the synthesis of a BCP analogue of fenofibrate alongside postsynthetic modifications of mono- and diketones. The thorough discussion of weak hydrogen bonding interactions provides valuable insights into potential binding modes for bicyclo[1.1.1]­pentane whose understanding is crucial for molecular design in both medicinal and material applications.

## Experimental Section

### General Experimental Information

^1^H and ^13^C­{^1^H} NMR spectra were recorded at 298 K on Bruker Avance III 400 MHz and Bruker Avance HD 400 MHz. The NMR spectra were run in CDCl_3_ with a solvent peak at δ_H_ = 7.26 ppm and δ_C_ = 77.2 ppm and CD_2_Cl_2_ with solvent peaks at δ_H_ = 5.32 ppm and δ_C_ = 53.84 ppm. Splitting of ^1^H NMR spectra resonances were reported using abbreviations (s = singlet, d = doublet, appd = apparent doublet, dd = doublet of doublets, t = triplet, m = multiplet).

High-resolution mass spectra (HRMS) were obtained using Bruker micrOTOF-Q III spectrometer interfaced to a Dionex UltiMate 3000 LC for both electron spray ionization (ESI) and atmospheric pressure chemical ionization (APCI) measurements. Melting points (Mp) were measured using a Stuart SP-10-point apparatus and were left uncorrected. X-ray crystallography data collection details can be found below in the single X-ray crystallography data section.

Bicyclo­[1.1.1]­pentane-1,3-dicarboxylic acid **1** was purchased from Fluorochem; 3-(methoxycarbonyl)­bicyclo[1.1.1]­pentane-1-carboxylic acid **2**, 3-fluorobicyclo[1.1.1]­pentane 1-carboxylic acid **8a**, and 3-methylbicyclo[1.1.1]­pentane 1-carboxylic acid **8b** were provided by Enamine Ltd. All other chemicals were obtained from commercial suppliers and used without further purification unless specified. Dichloromethane (DCM) was dried over P_2_O_5_ and distilled before use. Fluka silica gel (high purity (w/Ca, ∼0.1%), pore size 60 Å, 230–400 mesh particle size (Sigma-Aldrich)) was utilized for column chromatography with the solvent systems specified in procedures. Flash chromatography was carried out on a Biotage Selekt Flash Purification System using preloaded silica columns: 40–60 μm particle size, mesh size: 230–400, pore size 60 Å. Reactions were monitored through thin layer chromatography with silica gel (Merck). For any reaction requiring heating, oil baths were used as heat sources.

#### (3-Methoxycarbonyl)­bicyclo­[1.1.1]­pentane-1-carboxylic Acid (**2**)

Synthesis modified from literature procedures.^61^ Trimethylsilyl chloride (1.27 mL, 10.0 mmol) was added dropwise to bicyclo[1.1.1]­pentane-1,3-dicarboxylic acid (1.560 g, 10.0 mmol) while stirring. Methanol (100 mL) was added to the solution and the reaction was stirred at room temperature overnight. Volatiles were removed under reduced pressure, yielding the dimethyl bicyclo[1.1.1]­pentane-1,3-dicarboxylate as white crystals (1.840 g, 10.0 mmol, >99%). ^1^H NMR (400 MHz, CDCl_3_, 298 K): δ = 3.72 (3H, s), 2.35 (6H, s) ppm. ^13^C­{^1^H} NMR (150 MHz, CDCl_3_, 298 K): δ = 169.7, 52.9, 37.6 ppm. Dimethyl bicyclo[1.1.1]­pentane-1,3-dicarboxylate was subjected to hydrolysis according to a literature procedure, yielding (3-methoxycarbonyl)­bicyclo[1.1.1]­pentane-1-carboxylic acid (0.975 g, 5.73 mmol, 57%).^2^ ^1^H NMR (400 MHz, CDCl_3_, 298 K): δ = 3.72 (s, 3H), 2.38 (s, 6H) ppm.

#### Methyl-3-(chlorocarbonyl)­bicyclo­[1.1.1]­pentane-1-carboxylate (**3**)

Synthesis modified from literature procedures.^62^ (3-Methoxycarbonyl)­bicyclo[1.1.1]­pentane-1-carboxylic acid (0.975 g, 5.73 mmol) was dissolved in diethyl ether (30 mL). Oxalyl chloride (0.98 mL, 11.5 mmol) and DMF (44 μL, 0.573 mmol) were added dropwise and stirred for 30 min at room temperature. Volatiles were removed under reduced pressure, yielding methyl-3-(chlorocarbonyl)­bicyclo[1.1.1]­pentane-1-carobxylate (1.07 g, 5.73 mmol, >99%).^1^H NMR (400 MHz, CDCl_3_, 298 K): δ = 3.71 (3H, s), 2.45 (6H, s).

#### Bicyclo­[1.1.1]­pentane-1,3-dicarbonyl Dichloride (**4**)

Synthesis modified from literature procedures.^62^ Bicyclo[1.1.1]­pentane-1,3-dicarboxylate (1.000 g, 6.410 mmol) was dissolved in diethyl ether (200 mL). Oxalyl chloride (2.2 mL, 25.64 mmol) and DMF (0.05 mL, 0.64 mmol) were added dropwise and stirred for 30 min at room temperature. Volatiles were removed under reduced pressure, yielding bicyclo[1.1.1]­pentane-1,3-dicarbonyl dichloride (1.237 g, 6.410 mmol, >99%) ^1^H NMR (400 MHz, CDCl_3_, 298 K): δ = 2.58 (s, 6H) ppm.

### Friedel–Crafts General Procedure A

Methyl (3-chlorocarbonyl)­bicyclo[1.1.1]­pentane-1-carboxylate (50 mg, 0.266 mmol) [unless stated otherwise] and aluminum trichloride (177 mg, 1.329 mmol) were dissolved in freshly distilled dichloromethane (10 mL), and cooled to 0 °C. Hydrocarbon (1 equiv., 0.266 mmol) was added and stirred at room temperature for 1 or 24 h, as specified for each compound. The reaction was quenched with ice H_2_O, phases were separated, and the organic phase was washed with H_2_O (3 × 50 mL), NaHCO_3_ (1 × 50 mL) and brine (1 × 50 mL), and dried with Na_2_SO_4_, and concentrated *in vacuo* and purified by precipitation or column chromatography on silica gel.

#### Methyl 3-(4-methoxybenzoyl)­bicyclo[1.1.1]­pentane-1-carboxylate (**6a**)

Reaction time of 1 h. The product was purified by recrystallization from DCM/hexane, yielding an off white solid (61 mg, 0.234 mmol, 88%; *R*_*f*_ = 0.3 (DCM); Mp = 101 °C; ^1^H NMR (400 MHz, CDCl_3_, 298 K): δ = 7.97 (d, *J* = 8.9 Hz, 2H), 6.93 (d, *J* = 8.9 Hz, 2H) 3.85 (s, 3H), 3.70 (s, 3H), 2.52 (s, 6H) ppm; ^13^C­{^1^H} NMR (101 MHz, CDCl_3_, 298 K): δ = 195.0, 170.1, 163.7, 131.3, 129.3, 113.9, 55.6, 54.6, 52.0, 43.8, 38.3, 31.1 ppm; HRMS (ESI) *m/z:* [M + Na]^+^ calcd for C_15_H_16_NaO_4_ 283.0941, Found: 283.0949. Crystal of **6a** were grown at room temperature via diffusion in 1:1 dichloromethane/hexane as the solvent and antisolvent, respectively.

#### Methyl 3-(4-Methylbenzoyl)­bicyclo[1.1.1]­pentane-1-carboxylate (**6b**)

Reaction time of 24 h, scale: methyl (3-chlorocarbonyl)­bicyclo[1.1.1]­pentane-1-carboxylate (1.500 mmol). The compound was purified by column chromatography on silica gel, eluting in a gradient of 0–100% DCM/ethyl acetate to afford a yellow-orange solid (210 mg, 0.861 mmol, 57*%*); Mp = 72 °C; *R*_*f*_ = 0.71 (DCM); ^1^H NMR (400 MHz, CD_2_Cl_2_, 298 K): δ = 7.88–7.86 (d, *J* = 8.3 Hz, 2H), 7.28–7.26 (d, *J* = 8.0, 2H), 3.69 (s, 3H), 2.51 (s, 6H), 2.40 (s, 3H) ppm; ^13^C­{^1^H} NMR (101 MHz, CD_2_Cl_2_, 298 K): δ = 196.3, 170.1, 144.5, 134.1, 129.6, 129.2, 54.7, 52.0, 44.1, 38.5, 21.8 ppm; HRMS (ESI) *m*/*z*: [M + Na]^+^ calcd for C_15_H_16_NaO_3_ 267.0992, Found 267.1001. Crystals of **6b** were grown at room temperature via diffusion in 1:1 dichloromethane/hexane as the solvent and antisolvent, respectively.

#### Methyl 3-(2,4-Dimethoxybenzoyl)­bicyclo[1.1.1]­pentane-1-carboxylate (**6c**)

Reaction time of 24 h. The compound was purified by chromatography on silica gel, eluting in a gradient of 0–90% DCM/ethyl acetate to afford a yellow-green oil (7 mg, 0.024 mmol, 51%); *R*_*f*_ = 0.3 (DCM); ^1^H NMR (400 MHz, CDCl_3_, 298 K): δ = 7.56–7.53 (d, *J* = 8.6 Hz, 1H), 6.51–6.49 (dd, *J* = 8.58 Hz, 2.3 Hz, 1H), 6.44–6.43 (d, *J* = 2.2 Hz, 1H), 3.86 (appd, 6H), 3.69 (s, 3H), 2.38 (s, 6H) ppm; ^13^C­{^1^H} NMR (101 MHz, CDCl_3_¸298 K): δ = 198.4, 170.6, 164.2, 159.8, 132.3, 121.0, 104.9, 98.3, 55.5, 55.2, 53.4, 51.8, 45.2, 37.5 ppm; HRMS (ESI) *m*/*z*: [M + Na]^+^ calcd for C_16_H_18_NaO_5_ 313.1046, Found: 313.1048. Crystals of **6c** was grown at room temperature via diffusion in 1:1 dichloromethane/hexane as the solvent and antisolvent, respectively.

#### Methyl 3-(3,4-Dimethylbenzoyl)­bicyclo[1.1.1]­pentane-1-carboxylate (**6d**)

Reaction time of 24 h. The compound was purified by flash chromatography on silica gel, eluting in a gradient of 25–100% hexane/DCM, to afford a light yellow solid (15 mg, 0.058 mmol, 22%); Mp = 70–72 °C; *R*_*f*_ = 0.5 (hexane/DCM = 3:1); ^1^H NMR (400 MHz, CDCl_3_, 298 K): δ = 7.74 (s, 1H), 7.72–7.70 (dd, *J* = 7.8 Hz, *J* = 1.6 Hz, 1H), 7.21–7.19 (d, *J* = 7.8 Hz 1H), 3.72 (s, 3H), 2.54 (s, 6H), 2.32 (app d, 6H) ppm; ^13^C­{^1^H} NMR (101 MHz, CDCl_3_, 298 K): δ = 196.4, 170.0, 142.9, 137.0, 134.1, 129.9, 129.8, 126.7, 54.5, 51.9, 43.8, 38.2, 22.7, 20.1, 19.8, 14.1 ppm; HRMS (APCI) *m/z:* [M + H]^+^ calcd for C_16_H_19_O_3_ 259.1329; Found 259.1330.

#### Methyl 3-(2,4-Dimethylbenzoyl)­bicyclo[1.1.1]­pentane-1-carboxylate (**6e**)

Reaction time of 24 h. The compound was purified by flash chromatography on silica gel, eluting in a gradient of 25–100% hexane/DCM, to afford a light yellow solid (14 mg, 0.054 mmol, 22%); Mp = 69–71 °C; *R*_*f*_ = 0.5 (hexane/DCM = 3:1); ^1^H NMR (400 MHz, CDCl_3_, 298 K): δ = 7.57–7.55 (d, *J* = 7.8 Hz, 1H), 7.07 (s, 1H), 7.05–7.03 (d, *J* = 8.0 Hz, 1H), 3.70 (s, 3H), 2.45 (s, 6H), 2.42 (s, 3H), 2.35 (s, 3H) ppm; ^13^C­{^1^H} NMR (101 MHz, CDCl_3_, 298 K): δ = 200.0, 170.1, 141.9, 138.7, 133.5, 132.9, 129.0, 125.9, 54.0, 51.8, 44.7, 37.9, 21.4, 21.0 ppm; HRMS (APCI) *m/z:* [M + H]^+^ calcd for C_16_H_19_O_3_ 259.1329; Found 259.1330.

#### Methyl 3-(2,5-Dimethylbenzoyl)­bicyclo[1.1.1]­pentane-1-carboxylate (**6f**)

Reaction time of 24 h, scale: methyl (3-chlorocarbonyl)­bicyclo[1.1.1]­pentane-1-carboxylate (1.00 mmol) The compound was purified by flash chromatography on silica gel, eluting in a gradient of 0–100% DCM/hexane to afford an off-white solid (149 mg, 0.577 mmol, 58%); Mp = 90 °C; *R*_*f*_ = 0.39 (hexane/DCM = 1:2); ^1^H NMR (400 MHz, CDCl_3_, 298 K): δ = 7.35 (m, 1H), 7.17 (m, 1H), 7.13 (m, 1H), 3.69 (s, 3H), 2.44 (s, 6H), 2.35 (appd, 6H) ppm; ^13^C­{^1^H} NMR (101 MHz, CDCl_3_, 298 K): δ *=* 201.0, 170.0, 136.6, 134.8, 134.6, 131.8, 131.7, 128.7, 53.8, 53.8, 53.8, 51.8, 44.6, 37.9, 21.0, 20.2 ppm; HRMS (ESI) *m/z:* [M + Na]^+^ calcd for C_16_H_18_NaO_3_ 281.1148; Found 281.1147.

#### Methyl 3-(2,3,5,6-Tetramethylbenzoyl)­bicyclo[1.1.1]­pentane-1-carboxylate (**6g**)

Reaction time of 24 h, scale: methyl (3-chlorocarbonyl)­bicyclo[1.1.1]­pentane-1-carboxylate (0.500 mmol). The compound was purified by flash chromatography on silica gel, eluting with 0–100% DCM/*n*-hexane to yield a white solid (95 mg, 0.332 mmol, 66%); *R*_*f*_ = 0.63 (DCM); ^1^H NMR (400 MHz, CDCl_3_, 298 K): δ = 6.94 (s, 1H), 3.67 (s, 3H), 2.30 (s, 6H), 2.18 (s, 6H), 2.01 (s, 6H) ppm; ^13^C­{^1^H} NMR (101 MHz, CDCl_3_, 298 K): δ = 209.0, 170.0, 139.7, 134.4, 131.8, 128.4, 52.7, 51.9, 45.4, 37.4, 19.4, 16.5 ppm. HRMS (ESI) *m*/*z*: [M + Na]^+^ calcd for C_18_H_22_NaO_3_: 309.1461, Found 309.1461.

#### Methyl 3-(2,3-Dihydrobenzo­[b]­[1,4]­dioxine-6-carbonyl)­bicyclo[1.1.1]­pentane-1-carboxylate (**6h**)

To a stirred suspension of AlCl_3_ (3550 mg, 26.50 mmol, 10.00 equiv) in CH_2_Cl_2_ (15 mL) was added 2,3-dihydro-1,4-benzodioxine (360 mg, 2.65 mmol, 1.00 equiv) dropwise at 0 °C. A solution of methyl (3-chlorocarbonyl)­bicyclo[1.1.1]­pentane-1-carboxylate (50 mg, 2.65 mmol, 1.00 equiv) in CH_2_Cl_2_ (5 mL) was added dropwise at 0 °C. The solution was stirred at 30 °C for 3 h, then poured into ice (100 mL), and adjusted to pH = 7 with NaHCO_3_. The mixture was extracted with CH_2_Cl_2_ (3 × 150 mL). The combined organic layers were dried over Na_2_SO_4_, filtered, and concentrated under reduced pressure. The crude product was recrystallized from a mixture of hexane/MeO*t*Bu, (9:1), yielding the product as a white solid (340 mg, 1.18 mmol, 44%); Mp = 87–88 °C; ^1^H NMR (500 MHz, CDCl_3_, 298 K): δ = 7.63–7.43 (m, 2H), 6.90 (d, *J* = 9.0 Hz, 1H), 4.32–4.26 (dd, *J* = 5.5 Hz, *J* = 2.4 Hz, 4H), 3.71 (s, 3H), 2.52 (s, 6H) ppm; ^13^C­{^1^H} NMR (126 MHz, CDCl_3_, 298 K): δ = 194.9, 170.1, 148.3, 143.4, 130.0, 123.2, 118.5, 117.4, 64.9, 64.2, 54.7, 51.9, 43.8, 38.3 ppm; HRMS (ESI-TOF) *m*/*z*: [M + H]^+^ calcd for C_16_H_17_O_5_^+^, 289.1071; Found 289.1057. The compound is now commercially available through Enamine Ltd. (www.enamine.net) under the code EN300–52506059.

#### Methyl 3-(4-Hydroxy-2,3-dimethoxybenzoyl)­bicyclo[1.1.1]­pentane-1-carboxylate (**6i**)

Reaction time of 24 h, scale: methyl (3-chlorocarbonyl)­bicyclo[1.1.1]­pentane-1-carboxylate (0.500 mmol). The crude was subjected to flash chromatography on silica gel, eluting with 0–100% DCM/*n*-hexane to yield a white solid (43 mg, 0.140 mmol, 29%); Mp = 120–121 °C; *R*_*f*_ = 0.48 (DCM); ^1^H NMR (400 MHz, CDCl_3_, 298 K): δ = 12.53 (s, 1H), 7.67 (d, *J* = 9.0 Hz, 1H), 6.50 (d, *J* = 9.0 Hz, 1H), 3.92 (s, 3H), 3.86 (s, 3H), 3.71 (s, 3H), 2.57 (s, 6H) ppm. ^13^C­{^1^H} NMR (101 MHz, CDCl_3_, 298 K): δ = 200.1, 169.7, 158.8, 157.8, 136.7, 127.2, 114.6, 103.0, 60.7, 56.2, 54.8, 51.9, 43.7, 38.3 ppm; HRMS (APCI) *m*/*z*: [M-H]^−^ calcd for C_16_H_17_O_6_ 305.1031, Found 305.1031.

#### Methyl 3-(4-Hydroxybenzoyl)­bicyclo[1.1.1]­pentane-1-carboxylate (**6j**)

Reaction time of 24 h, scale: methyl (3-chlorocarbonyl)­bicyclo[1.1.1]­pentane-1-carboxylate (2.7 mmol). The compound was subjected to flash chromatography on silica gel, eluting with 0–10% ethyl acetate/DCM to yield a colorless oil (165 mg, 0.670 mmol, 25%); ^1^H NMR (400 MHz; CDCl_3_, 298 K): δ = 7.94 (d, *J* = 8.8 Hz, 2H), 7.29 (bs, 1H), 6.92 (d, *J* = 8.8 Hz, 2H), 3.73 (s, 3H), 2.54 (s, 6H) ppm; ^13^C­{^1^H} NMR (101 MHz; CDCl_3_, 298 K): δ = 195.7, 170.5, 161.2, 131.7, 128.7, 115.6, 54.6, 52.1, 43.7, 38.2 ppm; HRMS (ESI) *m*/*z*: [M + H]^+^ calcd for C_14_H_13_O_4_ 245.0819, Found 245.0823.

#### Methyl 3-(Furan-2-carbonyl)­bicyclo[1.1.1]­pentane-1-carboxylate (**6k**)

Reaction time of 24 h, scale: **3** (0.532 mmol). The compound was purified by flash chromatography on silica gel, eluting in a gradient of 50–100% hexane/DCM to affording a brown solid (24 mg, 0.107 mmol, 20%); Mp = 86 °C; *R*_*f*_ = 0.38 (hexane/DCM = 1:1); ^1^H NMR (400 MHz, CDCl_3_, 298 K): δ = 7.60 (s, 1H), 7.21 (d, *J* = 3.6 Hz, 1H), 6.55–6.54 (q, *J* = 1.6 Hz, 1H), 3.70 (s, 3H), 2.49 (s, 6H) ppm; ^13^C­{^1^H} NMR (101 MHz, CDCl_3_, 298 K): δ = 185.1, 169.9, 152.2, 146.8, 118.4, 112.3, 53.6, 51.9, 42.5, 37.9 ppm; HRMS (APCI) *m*/*z*: [M + H]^+^ calcd for C_12_H_13_O_4_ 221.0808, Found: 221.0805. Crystals of **6k** were grown at room temperature via diffusion in 1:1 dichloromethane/hexane as the solvent and antisolvent, respectively.

#### Methyl 3-(Thiophene-2-carbonyl)­bicyclo[1.1.1]­pentane-1-carboxylate (**6l**)

Reaction time of 24 h. The compound was purified by flash chromatography on silica gel, eluting in a gradient of 25–33% hexane/ethyl acetate to afford a light brown solid (59 mg, 0.250 mmol, 0.94%). Large scale reaction (2.7 mmol) with a reaction time of 24 h yielded the light brown solid (510 mg, 80%); Mp = 74 °C; *R*_*f*_ = 0.48 (hexane: ethyl acetate = 2:1); ^1^H NMR (400 MHz, CDCl_3_, 298 K): δ = 7.80 (dd, *J* = 4.8 Hz, *J* = 2.8 Hz, 1H), 7.66 (dd, *J* = 6.0 Hz, *J* = 3.9 Hz, 1H), 7.14 (dd, *J* = 4.9 Hz, *J* = 3.9 Hz, 1H), 3.71 (s, 3H), 2.52 (s, 6H) ppm; ^13^C­{^1^H} NMR (101 MHz, CDCl_3_, 298 K): δ = 189.0, 169.8, 142.3, 134.0, 133.0, 133.0, 128.3, 128.2, 54.1, 52.0, 51.8, 43.3, 37.7 ppm; HRMS (APCI) *m/z:* [M + H]^+^ calcd for C_12_H_13_O_3_S 237.05780 Found: 237.0584. Crystal of **6l** were grown at room temperature via diffusion in 1:1 dichloromethane/hexane as the solvent and antisolvent, respectively.

#### Cyclopenta-2,4-dien-1-yl­(2-(3-(methoxycarbonyl)­bicyclo­[1.1.1]­pentane-1-carbonyl)­cyclopenta-2,4-dien-1-yl)­iron (**6m**)

Reaction time of 1 h. The compound was purified by flash chromatography on silica gel, eluting in a gradient of 75–100% hexane/ethyl acetate to afford a yellow-orange solid (25 mg, 0.075 mmol, 28%); Mp = 112 °C; *R*_*f*_ = 0.28 (ethyl acetate/hexane = 1:3); ^1^H NMR (400 MHz, CDCl_3_, 298 K): δ = 4.84 (2H, t, *J* = 1.8 Hz), 4.55 (2H, t, *J* = 1.8 Hz), 4.21 (5H, s), 3.73 (3H, s), 2.48 (6H, s) ppm; ^13^C­{^1^H} NMR (101 MHz, CDCl_3_, 298 K): δ = 200.7, 170.1, 72.5, 69.9, 69.8, 54.0, 51.9, 43.6, 37.5, 29.7 ppm; HRMS (APCI) *m/z:* [M + H]^+^ calcd for C_18_H_19_FeO_3_ 339.0678, Found: 339.0680. Crystals of **6m** were grown at room temperature via diffusion in 1:1 dichloromethane/hexane as the solvent and antisolvent, respectively.

#### Methyl 3-(Pyrene-1-carbonyl)­bicyclo[1.1.1]­pentane-1-carboxylate (**6n**)

Reaction time of 24 h, scale: methyl (3-chlorocarbonyl)­bicyclo[1.1.1]­pentane-1-carboxylate (0.532 mmol). The compound was purified by flash chromatography on silica gel, eluting in a gradient of 0–100% hexane/DCM to affording the product in fraction 4. Fraction 4 was then recrystallized in DCM/hexane, yielding the white solid (86 mg, 0.244 mmol, 46%); Mp = 127 °C; *R*_*f*_ = 0.12 (hexane/DCM = 1:1); ^1^H NMR (400 MHz, CDCl_3_, 298 K): δ = 8.67–8.65 (d, *J* = 9.3 Hz, 1H), 8.25–8.23 (d, *J* = 7.9 Hz, 1H), 8.18–8.17 (m, 1H), 8.16–8.12 (m, 3H), 8.09 (overlapped s, 1H), 8.07 (overlapped s, 1H), 8.00–7.97 (m, 2H) 3.70 (s, 3H), 2.59 (s, 6H) ppm; ^13^C­{^1^H} NMR (101 MHz, CDCl_3_, 298 K): δ = 201.0, 170.0, 133.6, 131.0, 130.5, 129.6, 129.6, 129.3, 127.0, 126.5, 126.3, 126.1, 126.0, 124.9, 124.5, 124.2, 123.7, 54.2, 52.8, 51.9, 45.3, 40.9, 37.9, 29.7 ppm; HRMS (APCI) *m/z:* [M + H]^+^ calcd for C_24_H_19_O_3_ 355.1329, Found: 355.1327. Crystals of **6n** were grown at room temperature via diffusion in 1:1 dichloromethane/hexane as the solvent and antisolvent, respectively.

#### Methyl 3-([1,1’-Biphenyl]-4-carbonyl)­bicyclo[1.1.1]­pentane-1-carboxylate (**6o**)

Reaction time of 1 h. The product was purified by recrystallization from DCM/hexane, yielding a white solid (70 mg, 0.229 mmol, 86%); Mp = 120 °C; ^1^H NMR (400 MHz, CDCl_3_, 298 K): δ = 8.06 (d, *J* = 8.2 Hz, 2H), 7.70 (d, *J* = 8.3 Hz, 2H), 7.65 (d, *J* = 7.4 Hz, 2H), 7.48 (t, *J* = 7.3 Hz, 2H), 7.44 (t, *J* = 7.6 Hz, 1H), 3.73 (s, 3H), 2.58 (s, 6H) ppm; ^13^C­{^1^H} NMR (101 MHz, CDCl_3_, 298 K): δ = 196.1, 169.9, 146.0, 139.8, 134.8, 129.5, 129.0, 128.3, 127.3, 127.3, 54.5, 51.9, 43.9, 38.3 ppm; HRMS (APCI) *m/z:* [M + H]^+^ calcd for C_20_H_19_O_3_ 307.1329 Found: 307.1331. Crystals of **6o** were grown at room temperature via diffusion in 1:1 dichloromethane/hexane as the solvent and antisolvent, respectively.

#### Methyl 3-([1,1“:4”,1’’-Terphenyl]-4-carbonyl)­bicyclo[1.1.1]­pentane-1-carboxylate (**6p**)

Reaction time of 24 h. The compound was purified by flash chromatography on silica gel, eluting in a gradient of 0–100% DCM/hexane, to afford a white solid (92 mg, 0.241 mmol, 48%) Mp = 210–212 °C; *R*_*f*_ = 0.43 (DCM); ^1^H NMR (400 MHz, CDCl_3_, 298 K): δ = 8.08 (d, *J* = 8.4 Hz 2H), 7.74 (d, *J* = 8.4 Hz, 2H), 7.71 (s, 4H), 7.66 (d, *J* = 7.8 Hz, 2H), 7.48 (t, *J* = 7.8 Hz, 2H), 7.38 (t, *J* = 7.8 Hz, 1H), 3.73 (s, 3H), 2.60 (s, 6H) ppm; ^13^C­{^1^H} NMR (101 MHz, CDCl_3_, 298 K): δ = 196.1, 169.9, 145.4, 141.2, 140.4, 138.6, 134.8, 129.5, 129.5, 128.9, 128.9, 127.7, 127.7, 127.6, 127.1, 127.0, 127.0, 54.6, 54.6, 54.5, 54.5, 54.5, 51.9, 43.9, 38.3 ppm; HRMS (ESI) *m/z:* [M + H]^+^ calcd for C_26_H_22_NaO_3_ 405.1461; Found 405.1449.

#### Dimethyl 3,3′-(Carbazole-1,6-dicarbonyl)­bis­(bicyclo[1.1.1]­pentane-1-carboxylate) (**6q**)

Reaction time of 24 h, scale: methyl (3-chlorocarbonyl)­bicyclo[1.1.1]­pentane-1-carboxylate (0.532 mmol). The solution was recrystallized from DCM/hexane. The hexane layer was purified by flash chromatography on silica gel, eluting in a gradient of 33–100% hexane/DCM to afford the product in fraction four. Fraction four was purified by flash chromatography on silica gel, eluting the product in a gradient of hexane/DCM to DCM, removing the product with ethyl acetate 100% as a light yellow solid (7 mg, 0.015 mmol, 6%); Mp = 257 °C; *R*_*f*_ = 0.49 (hexane/DCM = 1:2); ^1^H NMR (400 MHz, CDCl_3_, 298 K): δ = 10.76 (s, 1H, NH), 8.77 (s, 1H), 8.34 (d, *J* = 7.7 Hz, 1H), 8.19–8.17 (dd, *J* = 7.7 Hz, *J* = 0.9 Hz, 1H, overlapped), 8.17–8.15 (dd, *J* = 8.37 Hz, *J* = 1.46 Hz, 1H, overlapped), 7.55 (d, *J* = 8.77 Hz, 1H), 7.35 (t, *J* = 7.7 Hz, 1H), 3.750 (s, 3H, overlapped), 3.745 (s, 3H, overlapped), 2.67 (s, 6H), 2.64 (s, 6H) ppm; ^13^C­{^1^H} NMR (101 MHz, CDCl_3_, 298 K): δ = 197.9, 195.7, 169.8, 142.7, 140.1, 129.1, 128.8, 127.9, 126.7, 125.2, 122.4, 122.1, 119.4, 118.8, 111.1, 54.8, 54.7, 52.0, 51.9, 44.0, 44.0, 38.3, 38.3 ppm; HRMS (APCI) *m/z:* [M + H] calcd for C_28_H_26_NO_6_ 472.1755, Found 472.1757.

#### Dimethyl 3,3′-(Carbazole-3,6-dicarbonyl)­bis­(bicyclo[1.1.1]­pentane-1-carboxylate) (**6r**)

Reaction time of 24 h, scale: methyl (3-chlorocarbonyl)­bicyclo[1.1.1]­pentane-1-carboxylate (0.532 mmol). The compound was purified by flash chromatography on silica gel, eluting in a gradient of 50–100% hexane/DCM to affording the product in the second fraction. The second fraction was purified by flash chromatography on silica gel, eluting the product in a gradient of 50–100% DCM/ethyl acetate to afford a yellow solid (7 mg, 0.015 mmol, 6%); Mp = 256 °C; *R*_*f*_ = 0.68 (hexane/ethyl acetate = 1:1); ^1^H NMR (400 MHz, CDCl_3_, 298 K): δ = 8.79 (s, 2H), 8.74 (s, 1H), 8.17–8.14 (dd, *J* = 8.9 Hz, *J* = 1.7 Hz, 2H), 7.51–7.49 (d, *J* = 8.7 Hz, 2H), 3.75 (s, 6H), 2.64 (s, 12H) ppm; ^13^C­{^1^H} NMR (101 MHz, CDCl_3_, 298 K): δ = 196.2, 170.5, 143.3, 129.7, 128.4, 123.8, 122.9, 111.3, 55.1, 53.8, 52.3, 44.4, 38.7 ppm; HRMS (ESI) *m/z:* [M]^−^ calcd for C_28_H_24_NO_6_ 470.1609, Found: 470.1614. Crystals of **6r** were grown at room temperature via diffusion in 1:1 dichloromethane/hexane as the solvent and antisolvent, respectively.

#### Methyl (S)-3-((1-Methoxy-1-oxo-3-phenylpropan-2-yl)­carbamoyl)­bicyclo[1.1.1]­pentane-1-carboxylate (**6s**)

Reaction time of 24 h. Concentration of the reaction mixture after washing yielded the pure white solid product (61 mg, 0.266 mmol, 70%); Mp = 100 °C; ^1^H NMR (400 MHz, CDCl_3_, 298 K): δ = 7.31–7.24 (m, 3H), 7.07–7.05 (d, *J* = 7.9 Hz, 2H), 4.88–4.83 (dt, *J* = 7.9 Hz, *J* = 5.9 Hz, 1H), 3.74 (s, 3H), 3.68 (s, 3H), 3.09–3.06 (dq, *J* = 16.1 Hz, *J* = 6.2 Hz, 2H, 2.24 (s, 6H); ^13^C­{^1^H} NMR (150 MHz, CDCl_3_, 298 K): δ = 171.8, 169.7, 168.4, 135.6, 129.3, 128.6, 127.3, 52.7, 52.4, 52.2, 51.8, 39.1, 37.7, 36.8; HRMS (APCI) *m*/*z* [M + H]^+^ calcd for C_18_H_22_NO_5_ 332.1492, Found 332.1496.

#### 1-Methyl-3-phenyl Bicyclo[1.1.1]­pentane-1,3-dicarboxylate (**6t**)

Reaction time 24 h, scale (2.7 mmol). The compound was purified by flash chromatography on silica gel, eluting in a gradient of 0–100% *n-*hexane/DCM to affording a colorless oil (159 mg, 24%); *R*_*f*_ = 0.56 (DCM); ^1^H NMR (400 MHz, CDCl_3_, 298 K): δ = 7.37 (t, *J* = 7.5 Hz, 2H), 7.22 (t, *J* = 7.5 Hz, 1H), 7.08 (d, *J* = 7.5 Hz, 2H), 3.71 (s, 3H), 2.45 (s, 6H); ^13^C­{^1^H} NMR (101 MHz, CDCl_3_, 298 K): δ = 169.6, 167.5, 150.4, 129.5, 126.0, 121.4, 53.1, 51.9, 37.9, 37.8; HRMS (APCI) *m/z:* [M + Na]^+^ calcd for C_14_H_14_NaO_4_ 269.0784, Found 269.0787.

### Friedel–Crafts General Procedure B

Bicyclo­[1.1.1]­pentane 1,3-dicarbonyl dichloride (unless otherwise stated) (193 mg, 1 mmol), aluminum trichloride (1330 mg, 10 mmol) were dissolved in freshly distilled dichloromethane (20 mL) and cooled to 0 °C. Aromatic compound (2 equiv., 2 mmol) was added, and the mixture was stirred at room temperature for 1–24 h, as specified for each compound. The crude was washed with H_2_O (3 × 50 mL), dried with Na_2_SO_4_, concentrated *in vacuo* and purified by crystallization or column chromatography on silica gel.

#### Bicyclo­[1.1.1]­pentane-1,3-diylbis­((4-methoxyphenyl)­methanone (**7a**)

Reaction time of 1 h, scale: bicyclo[1.1.1]­pentane 1,3-dicarbonyl dichloride (1.30 mmol). The product was purified by precipitation with *n*-hexane and DCM, yielding a white solid (222 mg, 0.660 mmol, 51%); Mp = 151–154 °C; ^1^H NMR (400 MHz, CDCl_3_, 298 K): δ = 8.05–8.02 (d, *J* = 9.0,4H), 6.97–6.95 (d, *J* = 9.0, 4H), 3.89 (s, 6H), 2.77 (s, 6H) ppm; ^13^C­{^1^H} NMR (101 MHz, CDCl_3_, 298 K): δ *=* 195.3, 163.6, 131.3, 129.3, 113.8, 56.2, 55.5, 44.3 ppm; HRMS (ESI) *m/z:* [M + H]^+^ calcd for C_21_H_21_O_4_ 337.1434 Found 337.1439. Crystals of **7a** were grown at room temperature via slow evaporation of d-chloroform.

#### 1,3-Bis­(*p*-methylbenzoyl)-bicyclo­[1.1.1]­pentane (**7b**)

Reaction time of 1 h, scale: bicyclo[1.1.1]­pentane 1,3-dicarbonyl dichloride (4.62 mmol). The compound was purified by flash chromatography on silica gel, eluting in a gradient of 0–100% hexane/DCM, to afford a yellow solid (64 mg, 2.08 mmol, 45%); Mp = 126 °C; *R*_*f*_ = 0.11 (hexane/DCM = 1:1); ^1^H NMR (400 MHz, CD_2_Cl_2_, 298 K): δ = 7.90 (d, *J* = 7.8 Hz, 4H), 7.29 (d, *J* = 7.8 Hz, 4H), 2.71 (s, 6H), 2.40 (s, 6H) ppm; ^13^C­{^1^H} NMR (101 MHz, CD_2_Cl_2_, 298 K): δ = 196.6, 144.6, 134.2, 129.6, 129.3, 56.4, 44.7, 21.8 ppm; HRMS (APCI) *m/z:* [M + H]^+^ calcd for C_21_H_21_O_2_ 305.1536, Found: 305.1530. Crystals of **7b** were grown at room temperature via diffusion in 1:1 dichloromethane/hexane as the solvent and antisolvent, respectively.

#### Bicyclo­[1.1.1]­pentane-1,3-diylbis­((3,4-dimethylphenyl)­methanone) (**7c**)

Reaction time of 24 h, scale: bicyclo[1.1.1]­pentane 1,3-dicarbonyl dichloride (0.331 mmol). The compound was purified by flash chromatography on silica gel, eluting in a gradient of 0–100% hexane/DCM, to afford a pale-yellow oil (42 mg, 0.126 mmol, 38%); *R*_*f*_ = 0.5 (DCM); ^1^H NMR (400 MHz, CDCl_3_, 298 K): δ *=* 7.81 (m, 4H), 7.23 (d, *J* = 7.8 Hz, 2H), 2.78 (s, 6H), 2.35 (s, 12H) ppm; ^13^C­{^1^H} NMR (101 MHz, CDCl_3_, 298 K): δ *=* 196.9, 142.9, 137.1, 134.2, 129.9, 129.8, 126.7, 56.2, 44.4, 20.1, 19.9 ppm; HRMS (APCI) *m/z:* [M + H]^+^ calcd for C_23_H_25_O_2_ 333.1849; Found 333.1843.

#### Bicyclo­[1.1.1]­pentane-1,3-diylbis­((2,4-dimethylphenyl)­methanone) (**7d**)

Reaction time of 24 h, scale: bicyclo[1.1.1]­pentane 1,3-dicarbonyl dichloride (0.336 mmol). The compound was purified by flash chromatography on silica gel, eluting in a gradient of 0–100% hexane/DCM, to afford a pale yellow oil (49 mg, 0.147 mmol, 44%); *R*_*f*_ = 0.43 (DCM); ^1^H NMR (400 MHz, CDCl_3_, 298 K): δ *=* 7.62 (d, *J* = 7.8 Hz, 2H), 7.10 (s, 2H), 7.08 (d, *J* = 7.9 Hz, 2H), 2.61 (s, 6H), 2.45 (s, 6H), 2.37 (s, 6H) ppm; ^13^C­{^1^H} NMR (101 MHz, CDCl_3_, 298 K): δ *=* 200.6, 141.8, 138.5, 133.7, 132.8, 128.9, 125.9, 55.1, 44.9, 21.4, 21.0 ppm; HRMS (APCI) *m/z:* [M + H]^+^ calcd for C_23_H_25_O_2_ 333.1849; Found 333.1857.

#### Bicyclo­[1.1.1]­pentane-1,3-diylbis­((2,5-dimethylphenyl)­methanone) (**7e**)

Reaction time of 24 h, scale: bicyclo[1.1.1]­pentane-1,3-dicarbonyl dichloride (0.466 mmol). The compound was purified by flash chromatography on silica gel, eluting in a gradient of 0–100% hexane/DCM, to afford a brown oil (85 mg, 0.256 mmol, 55%); *R*_*f*_ = 0.50 (hexane/DCM = 1:1); ^1^H NMR (400 MHz, CDCl_3_, 298 K): δ *=* 7.39 (d, *J* = 1.6 Hz, 2H), 7.19 (dd, *J* = 7.8 Hz, *J* = 1.6 Hz, 2H), 7.16 (d, *J* = 7.8 Hz, 2H), 2.59 (s, 6H), 2.39 (appd, 12H) ppm; ^13^C­{^1^H} NMR (101 MHz, CDCl_3_, 298 K): δ *=* 201.6, 136.8, 134.8, 134.5, 131.8, 131.7, 128.6, 54.7, 44.9, 21.0, 20.1 ppm; HRMS (APCI) *m/z:* [M + H]^+^ calcd for C_23_H_25_O_2_ 333.1849; Found 333.1848.

#### Bicyclo­[1.1.1]­pentane-1,3-diylbis­((2,3,5,6-tetramethylphenyl)­methanone) (**7f**)

Reaction time of 24 h, scale: bicyclo[1.1.1]­pentane 1,3-dicarbonyl dichloride (10 mmol). The crude was subjected to flash chromatography on silica gel, eluting with 0–100% DCM/*n*-hexane, and then precipitated with *n*-hexane from a DCM solution to yield a white solid (101 mg, 48%). Mp = 271–273 °C; *R*_*f*_ = 0. 56 (DCM); ^1^H NMR (400 MHz, CDCl_3_, 298 K): δ = 6.94 (s, 2H), 3.67 (s, 6H), 2.30 (s, 12H), 2.18 (s, 12H), 2.01 (s, 12H) ppm. ^13^C­{^1^H} NMR (101 MHz, CDCl_3_, 298 K): δ *=* 209.1, 139.8, 134.4, 131.8, 128.4, 52.6, 45.1, 19.4, 16.5 ppm. HRMS (ESI) *m*/*z*: [M + Na]^+^ calcd for C_27_H_32_NaO_2_ 411.2295; found 411.2298.

#### Bicyclo­[1.1.1]­pentane-1,3-diylbis­(furan-2-ylmethanone) (**7g**)

Reaction time of 24 h. The compound was purified by flash chromatography on silica gel, eluting in a gradient of 0–10% DCM/ethyl acetate, to afford a colorless oil (90 mg, 0.352 mmol, 36%). *R*_*f*_ = 0.72 (DCM/ethyl acetate = 9:1); ^1^H NMR (400 MHz, CD_2_Cl_2_, 298 K): δ = 7.70 (dd, *J* = 1.7 Hz, *J* = 0.7 Hz, 2H), 7.30 (dd, *J* = 3.7 Hz, *J* = 0.7 Hz, 2H), 6.62 (dd, *J* = 3.7 Hz, *J* = 1.7 Hz, 2H), 2.67 (s, 6H) ppm; ^13^C­{^1^H} NMR (101 MHz, CD_2_Cl_2_, 298 K): δ *=* 185.0, 152.2, 146.8, 118.3, 112.2, 54.3, 42.6 ppm; HRMS (APCI) *m/z:* [M+K]^+^ calcd for C_15_H_12_KO_4_ 295.0367; Found 295.0375.

#### Bicyclo­[1.1.1]­pentane-1,3-diylbis­(thiophen-2-ylmethanone) (**7h**)

Reaction time of 1 h: yield 17%. Reaction time of 24 h: yield 54%, scale: bicyclo[1.1.1]­pentane 1,3-dicarbonyl dichloride (1.000 mmol). The product was purified by precipitation with *n*-hexane and DCM, yielding an off-white solid (156 mg, 0.541 mmol, 54%); Mp = 122–125 °C; *R*_*f*_ = 0.30 (DCM); ^1^H NMR (400 MHz, CDCl_3_, 298 K): δ = 7.88 (dd, *J* = 3.7 Hz, *J* = 3.05 Hz, 2H), 7.71 (dd, *J* = 4.9 Hz, *J* = 4.2 Hz, 2H), 7.19 (dd, *J* = 4.7 Hz, *J* = 4.0 Hz 2H), 2.76 (s, 6H) ppm; ^13^C­{^1^H} NMR (101 MHz, CDCl_3_, 298 K): δ = 189.2, 142.3, 134.1, 133.1, 128.3, 55.4, 54.1, 43.4 ppm; HRMS (ESI) *m/z:* [M + Na]^+^ calcd for C_15_H_12_NaO_2_S_2_ 311.0171; Found 311.0171.

#### Bicyclo­[1.1.1]­pentane-1,3-diylbis­([1,1’-biphenyl]-4-ylmethanone) (**7i**)

Reaction time of 1 h, scale: bicyclo[1.1.1]­pentane 1,3-dicarbonyl dichloride (1.000 mmol). The compound was purified by flash chromatography on silica gel, eluting in a gradient of 50–100% DCM/*n*-hexane, to afford an off-white solid (121 mg, 0.282 mmol, 28%); Mp = 261 °C; *R*_*f*_ = 0.53 (DCM); ^1^H NMR (400 MHz, CDCl_3_, 298 K): δ = 8.15 (d, *J* = 8.4 Hz, 4H), 7.74 (d, *J* = 8.4 Hz, 4H), 7.67 (d, *J* = 7.5 Hz, 4H), 7.51 (t, *J* = 7.5 Hz, 4H), 7.44 (t, *J* = 7.5 Hz, 2H), 2.87 (s, 6H) ppm; ^13^C­{^1^H} NMR (101 MHz, CDCl_3_, 298 K): δ = 196.5, 146.0, 139.8, 134.9, 129.5, 129.0, 128.4, 127.4, 127.3, 127.3, 56.2, 44.5 ppm; HRMS (APCI) *m/z:* [M + H]^+^ calcd for C_31_H_25_O_2_ 429.1849; Found 429.1851.

#### Bicyclo­[1.1.1]­pentane-1,3-diylbis­([1,1“:4”,1’’-terphenyl]-4-ylmethanone) (**7j**)

Reaction time of 1 h, scale: bicyclo[1.1.1]­pentane 1,3-dicarbonyl dichloride (0.302 mmol). The product was purified by precipitation with *n*-hexane from DCM, followed by precipitation with methanol from DCM, yielding a white solid (98 mg, 0.169 mmol, 56%); Mp = 261 °C; *R*_*f*_ = 0.76 (DCM); ^1^H NMR (400 MHz, CDCl_3_, 298 K): δ = 8.14 (d, *J* = 7.8 Hz, 4H), 7.76 (d, *J* = 7.8 Hz, 4H), 7.73 (m, 8H), 7.66 (d, *J* = 7.5 Hz, 4H), 7.48 (t, *J* = 7.5 Hz, 4H), 7.39 (t, *J* = 7.5 Hz, 2H), 2.89 (s, 6H) ppm; ^13^C­{^1^H} NMR (101 MHz, CDCl_3_, 298 K): δ *=* 196.4, 145.4, 141.3, 140.3, 138.6, 134.9, 129.6, 128.9, 127.7, 127.6, 127.1, 127.1, 56.2, 44.6 ppm; HRMS (APCI) *m*/*z* [M + H]^+^ calcd for C_43_H_33_O_2_ 581.2475 Found 581.2482.

#### Bicyclo­[1.1.1]­pentane-1,3-diylbis­([1,1’-ferrocenyl]-4-ylmethanone) (**7k**)

Reaction time of 24 h, scale: bicyclo[1.1.1]­pentane 1,3-dicarbonyl dichloride (0.616 mmol). The product was purified by precipitation with *n*-hexane and DCM, yielding a red solid (167 mg, 0.339 mmol, 55%); Mp = 190 °C (decomposed); *R*_*f*_ = 0.71 (DCM/ethyl acetate = 9.5:0.5); ^1^H NMR (400 MHz; CDCl_3_, 298 K): δ = 4.88 (m, 4H), 4.56 (m, 4H), 4.23 (s, 10H), 2.63 (s, 6H) ppm; ^13^C­{^1^H} NMR (101 MHz; CDCl_3_, 298 K): δ *=* 201.4, 70.0, 69.9, 55.2, 43.6 ppm; HRMS (ESI) *m/z:* [M + H]^+^ calcd for C_27_H_25_Fe_2_O_2_ 493.0549; Found 493.0540.

#### Bicyclo­[1.1.1]­pentane-1,3-diylbis­(naphthalen-1-ylmethanone) (**7l**)

Reaction time of 1 h. The product was purified by precipitation with *n*-hexane and DCM, yielding a off white solid (297 mg, 0.789 mmol, 60%); Mp = 126–133 °C; ^1^H NMR (400 MHz, CDCl_3_, 298 K) *δ =* 8.38 (d, *J* = 8.3 Hz, 2H), 7.99 (d, *J* = 8.2 Hz, 2H), 7.88 (d, *J* = 7.9 Hz, 2H), 7.84 (d, *J* = 7.1 Hz, 2H), 7.53 (m, 6H), 2.70 (s, 6H); ^13^C­{^1^H} NMR (101 MHz; CDCl_3_, 298 K): δ *=* 201.2, 134.6, 133.9, 132.3, 130.2, 128.5, 127.9, 127.3, 126.6, 125.6, 124.2, 55.1, 45.1; HRMS (APCI) *m/z:* [M + Na]^+^ calcd for C_27_H_20_NaO_2_ 399.1356; Found 399.1356.

#### Bicyclo­[1.1.1]­pentane-1,3-diylbis­((8,10-dihydropyren-1-yl)­methanone) (**7m**)

Reaction time of 24 h, scale: bicyclo[1.1.1]­pentane 1,3-dicarbonyl dichloride (1.000 mmol). The product was purified by precipitation with *n*-hexane and DCM, yielding a red-brown solid (230 mg, 0.438 mmol, 44%); Mp = 239 °C; *R*_*f*_ = 0.80 (hexane/DCM = 1:2); ^1^H NMR (400 MHz, CDCl_3_, 298 K): δ *=* 8.72 (d, *J* = 9.4 Hz, 2H), 8.35 (d, *J* = 8.2 Hz, 2H), 8.27 (d, *J* = 7.6 Hz, 4H), 8.20 (m, 6H), 8.10 (m, 4H), 2.89 (s, 6H) ppm; ^13^C­{^1^H} NMR (101 MHz, CDCl_3_, 298 K): δ *=* 201.6, 133.6, 131.3, 131.1, 130.6, 129.7, 129.6, 129.4, 127.1, 126.5, 126.4, 126.1, 125.9, 124.6, 124.3, 123.7, 55.5, 45.6 ppm; HRMS (APCI) *m/z:* [M + H]^+^ calcd for C_39_H_25_O_2_ 525.1849; Found 525.1855.

### Reactivity of Mono- and Diketones

#### (3-Methylbicyclo­[1.1.1]­pentan-1-yl)­(thiophen-2-yl)­methanone (**9b**)

Compound **8b** (63 mg, 0.5 mmol) was dissolved in 10 mL of dry DCM. Oxalyl chloride (85 μL, 1.0 mmol) and DMF (1 drop) were added, and the solution was stirred at RT for 1 h. Afterward the solution was cooled to 0 °C, AlCl_3_ (333 mg, 2.5 mmol) and thiophene (50 μL, 0.6 mmol) were added, and the mixture was stirred overnight, slowly warming up to RT. The reaction was quenched with ice H_2_O, washed with H_2_O, NaHCO_3_ and brine, dried with Na_2_SO_4_, filtered and concentrated. The crude was purified by flash chromatography on silica gel, eluting with 0–100% DCM/*n*-hexane to yield a brown oil (62 mg, 0.323 mmol, 65% over 2 steps). *R*_*f*_ = 0.75 (DCM); ^1^H NMR (400 MHz; CDCl_3_, 298 K): δ 7.82 (dd, *J* = 3.8 Hz, *J* = 1.1 Hz, 1H), 7.62 (dd, *J* = 5.1 Hz, *J* = 1.1 Hz, 1H), 7.13 (dd, *J* = 5.1 Hz, *J* = 3.8 Hz, 1H), 2.12 (s, 6H), 1.23 (s, 3H) ppm. ^13^C­{^1^H} NMR (101 MHz; CDCl_3_): δ 190.3, 143.0,133.4, 132.8, 128.0, 54.7, 43.6, 36.7, 18.0 ppm. HRMS (ESI) *m*/*z*: [M + Na]^+^ calcd for C_11_H_12_NaOS: 215.0501, Found 215.0501.

#### Methyl 3-(hydroxy­(thiophen-2-yl)­methyl)­bicyclo[1.1.1]­pentane-1-carboxylate (**10**)

NaBH_4_ (76 mg, 2.0 mmol) was added in portions to a stirred solution of **6o** (50 mg, 0.2 mmol) in methanol (10 mL) at 0 °C. After the addition was completed, the mixture was stirred at 0 °C for 30 min, then diluted with DCM, washed with NaHCO_3_ (1 × 30 mL), dried with Na_2_SO_4_, filtered and concentrated, yielding the carbinol spectroscopically pure as a colorless oil (38 mg, 0.159 mmol, 80%); *R*_*f*_ = 0.71 (DCM/ethyl acetate = 5:1); ^1^H NMR (400 MHz; CDCl_3_, 298 K): δ = 7.24 (dd, *J* = 1.0 Hz, *J* = 5.1 Hz, 1H), 6.98 (dd, *J* = 5.1 Hz, *J* = 3.4 Hz, 1H), 6.91 (dd, *J* = 1.0 Hz, *J* = 5.1 Hz, 1H), 4.98 (s, 1H), 3.65 (s, 3H), 1.96 (ddd, *J* = 1.4 Hz, *J* = 9.2 Hz, *J* = 24.2 Hz, 6H) ppm; ^13^C­{^1^H} NMR (101 MHz, CDCl_3_, 298 K): δ *=* 170.6, 144.7, 126.6, 124.7, 124.1, 69.7, 51.7, 49.6, 43.0, 37.6 ppm; HRMS (ESI) *m/z:* [M + Na]^+^ calcd for C_12_H_14_NaO_3_S 261.0556, Found 261.0559.

#### 3-(Thiophene-2-carbonyl)­bicyclo­[1.1.1]­pentane-1-carboxylic acid (**11**)

A solution of **10** (50 mg, 0.2 mmol) and NaOH (33 mg, 0.83 mmol) in MeOH (10 mL) was refluxed for 1 h, then diluted with DCM and washed with H_2_O. The aqueous phase was acidified to pH = 2 with 1 M HCl and washed with fresh DCM (10 mL). The organic layer was dried with Na_2_SO_4_, filtered and concentrated to afford the product as a white solid (38 mg, 0.171 mmol, 86%); Mp = 148–150 °C; ^1^H NMR (400 MHz; CDCl_3_, 298 K): δ = 7.84 (dd, *J* = 0.9 Hz, *J* = 3.8 Hz, 1H), 7.70 (dd, *J* = 0.9 Hz, *J* = 4.9 Hz, 1H), 7.18 (dd, *J* = 4.9 Hz, *J* = 3.8 Hz, 1H), 2.59 (s, 6H) ppm; ^13^C­{^1^H} NMR (101 MHz; CDCl_3_, 298 K): δ *=* 188.8, 174.6, 142.2, 134.2, 133.1, 128.3, 54.1, 43.2, 37.6 ppm; HRMS (ESI) *m/z:* [M + H]^+^ calcd for C_11_H_10_NaO_3_S 245.0243; Found 245.0239.

#### 3-(Thiophene-2-carbonyl)­bicyclo­[1.1.1]­pentane-1-carbonyl chloride (**12**)

Oxalyl chloride (26 μL, 0.3 mmol) and DMF (2 μL, 0.02 mmol) were added to a stirred solution of **11** (38 mg, 0.17 mmol) in diethyl ether (10 mL). The mixture was stirred at RT for 1 h, concentrated and dried under vacuum to afford the product as colorless oil (44 mg, 0.18 mmol, 100%); ^1^H NMR (400 MHz, CDCl_3_, 298 K): δ = 7.82 (dd, *J* = 0.9 Hz, *J* = 3.8 Hz, 1H), 7.72 (dd, *J* = 0.9 Hz, *J* = 4.9 Hz, 1H), 7.19 (dd, *J* = 4.9 Hz, *J* = 3.8 Hz, 1H), 2.67 (s, 6H) ppm; ^13^C­{^1^H} NMR (101 MHz, CDCl_3_, 298 K): δ *=* 187.9, 170.6, 141.9, 134.5, 133.2, 128.4, 55.0, 54.1, 45.3, 42.6 ppm. Note: HMRS not obtained for compound 8 due to instability of acid chlorides.

#### (3-(4-Methylbenzoyl)­bicyclo­[1.1.1]­pentan-1-yl)­(thiophen-2-yl)­methanone (**13**)

Toluene (50 μL, 0.47 mmol) was added to a suspension of **12** (45 mg, 0.17 mmol) and AlCl_3_ (113 mg, 0.85 mmol) in freshly distilled DCM (10 mL) at 0 °C. Upon addition the cooling bath was removed, and the reaction was stirred at RT for 24 h and poured into ice H_2_O. The phases were separated, the organic phase was washed with H_2_O (3 × 20 mL), dried with Na_2_SO_4_ and concentrated. The compound was purified by flash chromatography on silica gel, eluting in a gradient of 50–100% DCM/hexane, to afford a colorless oil (52 mg, 0.175 mmol, 43%).; *R*_*f*_ = 0.43 (DCM); ^1^H NMR (400 MHz, CDCl_3_, 298 K): δ = 7.94 (d, *J* = 8.2 Hz, 2H), 7.88 (dd, *J* = 0.9 Hz, *J* = 3.9 Hz, 1H), 7.70 (dd, *J* = 0.9 Hz, *J* = 5.0 Hz, 1H), 7.29 (d, *J* = 8.2 Hz, 2H), 7.19 (dd, *J* = 5.0 Hz, *J* = 3.9 Hz, 1H), 2.77 (s, 6H), 2.44 (s, 3H) ppm; ^13^C­{^1^H} NMR (101 MHz, CDCl_3_, 298 K): δ *=* 196.4, 189.4, 144.2, 142.3, 134.1, 133.6, 133.1, 129.3, 129.0, 128.3, 55.8, 54.1, 43.9, 43.9, 21.7 ppm; HRMS (ESI) *m/z:* [M + Na]^+^ calcd for C_18_H_16_NaO_2_S 319.0763; Found 319.0760.

#### Dimethyl 3,3′-((1R,2S) 1,2-Dihydroxy-1,2-di-*p*-tolylethane-1,2-diyl)­bis­(bicyclo­[1.1.1]­pentane-1-carboxylate) and Dimethyl 3,3′-((1S,2R) 1,2-Dihydroxy-1,2-di-*p*-tolylethane-1,2-diyl)­bis­(bicyclo­[1.1.1]­pentane-1-carboxylate) (**14a**)

Synthesis modified from literature procedures.^63^ Activated zinc powder (324 mg, 4.95 mmol) was added to anhydrous THF (15 mL), purged with argon, and cooled to 0 °C. Titanium tetrachloride (0.27 mL, 2.45 mmol) was added, and the solution was warmed to room temperature. The solution stirred for 30 min at room temperature then refluxed for 2.5 h. The mixture was cooled to 0 °C then **6b** (100 mg, 0.409 mmol) in THF (5 mL) was added dropwise. The solution was refluxed for 12 h. The solution was quenched with 10% K_2_CO_3_ (50 mL), extracted with Et_2_O (3 × 20 mL), dried with Na_2_SO_4_, and concentrated *in vacuo*. The product was purified by flash chromatography with a gradient of 50–100% hexane/DCM yielding an off white solid (16 mg, 0.033 mmol, 16%); *R*_*f*_ = 0.5 (DCM); Mp = 180–182 °C; ^1^H NMR (600 MHz, CD_2_Cl_2_ 298 K): δ *=* 7.72 (d, *J* = 8.0 Hz, 2H), 7.64 (d, *J* = 8.0 Hz, 2H), 7.23 (d, *J* = 7.8 Hz, 2H), 7.14 (d, *J* = 7.8 Hz, 2H), 3.56 (s, 6H), 2.38 (s, 6H), 1.71–1.70 (dd, *J* = 9.3 Hz, *J* = 1.6 Hz, 6H), 1.55–1.53­(dd, *J* = 9.3 Hz, *J* = 1.6 Hz, 6H) ppm; ^13^C­{^1^H} NMR (150 MHz, CDCl_3_, 298 K): δ *=* 170.5, 138.8, 136.7, 129.0, 128.0, 127.8, 126.6, 51.5, 50.6, 45.7, 37.6., 21.0 ppm; HRMS (APCI) *m/z:* [M]^−^ calcd for C_30_H_33_O_6_ 489.2283; Found 489.2284.

#### Dimethyl 3,3′-((1R,2R) 1,2-Dihydroxy-1,2-di-*p*-tolylethane-1,2-diyl)­bis­(bicyclo­[1.1.1]­pentane-1-carboxylate) and Dimethyl 3,3′-((1S,2S) 1,2-Dihydroxy-1,2-di-*p*-tolylethane-1,2-diyl)­bis­(bicyclo­[1.1.1]­pentane-1-carboxylate) (**14b**)

Synthesis modified from literature procedures.^63^ Activated zinc powder (496 mg, 7.58 mmol) was added to anhydrous THF (15 mL), purged with argon, and cooled to 0 °C. Titanium tetrachloride (0.27 mL, 3.78 mmol) was added, and the solution was warmed to room temperature. The solution stirred for 30 min at room temperature then refluxed for 2.5 h. The mixture was cooled to 0 °C then **6b** (150 mg, 0.626 mmol) in anhydrous THF (5 mL) was added dropwise. The solution was refluxed for 12 h. The solution was quenched with 10% K_2_CO_3_ (50 mL), extracted with Et_2_O (3 × 20 mL), dried with Na_2_SO_4_, and concentrated *in vacuo*. The product was recrystallized with hexane/DCM yielding an beige solid (39 mg, 0.079 mmol, 25%); Mp = 180–182 °C; ^1^H NMR (400 MHz, CD_2_Cl_2_, 298 K): δ *=* 7.15 (br m, 8H), 3.58 (3H), 2.94 (s, 2H), 2.38 (s, 6H), 1.99–1.96 (dd, *J* = 9.3 Hz, *J* = 1.4 Hz, 6H), 1.70–1.67 (dd, *J* = 9.3 Hz, 1.3 Hz, 6H) ppm; ^13^C­{^1^H} NMR (101 MHz, CD_2_Cl_2_, 298 K): δ *=* 170.2, 137.0, 136.8, 127.5, 78.8, 51.3, 51.1, 45.8, 38.0, 20.7 ppm; HRMS (ESI) *m*/*z*: [M]^−^ calcd for C_30_H_33_O_6_ 489.2283; Found 489.2269.

#### Bicyclo­[1.1.1]­pentane-1,3-diylbis­(*p*-tolylmethanol) (**15**)

Bicyclo­[1.1.1]­pentane-1,3-diylbis­(*p*-tolylmethanone) (91 mg, 0.3 mmol) was dissolved in THF/MeOH 1:1 (v/v) (8 mL) and cooled to 0 °C. To this solution, NaBH_4_ (113 mg, 3.0 mmol) was added in portions, and the mixture was stirred at 0 °C for 30 min. The mixture was diluted with DCM (10 mL), washed with Na_2_SO_4_ (1 × 20 mL), H_2_O (1 × 20 mL), and brine (1 × 20 mL), dried with Na_2_SO_4_, and concentrated to yield a white solid (79 mg, 0.256 mmol, 86%); Mp = 133 °C; ^1^H NMR (400 MHz, CDCl_3_, 298 K): δ *=* 7.11 (m, 8H), 4.65 (s, 2H), 2.34 (s, 6H), 1.98 (m, 6H) ppm; ^13^C­{^1^H} NMR (101 MHz, CDCl_3_, 298 K): δ = 138.6, 136.9, 128.8, 125.8, 73.7, 60.6, 45.7, 43.4, 21.1, 14.2 ppm; HRMS (ESI) *m/z:* [M + Na]^+^ calcd for C_21_H_24_NaO_2_ 331.1669; Found 331.1672

#### *N*,*N*’-(Bicyclo­[1.1.1]­pentane-1,3-diylbis­(*p*-tolylmethylene))­bis­(hexan-1-amine) (**16**)

A solution of **7b** (35 mg, 0.115 mmol) and *n*-hexylamine (2 mL, 15 mmol) in MeCN (2 mL) was subjected to microwave irradiation at 150 °C for 20 min. Volatiles were removed and the residue was redissolved in THF/MeOH 1:1 (4 mL) and cooled to 0 °C. NaBH_4_ (90 mg, 2.3 mmol) was added in portions and the mixture was stirred at 0 °C for 30 min, diluted with DCM (10 mL), washed with NaHCO_3_ (1 × 30 mL) and brine, dried with Na_2_SO_4_, filtered, concentrated and subjected to flash chromatography on silica gel, eluting with 0–100% ethyl acetate/DCM to afford a colorless oil (18 mg, 0.040 mmol, 34%); *R*_*f*_ = 0.42 (DCM/ethyl acetate = 1:1); ^1^H NMR (400 MHz; CDCl_3_, 298 K): δ = 7.1 (m, 8H), 3.60 (s, 2H), 2.40 (t, *J* = 7.4 Hz, 4H), 2.33 (s, 6H), 1.48–1.16 (m, 24H, overlapping), 0.87 (t, *J* = 7.0 Hz, 6H) ppm; ^13^C­{^1^H} NMR (101 MHz; CDCl_3_, 298 K): δ *=* 138.6, 136.1, 128.8, 128.7, 127.3, 127.2, 63.6, 63.5, 48.1, 46.3, 43.1, 31.8, 30.1, 27.0, 22.6, 21.1, 14.1 ppm; HRMS (ESI) *m/z:* [M + H]^+^ calcd for C_33_H_51_N_2_ 475.4047; Found 475.4047.

#### Bicyclo­[1.1.1]­pentane-1,3-diylbis­((4-methoxyphenyl)­(*p*-tolyl)­methanol) (**17**)

Caution! Extreme care should be taken both in the handling of the cryogen liquid nitrogen and its use in the Schlenk line trap to avoid the condensation of oxygen from air. 4-Methoxyphenylmagnesium bromide (2.0 mL, 1.0 mmol, 0.5 M in THF) was added dropwise to a stirred degassed solution of **7b** (91 mg, 0.3 mmol) in dry THF (3 mL) at 0 °C. The mixture was stirred at RT under argon overnight, diluted with DCM (10 mL), washed with NH_4_Cl (30 mL), dried with Na_2_SO_4_, filtered and concentrated. The residue was subjected to column chromatography on silica gel, eluting in gradient 0–10% ethyl acetate/DCM. The product was collected as colorless oil, which solidified upon standing (82 mg, 0.157 mmol, 53%); *R*_*f*_ = 0.51 (DCM: ethyl acetate = 9.5:0.5); ^1^H NMR (400 MHz, CDCl_3_, 298 K): δ *=* 7.38 (d, *J* = 8.9 Hz, 2H), 7.34 (d, *J* = 8.2 Hz, 2H), 7.13 (d, *J* = 8.2 Hz, 2H), 6.84 (d, *J* = 8.9 Hz, 2H), 3.81 (s, 6H), 2.36 (s, 6H), 2.18 (bs, 2H), 1.89 (s, 6H) ppm; ^13^C­{^1^H} NMR (101 MHz, CDCl_3_, 298 K): δ *=* 158.3, 142.0, 137.2, 136.4, 128.5, 125.1, 126.7, 113.1, 76.5, 55.2, 48.0, 47.3, 21.0 ppm; HRMS (ESI) *m/z:* [M]^−^ calcd for C_35_H_35_O_4_ 519.2541; Found 519.2530.

#### 1,1’-(Bicyclo­[1.1.1]­pentane-1,3-diyl)­bis­(1-(*p*-tolyl)-3-(trimethylsilyl)­prop-2-yn-1-ol) (**18**)

Caution! Extreme care should be taken both in the handling of the cryogen liquid nitrogen and its use in the Schlenk line trap to avoid the condensation of oxygen from air. *n*BuLi (700 μL, 1.76 mmol) was added dropwise to a stirred degassed solution of TMS-acetylene (250 μL, 1.76 mmol) in dry THF (3 mL) at −78 °C and stirred at this temperature for 1 h. Afterward, a solution of **7b** (91 mg, 0.3 mmol) in dry THF (3 mL) was added dropwise. The resulting solution was stirred at RT under argon overnight, diluted with DCM (10 mL), washed with NH_4_Cl (1 × 30 mL), dried with Na_2_SO_4_, filtered and concentrated. The residue was subjected to column chromatography on silica gel, eluting in gradient 0–10% ethyl acetate/DCM. The product was collected as colorless oil. (82 mg, 0.164 mmol, 52%); *R*_*f*_ = 0.43 (DCM); ^1^H NMR (400 MHz, CDCl_3_ 298 K): δ *=* 7.38 (d, *J* = 8.1 Hz, 4H), 7.12 (d, *J* = 8.1 Hz, 4H), 2.34 (s, 6H), 2.26 (bs, 2H), 1.48 (s, 6H), 0.23 (s, 18H) ppm; ^13^C­{^1^H} NMR (101 MHz, CDCl_3_, 298 K): δ *=* 138.0, 137.2, 128.5, 125.6, 105.8, 90.9, 72.6, 45.1, 44.9, 21.1, 0.0 ppm; HRMS (ESI) *m/z:* [M + Na]^+^ calcd for C_31_H_40_NaO_2_Si_2_ 523.2459; Found 523.2454.

#### Methyl 3-(4-((1-Isopropoxy-2-methyl-1-oxopropan-2-yl)­oxy)­benzoyl)­bicyclo[1.1.1]­pentane-1-carboxylate (**20a**)

Compound **6m** (24 mg, 0.1 mmol) and 1-methylethyl 2-bromo-2-methylpropanoate (20 μL, 0.1 mmol) were dissolved in 2 mL of DMF and K_2_CO_3_ (28 mg, 0.2 mmol) was added. The suspension was heated to 80 °C for 40 h, cooled to RT, diluted with DCM, washed with H_2_O and brine, dried with MgSO_4_, filtered, concentrated and subjected to flash chromatography on silica eluting with 0–100% DCM/*n*-hexane to yield a colorless oil (18 mg, 0.048 mmol, 48%); *R*_*f*_ = 0. 74 (5% ethyl acetate/DCM); ^1^H NMR (400 MHz, CDCl_3_, 298 K): δ = 7.90 (d, *J* = 8.9 Hz, 2H), 6.83 (d, *J* = 8.9 Hz, 2H), 5.10 (m, 1H), 3.70 (s, 3H), 2.52 (s, 6H), 1.66 (s, 6H), 1.19 (d, *J* = 6.3 Hz, 6H) ppm. ^13^C­{^1^H} NMR (101 MHz; CDCl_3_, 298 K): δ = 194.9, 173.1, 170.0, 160.0, 130.7, 129.5, 117.3, 79.4, 69.4, 54.5, 51.8, 43.7, 38.2, 25.3, 21.5 ppm; HRMS (ESI) *m*/*z*: [M + Na]^+^ calcd for C_21_H_26_NaO_6_ 397.1622, Found 397.1624.

### Crystallography

Crystals of **6a**, **6b**, **6c**, **6k**, **6l**, **6n**, **6m**, **6o**, **7a**, **7b**, **7l**, **14a**, and **14b** were mounted on a MiTeGen micromount with NVH immersion oil. Data for were collected from a shock-cooled single crystal at 100(2) K on a Bruker Apex Kappa Duo Imus CuKa Kappa diffractometer with a microfocus sealed X-ray tube using mirror optics as a monochromator and an APEX2 detector. The diffractometer was equipped with an Oxford Cobra low temperature device and used Cu Kα radiation (λ = 1.54178 Å). All data were integrated with SAINT and a multiscan absorption correction using SADABS was applied.^64,65^ The structures were solved with the XT structure solution program,^66^ using the intrinsic phasing solution method and refined against |F^2^| with XL using least-squares minimization within OLEX2.^67,68^ Hydrogen atoms, unless specified, were placed in geometrically calculated positions and refined using a riding model. Molecular graphics were generated using OLEX2. Details on data collection and refinement are given in Tables S1–S4.

## Supplementary Material



## Data Availability

The data underlying this study are available in the published article and its Supporting Information.

## Notes

This file contains ^1^H, ^13^C NMR, HRMS spectra, and X-ray data (PDF)

## Notes

This work was posted as a preprint on Chemrxiv (https://doi.org/10.26434/chemrxiv-2025-74k3l).
