# Supplementary material for: Bicyclo[1.1.1]pentane Ketones via Friedel–Crafts Acylation
Source: J Org Chem. 2025 Dec 22;91(1):87–103. doi: 10.1021/acs.joc.5c01754 (PMC12797227; doi:10.1021/acs.joc.5c01754)
Supplement: Supplementary file 1 [file jo5c01754_si_001.pdf]

Supporting Information

For

Bicyclo[1.1.1]pentane Ketones via Friedel-Crafts Acylation

Karolina Urbańska, Freya Ritterling, Brendan Twamley, Oliver Cseh, Vitalina Levchenko, Vasyl Ripenko, Pavel K. Mykhailiuk* and Mathias O. Senge*

Table of Contents

^{1}H NMR spectra S3

$^{13}C\{^{1}H\}$ NMR spectra S35

2D NMR spectra S57

Mass spectra S59

Single X-ray crystallography data S84

Crystallography and refinement details S86

Hirshfeld Surface Analysis S101

^{1}H NMR spectra

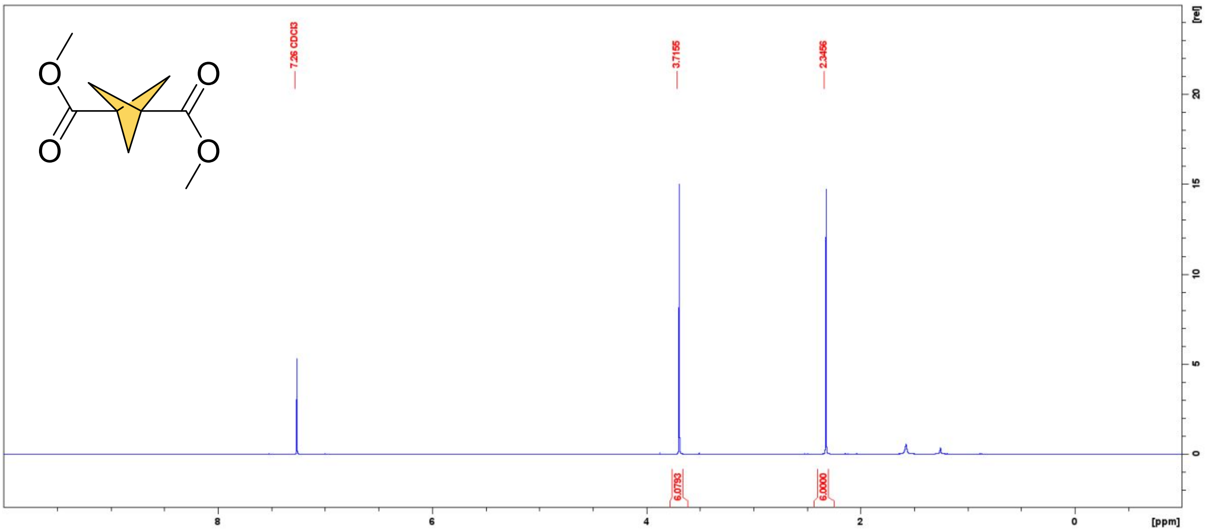

Figure S1: ^{1}H NMR (CDCl$_3$, 400 MHz, 298 K) spectrum of dimethyl bicyclo[1.1.1]pentane-1,3-dicarboxylate.

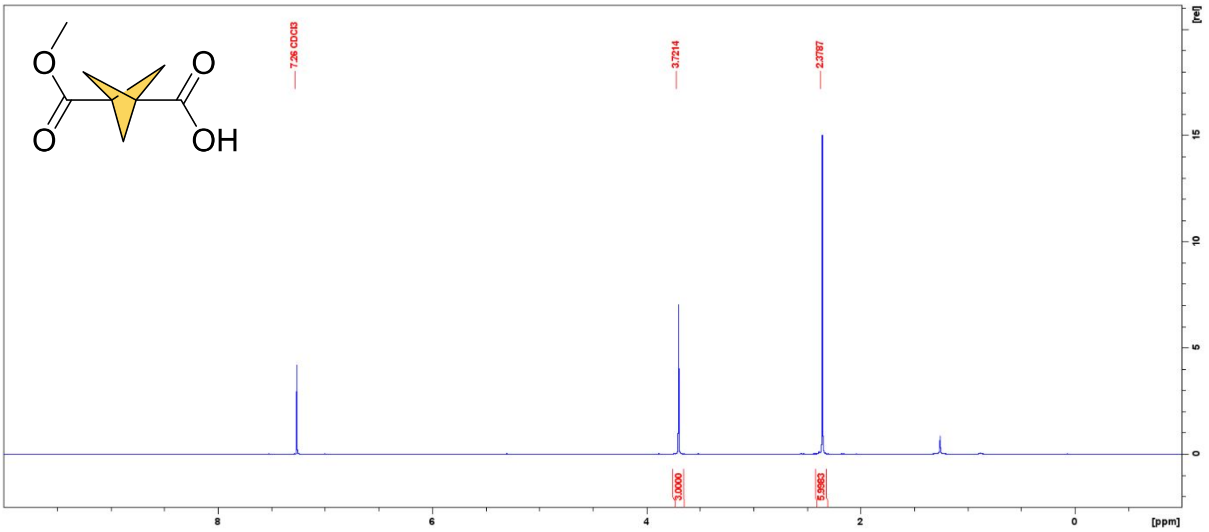

*Figure S2: ^{1}H NMR (CDCl$_3$, 400 MHz, 298 K) spectrum of **2**.*

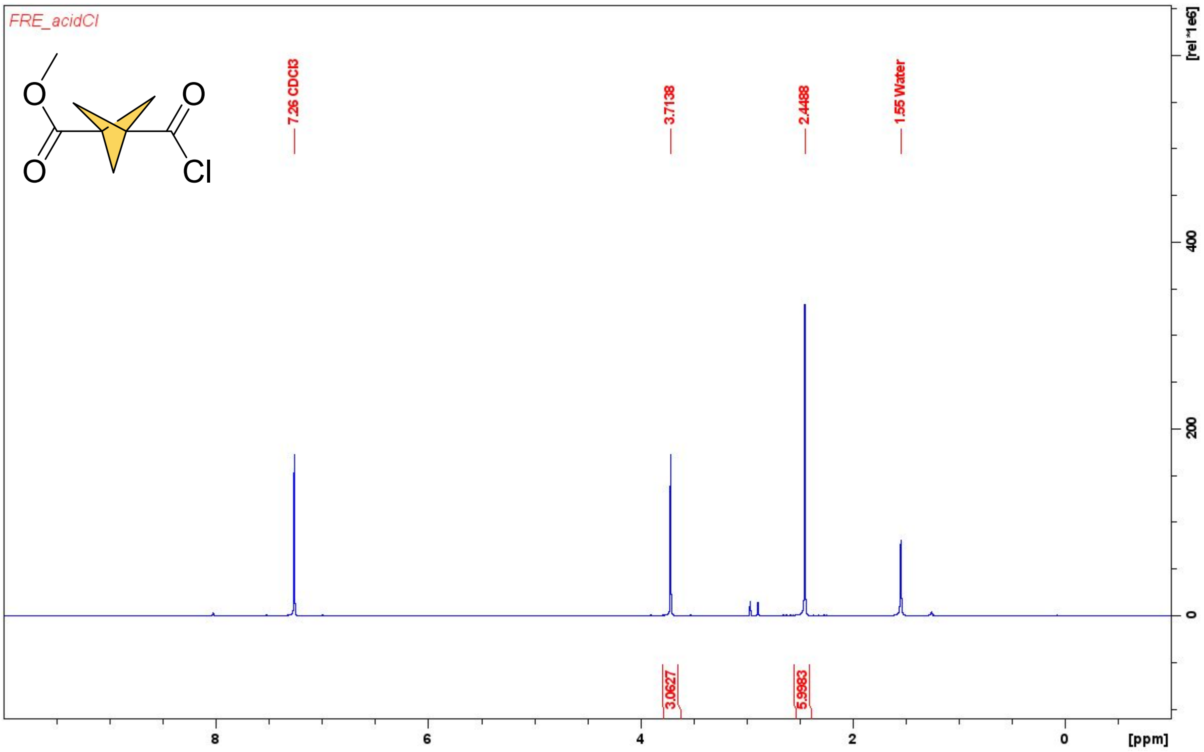

Figure S3: ^{1}H NMR ($CDCl_3$, 400 MHz, 298 K) spectrum of **3**.

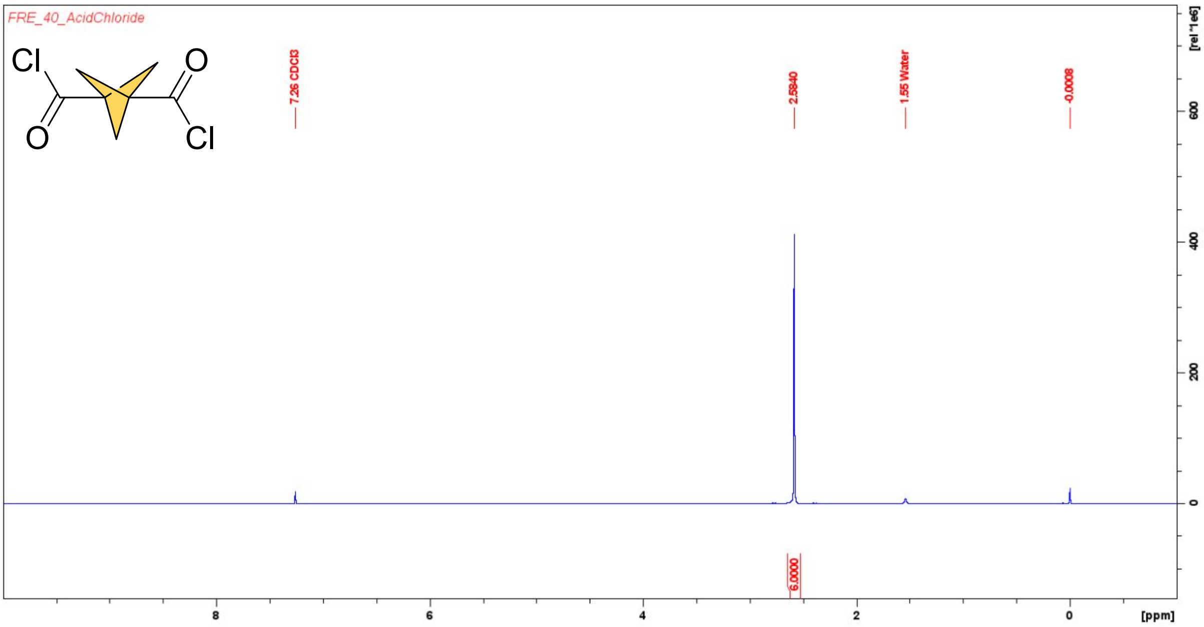

Figure S4: ^{1}H NMR ($CDCl_3$, 400 MHz, 298 K) spectrum of **4**.

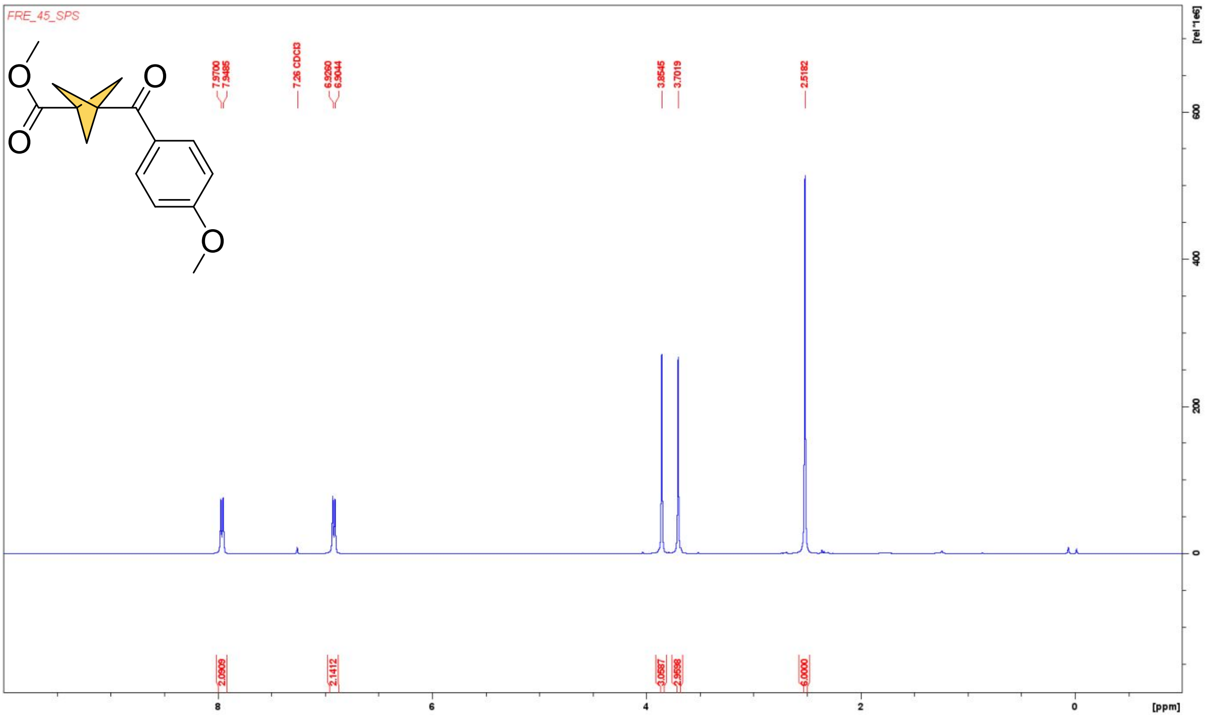

*Figure S5: ^{1}H NMR ($CDCl_3$, 400 MHz, 298 K) spectrum of **6a**.*

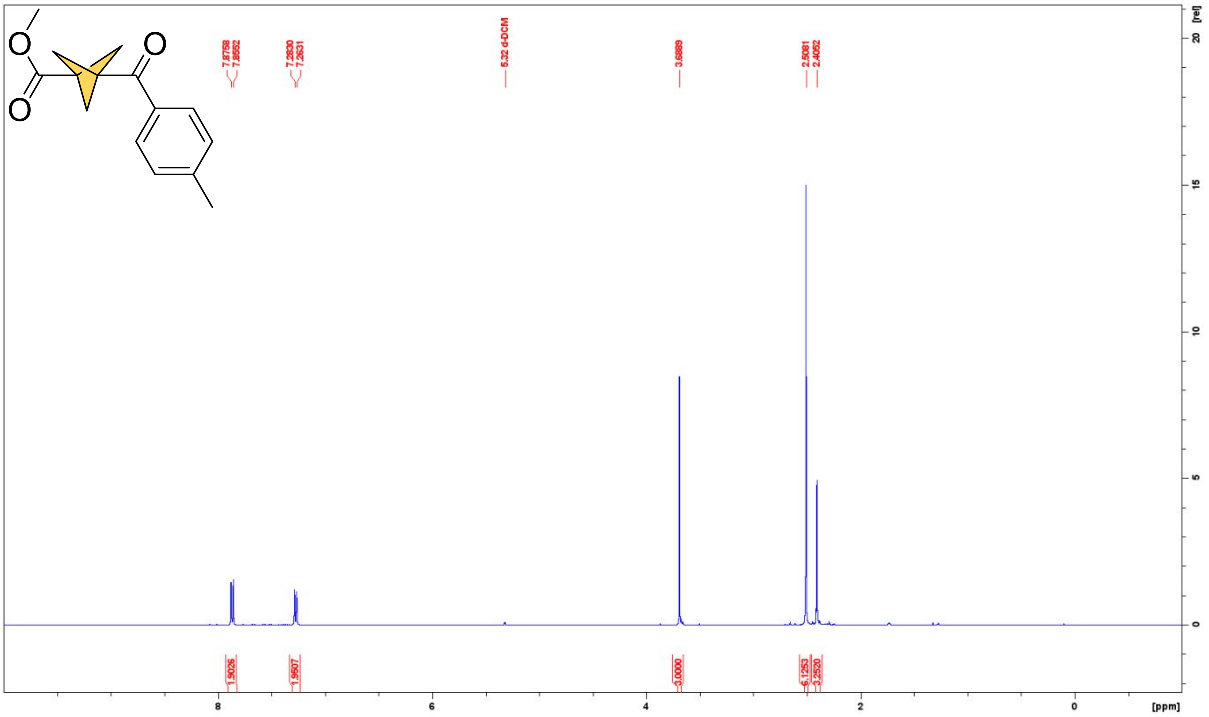

*Figure S6: ^{1}H NMR (d-CD_2Cl_2, 400 MHz, 298 K) spectrum of **6b**.*

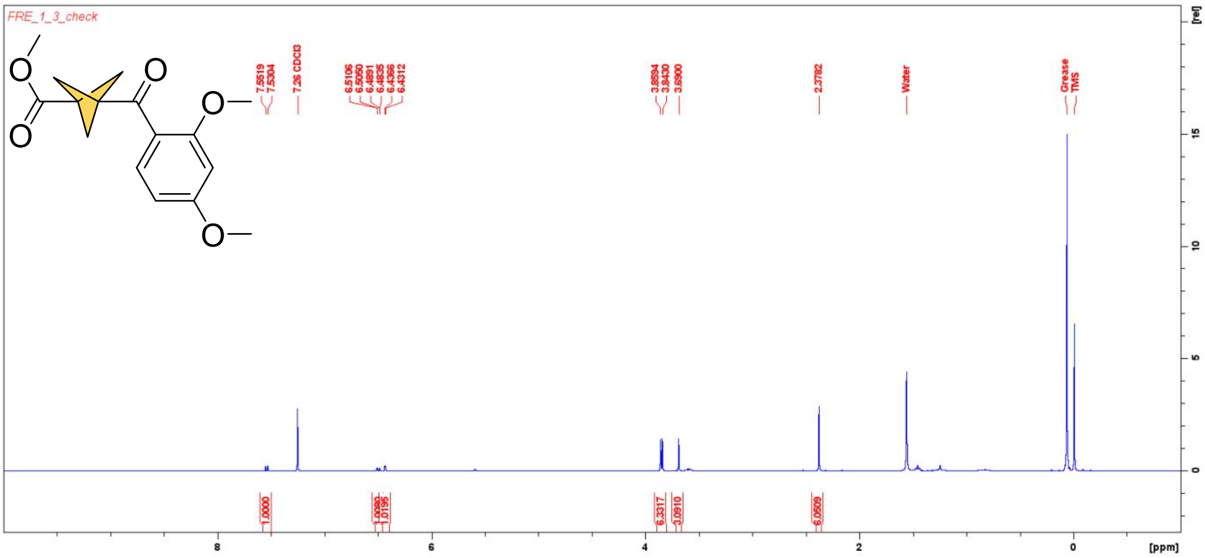

*Figure S7: ^{1}H NMR ($CDCl_3$, 400 MHz, 298 K) spectrum of **6c**.*

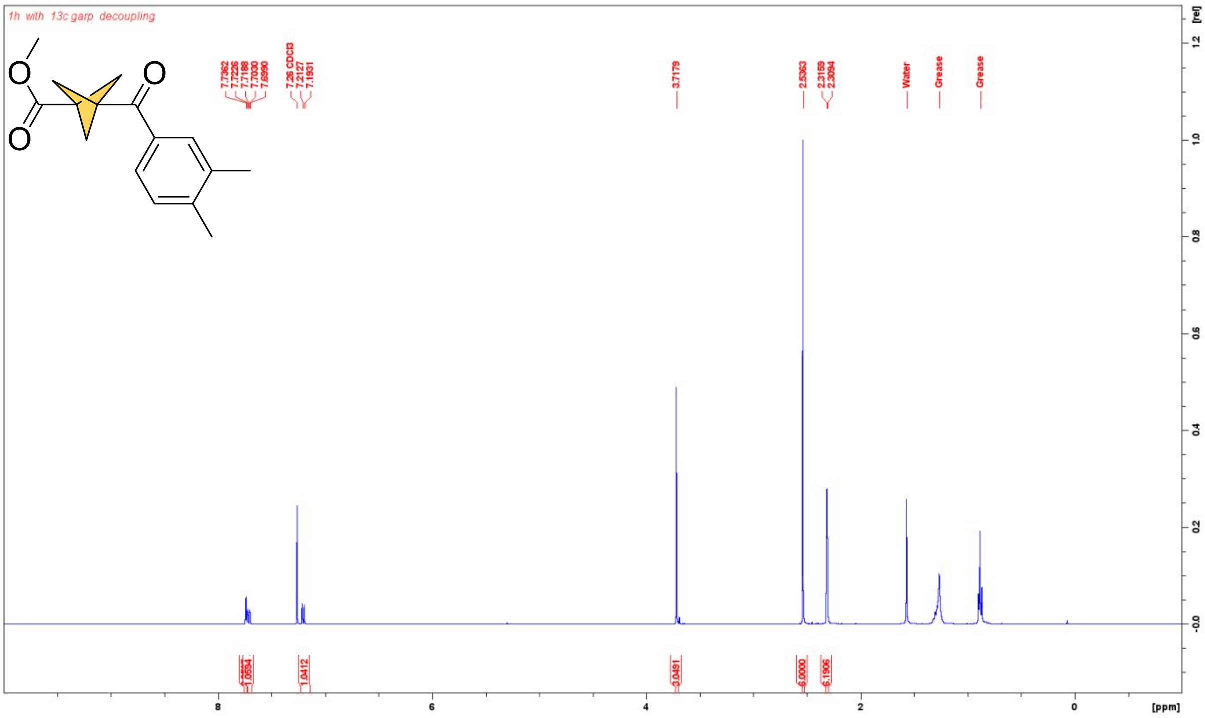

*Figure S8: ^{1}H NMR ($CDCl_3$, 400 MHz, 298 K) spectrum of **6d**.*

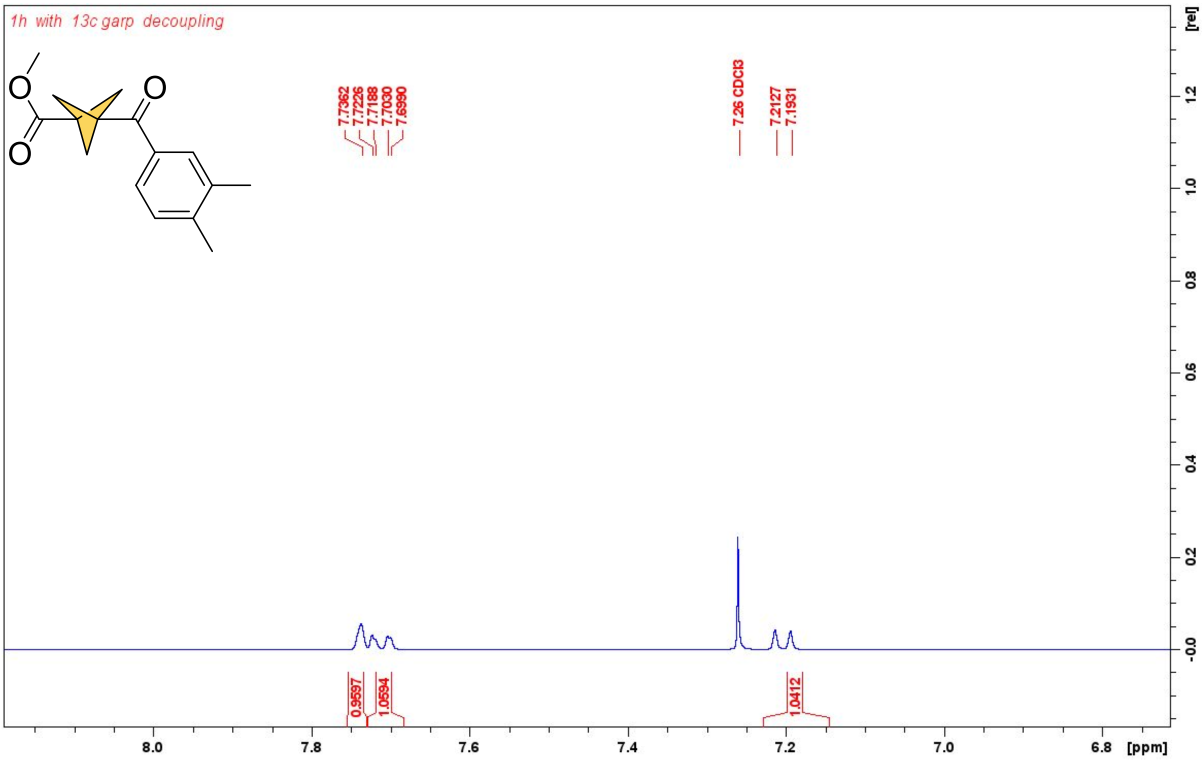

*Figure S9: ^{1}H NMR ($CDCl_3$, 400 MHz, 298 K) spectrum of **6d** zoomed in on aromatic region.*

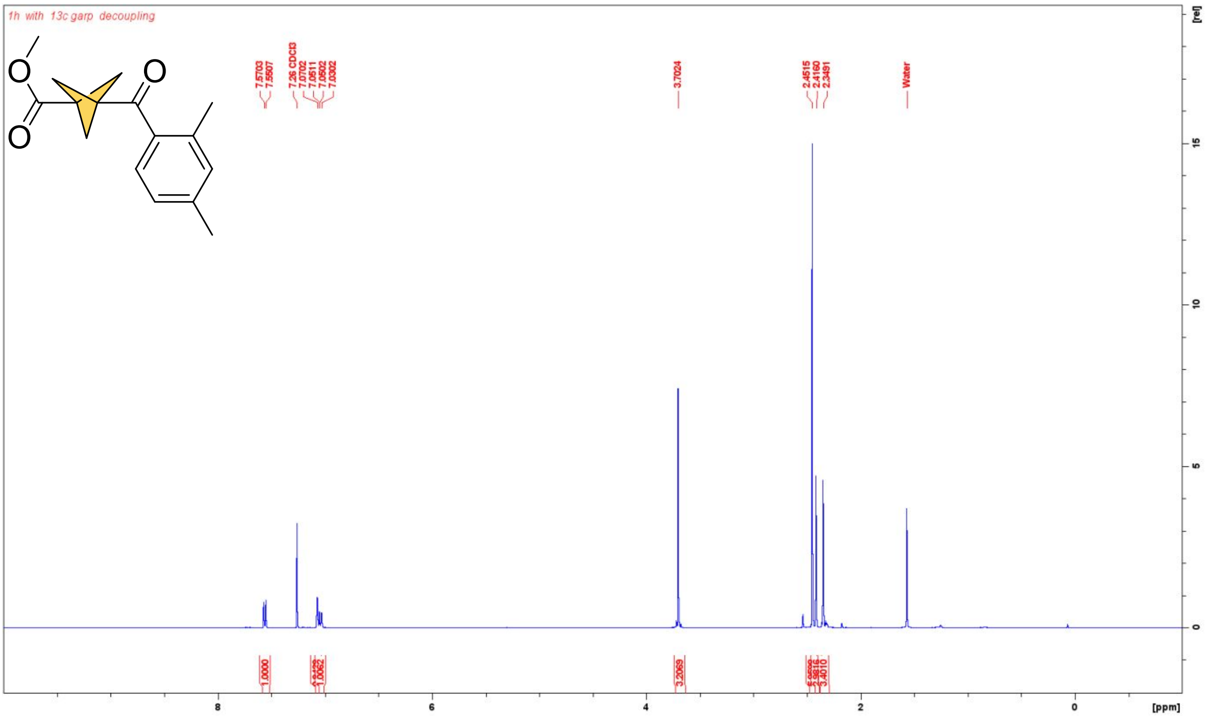

*Figure S10: ^{1}H NMR ($CDCl_3$, 400 MHz, 298 K) spectrum of **6e**.*

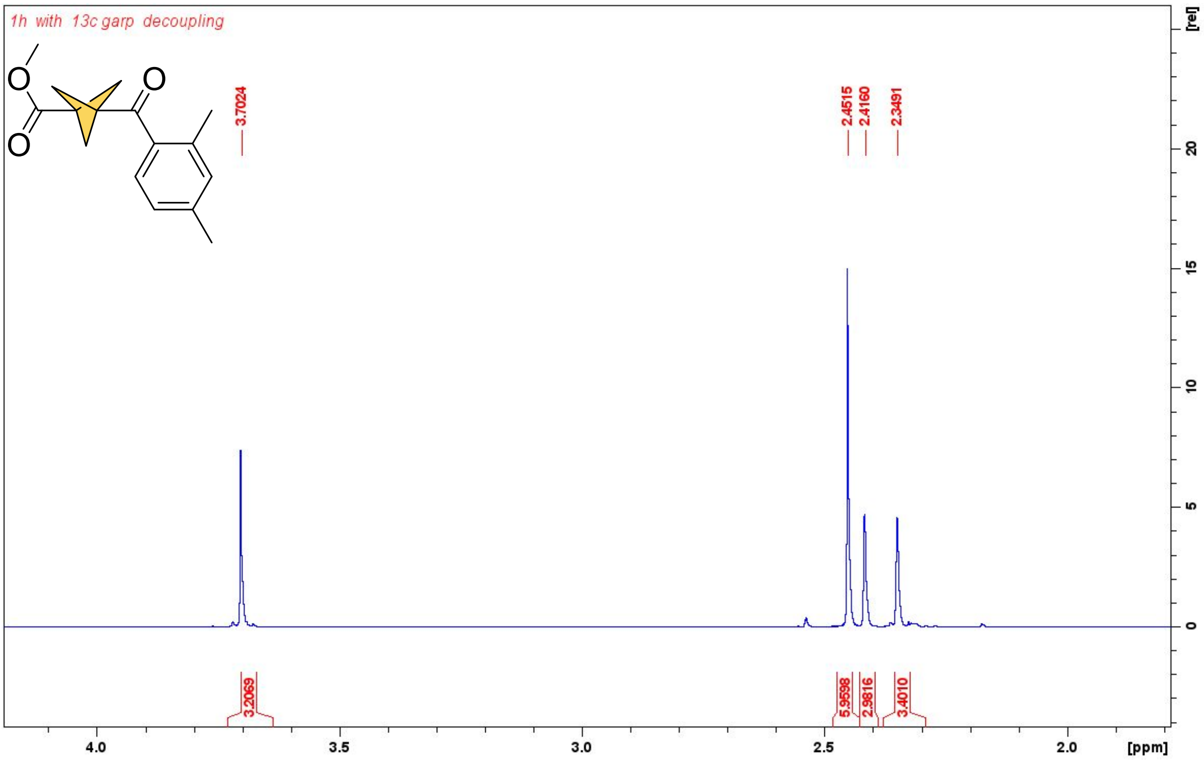

*Figure S11: ^{1}H NMR ($CDCl_3$, 400 MHz, 298 K) spectrum of **6e** zoomed in on aliphatic region.*

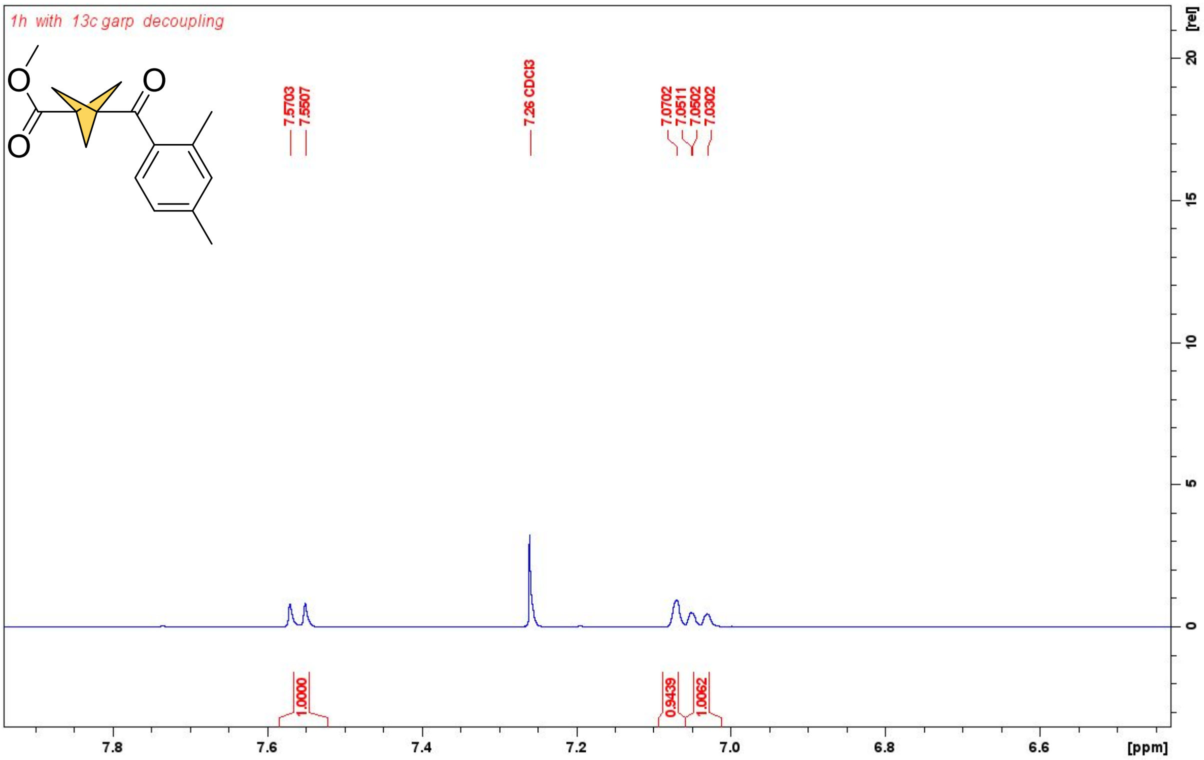

*Figure S12: ^{1}H NMR ($CDCl_3$, 400 MHz, 298 K) spectrum of **6e** zoomed in on aromatic region.*

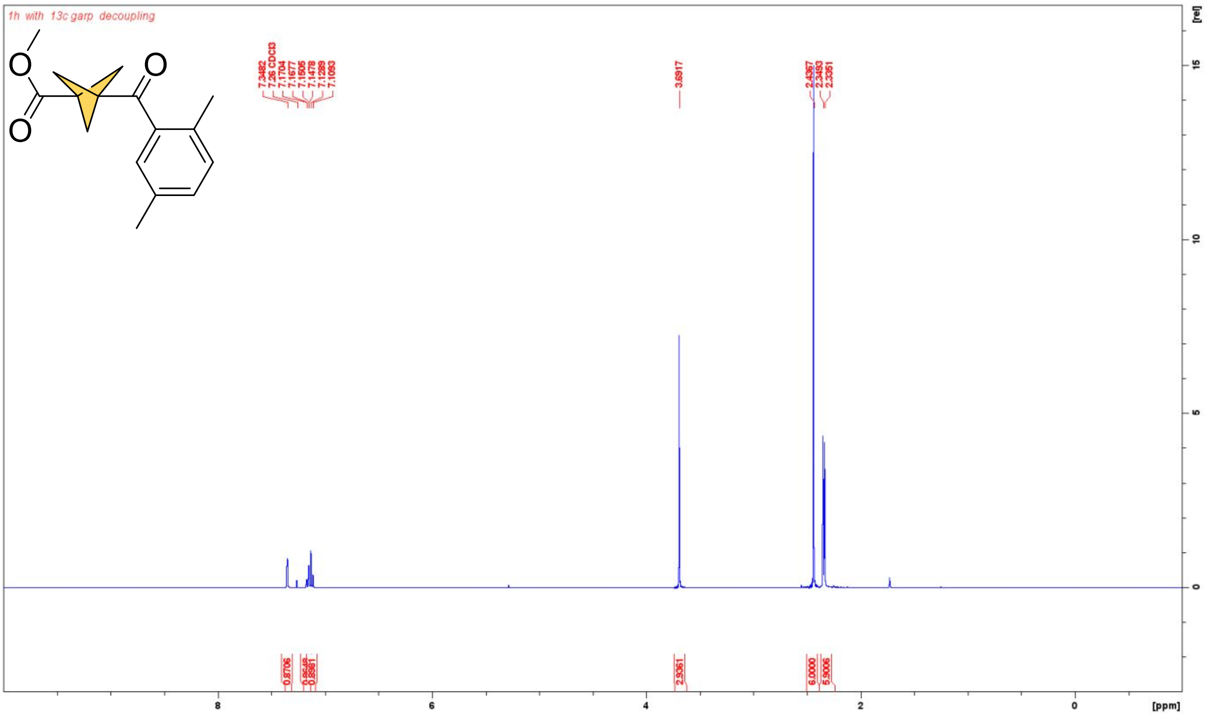

Figure S13: ^{1}H NMR ($CDCl_3$, 400 MHz, 298 K) spectrum of ***6f***.

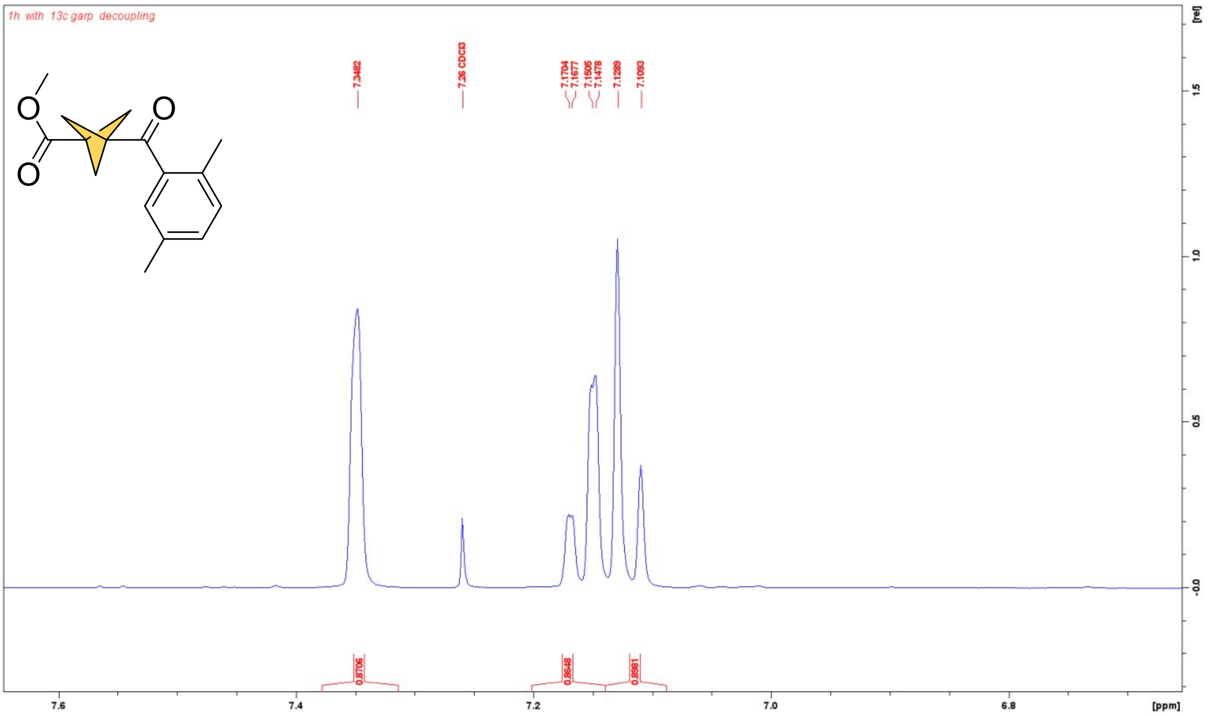

Figure S14: ^{1}H NMR ($CDCl_3$, 400 MHz, 298 K) spectrum of ***6f*** zoomed in on aromatic region.

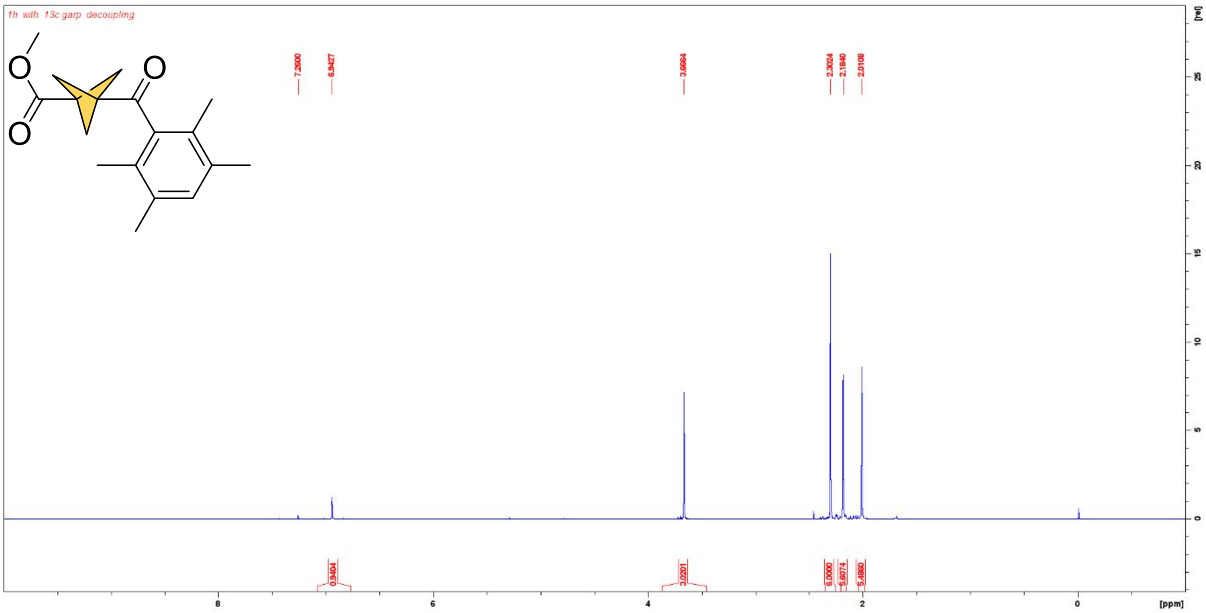

Figure S15: ¹H NMR (CDCl$_3$, 400 MHz, 298 K) spectrum of ***6g****.*

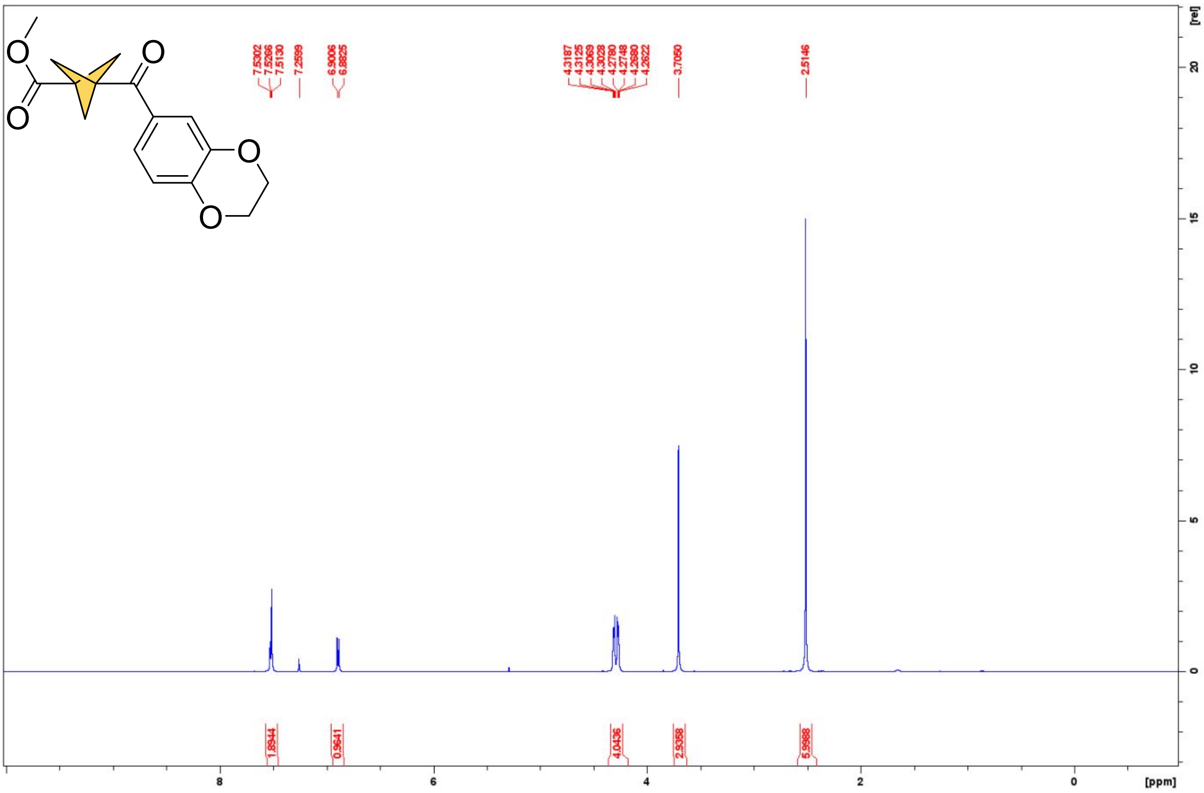

Figure S16: ¹H NMR (CDCl$_3$, 500 MHz, 298 K) spectrum of ***6h****.*

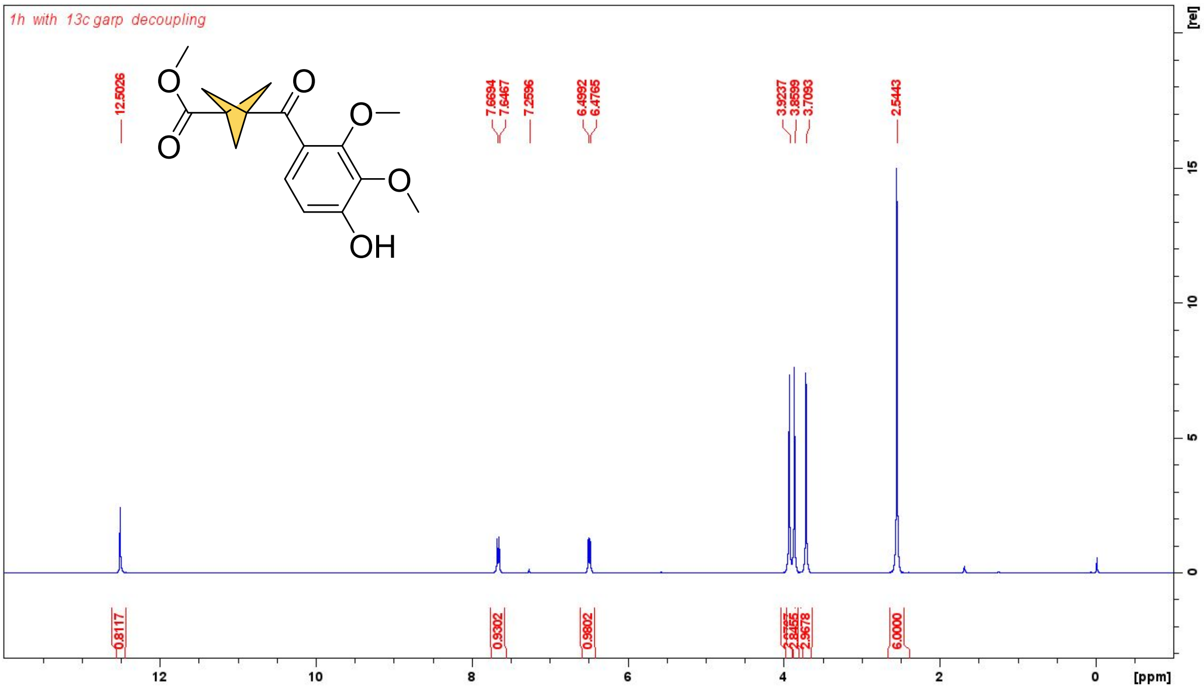

*Figure S17: ^{1}H NMR (CDCl$_3$, 400 MHz, 298 K) spectrum of **6i**.*

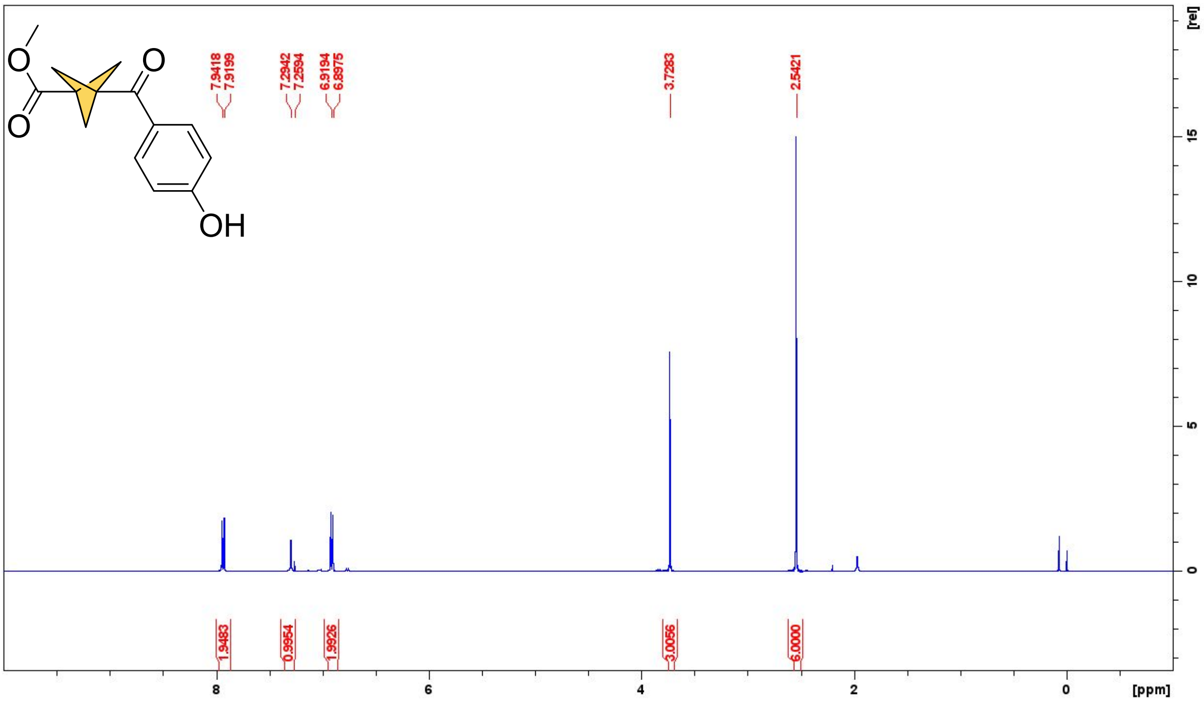

*Figure S18: ^{1}H NMR (CDCl$_3$, 400 MHz, 298 K) spectrum of **6j**.*

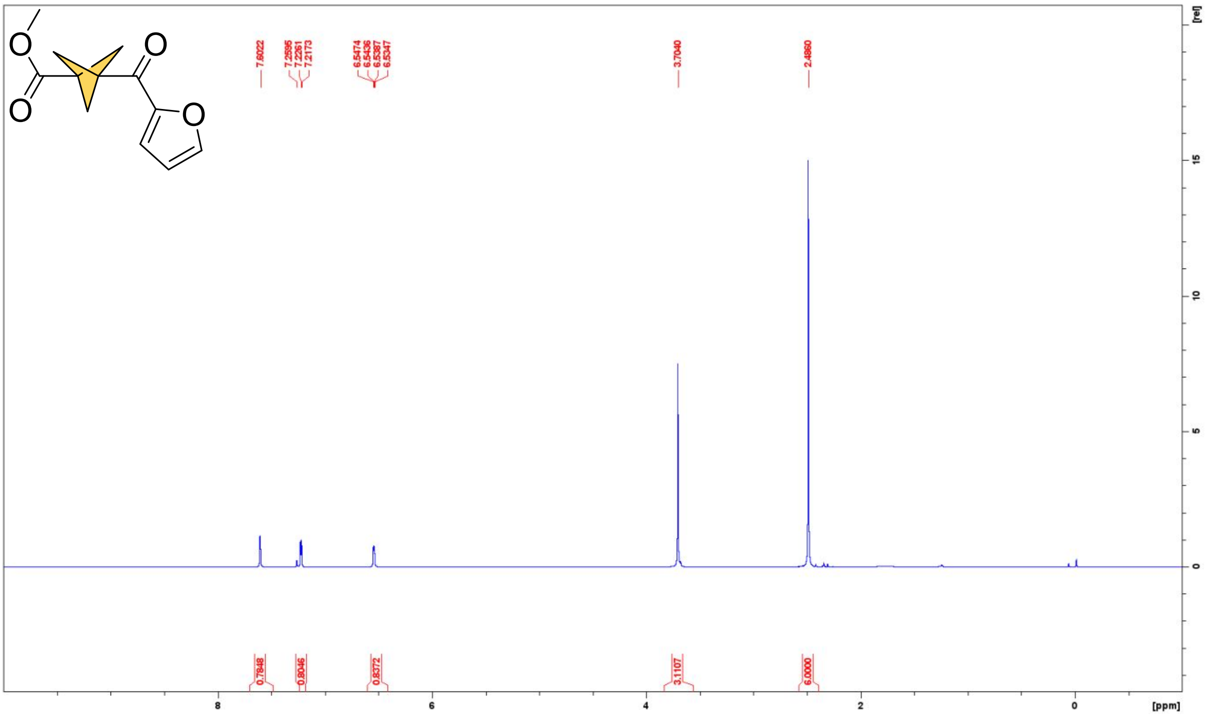

*Figure S19: ^{1}H NMR ($CDCl_3$, 400 MHz, 298 K) spectrum of **6k**.*

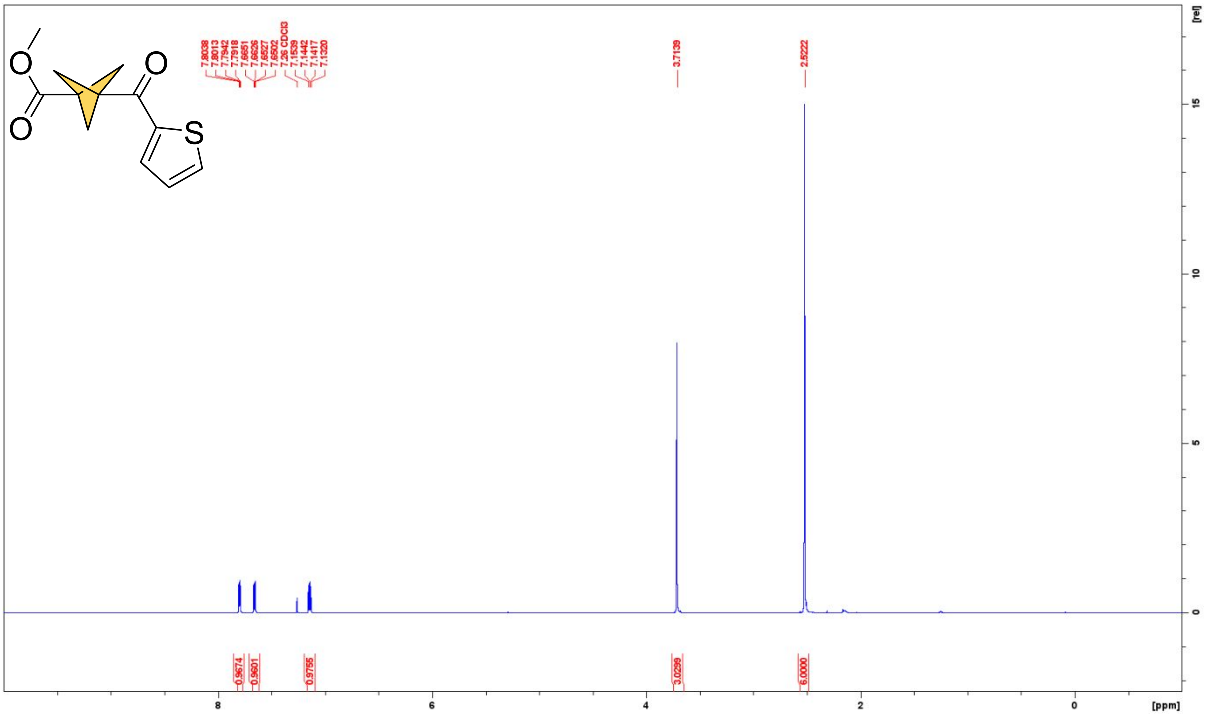

*Figure S20: ^{1}H NMR ($CDCl_3$, 400 MHz, 298 K) spectrum of **6l**.*

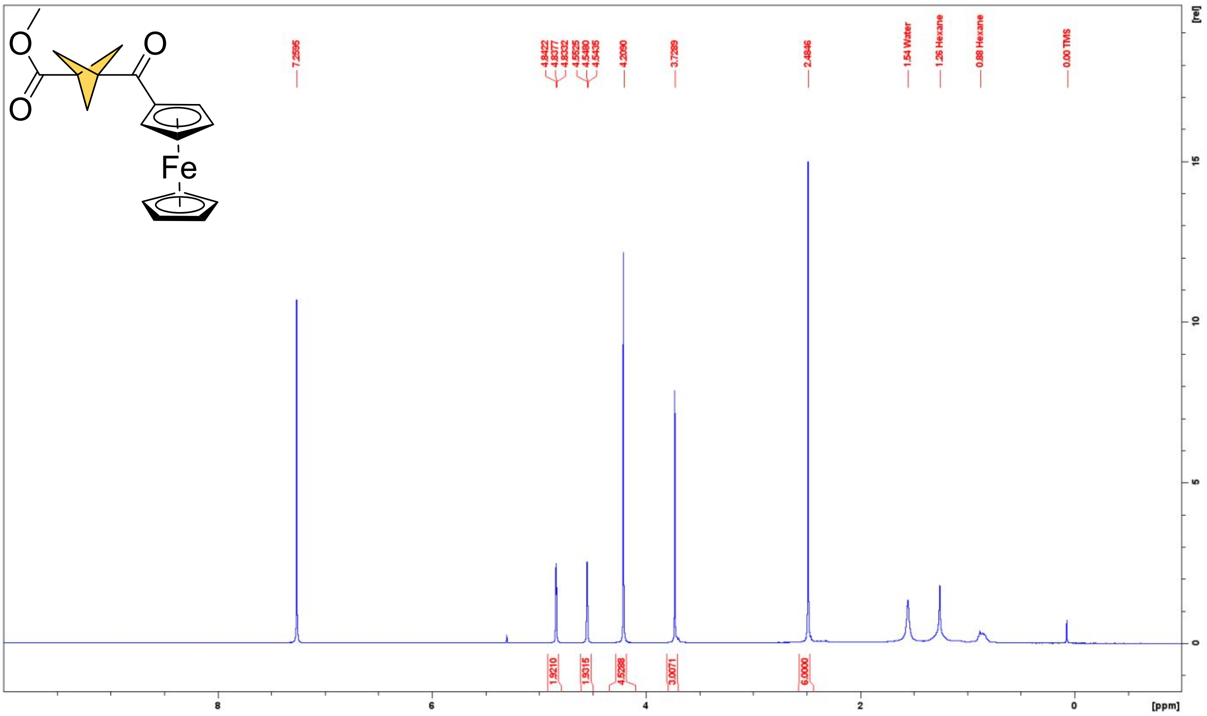

Figure S21: ^{1}H NMR ($CDCl_3$, 400 MHz, 298 K) spectrum of **6m.**

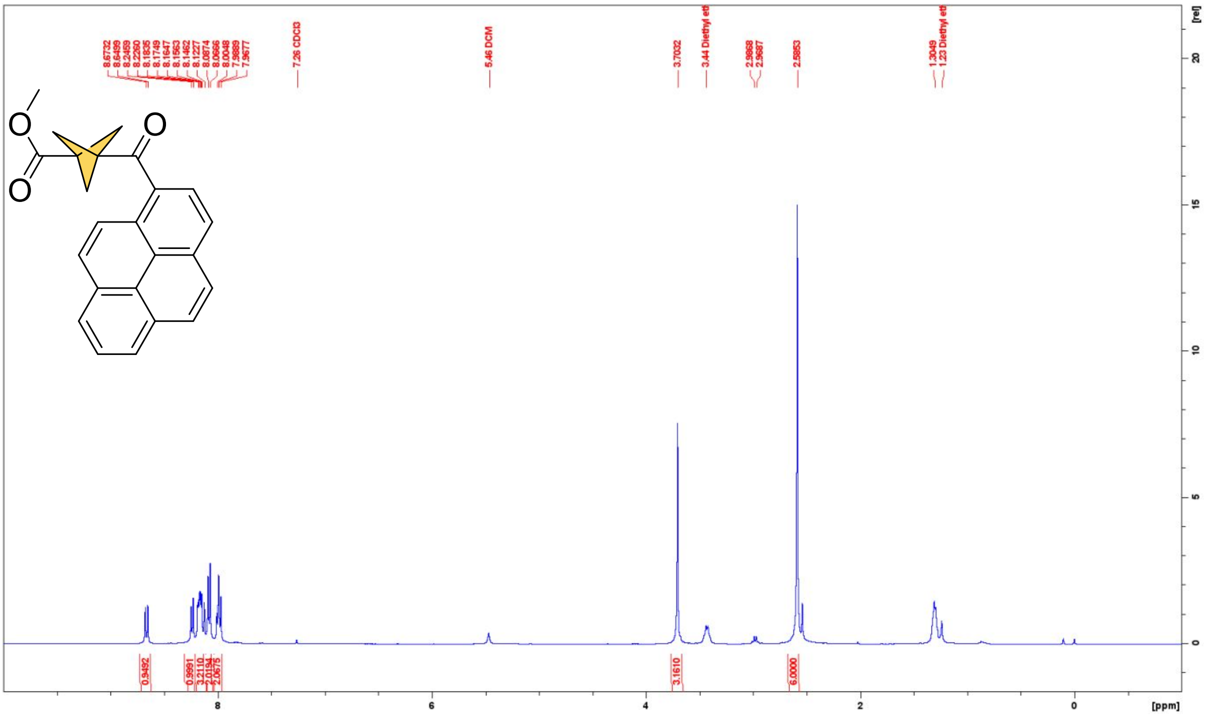

Figure S22: ^{1}H NMR ($CDCl_3$, 400 MHz, 298 K) spectrum of **6n**.

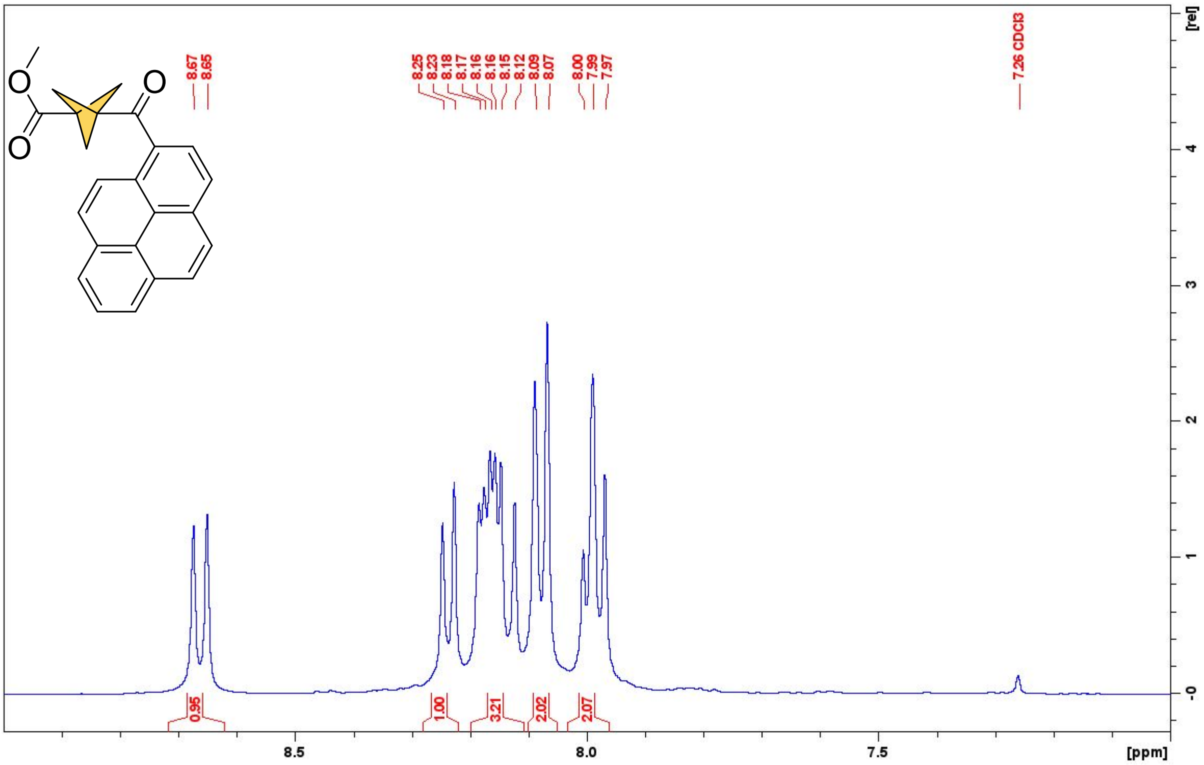

Figure S23: ^{1}H NMR ($CDCl_3$, 400 MHz, 298 K) spectrum of ***6n*** zoomed in on aromatic region.

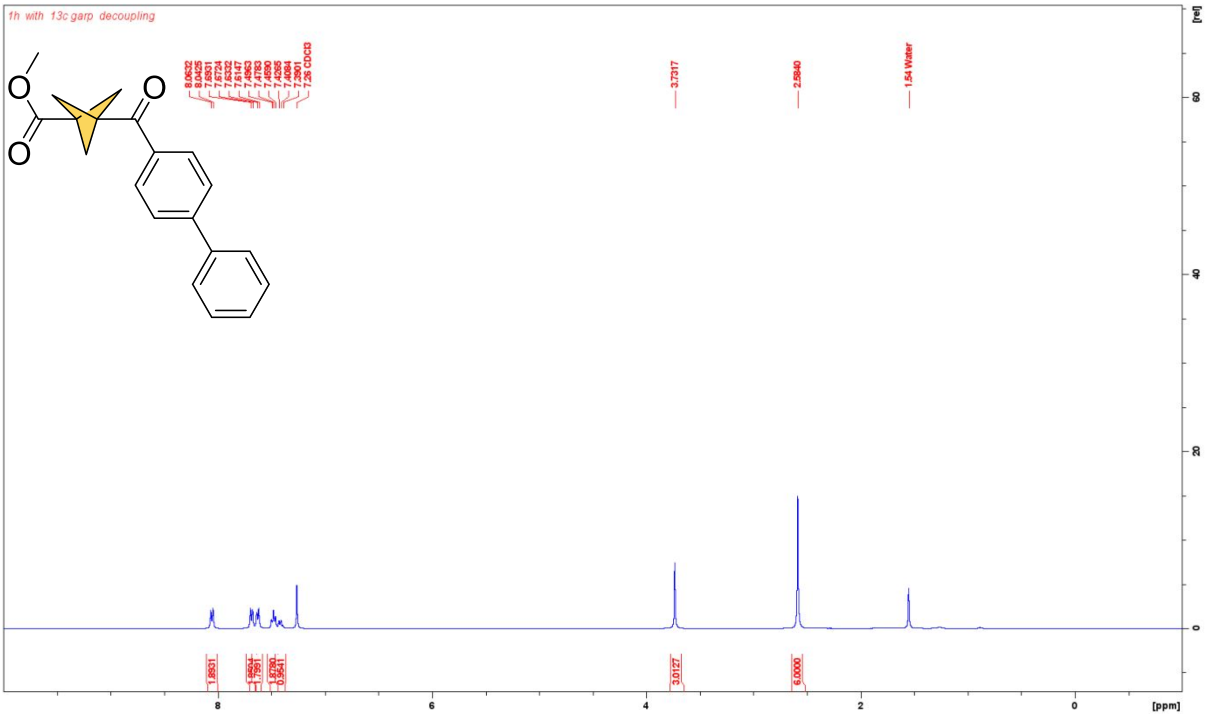

Figure S24: ^{1}H NMR ($CDCl_3$, 400 MHz, 298 K) spectrum of ***6o***.

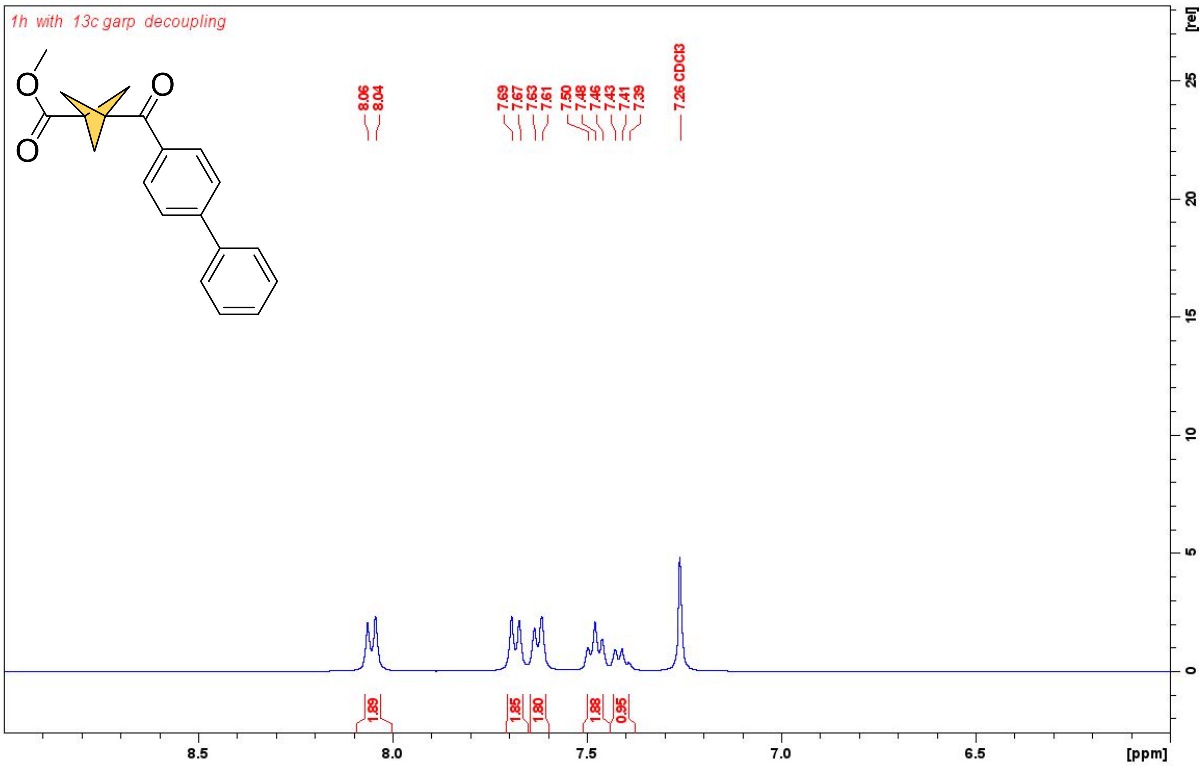

Figure S25: ^{1}H NMR ($CDCl_3$, 400 MHz, 298 K) spectrum of **6o** zoomed in on aromatic region.

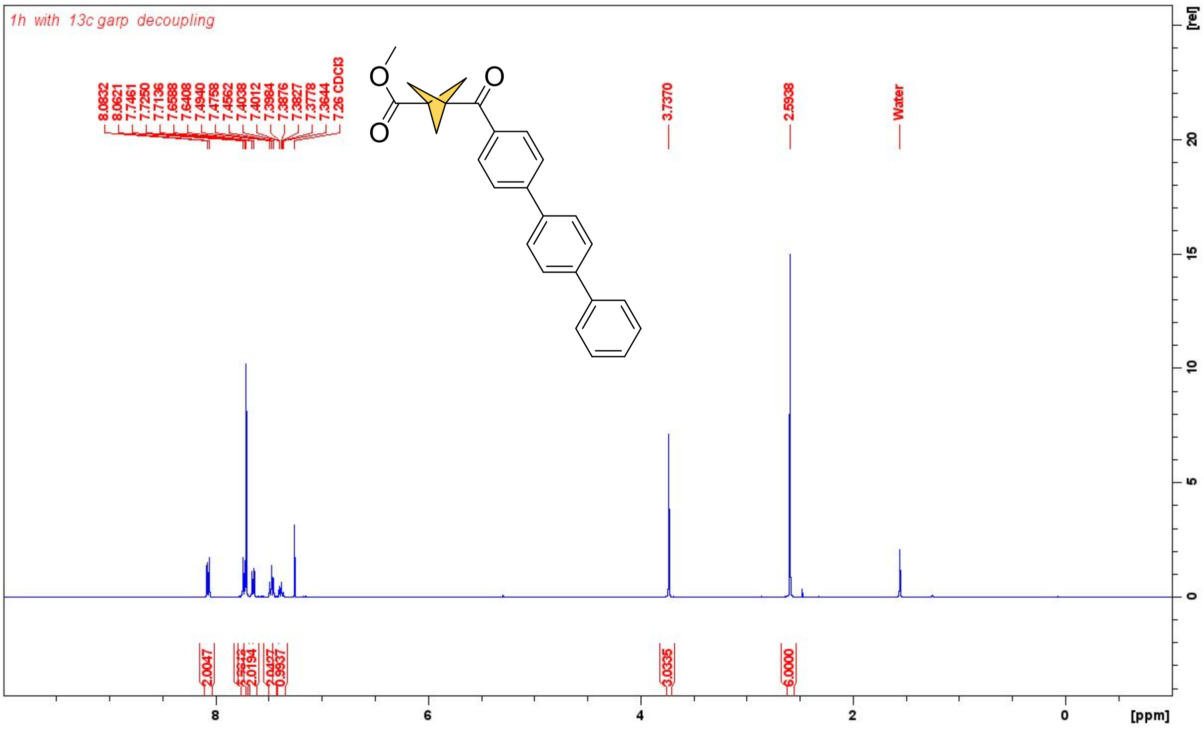

Figure S26: ^{1}H NMR ($CDCl_3$, 400 MHz, 298 K) spectrum of **6p**.

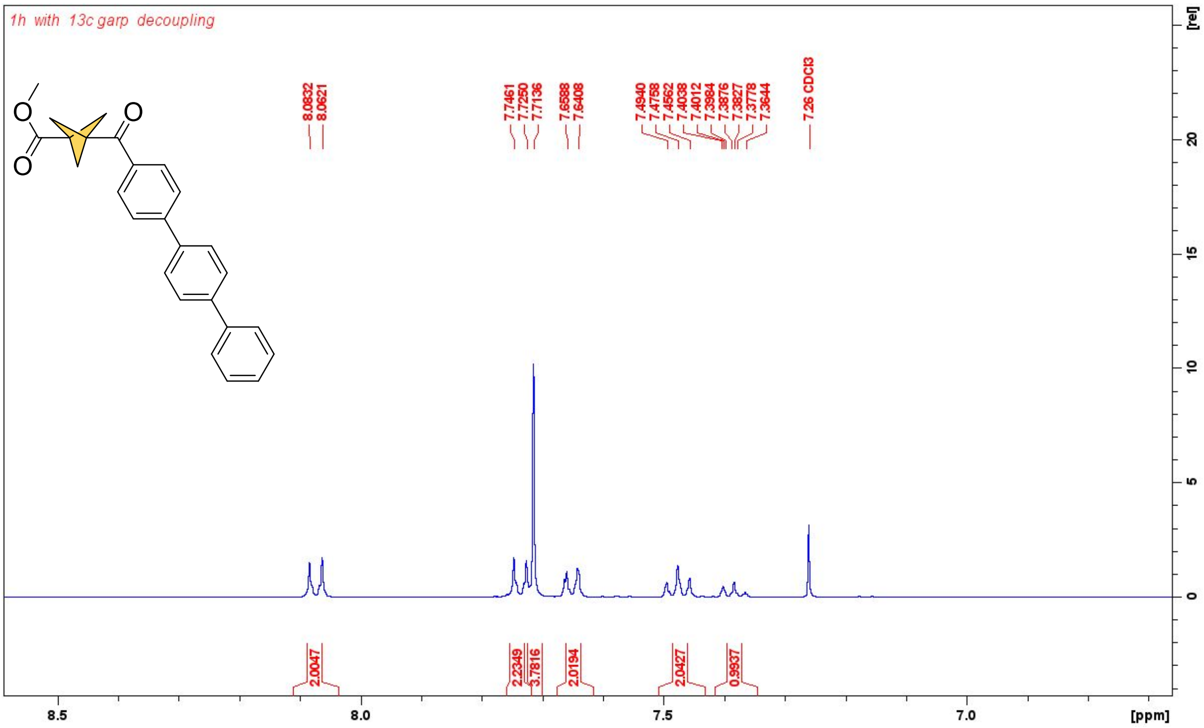

Figure S27: ^{1}H NMR ($CDCl_3$, 400 MHz, 298 K) spectrum of **6p** zoomed in on aromatic region.

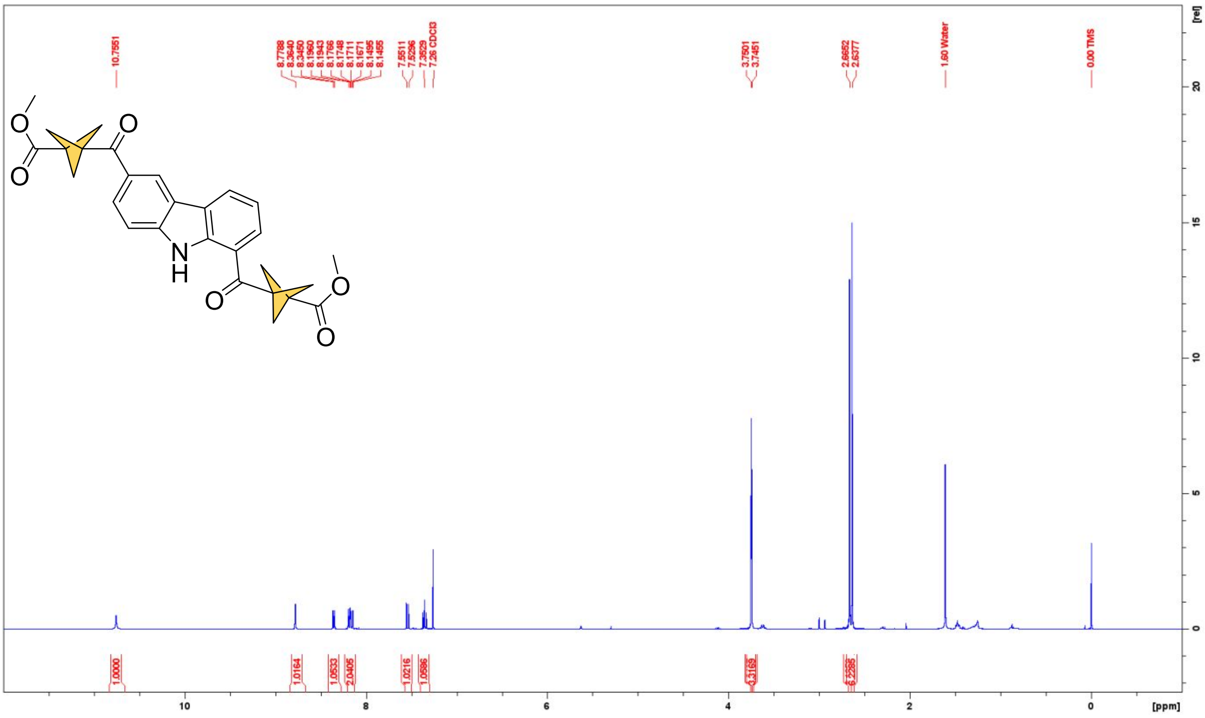

Figure S28: ^{1}H NMR ($CDCl_3$, 400 MHz, 298 K) spectrum of **6q**.

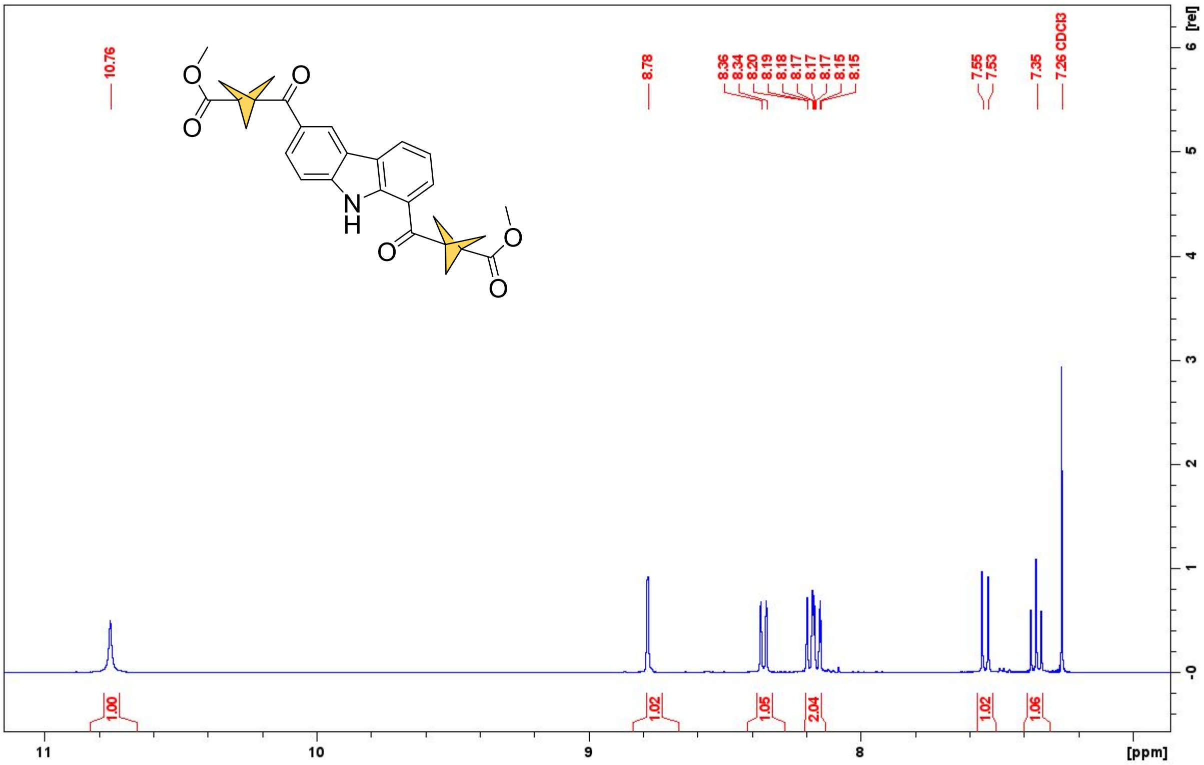

Figure S29: ¹H NMR ($CDCl_3$, 400 MHz, 298 K) spectrum of ***6q*** *zoomed in on aromatic region.*

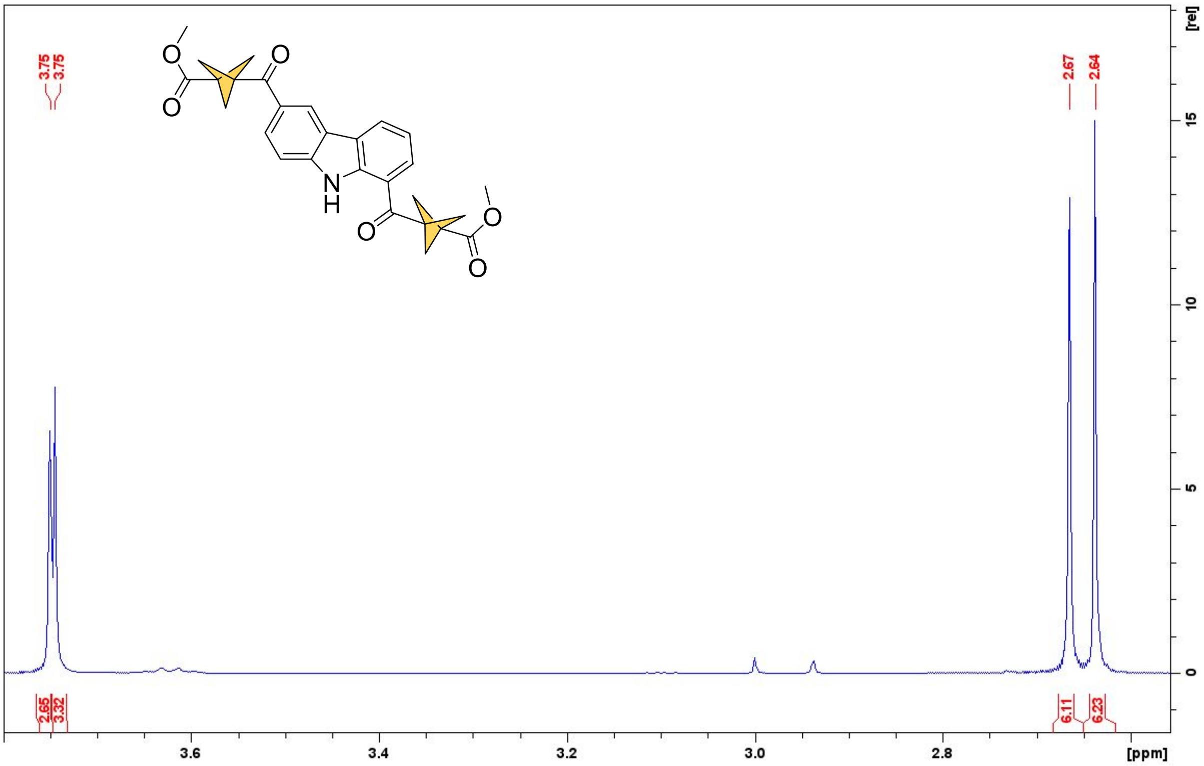

Figure S30: ¹H NMR ($CDCl_3$, 400 MHz, 298 K) spectrum of ***6q*** *zoomed in on aliphatic region.*

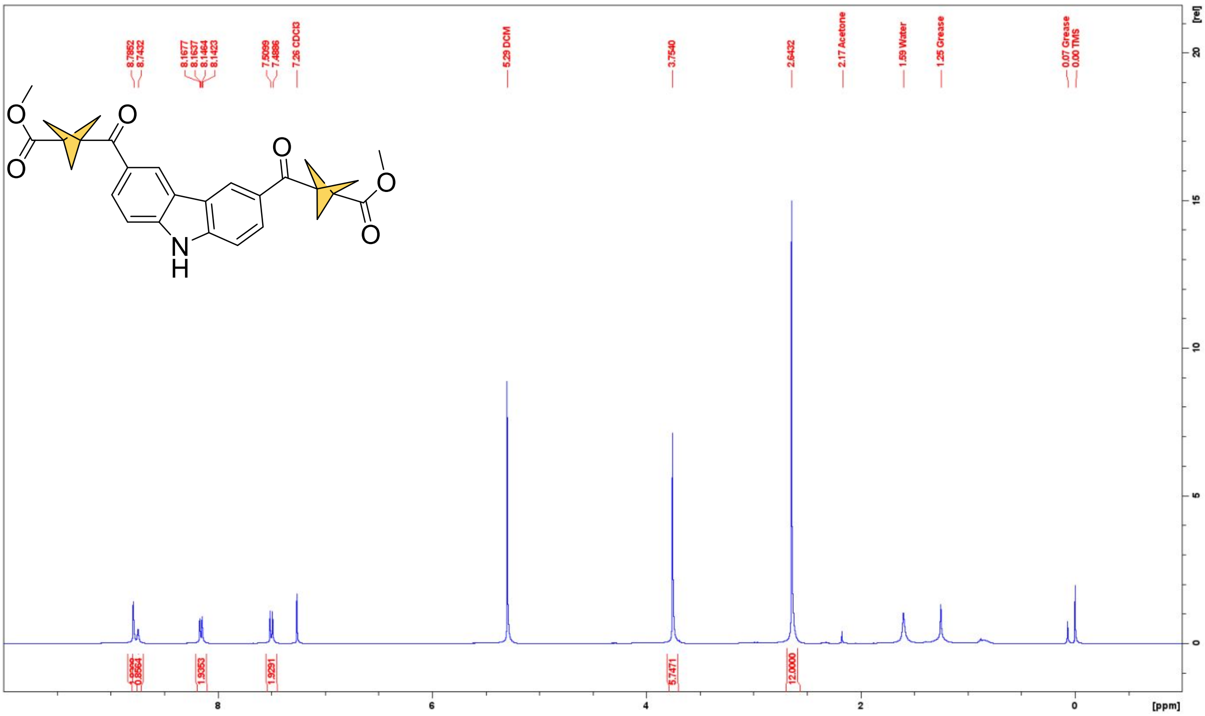

*Figure S31: ¹H NMR (CDCl$_3$, 400 MHz, 298 K) spectrum of **6r**.*

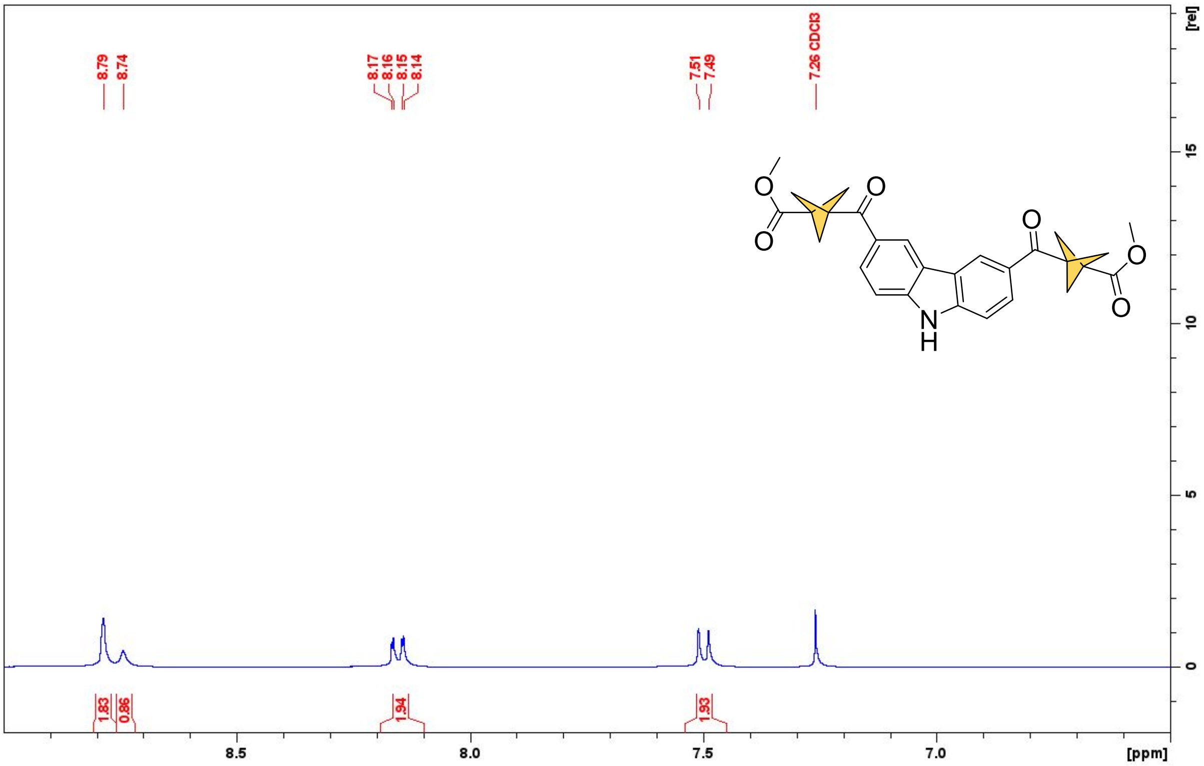

*Figure S32: ¹H NMR (CDCl$_3$, 400 MHz, 298 K) spectrum of **6r** zoomed in on aromatic region.*

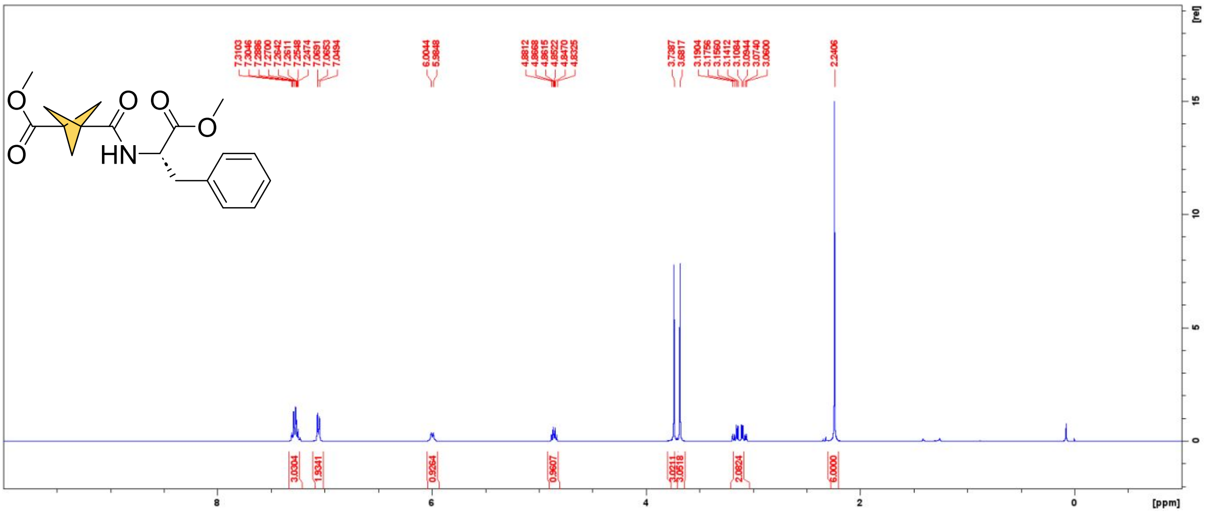

Figure S33: ^{1}H NMR (CDCl$_3$, 400 MHz, 298 K) spectrum of ***6s.***

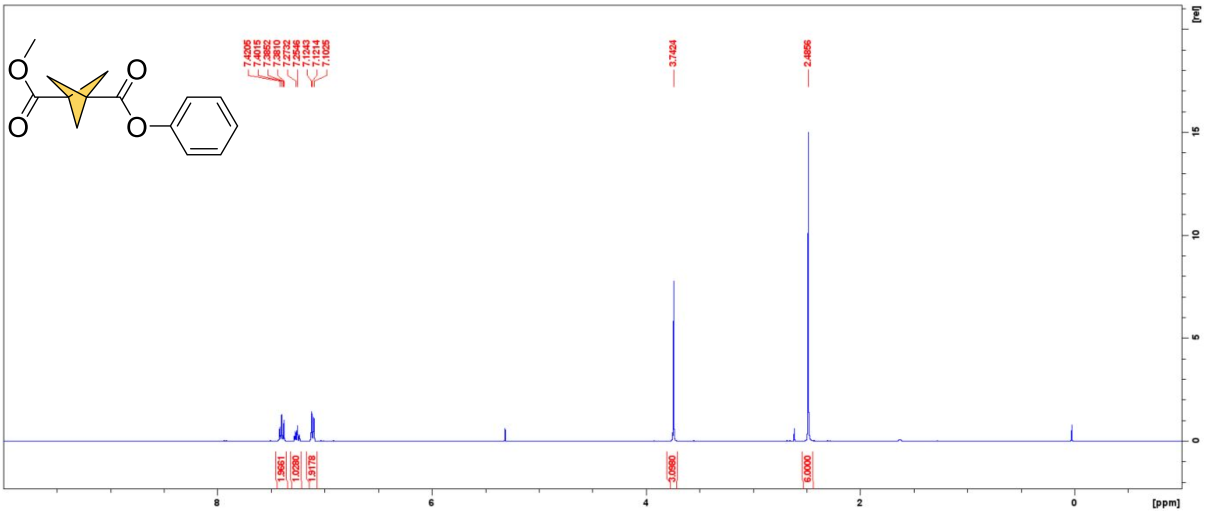

Figure S34: ^{1}H NMR (CDCl$_3$, 400 MHz, 298 K) spectrum of ***6t.***

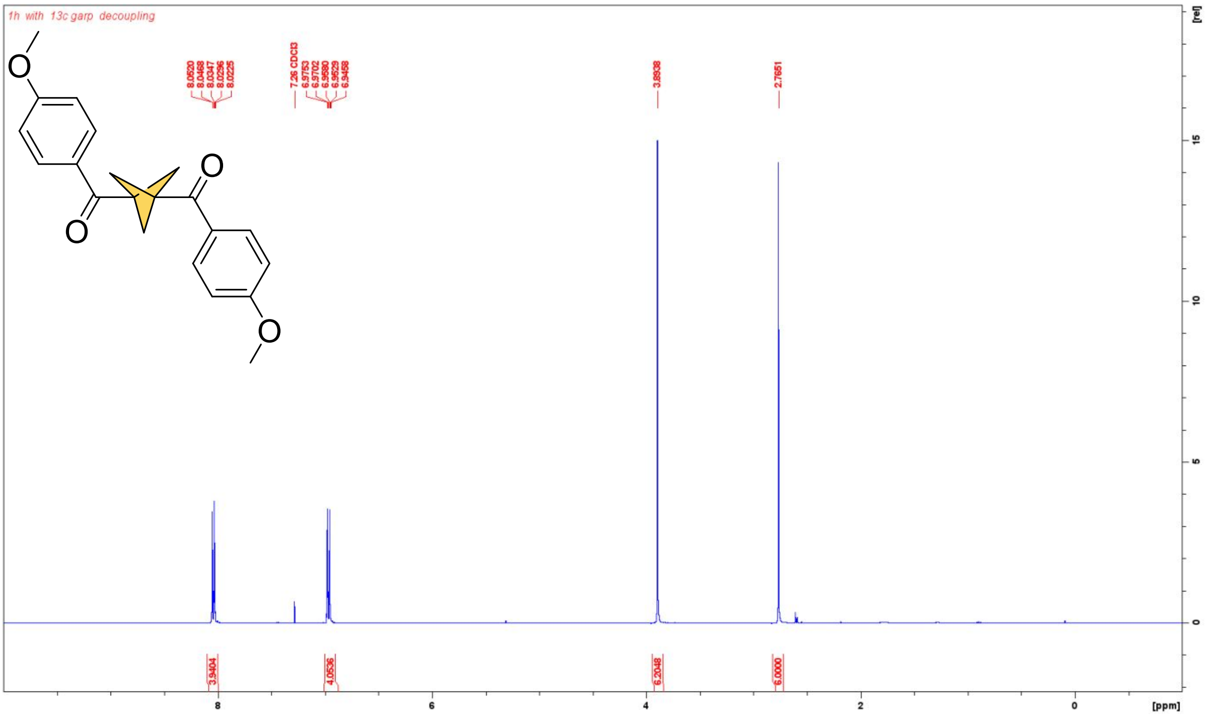

Figure S35: ^{1}H NMR ($CDCl_3$, 400 MHz, 298 K) spectrum of **7a**.

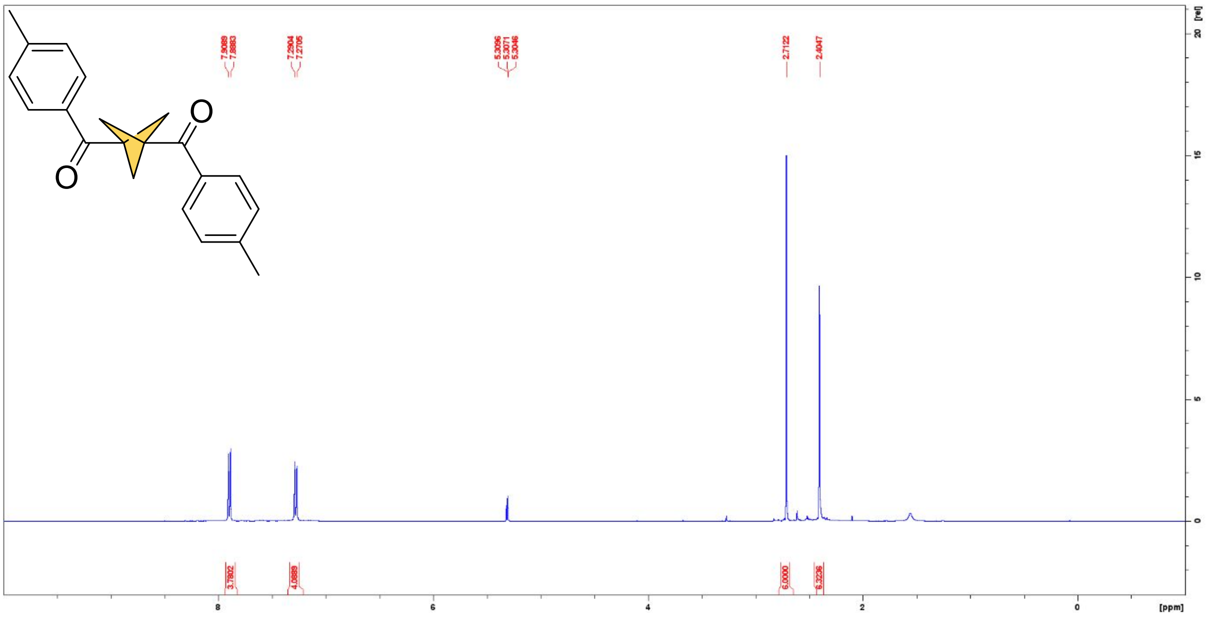

Figure S36: ^{1}H NMR (CD_2Cl_2, 400 MHz, 298 K) spectrum of **7b**.

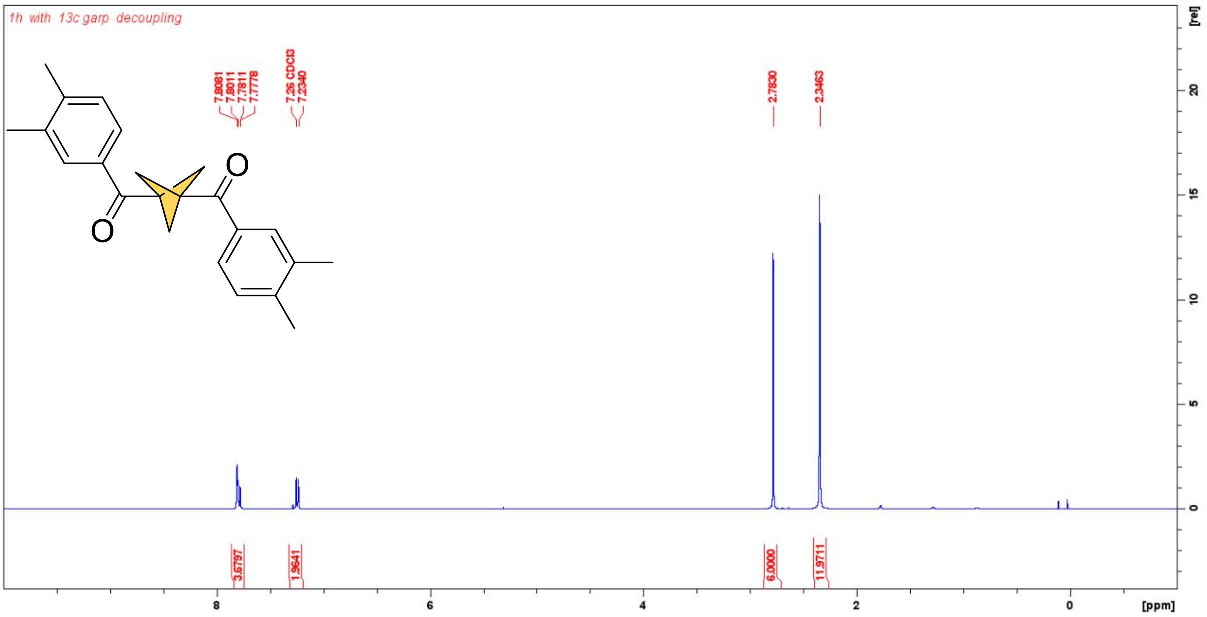

Figure S37: ^{1}H NMR ($CDCl_3$, 400 MHz, 298 K) spectrum of **7c**.

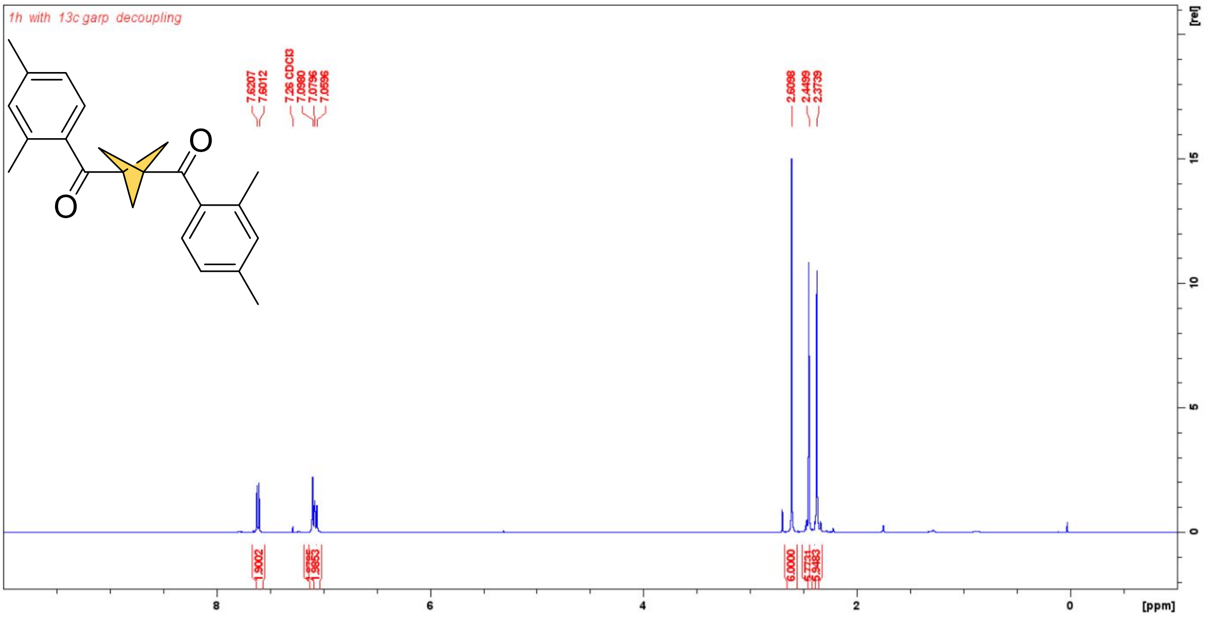

Figure S38: ^{1}H NMR ($CDCl_3$, 400 MHz, 298 K) spectrum of **7d**.

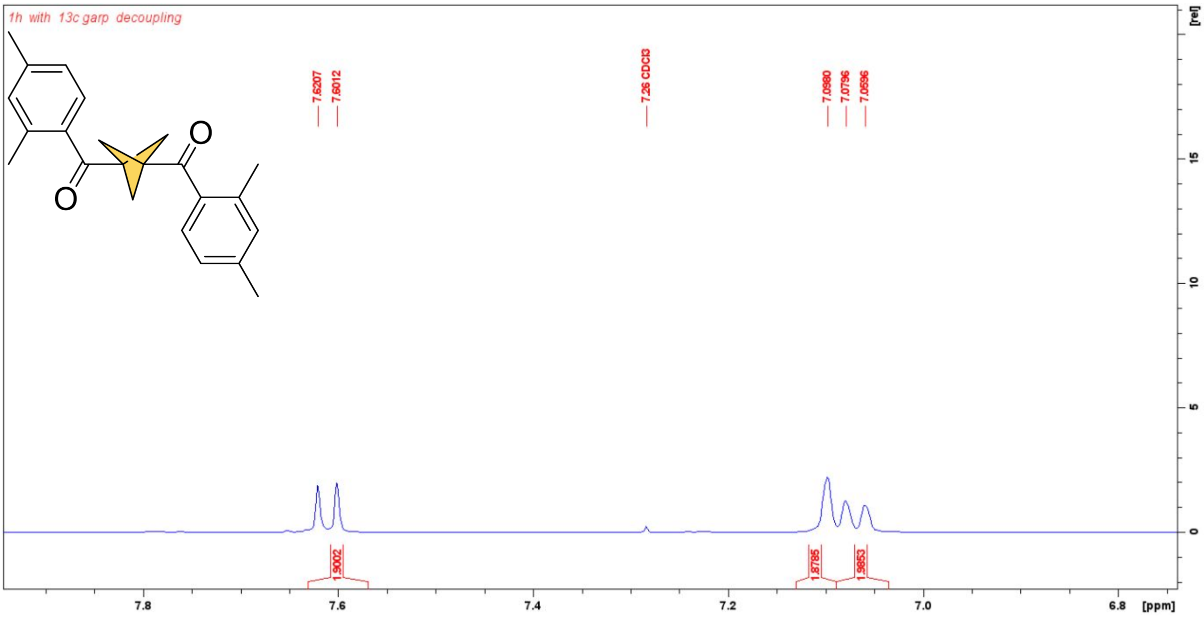

Figure S39: ^{1}H NMR ($CDCl_3$, 400 MHz, 298 K) spectrum of **7d** zoomed in aromatic region.

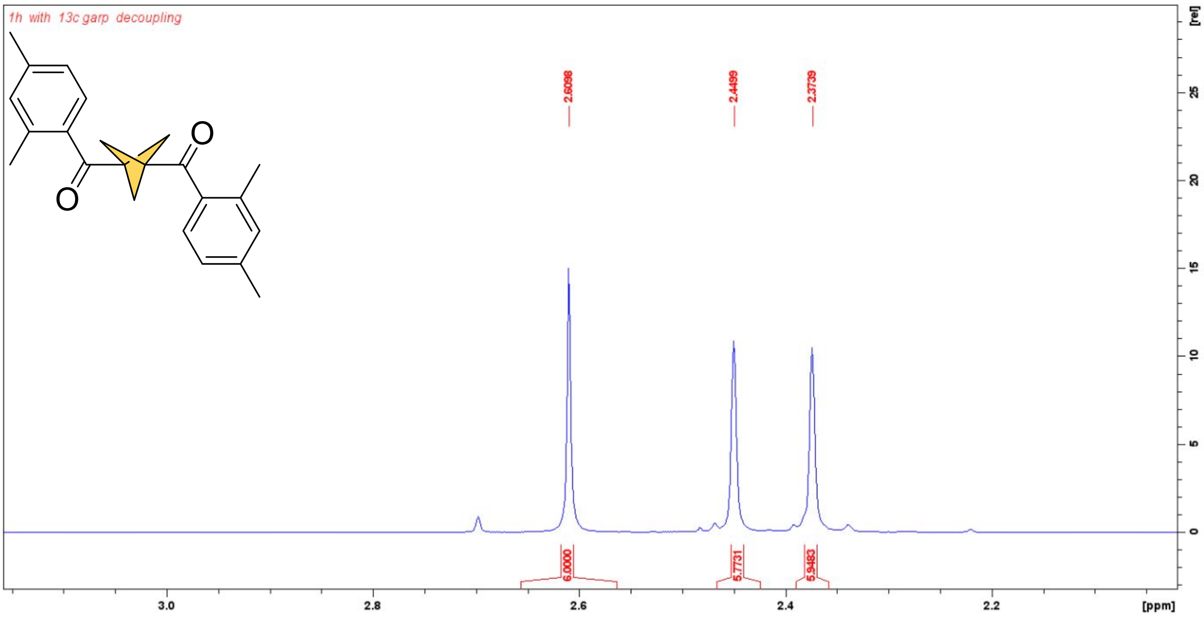

Figure S40: ^{1}H NMR ($CDCl_3$, 400 MHz, 298 K) spectrum of **7d** zoomed in on aliphatic region.

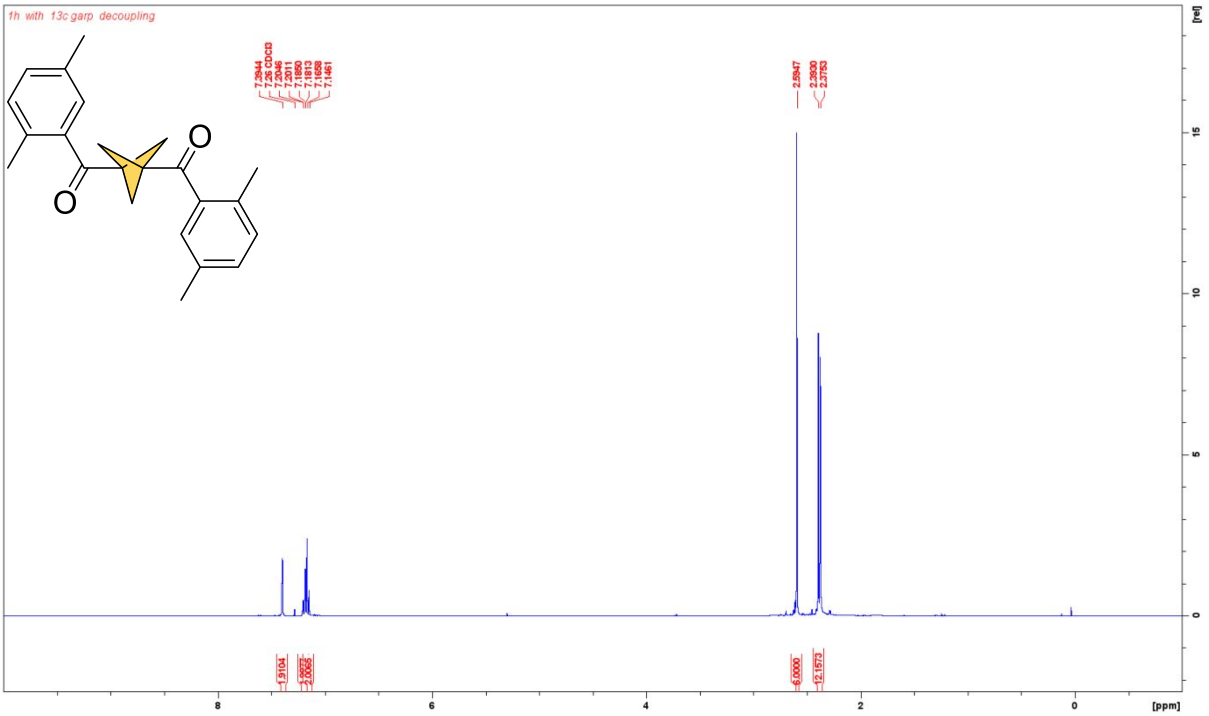

Figure S41: ^{1}H NMR ($CDCl_3$, 400 MHz, 298 K) spectrum of **7e**.

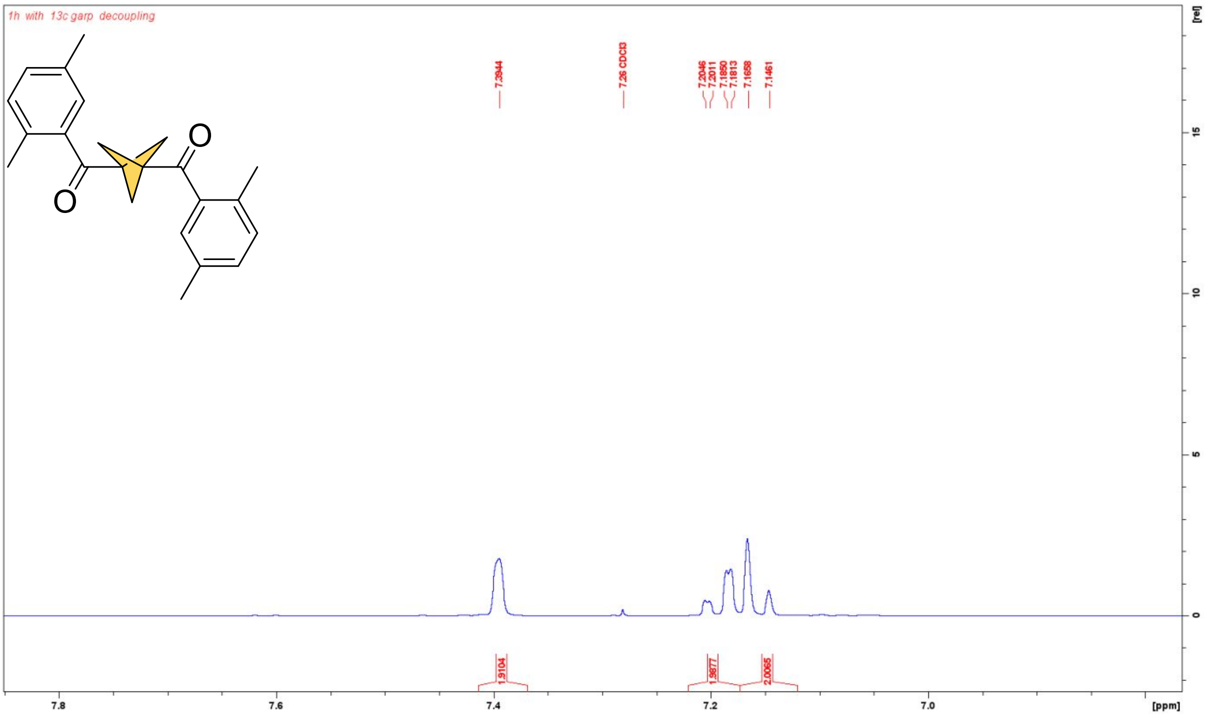

Figure S42: ^{1}H NMR ($CDCl_3$, 400 MHz, 298 K) spectrum of **7e** zoomed in on the aromatic region.

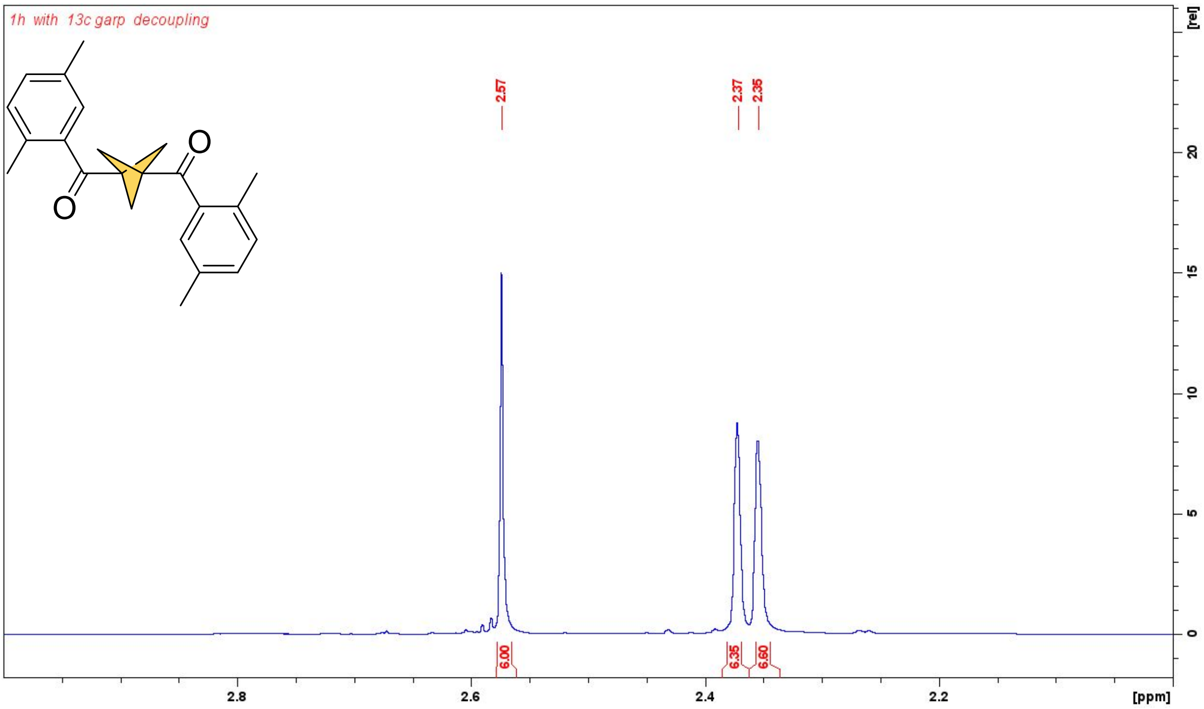

Figure S43: ^{1}H NMR ($CDCl_3$, 400 MHz, 298 K) spectrum of **7e** zoomed in on aromatic region.

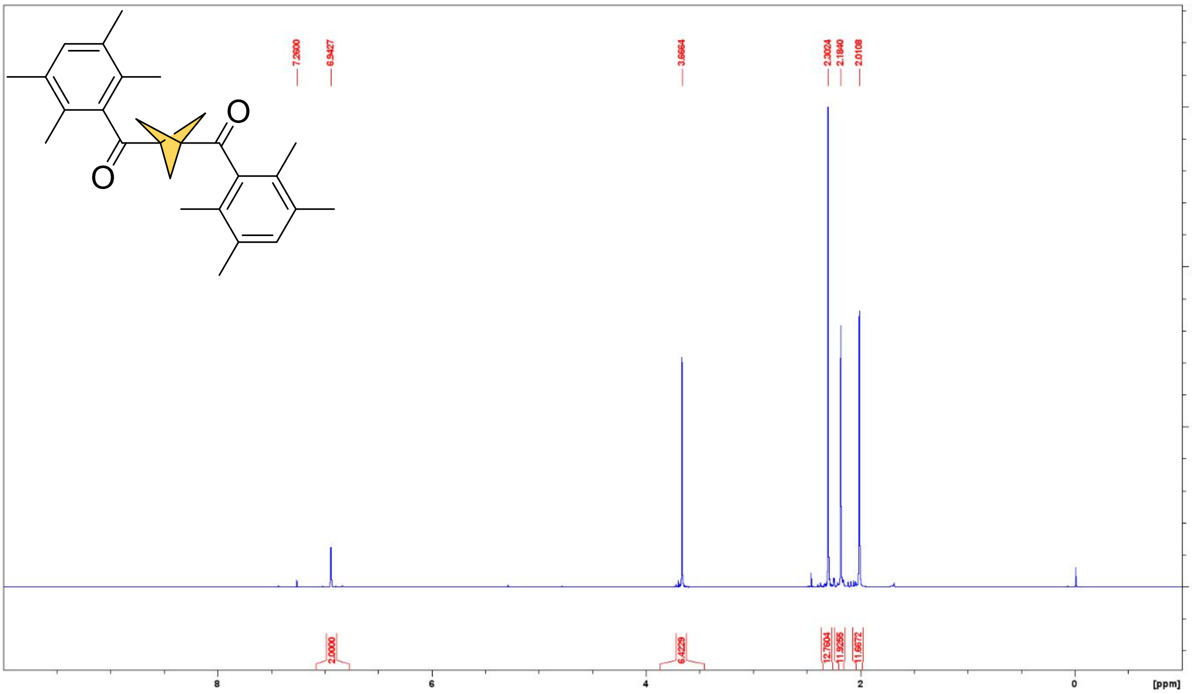

Figure S44: ^{1}H NMR ($CDCl_3$, 400 MHz, 298 K) spectrum of ***7f***.

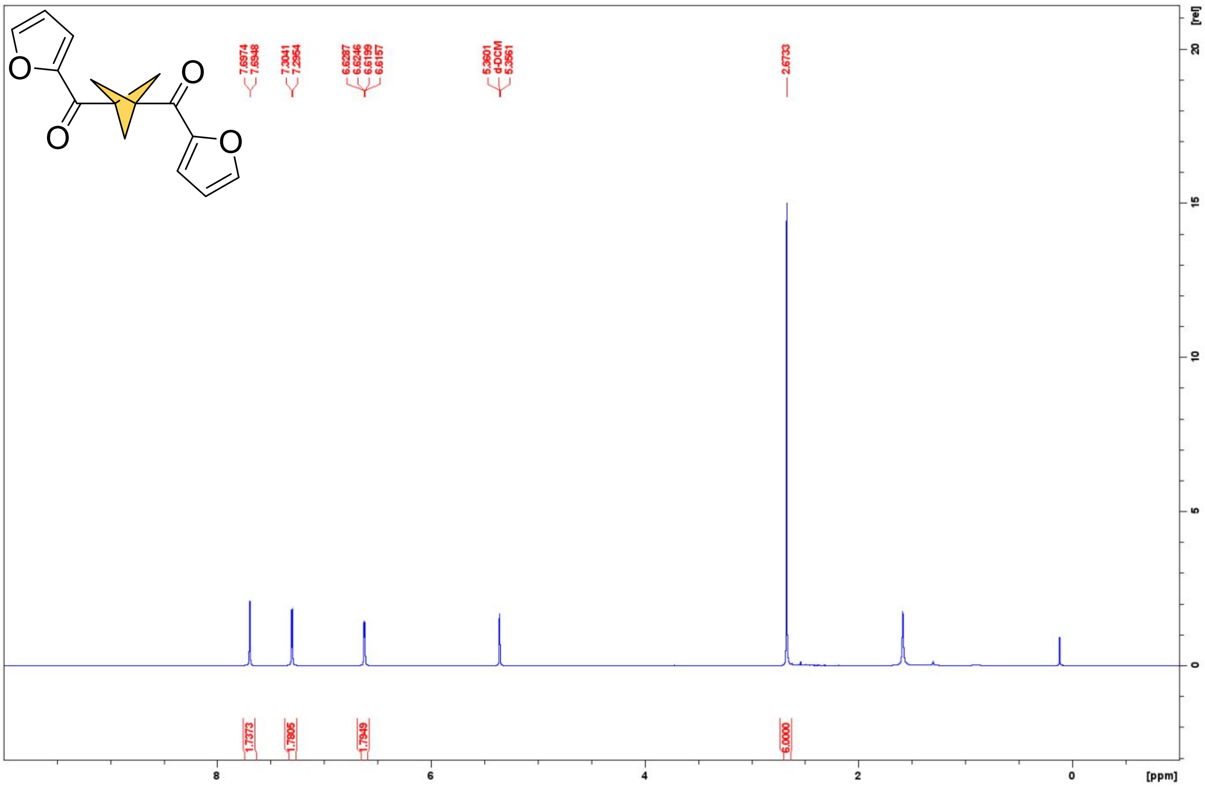

Figure S45: ^{1}H NMR (CD_2Cl_2, 400 MHz, 298 K) spectrum of **7g**.

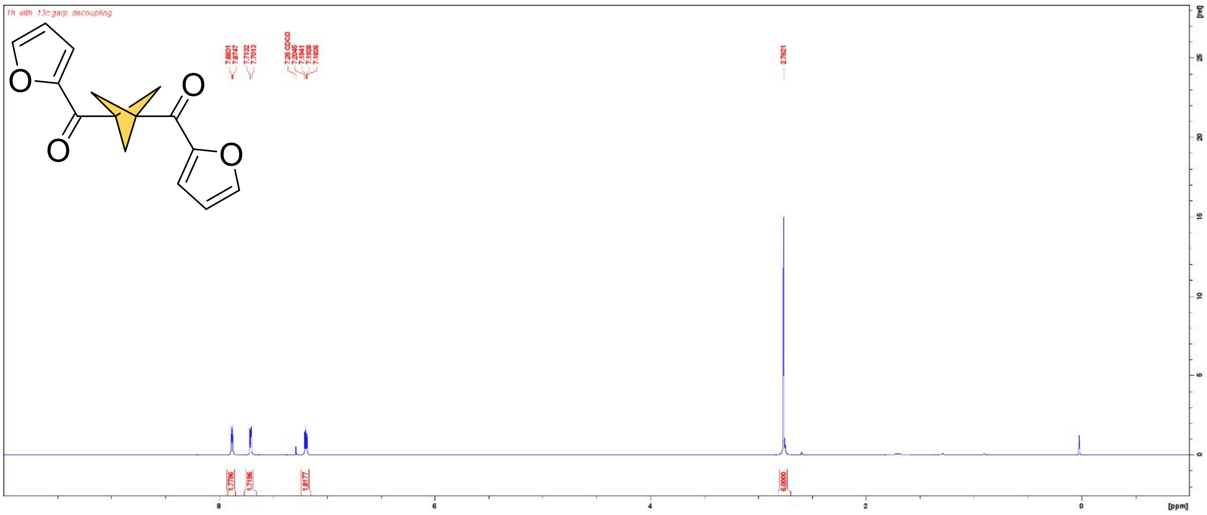

Figure S46: ^{1}H NMR ($CDCl_3$, 400 MHz, 298 K) spectrum of **7h**.

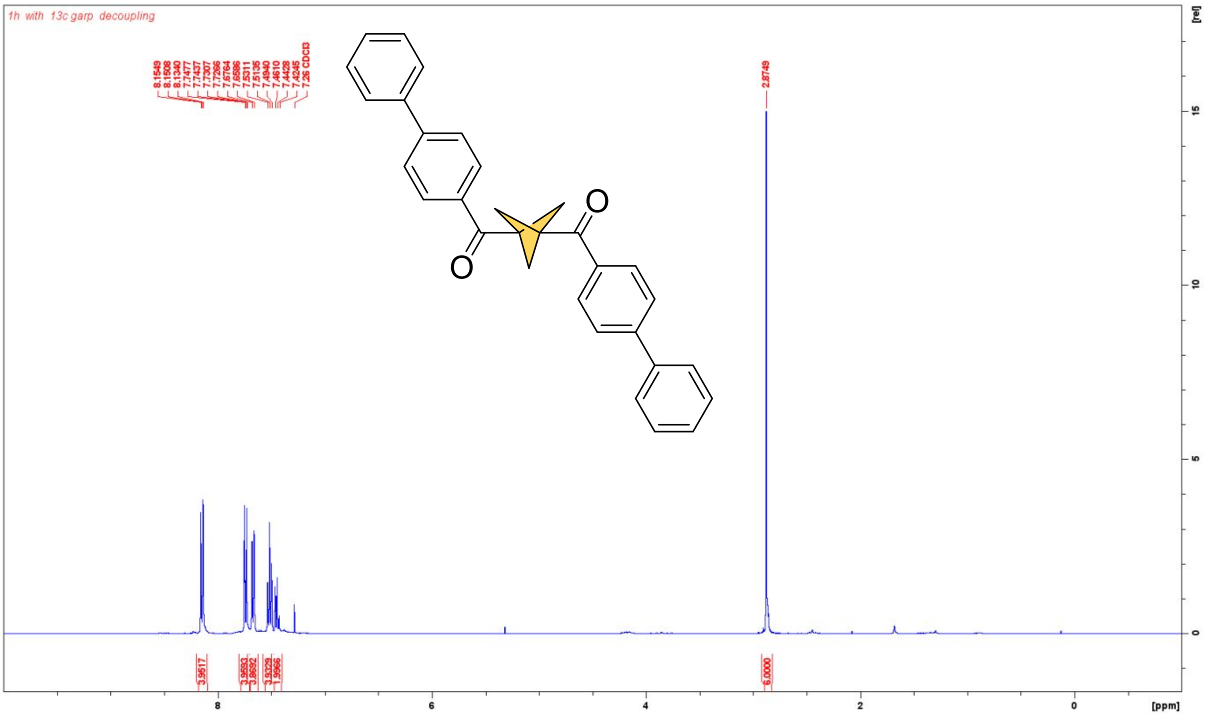

*Figure S47: ^{1}H NMR ($CDCl_3$, 400 MHz, 298 K) spectrum of **7i**.*

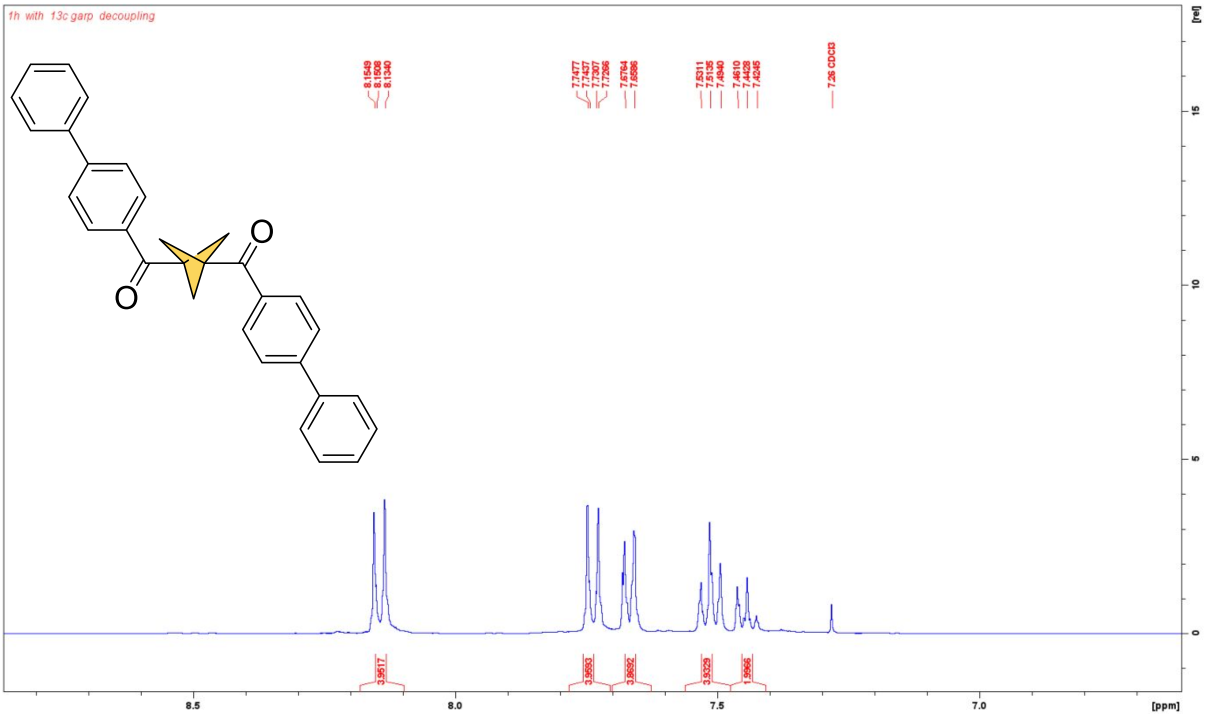

*Figure S48: ^{1}H NMR ($CDCl_3$, 400 MHz, 298 K) spectrum of **7i** zoomed in on the aromatic region.*

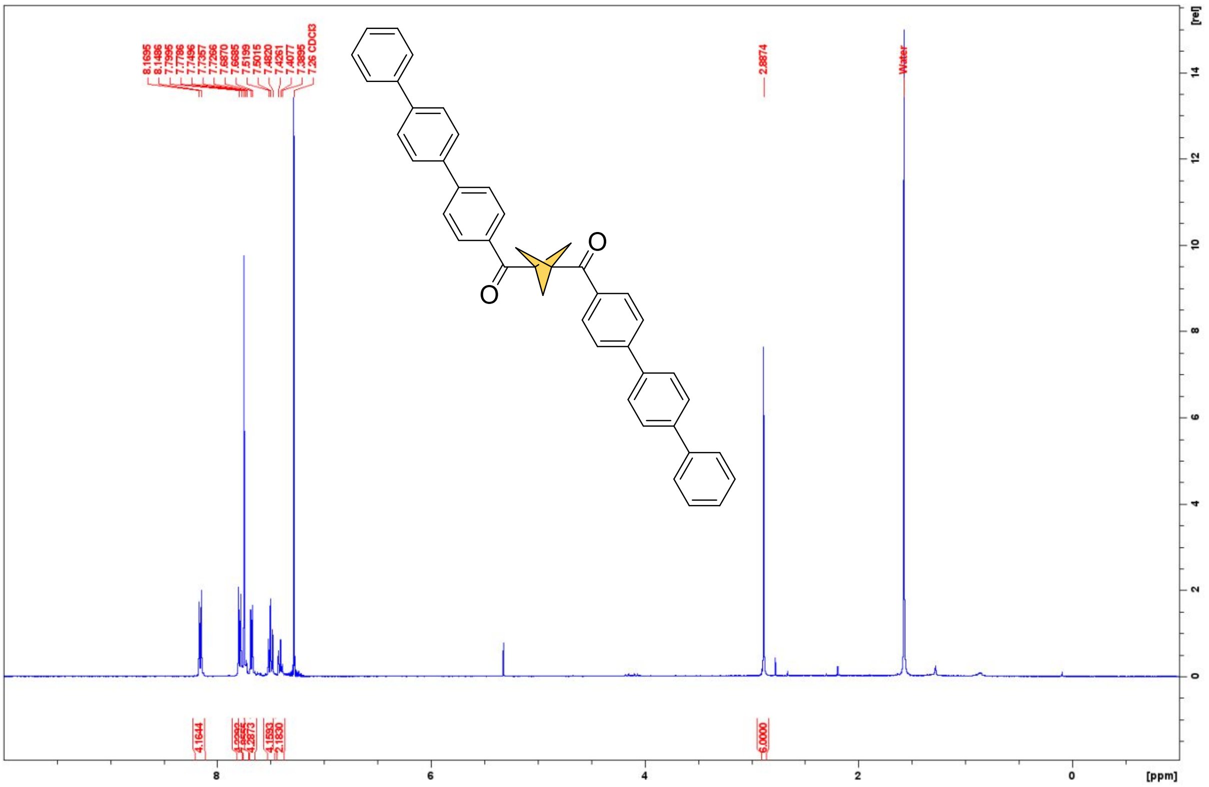

Figure S49: ^{1}H NMR ($CDCl_3$, 400 MHz, 298 K) spectrum of **7j**.

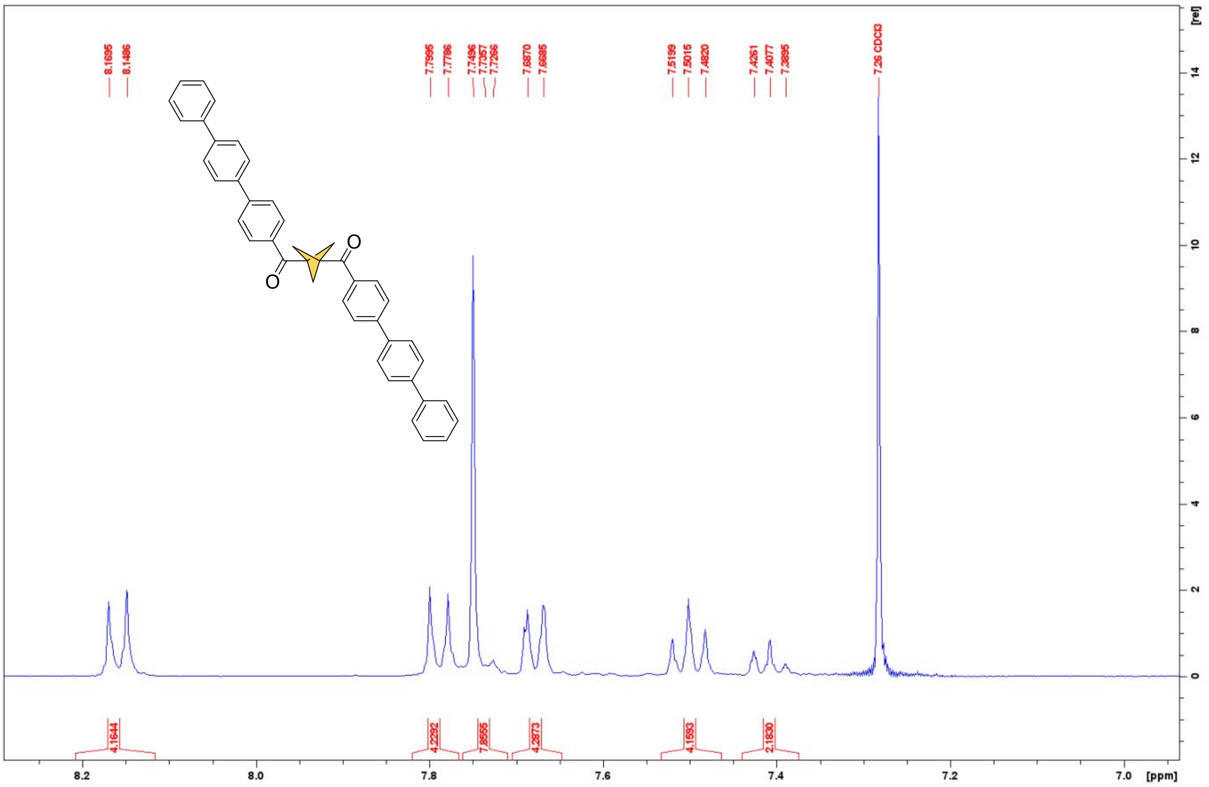

Figure S50: ^{1}H NMR ($CDCl_3$, 400 MHz, 298 K) spectrum of **7j** zoomed in on aromatic region.

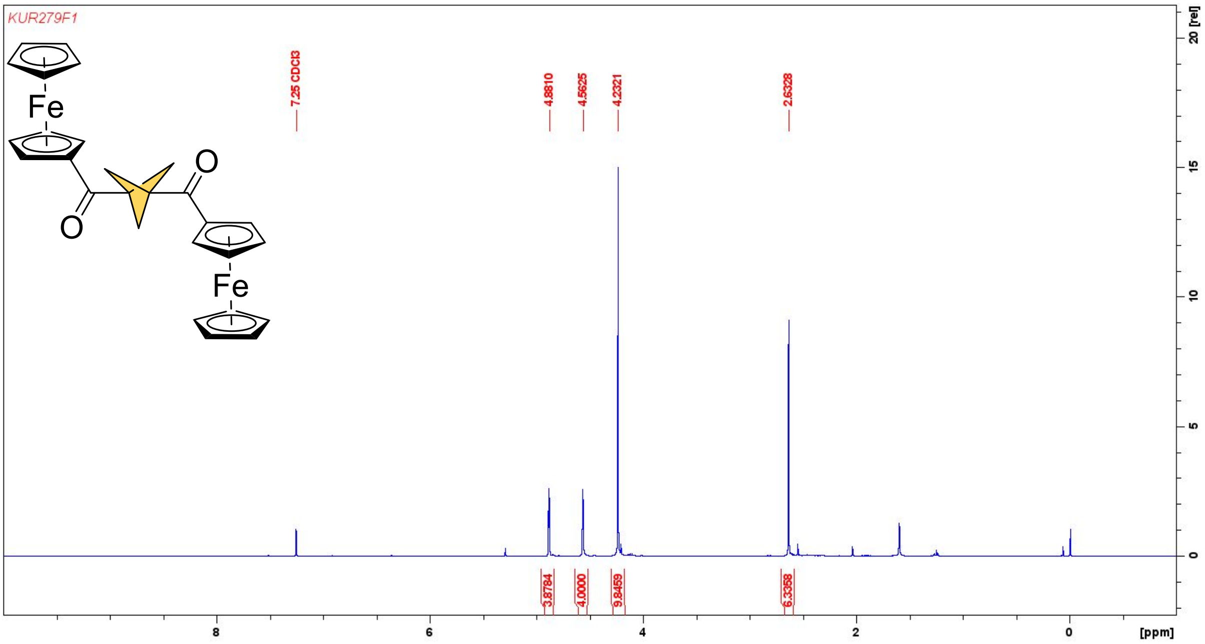

Figure S51: ${}^{1}H$ NMR ($CDCl_3$, 400 MHz, 298 K) spectrum of ***7k***.

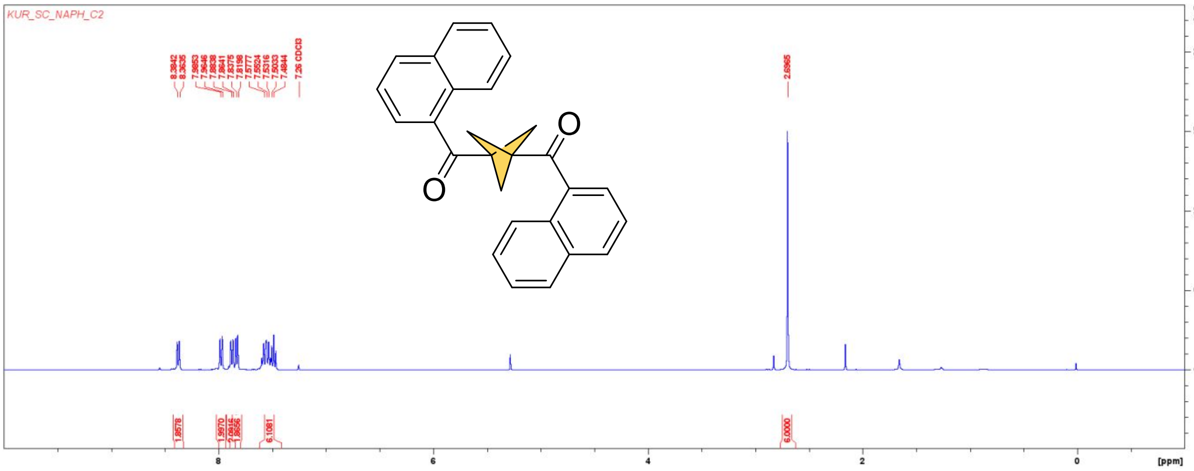

Figure S52: ${}^{1}H$ NMR ($CDCl_3$, 400 MHz, 298 K) spectrum of ***7l.***

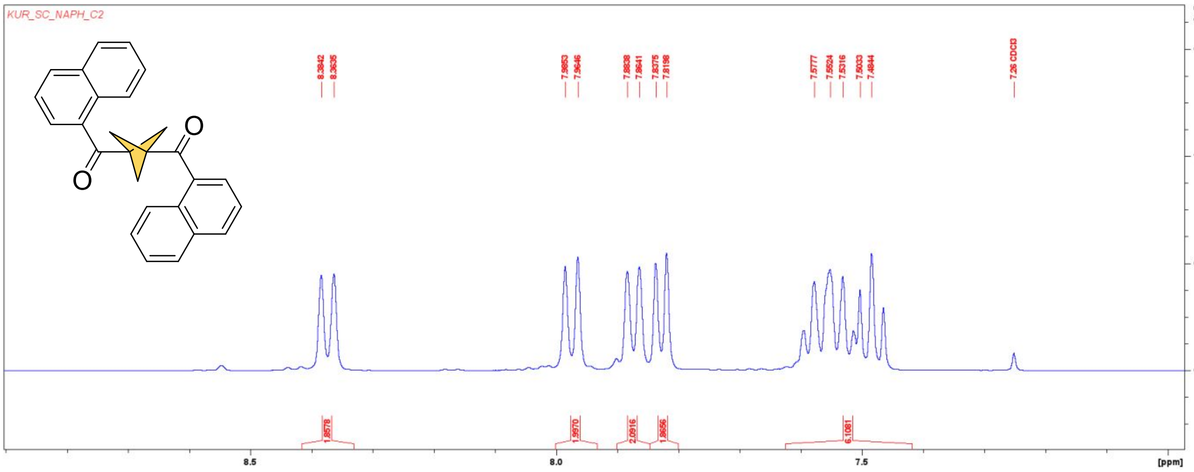

Figure S53: ${}^{1}H$ NMR ($CDCl_3$, 400 MHz, 298 K) spectrum of ***7l*** zoomed in on aromatic region.

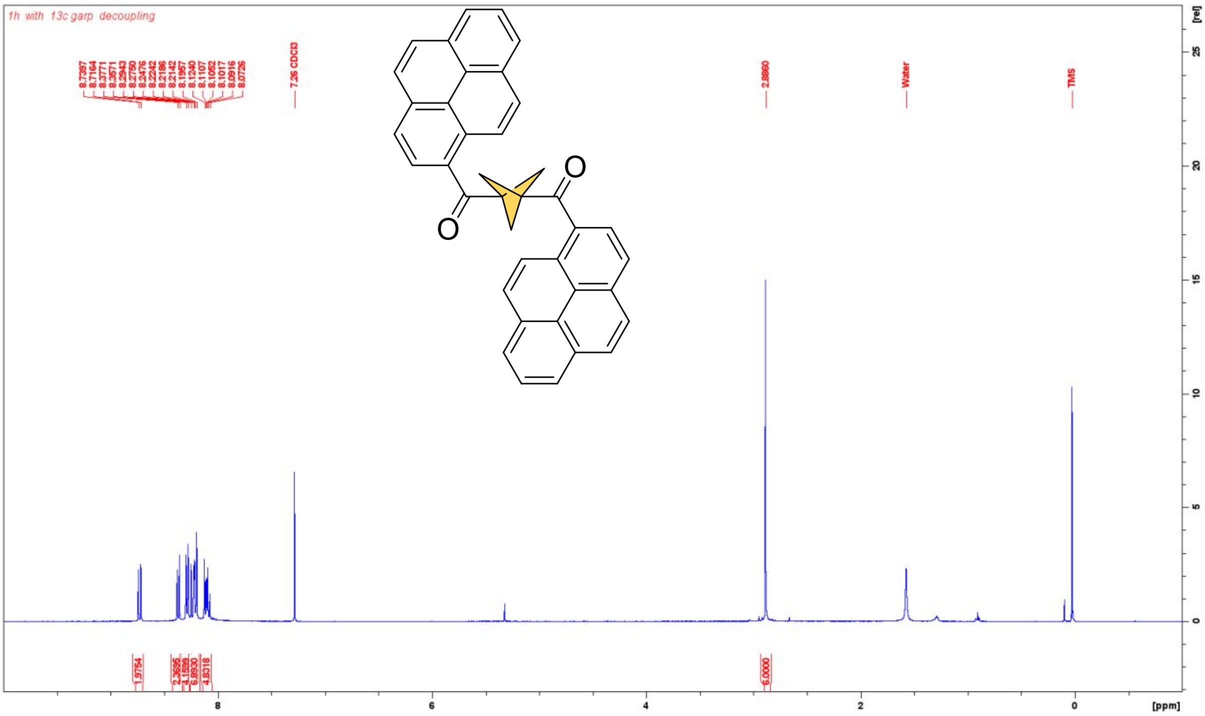

Figure S54: ^{1}H NMR ($CDCl_3$, 400 MHz, 298 K) spectrum of **7m**.

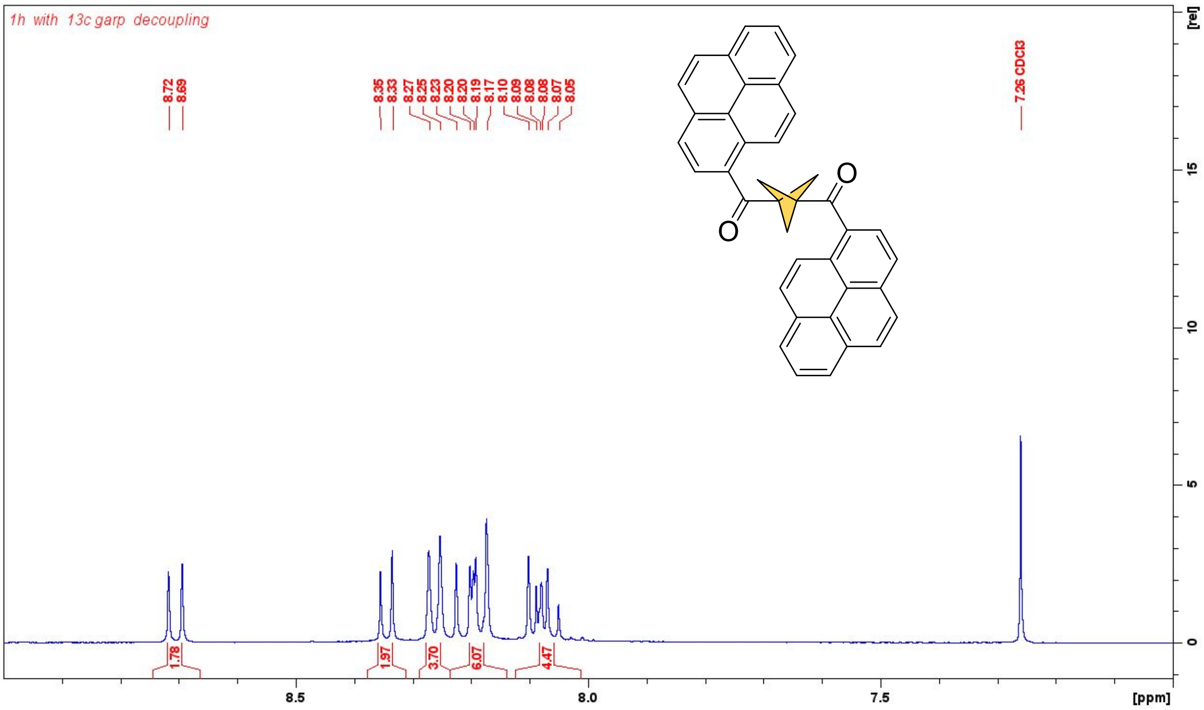

Figure S55: ^{1}H NMR ($CDCl_3$, 400 MHz, 298 K) spectrum of **7m** zoomed in on the aromatic region.

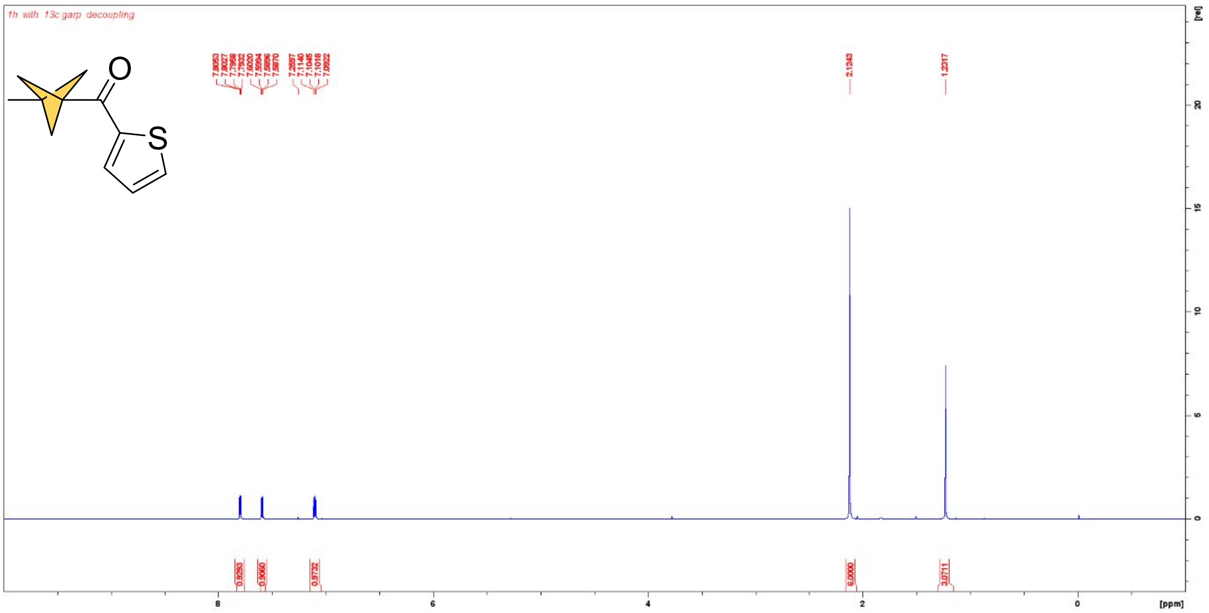

Figure S56: ^{1}H NMR ($CDCl_3$, 400 MHz, 298 K) spectrum of **9b**

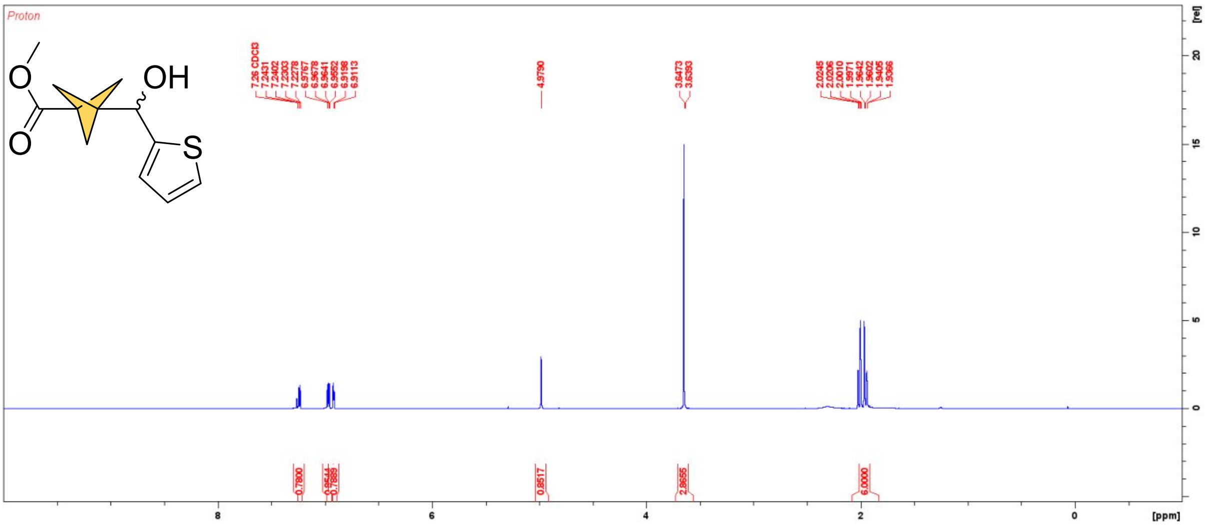

Figure S57: ^{1}H NMR ($CDCl_3$, 400 MHz, 298 K) spectrum of **10**.

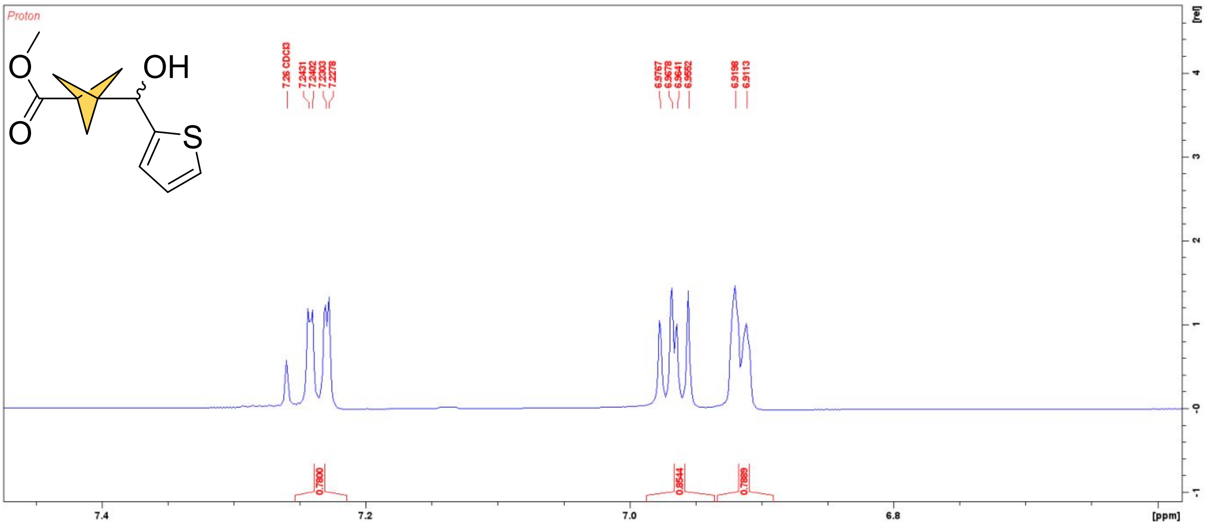

Figure S58: ^{1}H NMR ($CDCl_3$, 400 MHz, 298 K) spectrum of **10** zoomed in on aromatics.

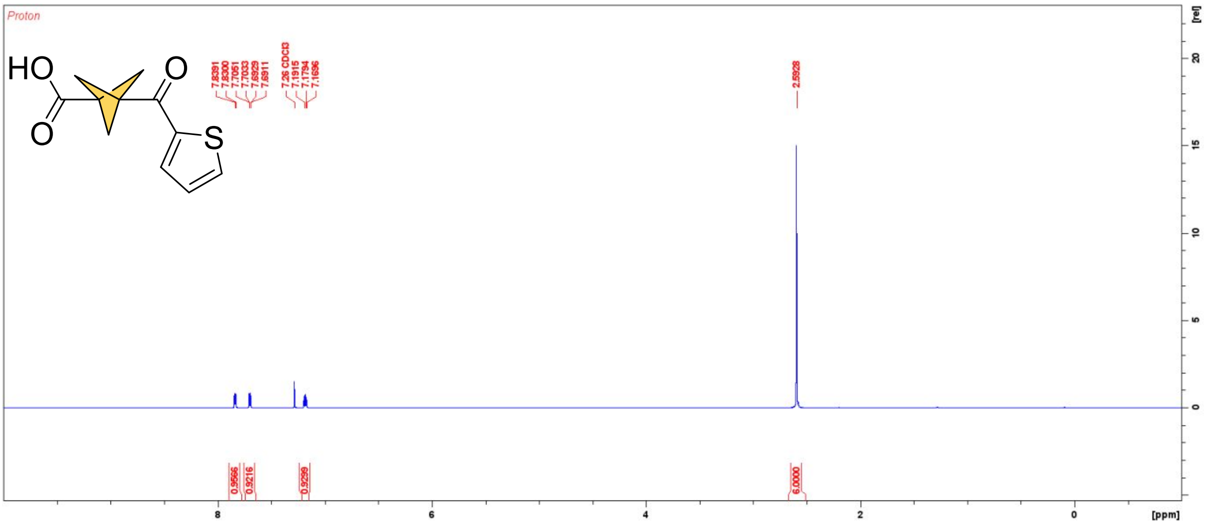

Figure S59: [1]*H NMR (CDCl*$_3$*, 400 MHz, 298 K) spectrum of* ***11***.

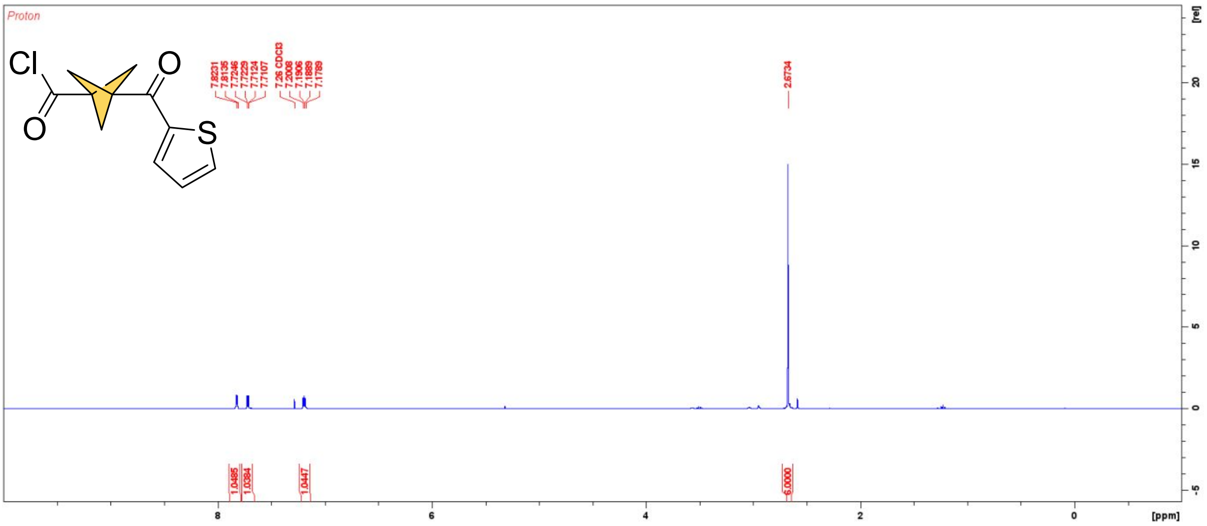

Figure S60: [1]*H NMR (CDCl*$_3$*, 400 MHz, 298K) spectrum of* ***12.***

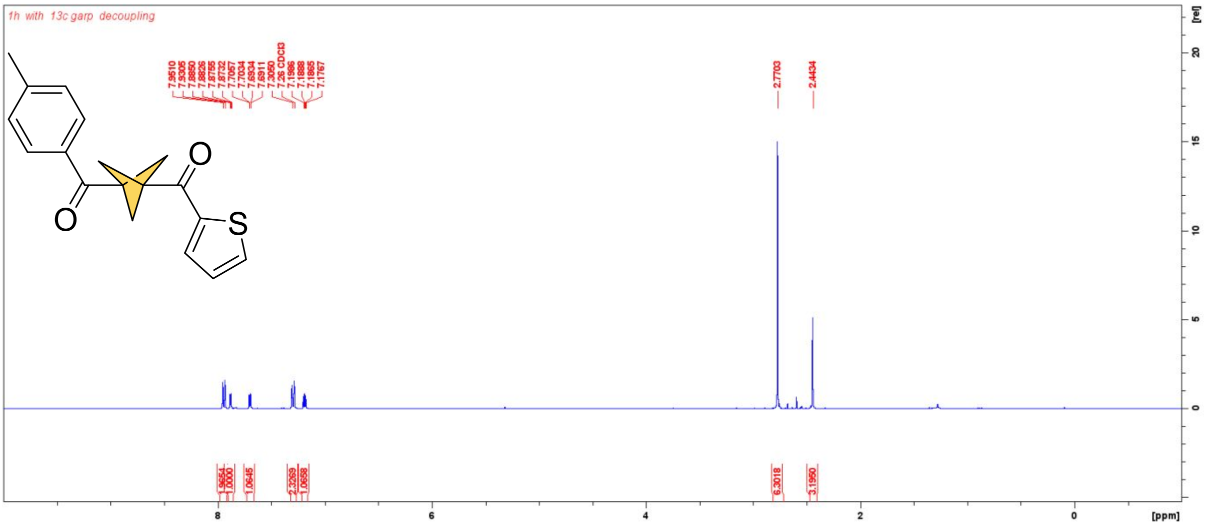

Figure S61: [1]*H NMR (CDCl*$_3$*, 400 MHz, 298K) spectrum of* ***13.***

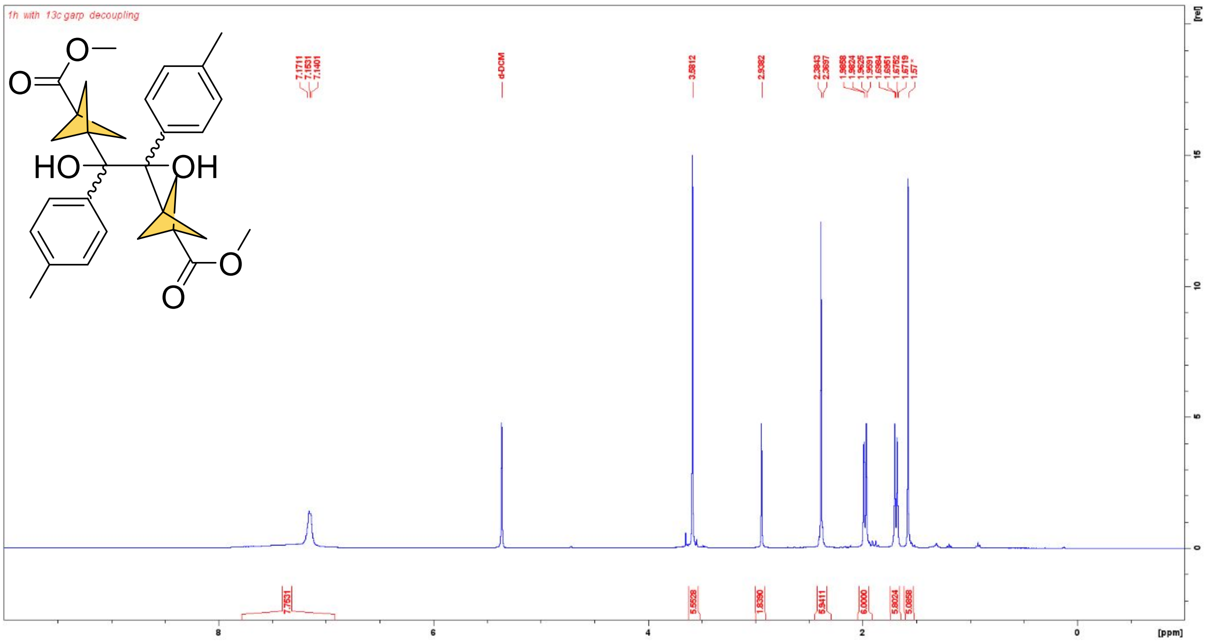

Figure S62: ^{1}H NMR ($CDCl_3$, 400 MHz, 298 K) spectrum of ***14a****.*

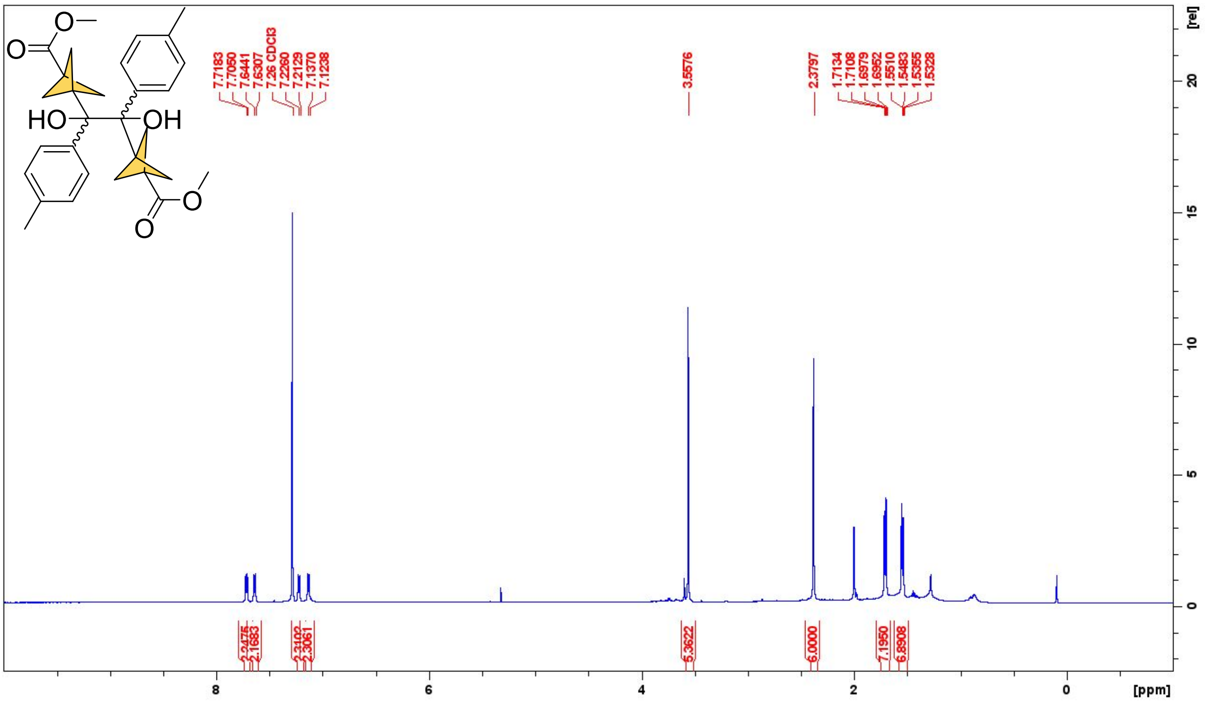

Figure S63: ^{1}H NMR ($CDCl_3$, 600 MHz, 298 K) spectrum of ***14b****.*

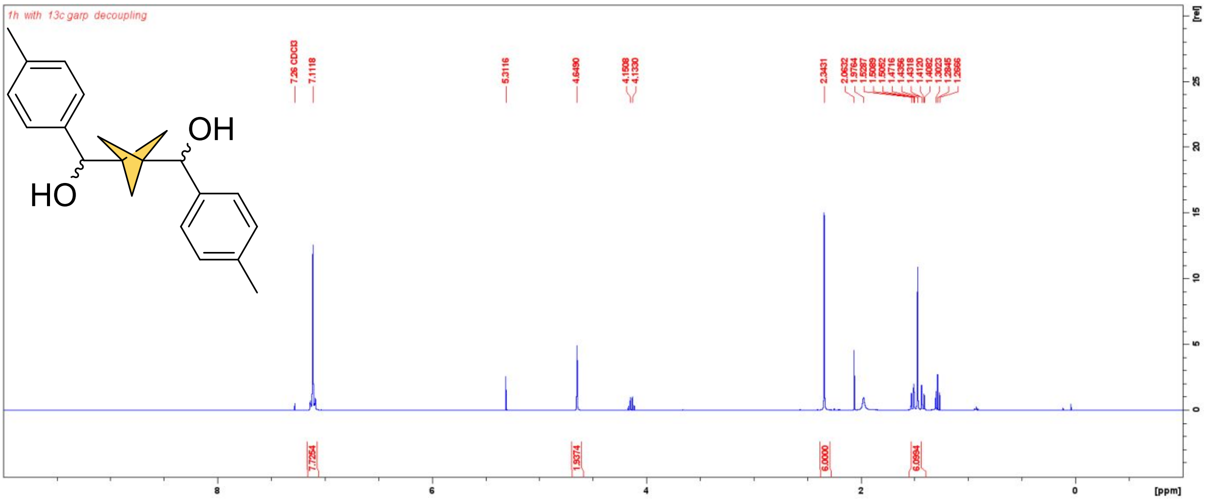

Figure S64: ^{1}H NMR ($CDCl_3$, 400 MHz, 298 K) spectrum of **15**.

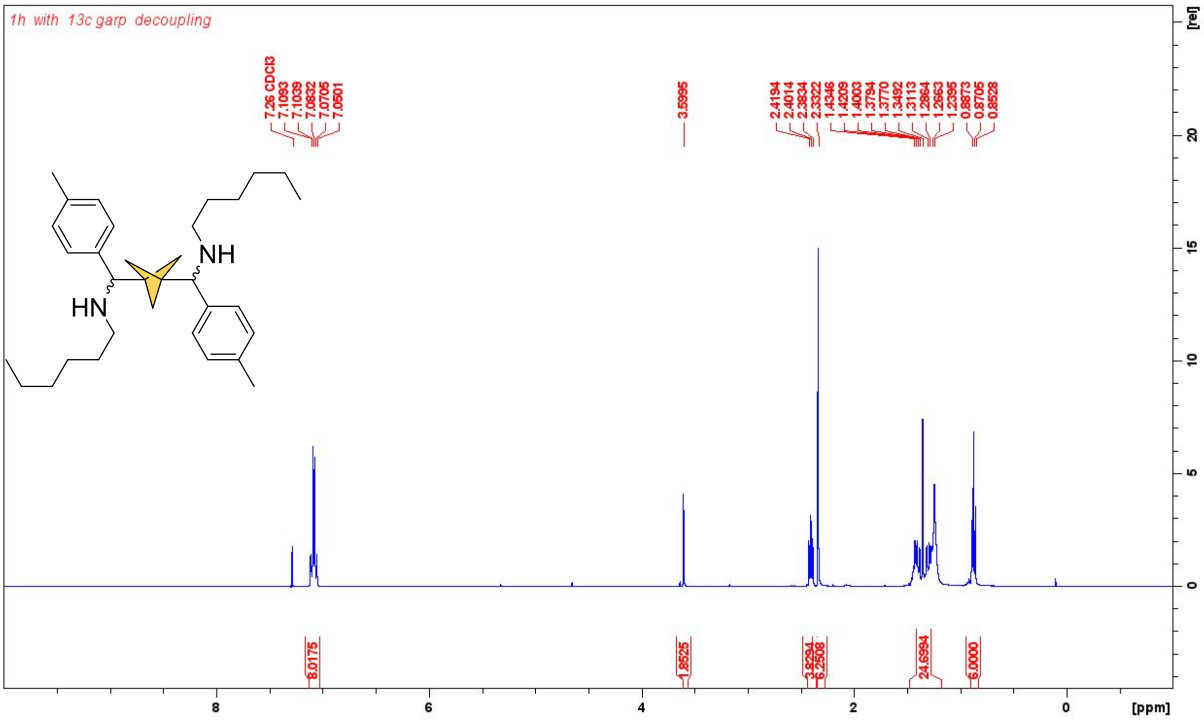

Figure S65: ^{1}H NMR ($CDCl_3$, 400 MHz, 298 K) spectrum of **16**.

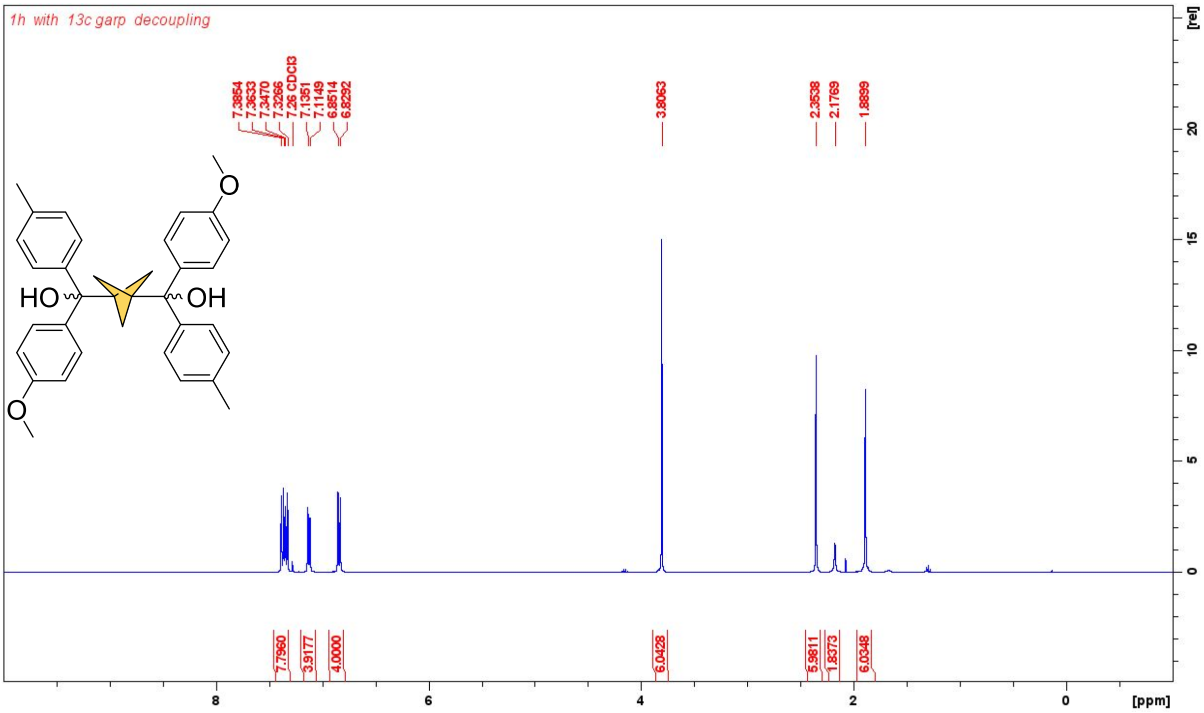

Figure S66: ^{1}H NMR ($CDCl_3$, 400 MHz, 298 K) spectrum of ***17***.

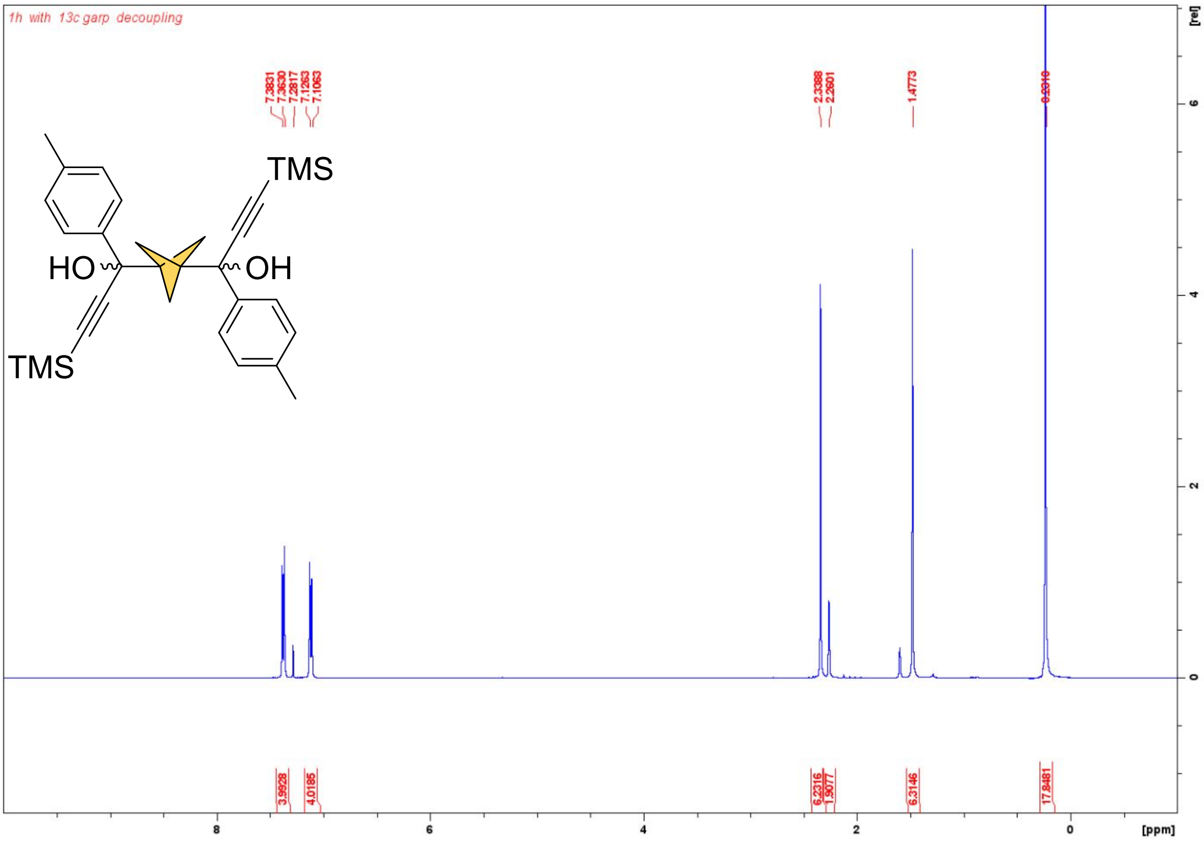

Figure S67: ^{1}H NMR ($CDCl_3$, 400 MHz, 298 K) spectrum of ***18***.

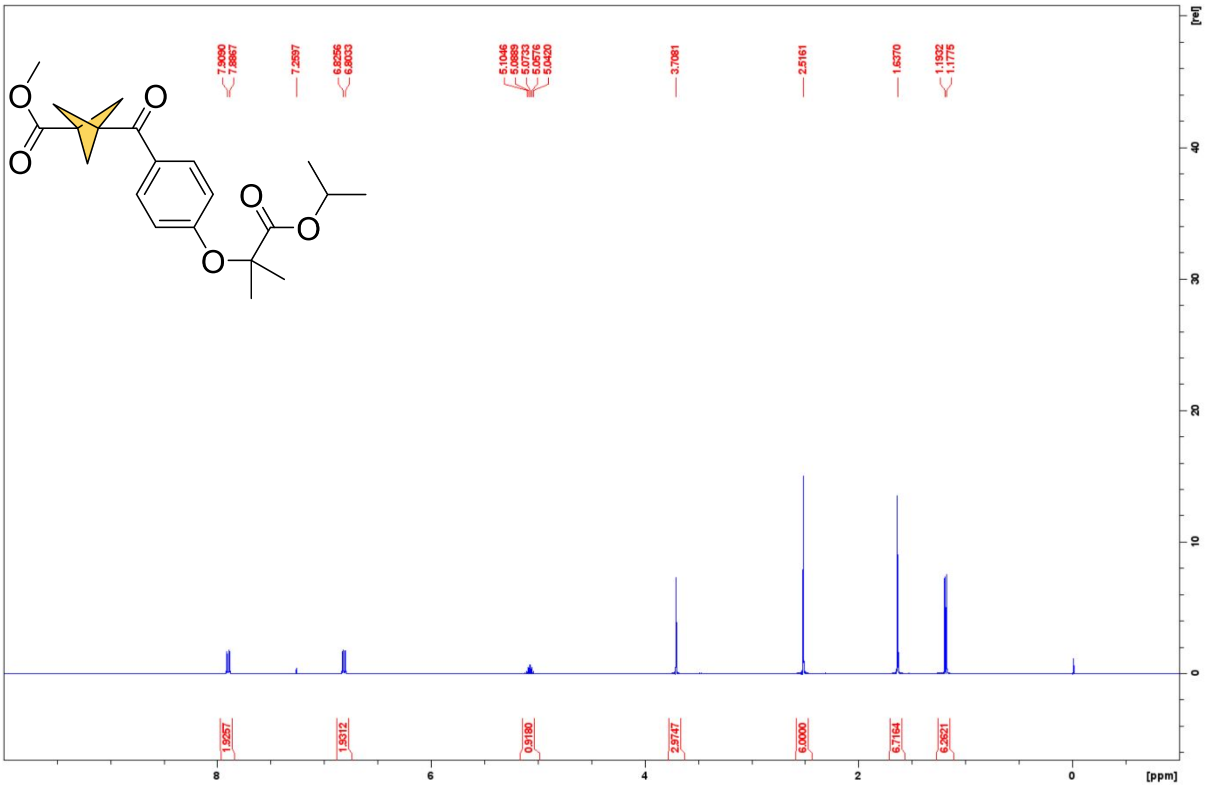

*Figure S68: ^{1}H NMR ($CDCl_3$, 400 MHz, 298 K) spectrum of **20a**.*

^{13}C{^{1}H} NMR spectra

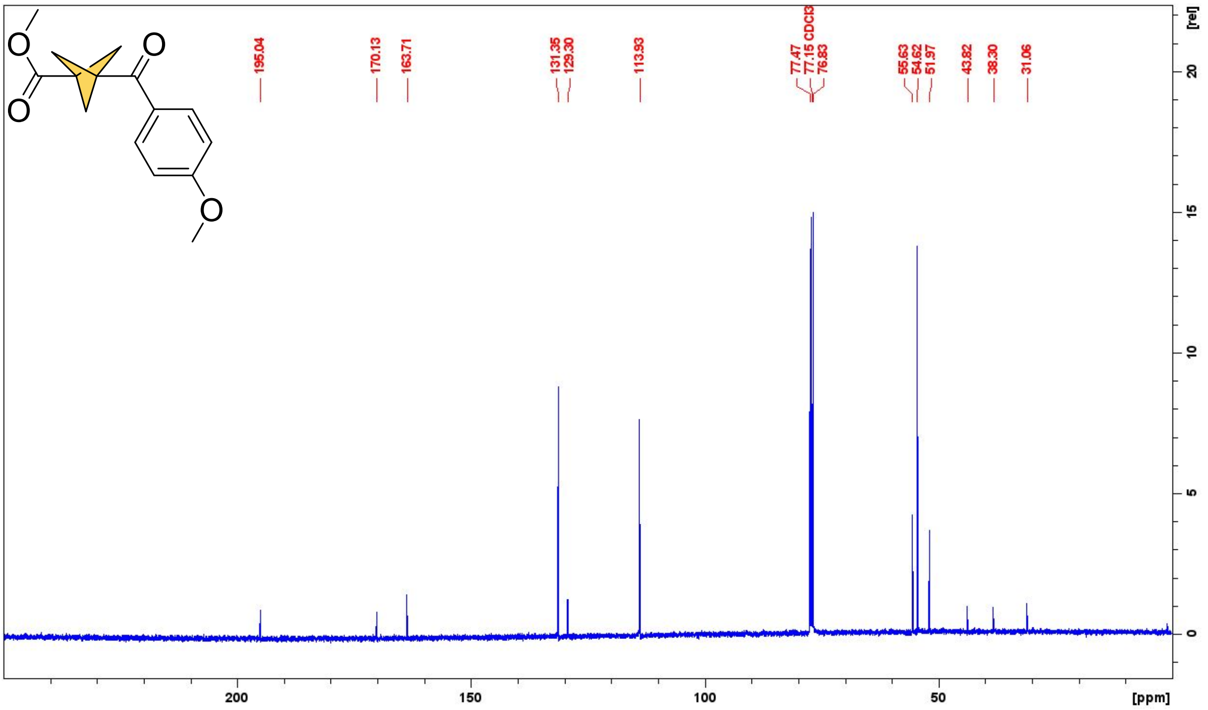

*Figure S69: ^{13}C{^{1}H} NMR ($CDCl_3$, 101 MHz, 298 K) spectrum of **6a**.*

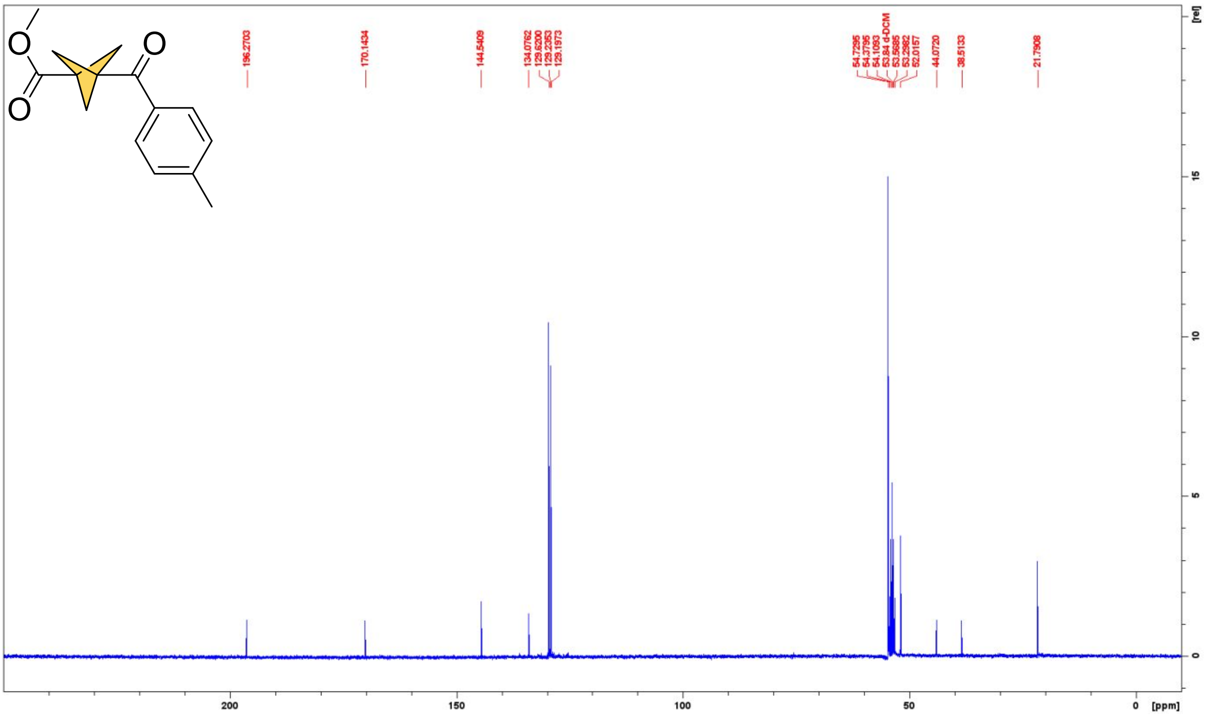

*Figure S70: $^{13}C\{^{1}H\}$ NMR (CD_2Cl_2, 101 MHz, 298 K) spectrum of **6b**.*

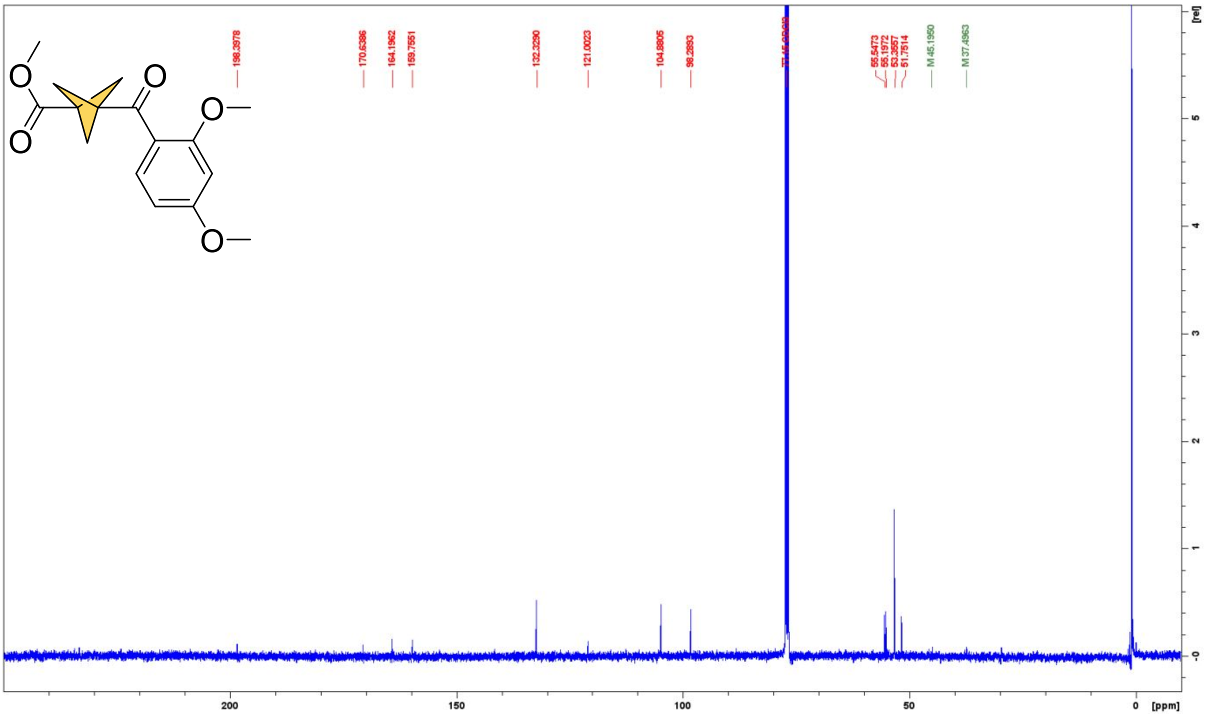

*Figure S71: $^{13}C\{^{1}H\}$ NMR ($CDCl_3$, 101 MHz, 298 K) spectrum of **6c**.*

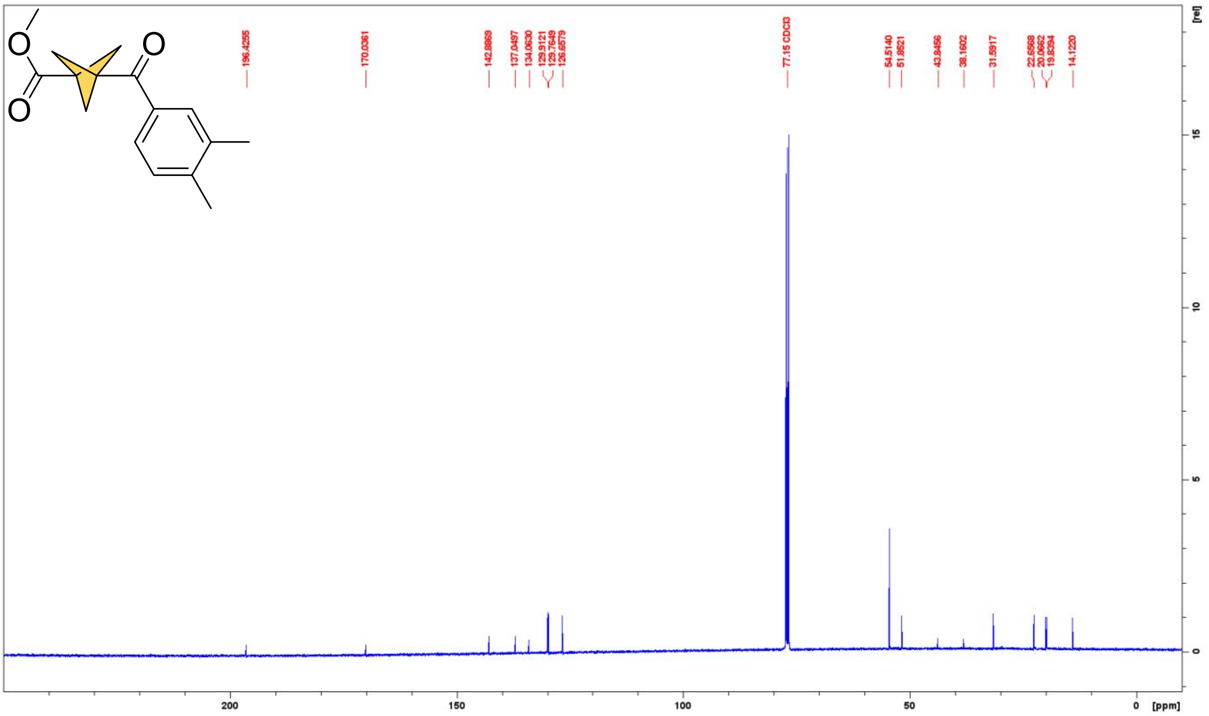

Figure S72: $^{13}C\{^{1}H\}$ NMR ($CDCl_3$, 101 MHz, 298 K) spectrum of ***6d.***

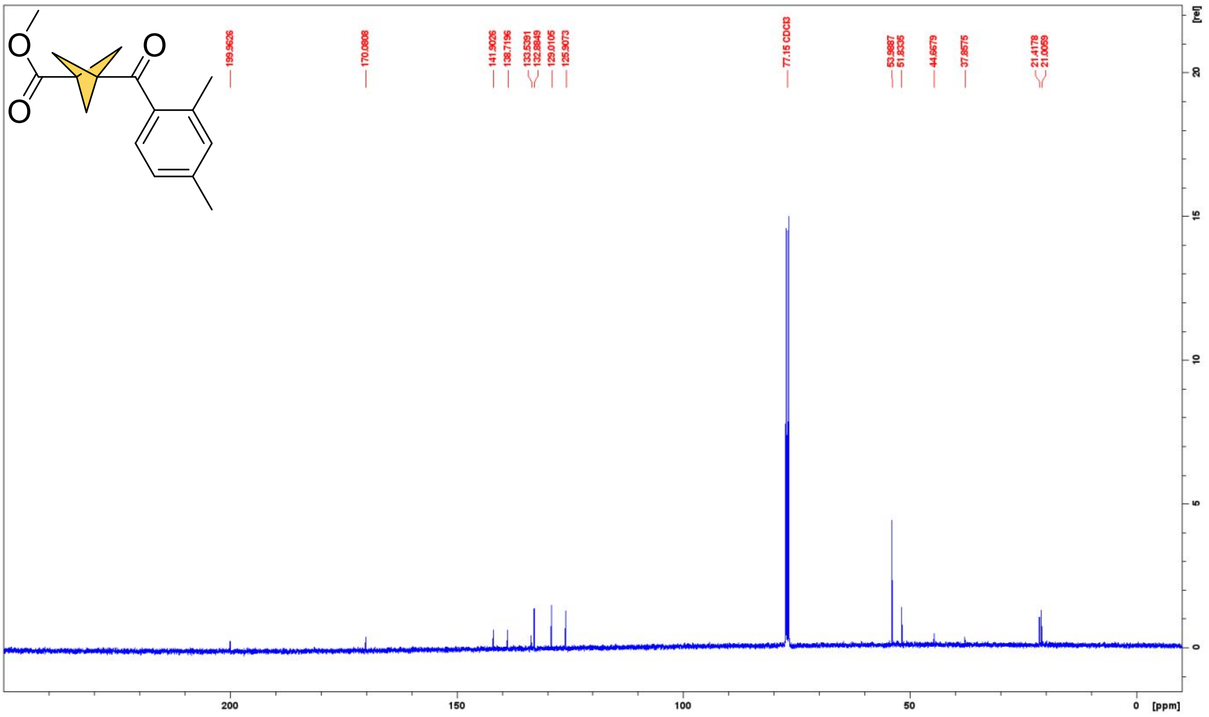

Figure S73: $^{13}C\{^{1}H\}$ NMR ($CDCl_3$, 101 MHz, 298 K) spectrum of ***6e.***

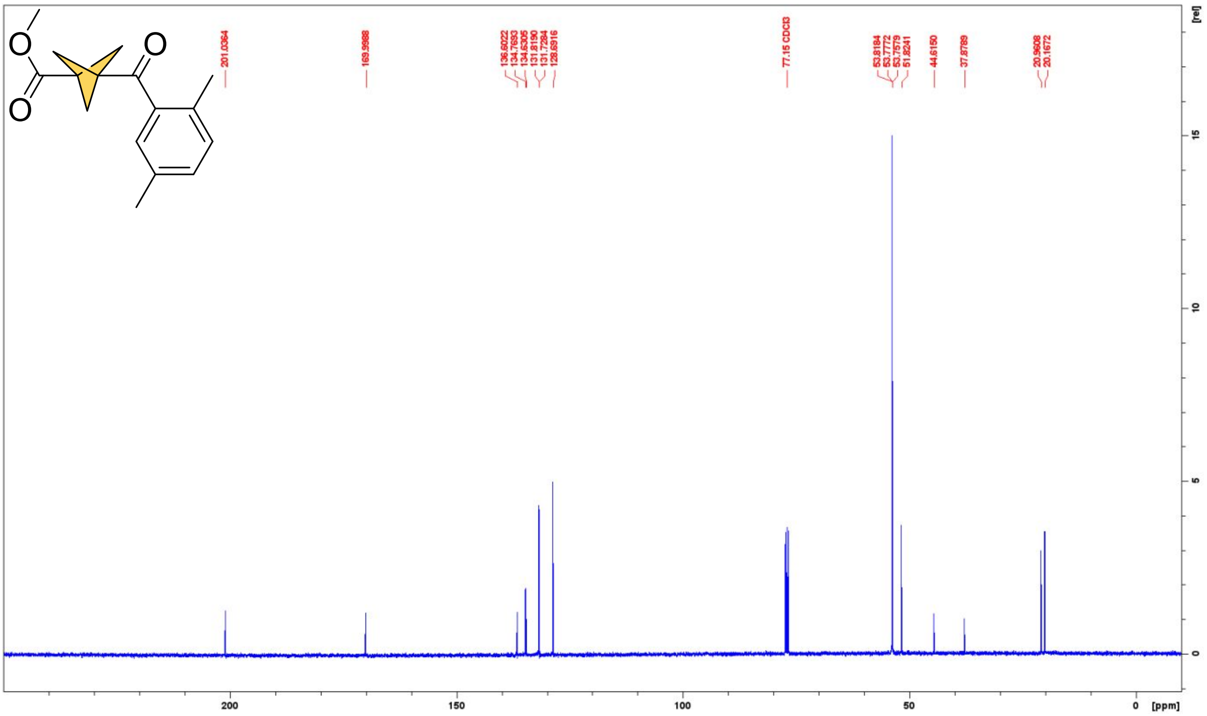

Figure S74: $^{13}C\{^{1}H\}$ NMR ($CDCl_3$, 101 MHz, 298 K) spectrum of ***6f***.

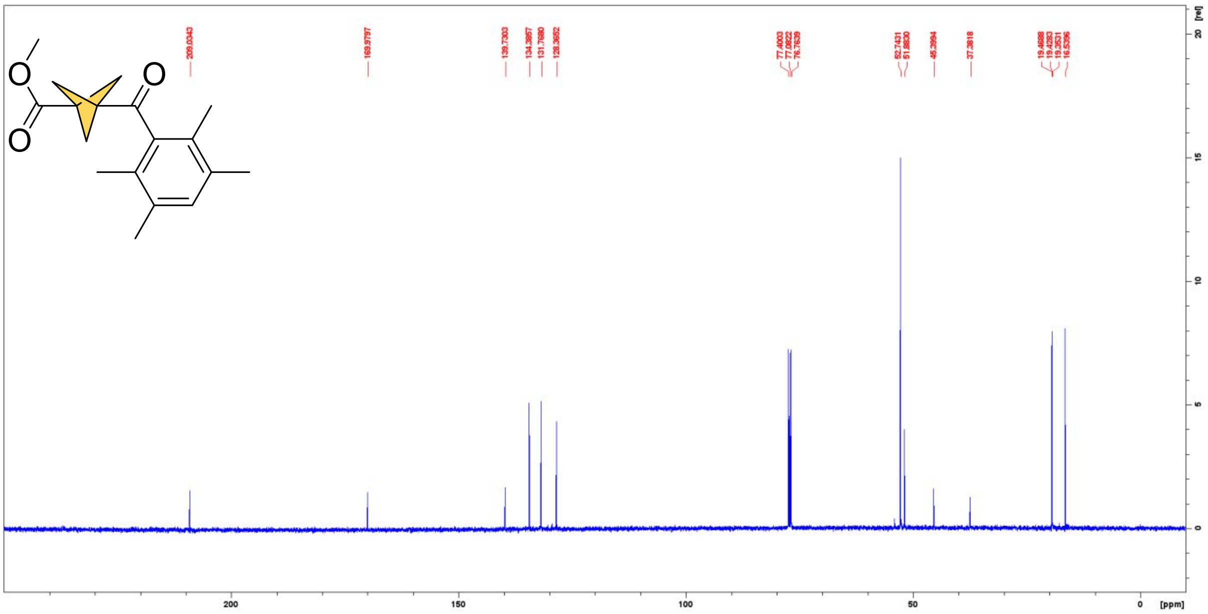

Figure S75: $^{13}C\{^{1}H\}$ NMR ($CDCl_3$, 101 MHz, 298 K) spectrum of ***6g***.

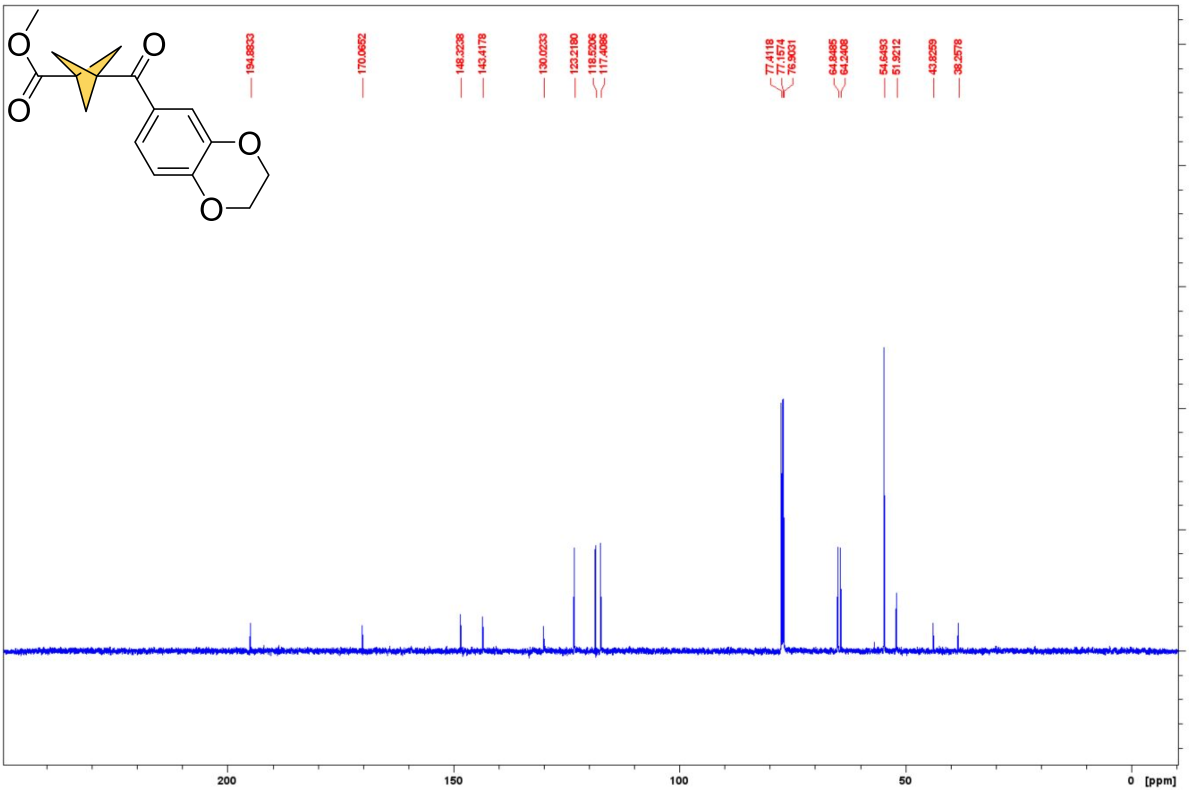

Figure S76: $^{13}C\{^1H\}$ NMR ($CDCl_3$, 126 MHz, 298 K) spectrum of ***6h****.*

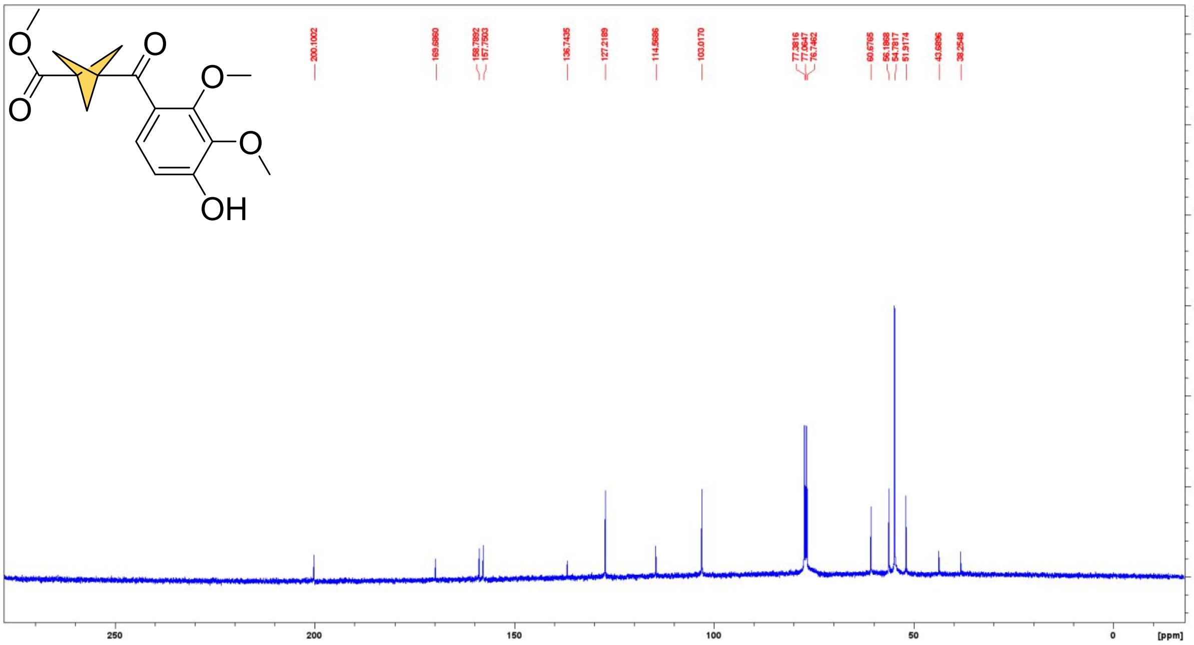

Figure S77 : $^{13}C\{^1H\}$ NMR ($CDCl_3$, 101 MHz, 298 K) spectrum of ***6i****.*

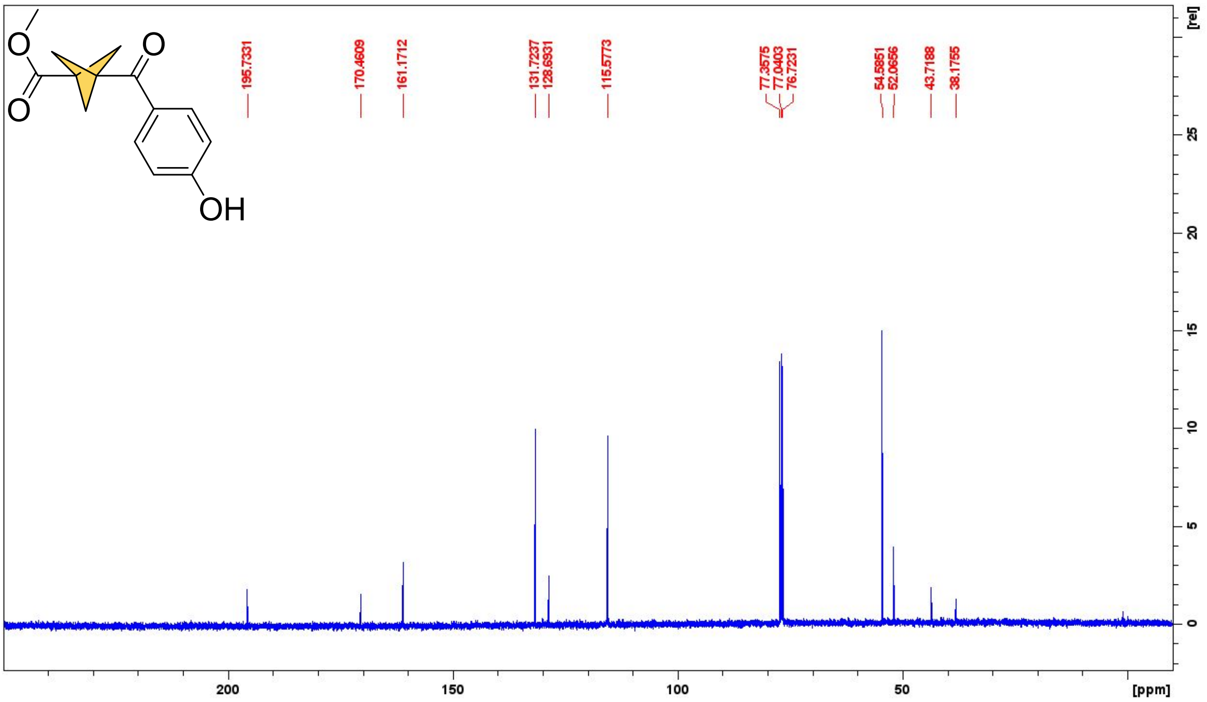

Figure S78: $^{13}C\{^{1}H\}$ NMR ($CDCl_3$, 101 MHz, 298 K) spectrum of ***6j.***

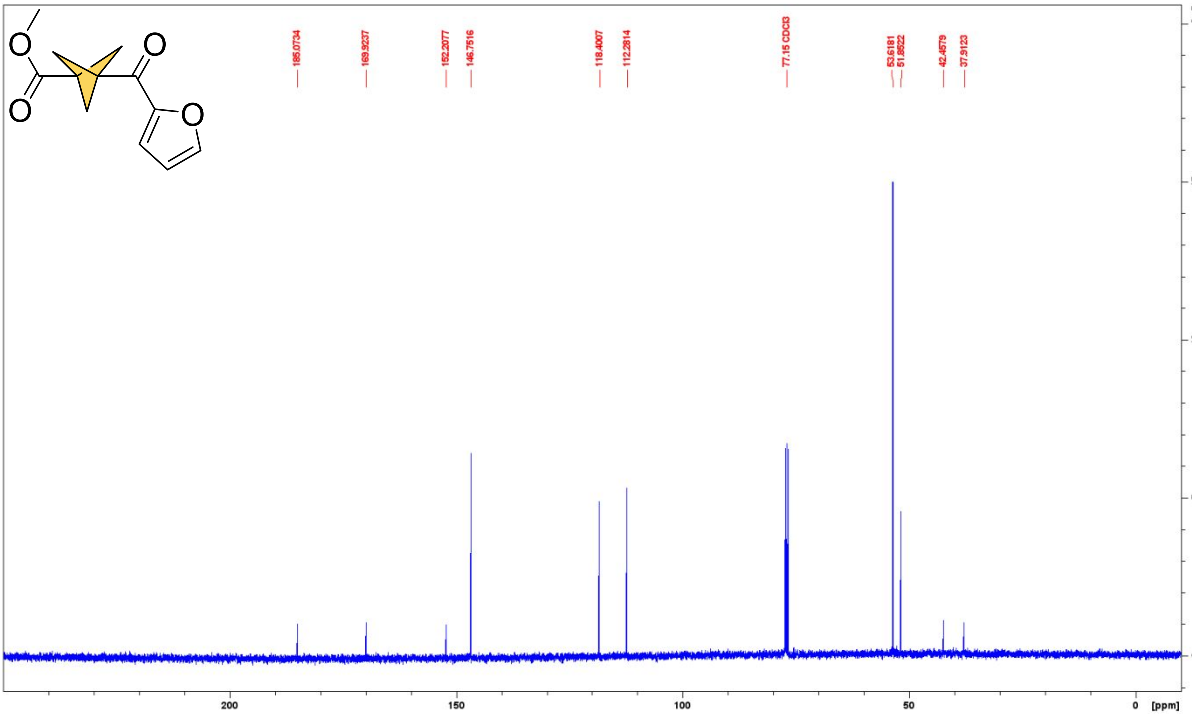

Figure S79: $^{13}C\{^{1}H\}$ NMR ($CDCl_3$, 101 MHz, 298K) spectrum of ***6k****.*

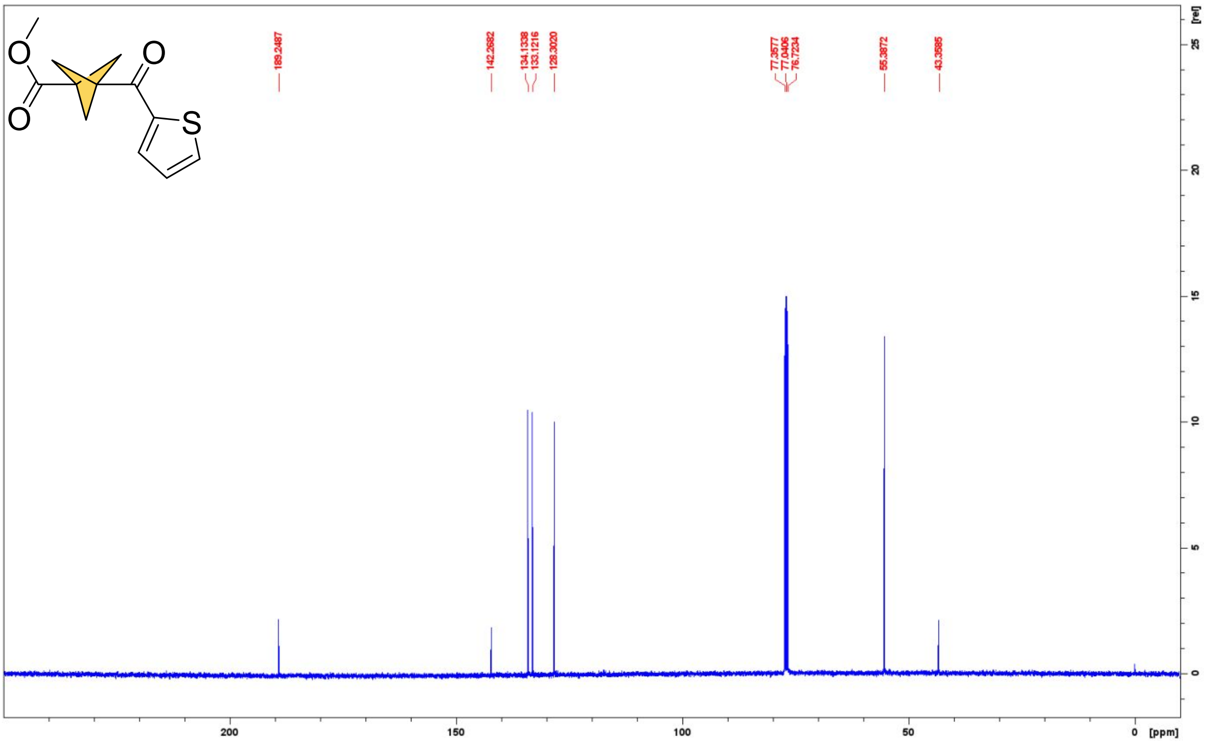

Figure S80: $^{13}C\{^{1}H\}$ NMR ($CDCl_3$, 101 MHz, 298 K) spectrum of ***6l.***

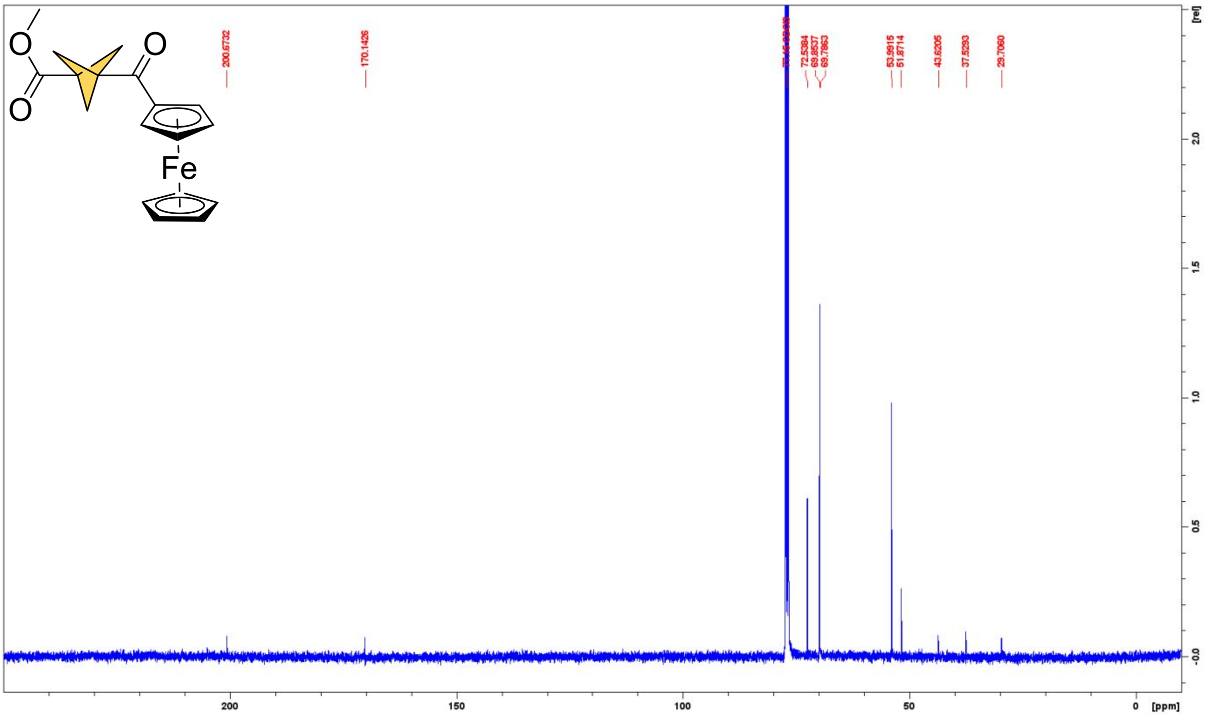

Figure S81: $^{13}C\{^{1}H\}$ NMR ($CDCl_3$, 101 MHz, 298 K) spectrum of ***6m***.

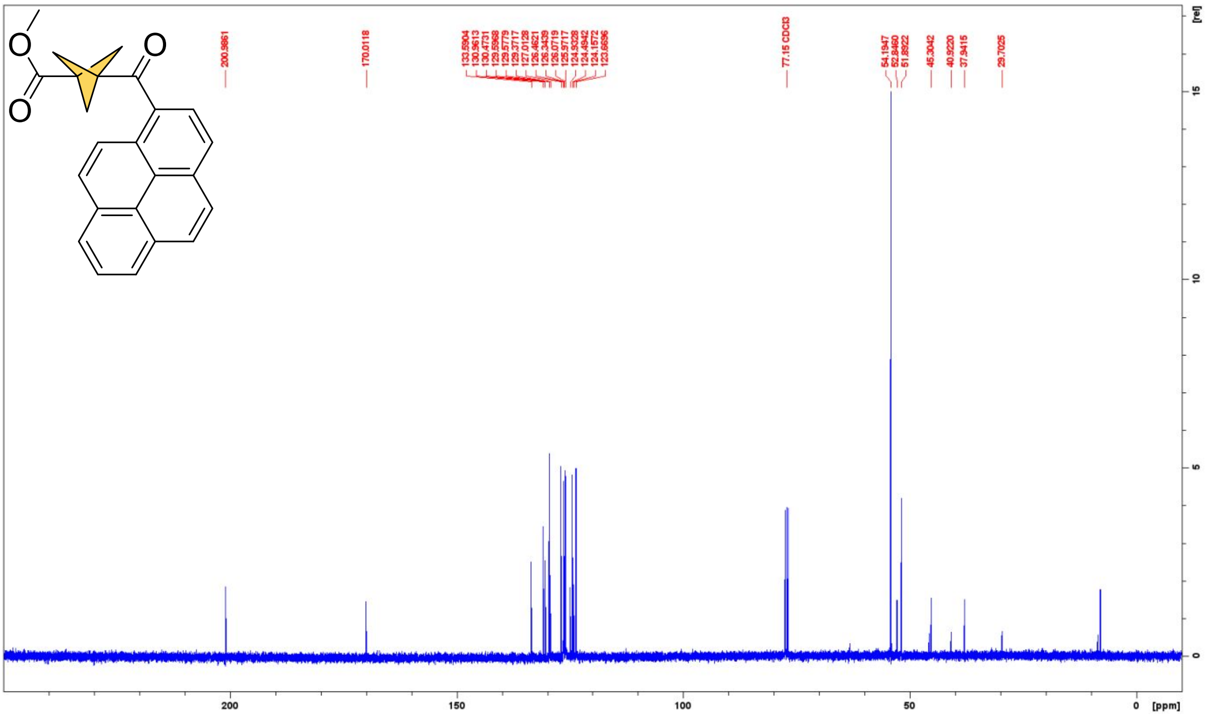

Figure S82: $^{13}C\{^{1}H\}$ NMR ($CDCl_3$, 101 MHz, 298 K) spectrum of ***6n***.

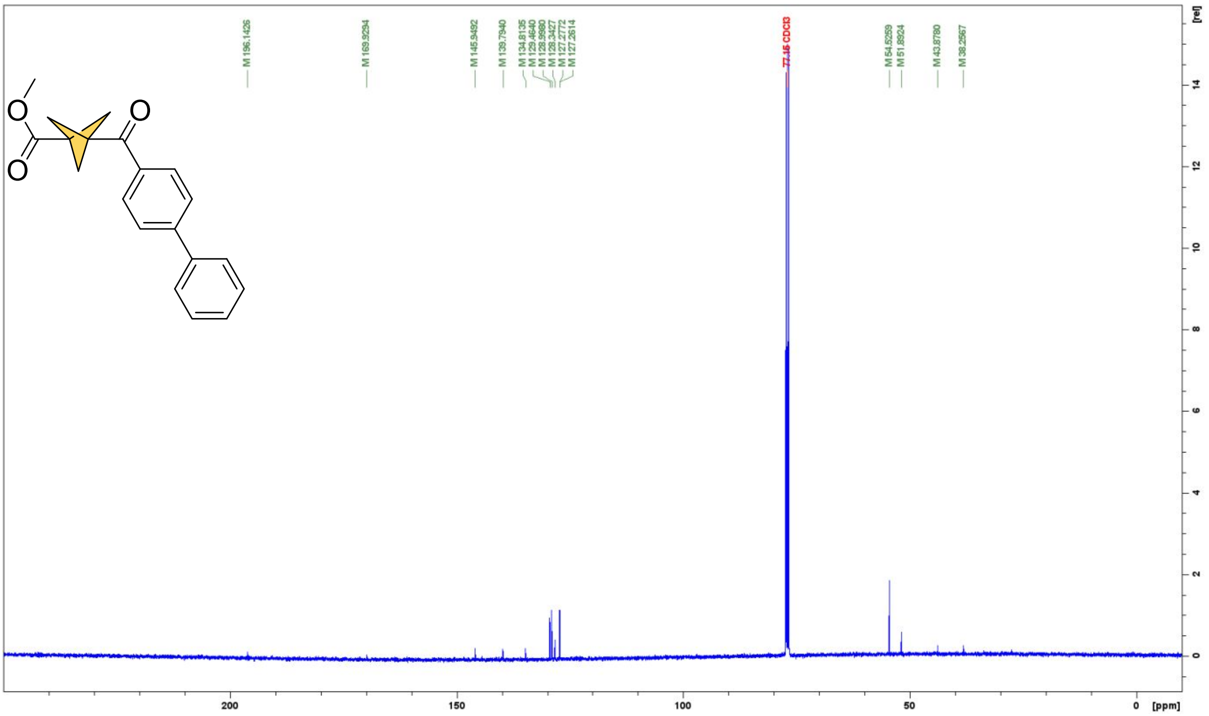

Figure S83: $^{13}C\{^{1}H\}$ NMR ($CDCl_3$, 101 MHz, 298 K) spectrum of ***6o***.

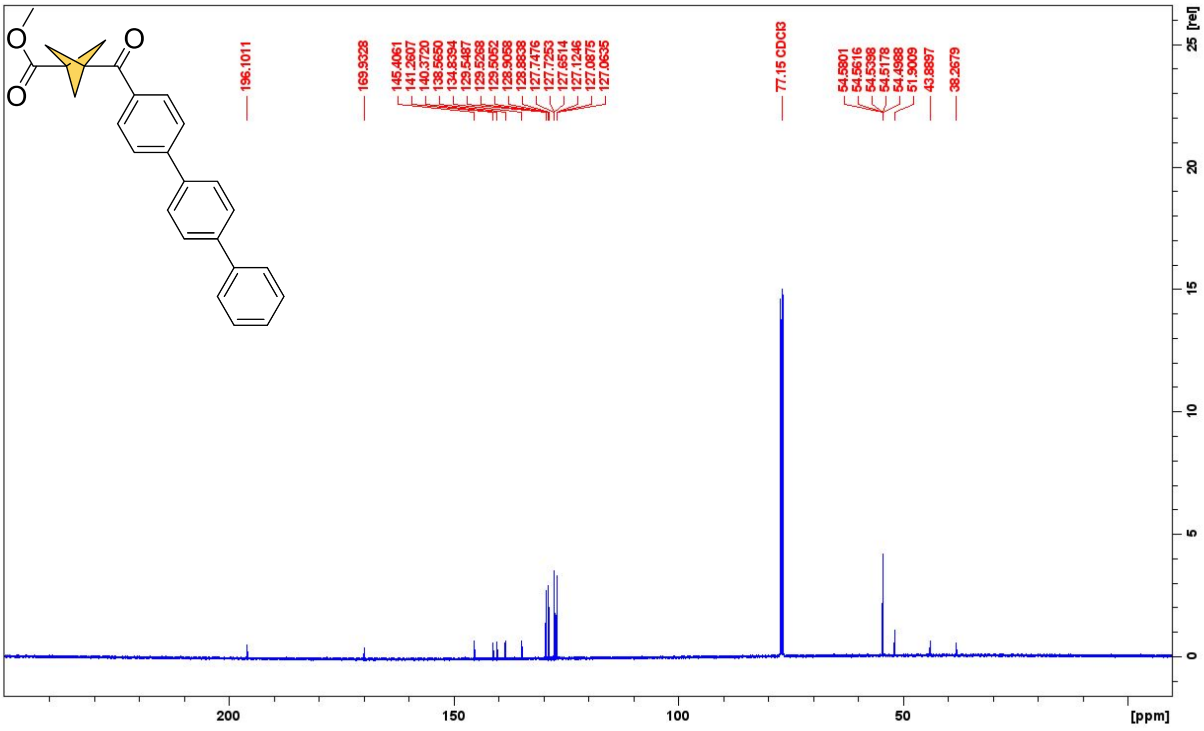

Figure S84: $^{13}C\{^{1}H\}$ NMR ($CDCl_3$, 101 MHz, 298 K) spectrum of ***6p***.

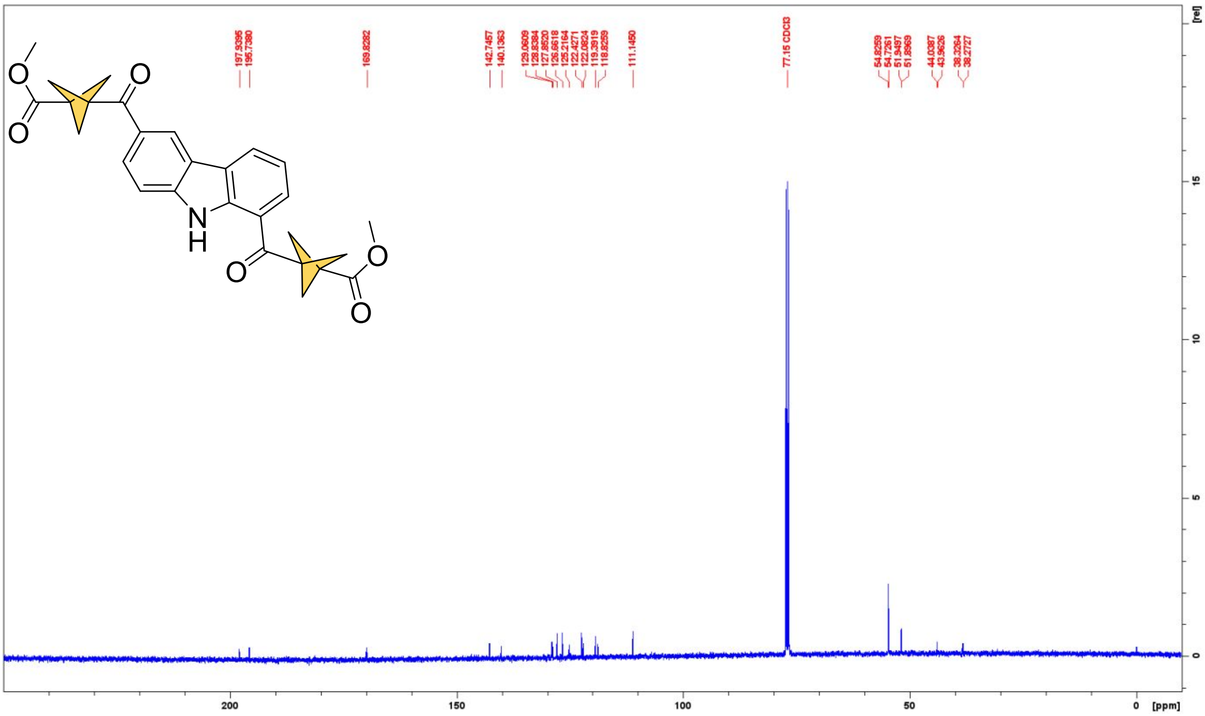

Figure S85: $^{13}C\{^{1}H\}$ NMR ($CDCl_3$, 101 MHz, 298 K) spectrum of ***6q***.

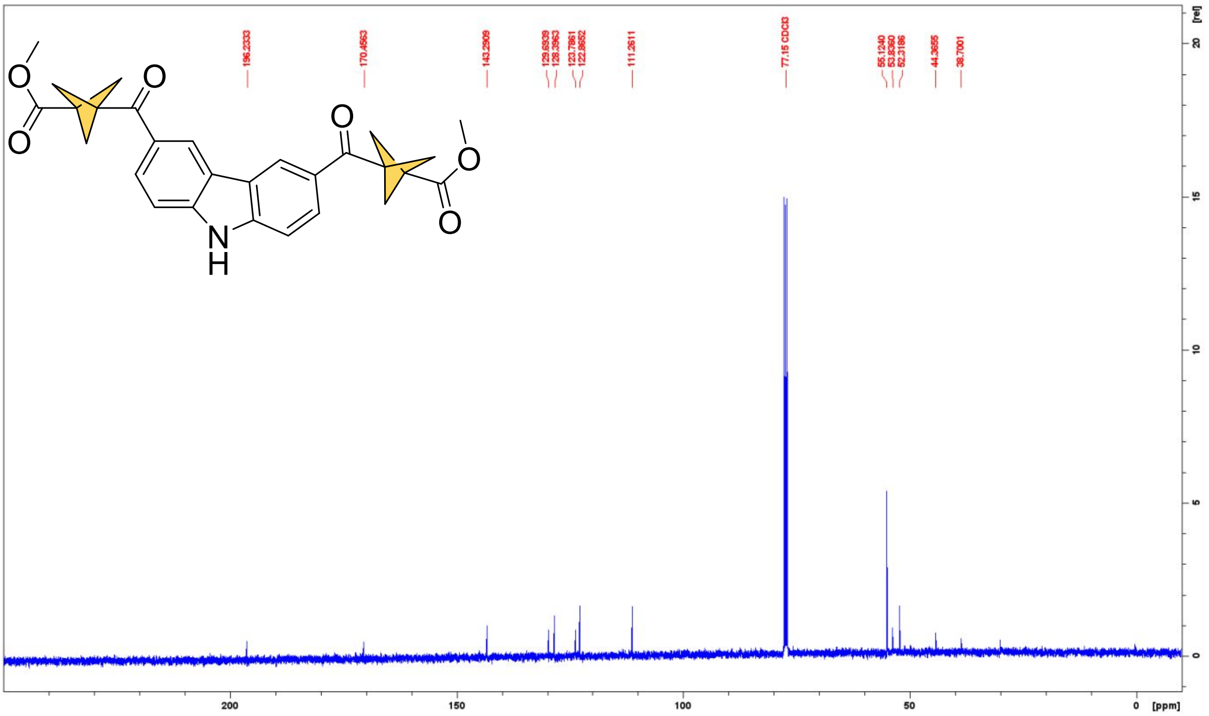

Figure S86: $^{13}C\{^{1}H\}$ NMR ($CDCl_3$, 101 MHz, 298 K) spectrum of **6r**.

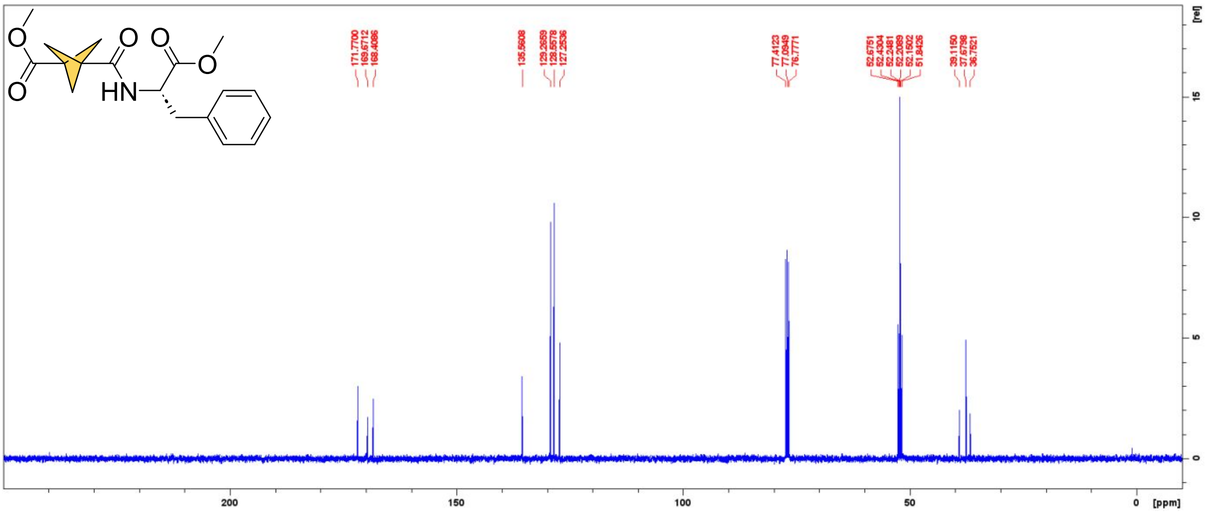

Figure S87: $^{13}C\{^{1}H\}$ NMR ($CDCl_3$, 101 MHz, 298 K) spectrum of **6s**.

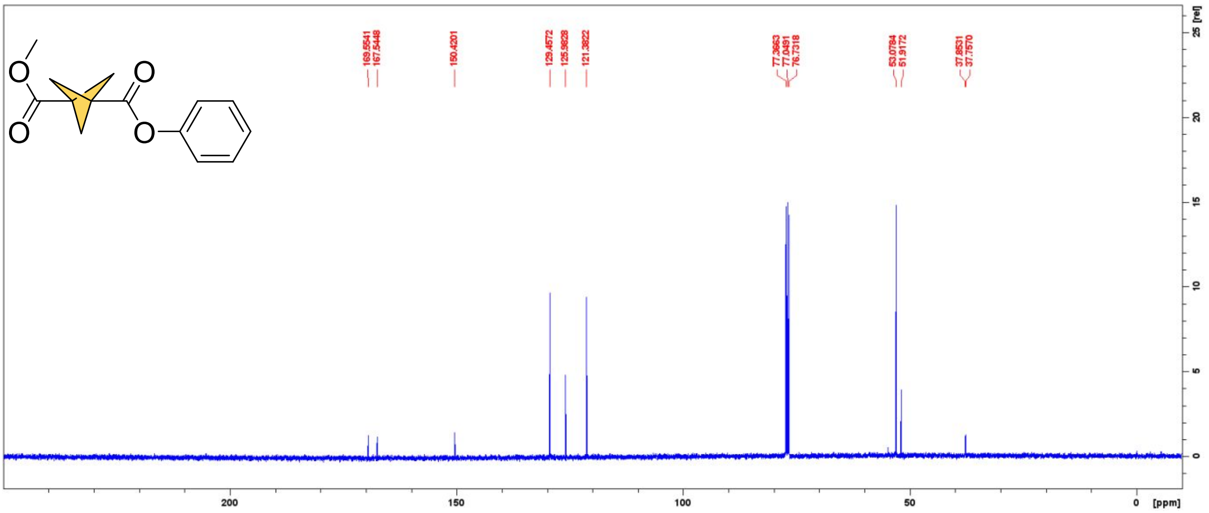

Figure S88: $^{13}C\{^{1}H\}$ NMR ($CDCl_3$, 101 MHz, 298 K) spectrum of ***6t****.*

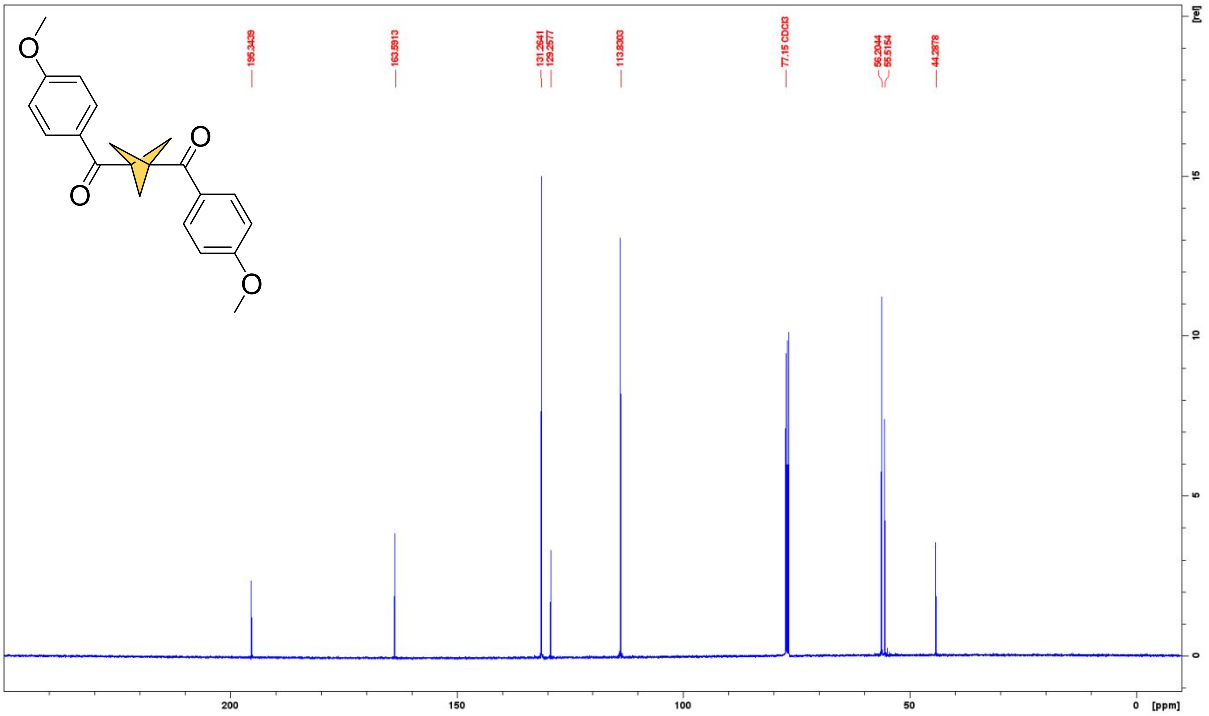

Figure S89: $^{13}C\{^{1}H\}$ NMR ($CDCl_3$, 101 MHz, 298 K) spectrum of ***7a****.*

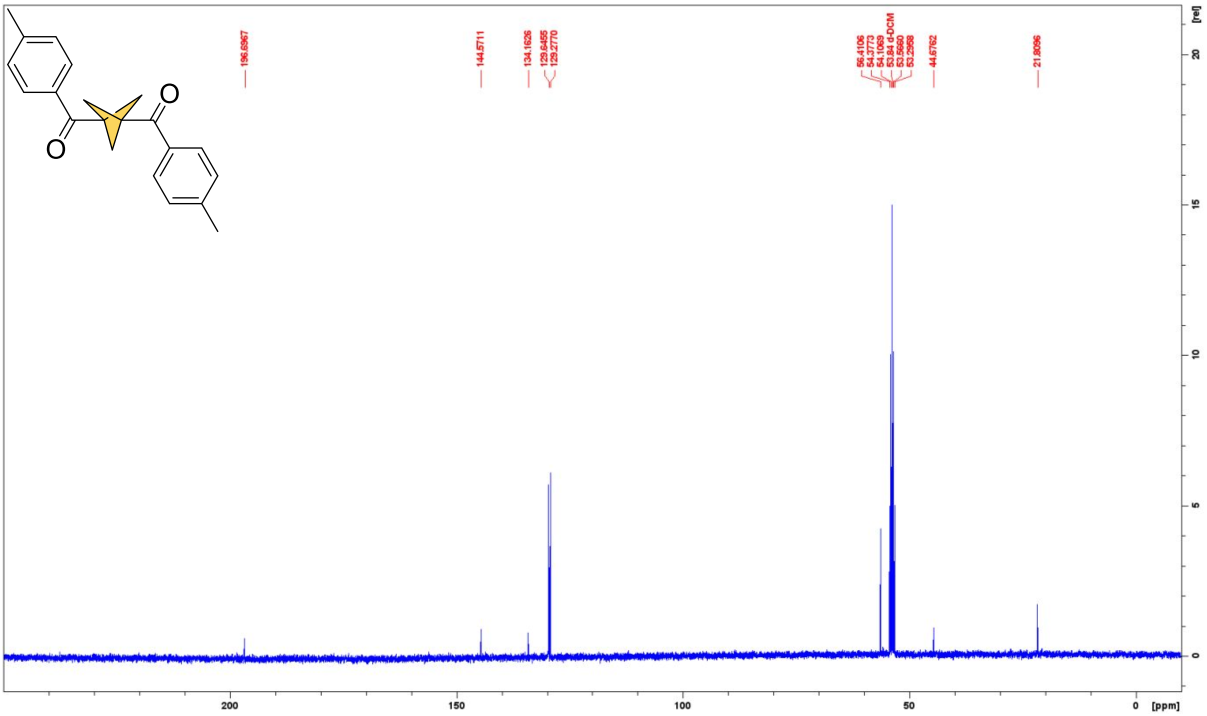

*Figure S90: 13C{1H} NMR (CD_2Cl_2, 101 MHz, 298 K) spectrum of **7b**.*

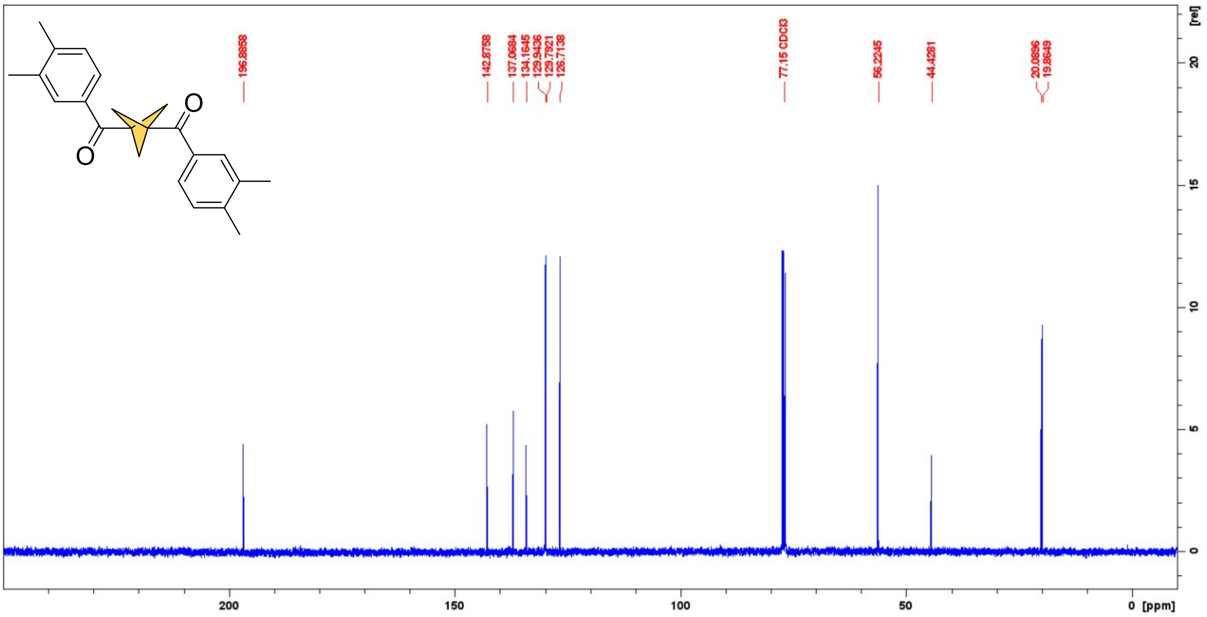

*Figure S91: $^{13}C\{^{1}H\}$ NMR ($CDCl_3$, 101 MHz, 298 K) spectrum of **7c**.*

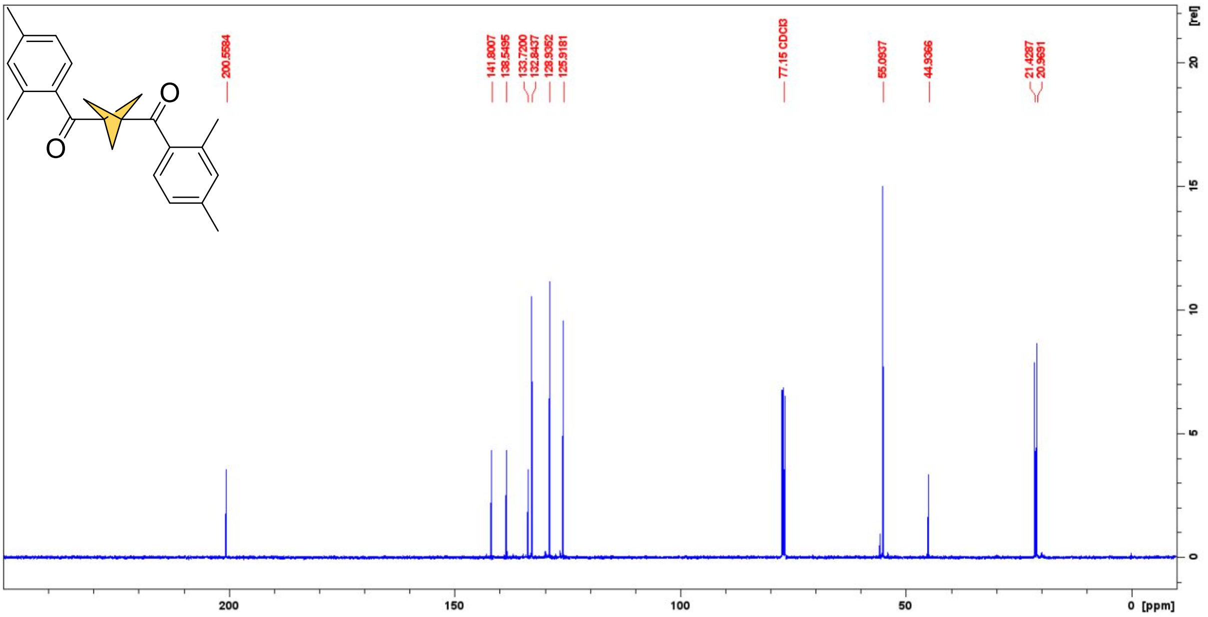

Figure S92: $^{13}C\{^{1}H\}$ NMR ($CDCl_3$, 101 MHz, 298 K) spectrum of ***7d.***

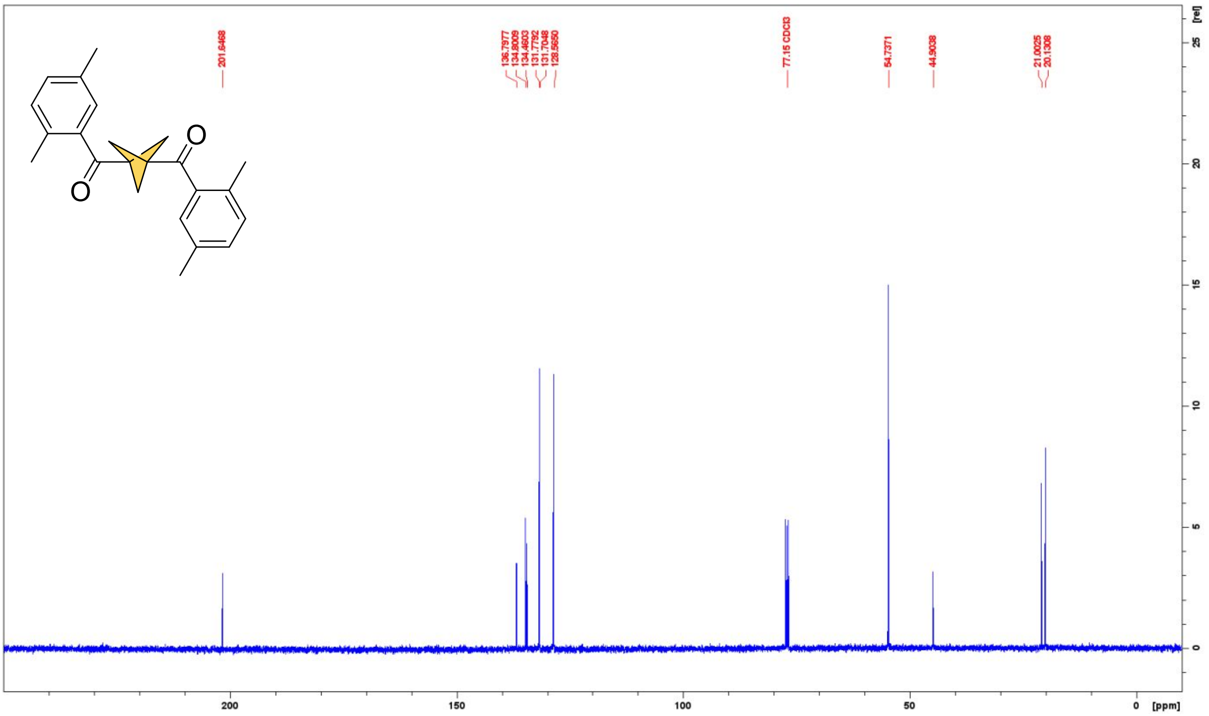

Figure S93: $^{13}C\{^{1}H\}$ NMR ($CDCl_3$, 101 MHz, 298 K) spectrum of ***7e.***

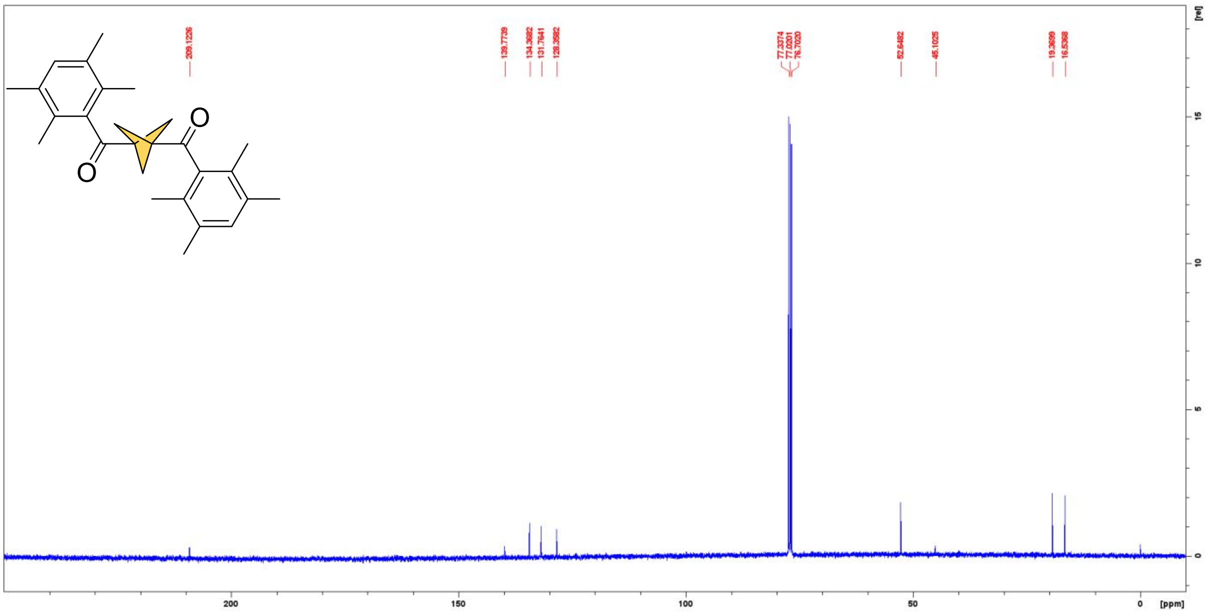

*Figure S94: $^{13}C\{^{1}H\}$ NMR ($CDCl_3$, 101 MHz, 298 K) spectrum of **7f**.*

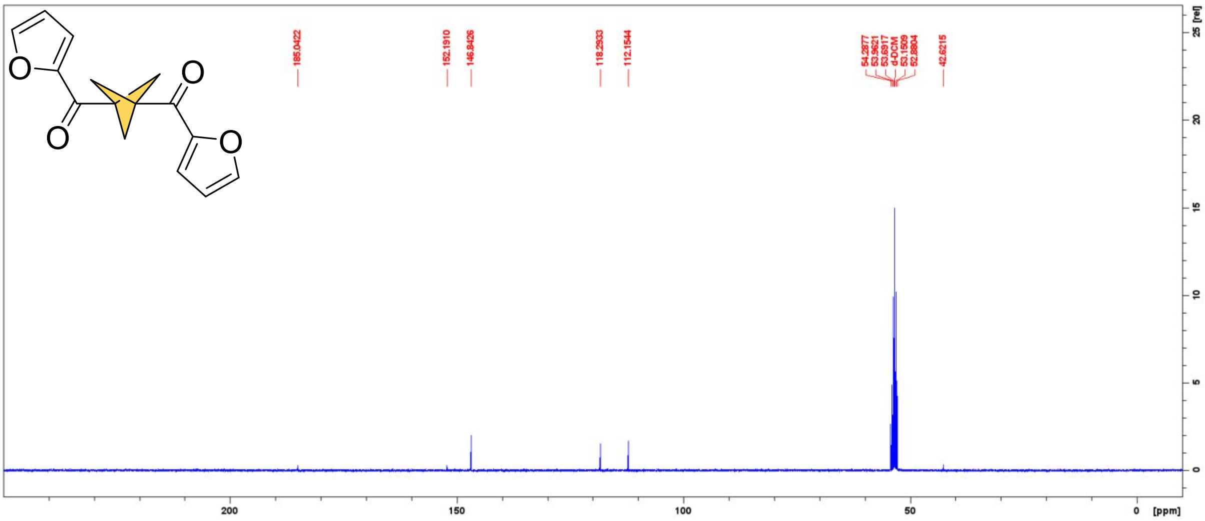

*Figure S95: $^{13}C\{^{1}H\}$ NMR (CD_2Cl_2, 101 MHz, 298 K) spectrum of **7g**.*

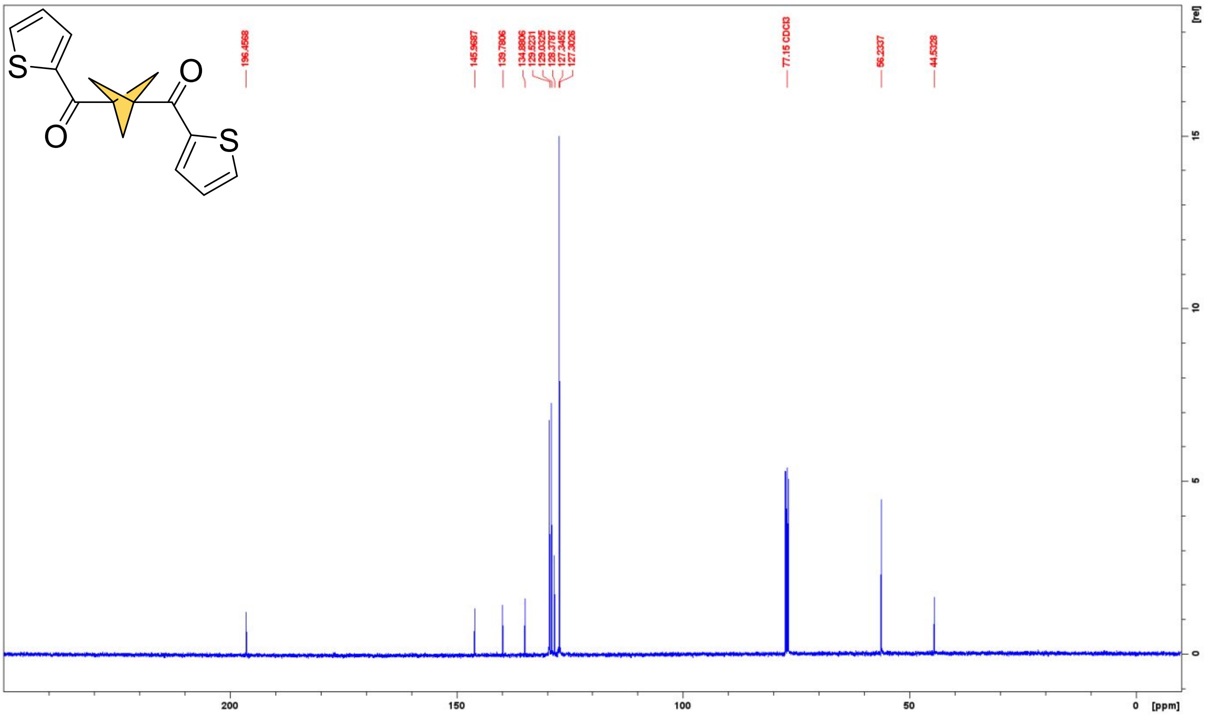

*Figure S96: $^{13}C\{^{1}H\}$ NMR ($CDCl_3$, 101 MHz, 298K) spectrum of **7h**.*

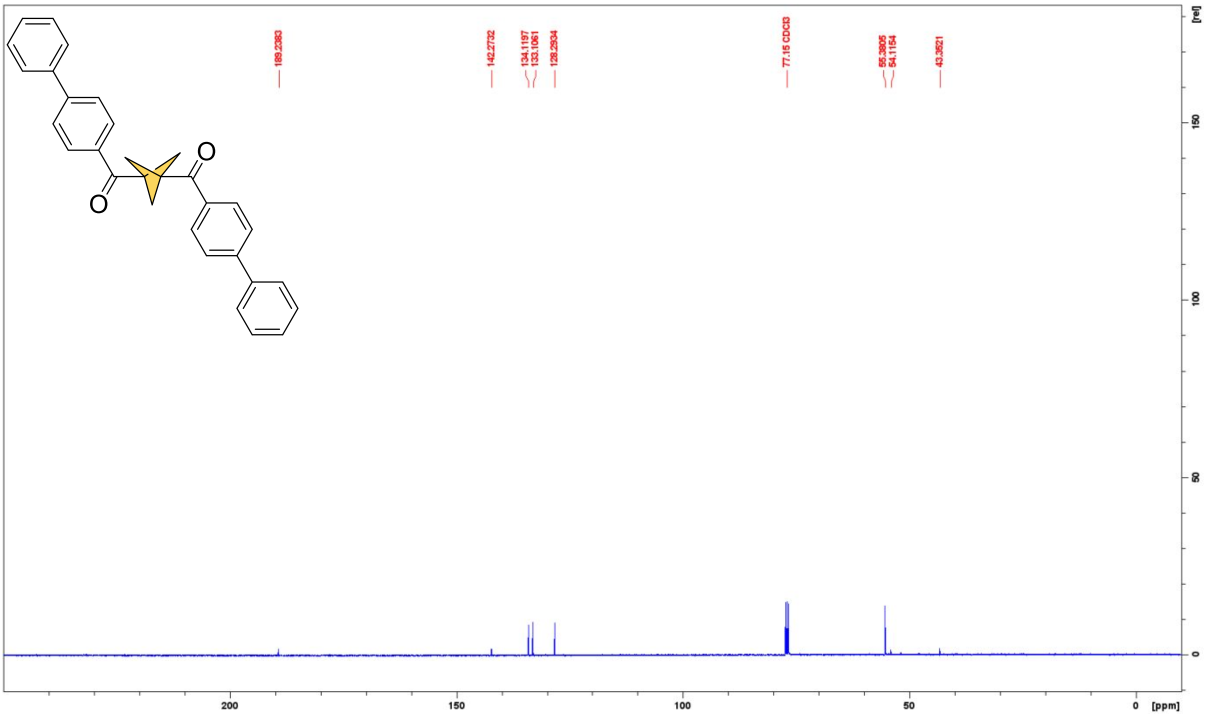

*Figure S97: $^{13}C\{^{1}H\}$ NMR ($CDCl_3$, 101 MHz, 298 K) spectrum of **7i**.*

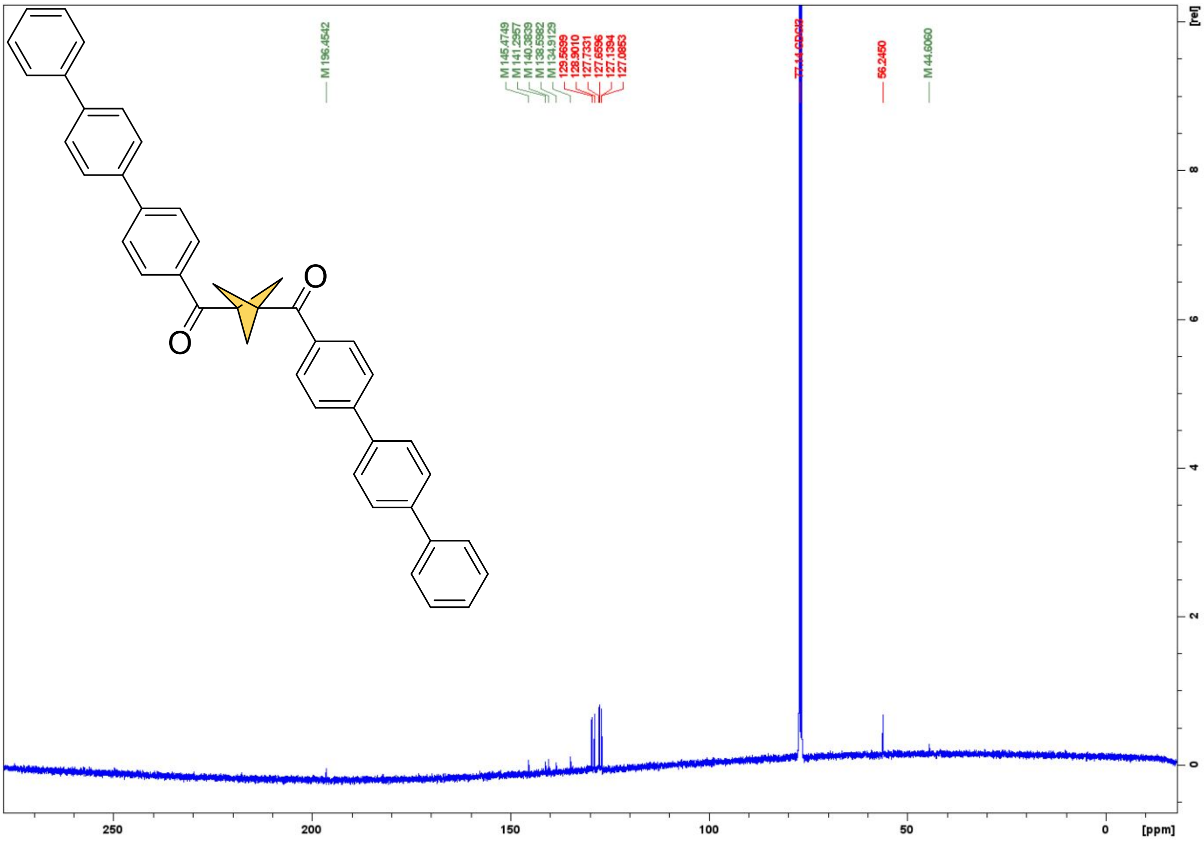

Figure S98: ${}^{13}C\{{}^{1}H\}$ NMR ($CDCl_3$, 101 MHz, 298 K) spectrum of ***7j****.*

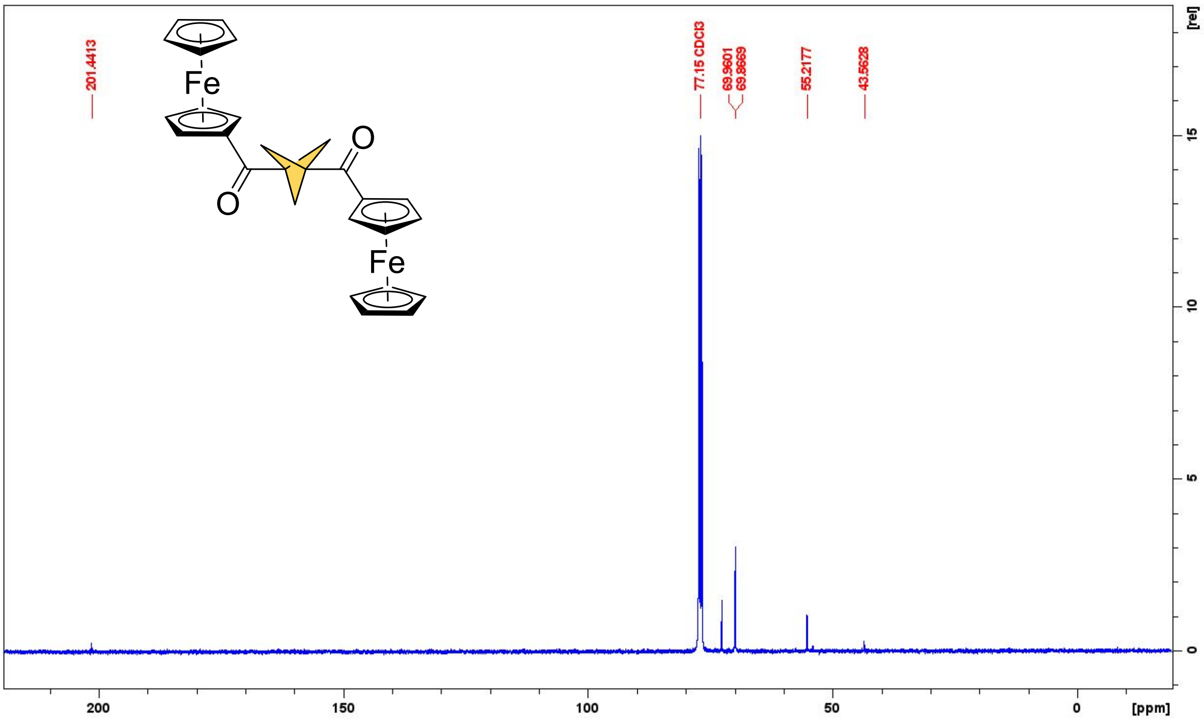

Figure S99: ${}^{13}C\{{}^{1}H\}$ NMR ($CDCl_3$, 101 MHz, 298 K) spectrum of ***7k.***

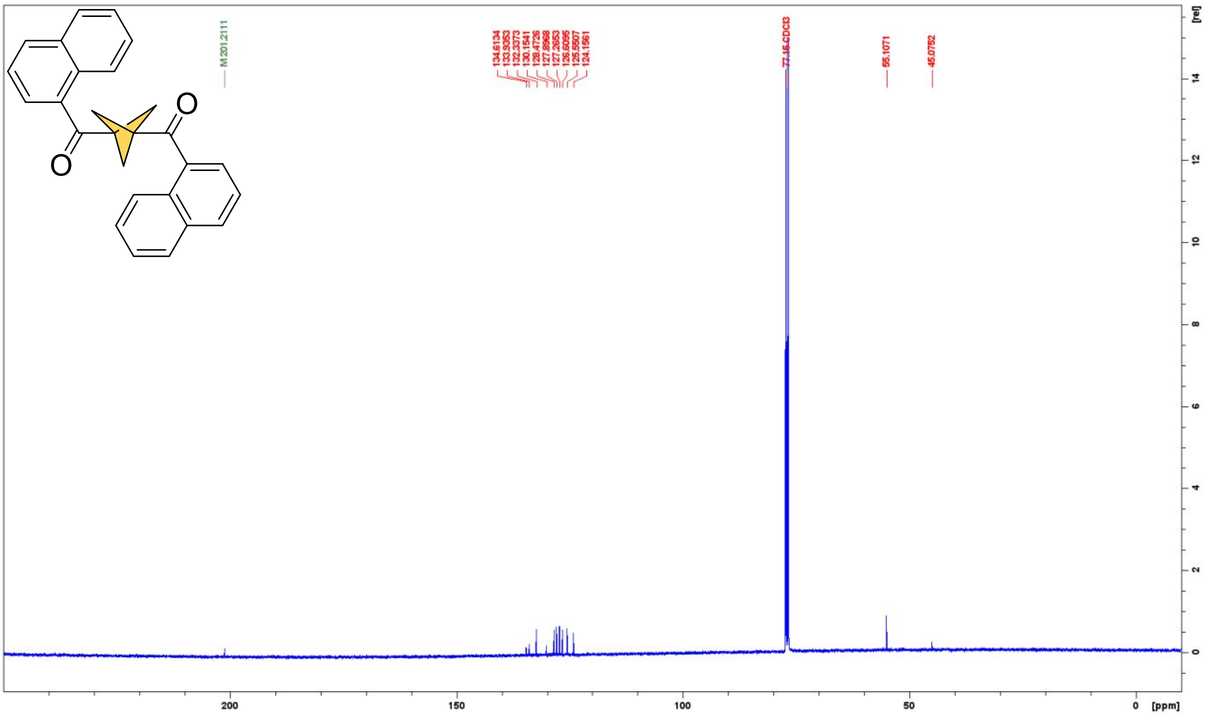

Figure S100: $^{13}C\{^{1}H\}$ NMR ($CDCl_3$, 101 MHz, 298K) spectrum of ***7l***.

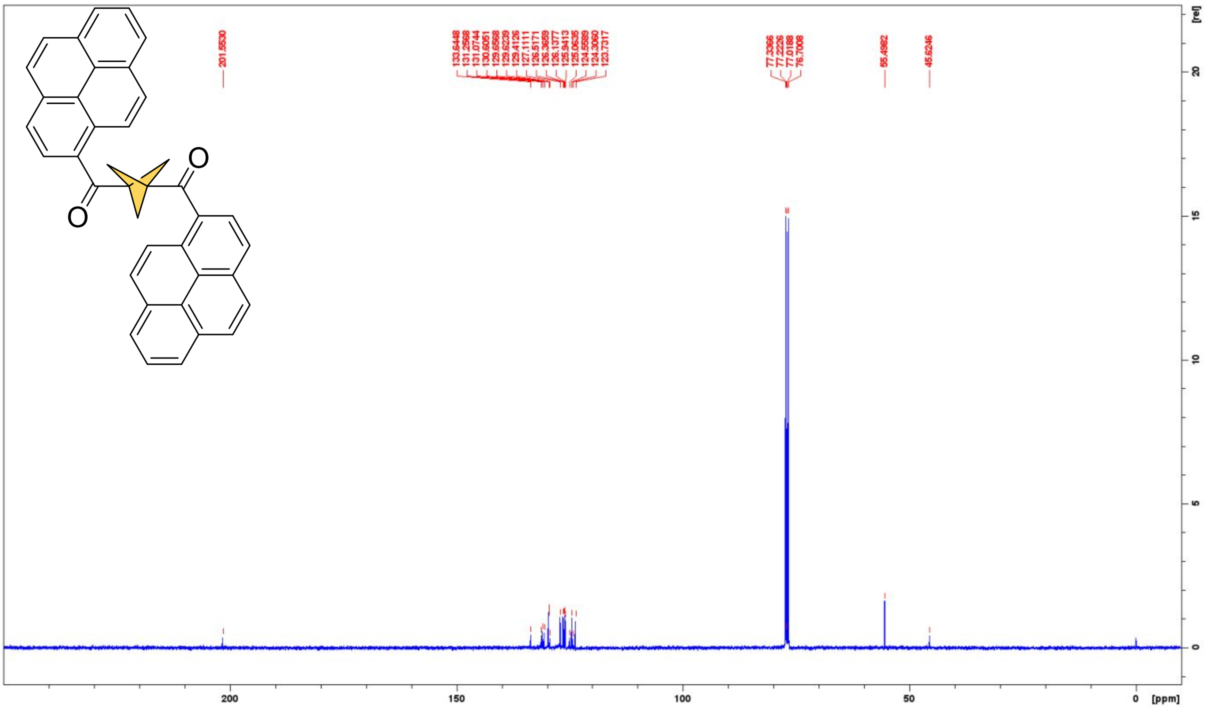

Figure S101: $^{13}C\{^{1}H\}$ NMR ($CDCl_3$, 101 MHz, 298 K) spectrum of ***7m***.

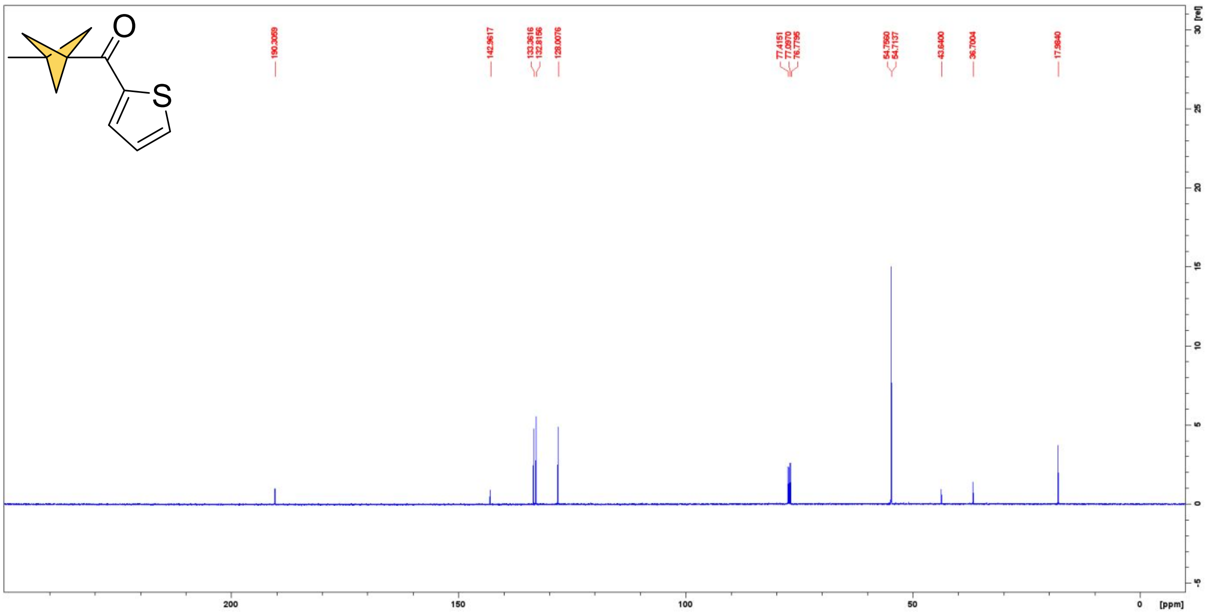

Figure S102: $^{13}C\{^{1}H\}$ NMR ($CDCl_3$, 101 MHz, 298 K) spectrum of ***9b****.*

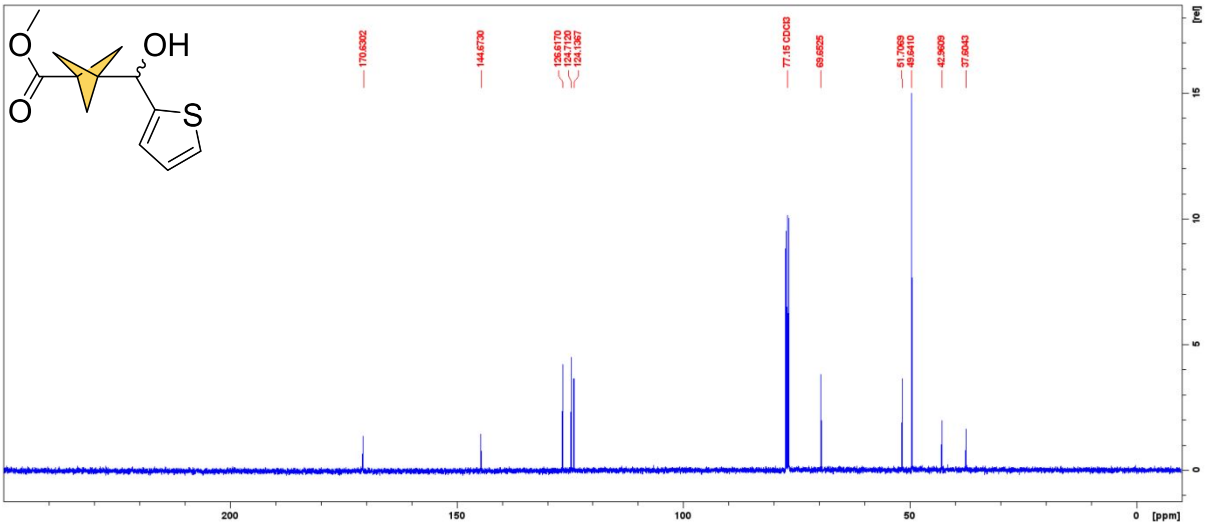

Figure S103: $^{13}C\{^{1}H\}$ NMR ($CDCl_3$, 101 MHz, 298 K) spectrum of ***10****.*

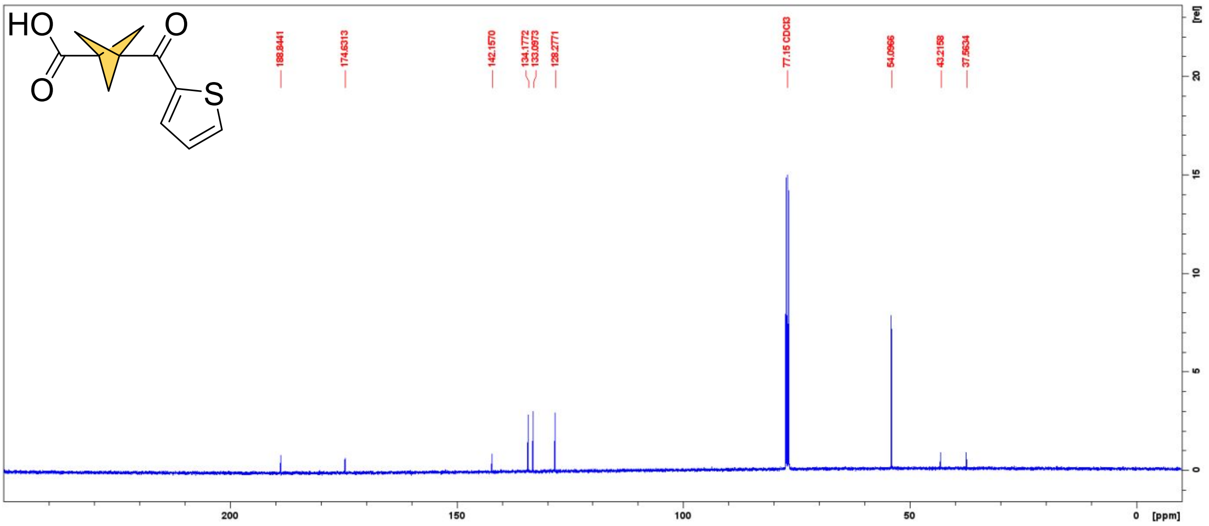

Figure S104: $^{13}C\{^{1}H\}$ NMR ($CDCl_3$, 101 MHz, 298 K) spectrum of ***11.***

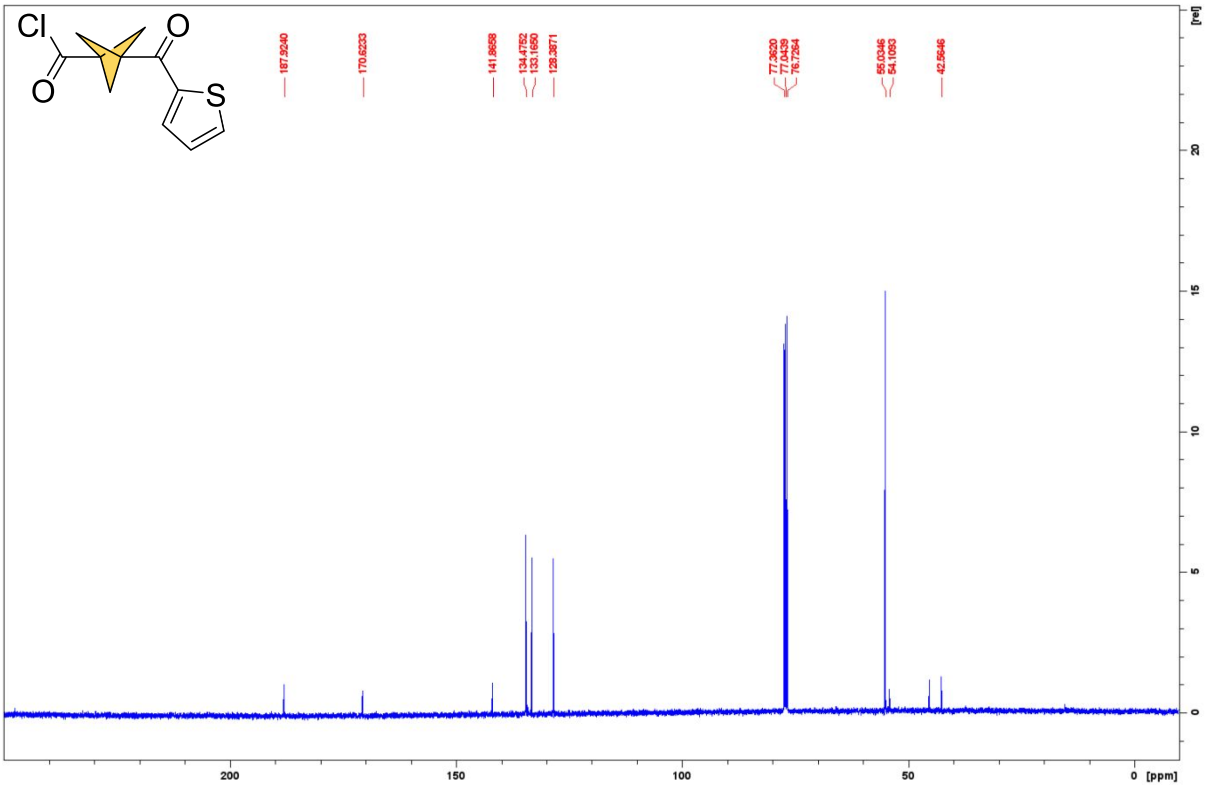

*Figure S105: $^{13}C\{^{1}H\}$ NMR ($CDCl_3$, 101 MHz, 298 K) spectrum of **12**.*

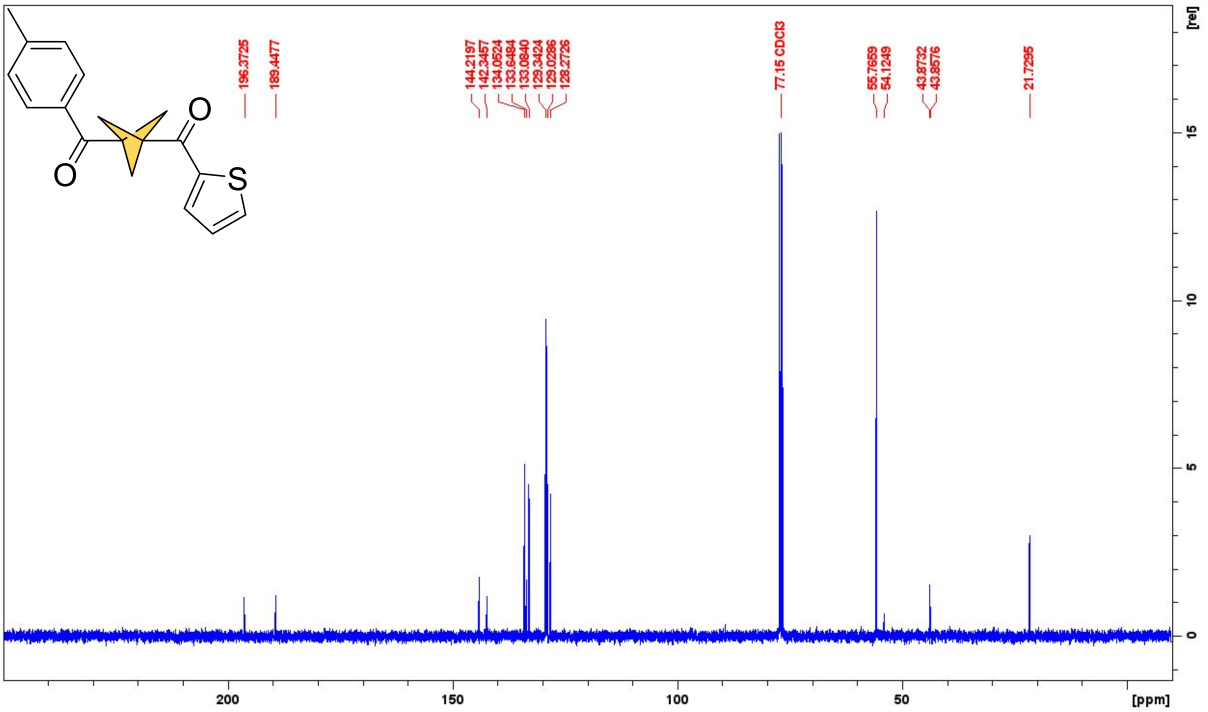

*Figure S106: $^{13}C\{^{1}H\}$ NMR ($CDCl_3$, 101 MHz, 298 K) spectrum of **13.***

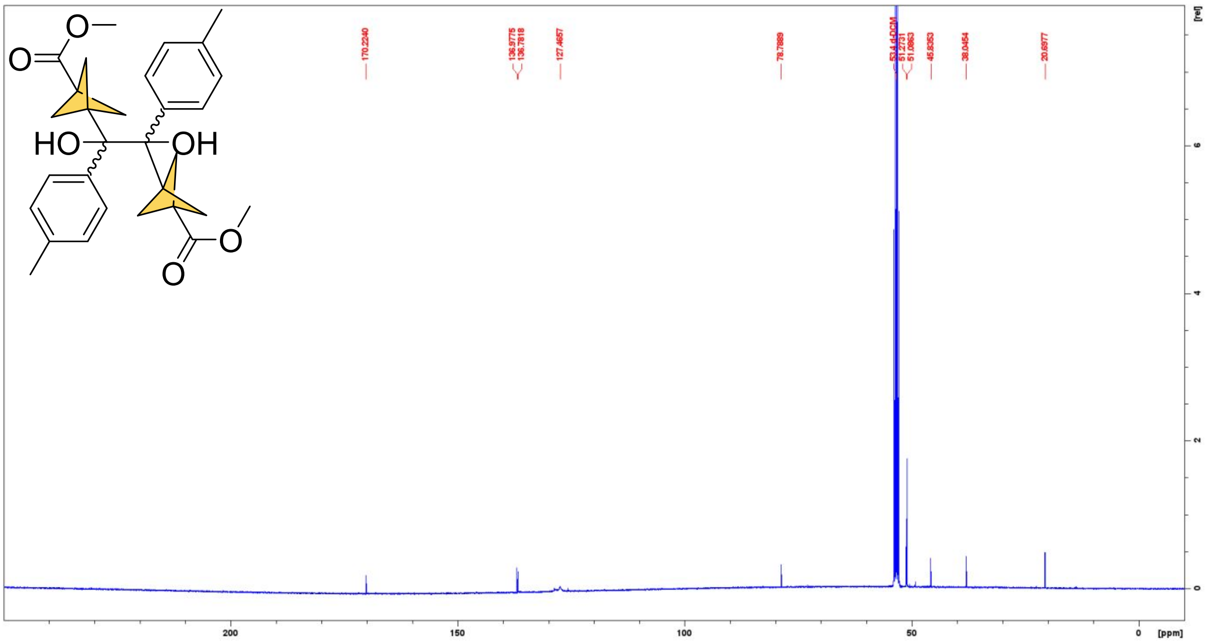

Figure S107: $^{13}C\{^{1}H\}$ NMR (CD_2Cl_2, 101 MHz, 298 K) spectrum of ***14a***.

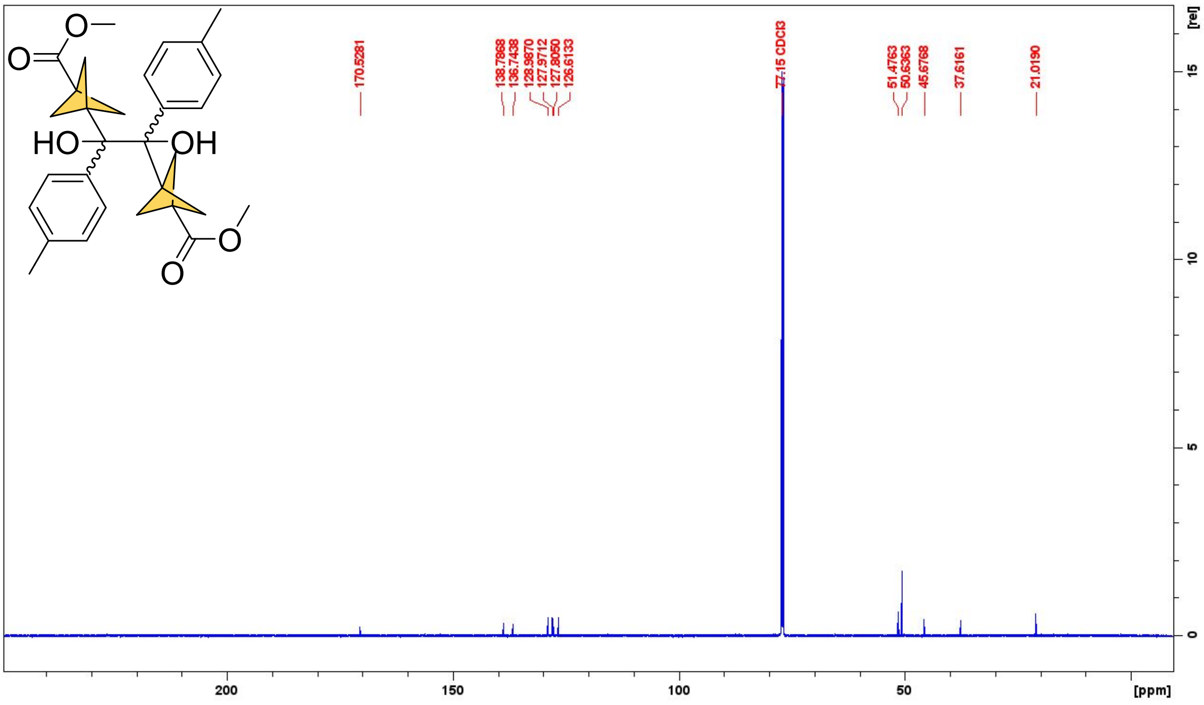

Figure S108: $^{13}C\{^{1}H\}$ NMR ($CDCl_3$, 101 MHz, 298 K) spectrum of ***14b***.

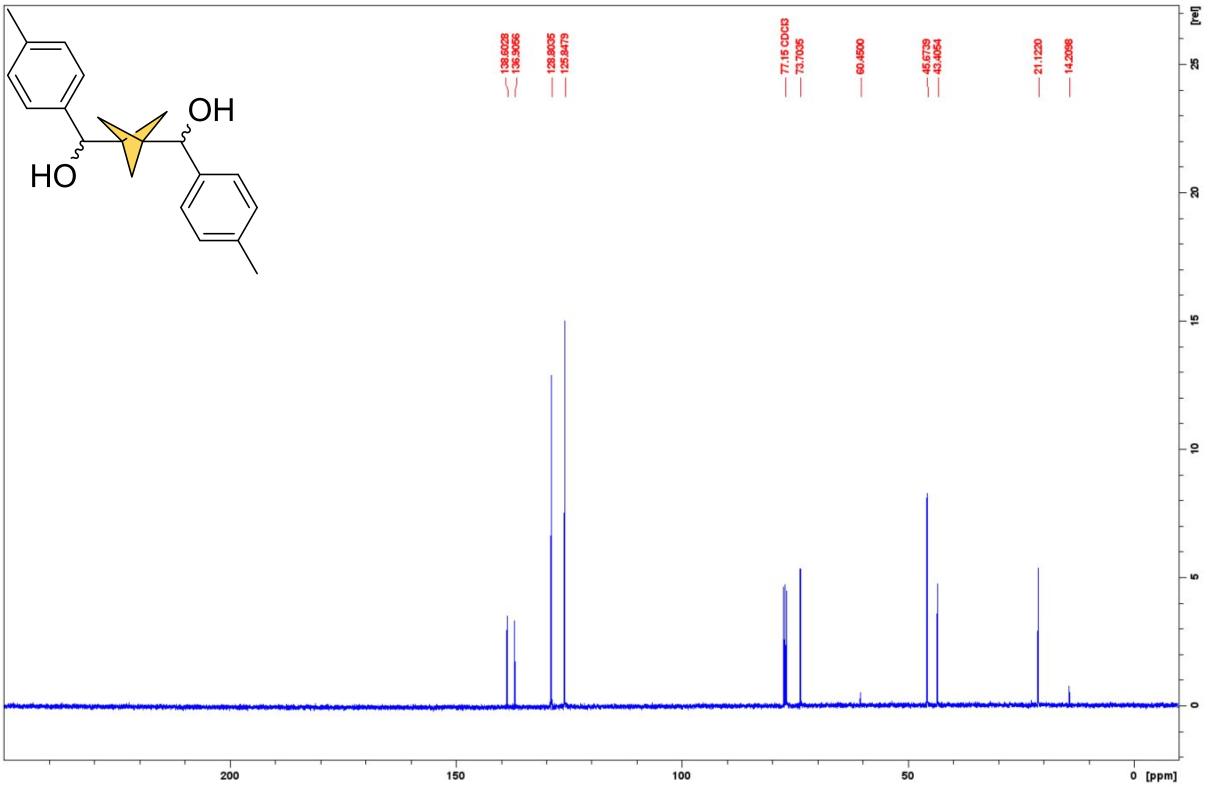

*Figure S109: $^{13}C\{^{1}H\}$ NMR ($CDCl_3$, 101 MHz, 298 K) spectrum of **15**.*

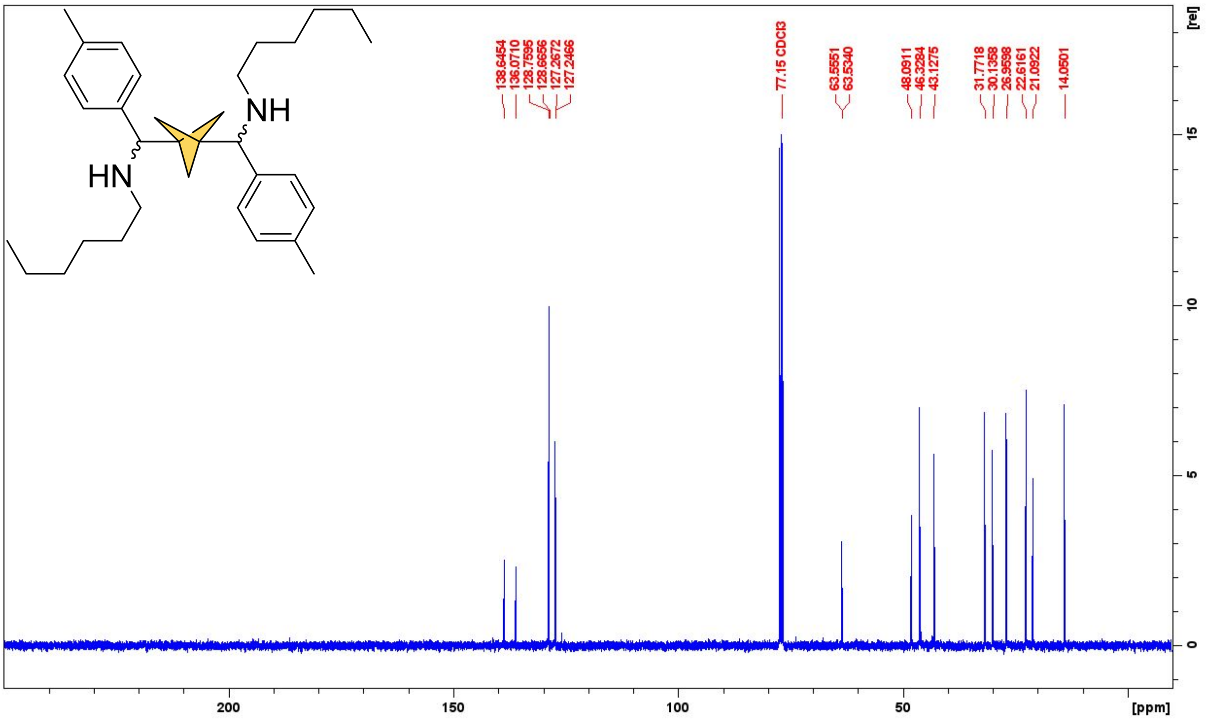

*Figure S110: $^{13}C\{^{1}H\}$ NMR ($CDCl_3$, 101 MHz, 298K) spectrum of **16**.*

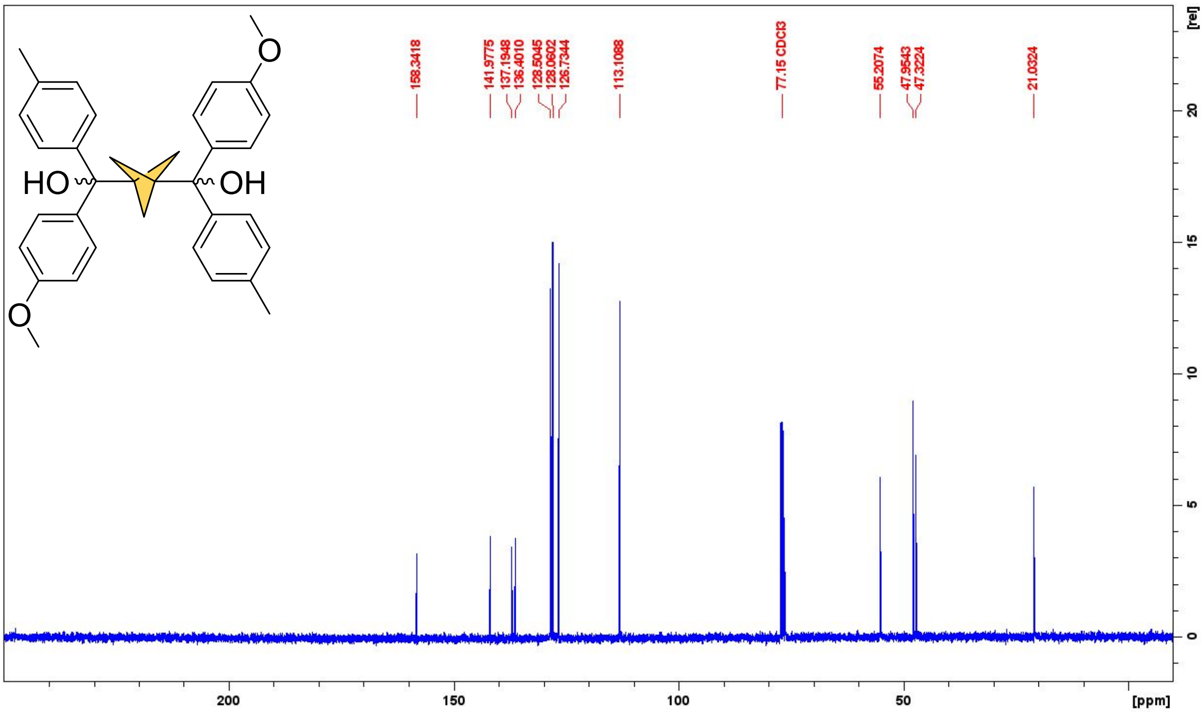

Figure S111: $^{13}C\{^{1}H\}$ NMR ($CDCl_3$, 101 MHz, 298 K) spectrum of ***17***.

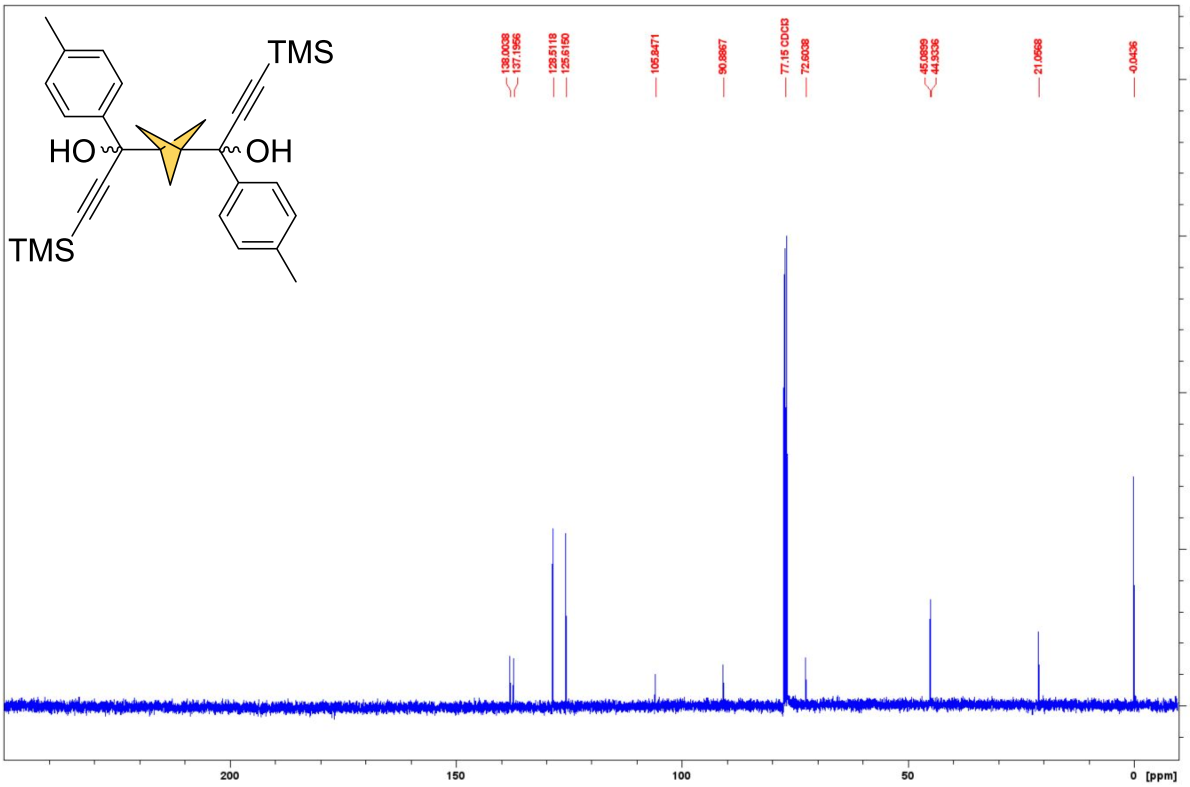

Figure S112: $^{13}C\{^{1}H\}$ NMR ($CDCl_3$, 101 MHz, 298 K) spectrum of ***18***.

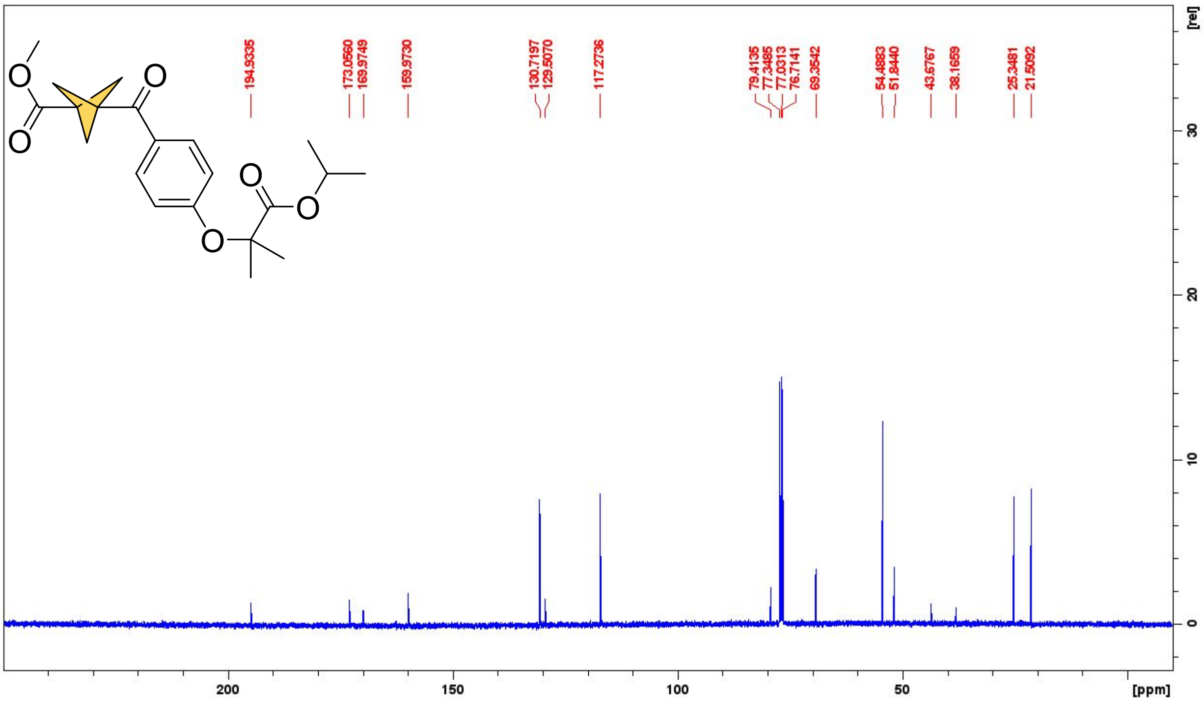

*Figure S113: $^{13}C\{^{1}H\}$ NMR ($CDCl_3$, 101 MHz, 298 K) spectrum of **20a**.*

2D NMR spectra

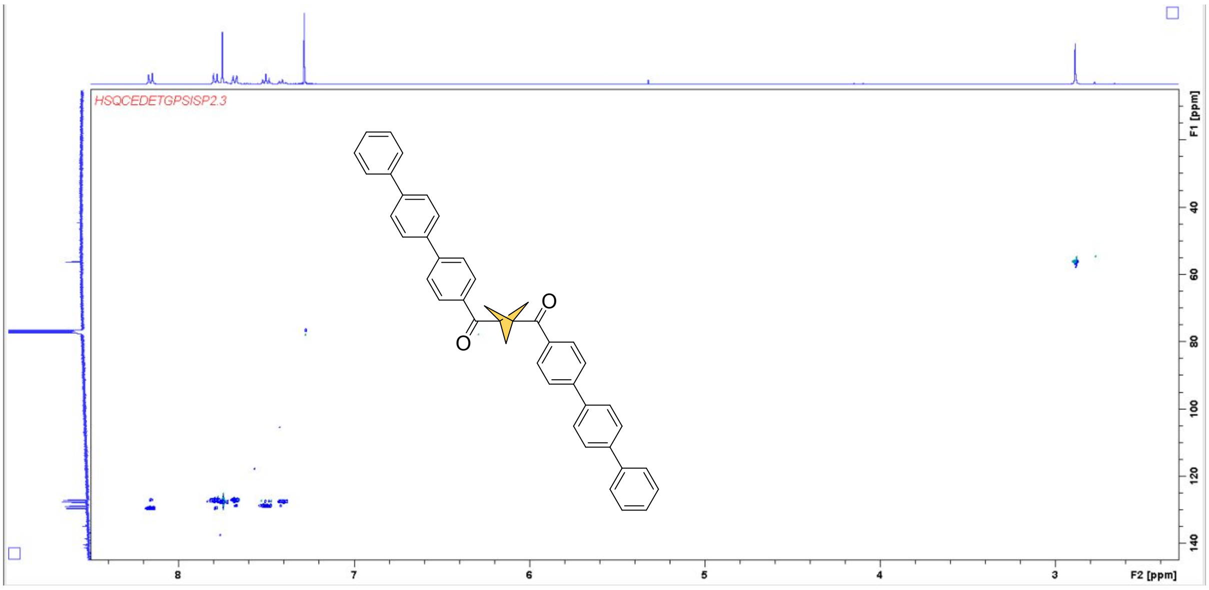

*Figure S114: HSQC ($CDCl_3$, 101 MHz, 298 K) spectrum of **7j**.*

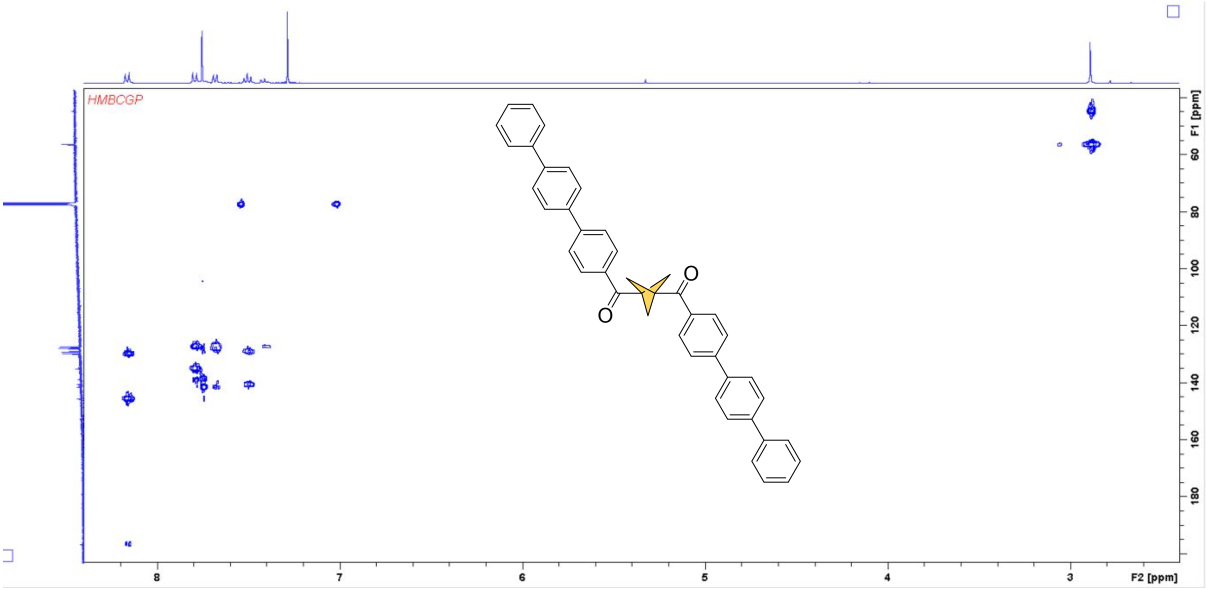

Figure S115: HMBC ($CDCl_3$, 101 MHz, 298 K) spectrum of ***7j***.

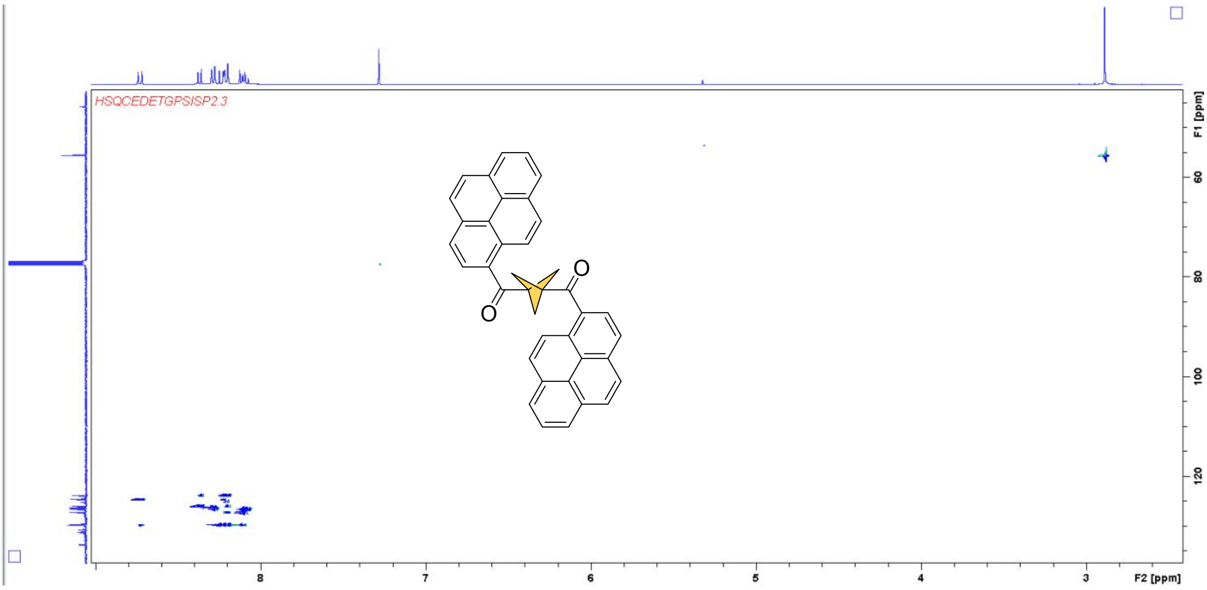

Figure S116: HSQC ($CDCl_3$, 101 MHz, 298 K) spectrum of ***7m***.

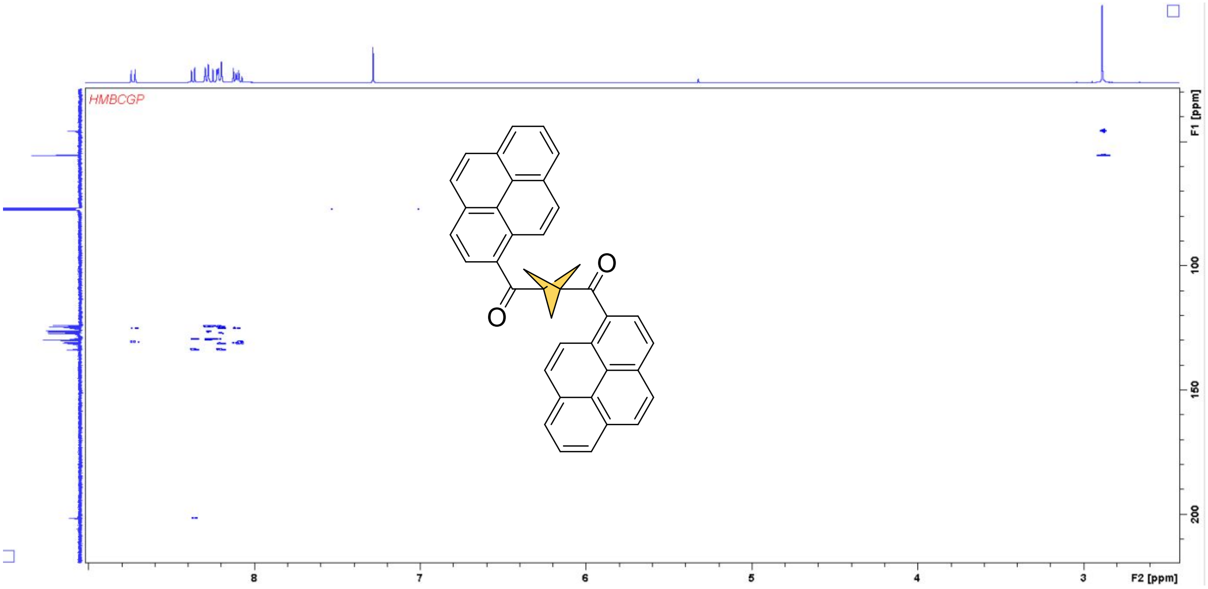

Figure S117: HMBC ($CDCl_3$, 101 MHz, 298 K) spectrum of ***7m***.

Mass spectra

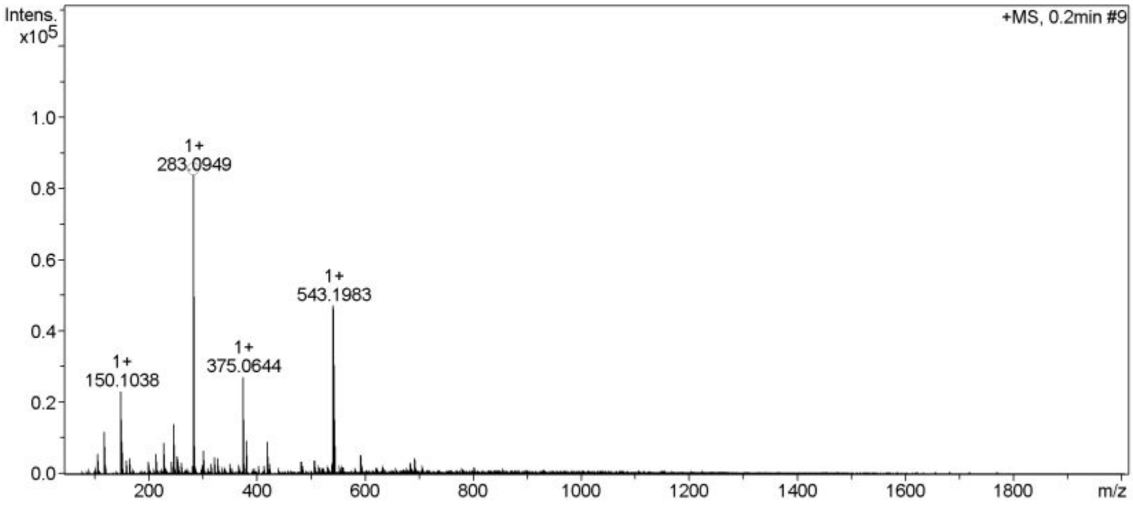

Meas. m/z	#	Ion Formula	m/z	err [mDa]	err [ppm]	rdb	N-Rule	e⁻ Conf	mSigma
283.094874	1	C15H16NaO4	283.094080	-0.8	-2.8	7.5	ok	even	5.3

Figure S118: HRMS (m/z)-ESI spectrum of ***6a***.

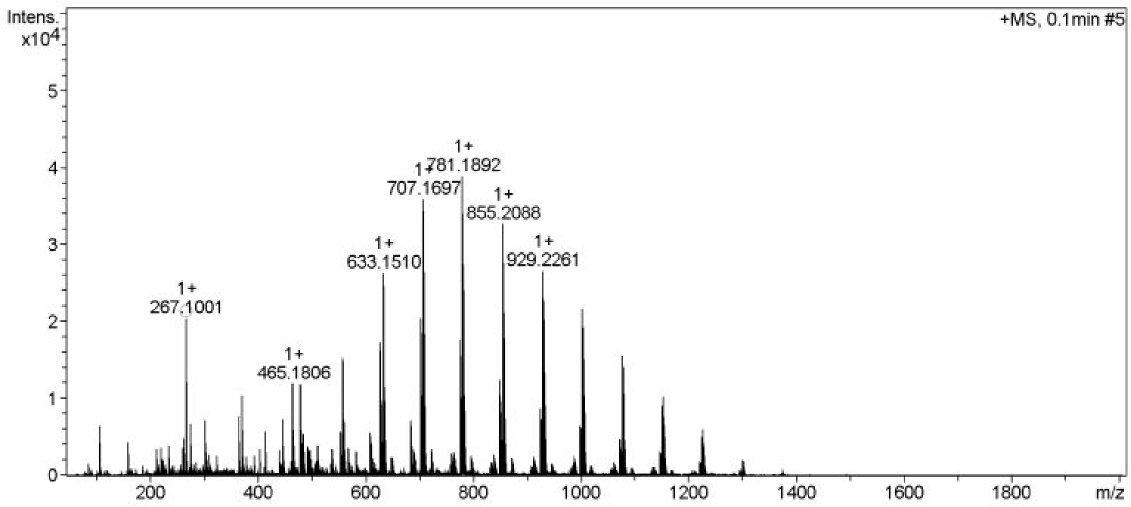

Meas. m/z	#	Ion Formula	m/z	err [mDa]	err [ppm]	rdb	N-Rule	e⁻ Conf	mSigma
267.100143	1	C15H16NaO3	267.099165	-1.0	-3.7	7.5	ok	even	4.6

*Figure S119: HRMS (m/z)- ESI spectrum of **6b**.*

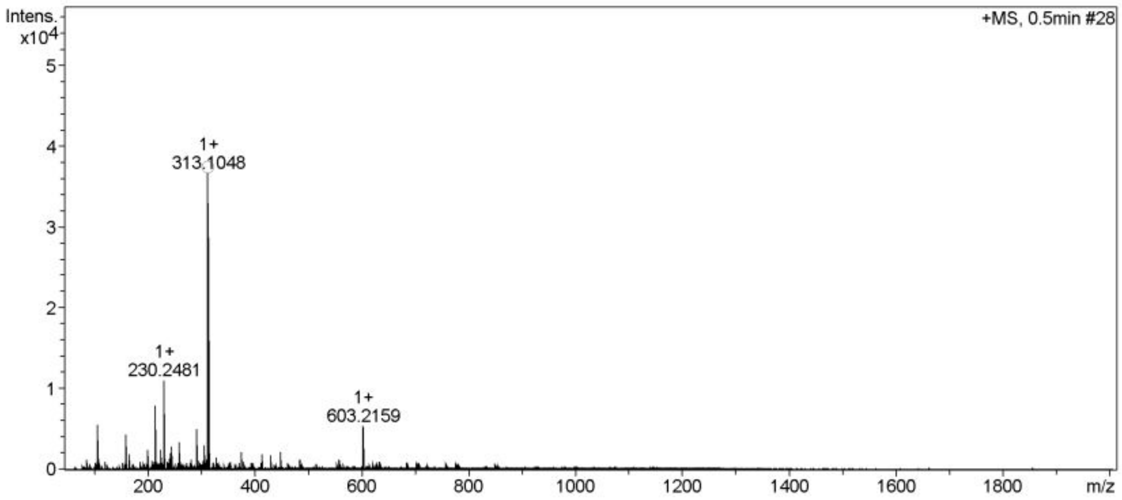

Meas. m/z	#	Ion Formula	m/z	err [mDa]	err [ppm]	rdb	N-Rule	e⁻ Conf	mSigma
313.104774	1	C16H18NaO5	313.104644	0.1	0.4	7.5	ok	even	3.0

*Figure S120: HRMS (m/z)- ESI spectrum of **6c**.*

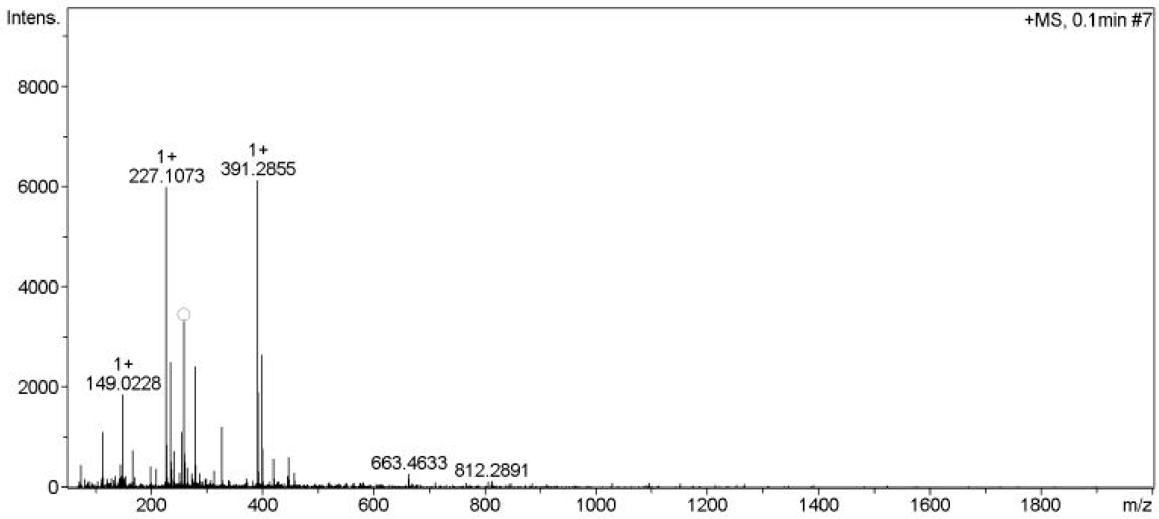

Meas. m/z	#	Ion Formula	m/z	err [mDa]	err [ppm]	rdb	N-Rule	e⁻ Conf	mSigma
259.132967	1	C16H19O3	259.132871	0.1	0.4	7.5	ok	even	77.2

Figure S121: HRMS (m/z)-APCI spectrum of ***6d****.*

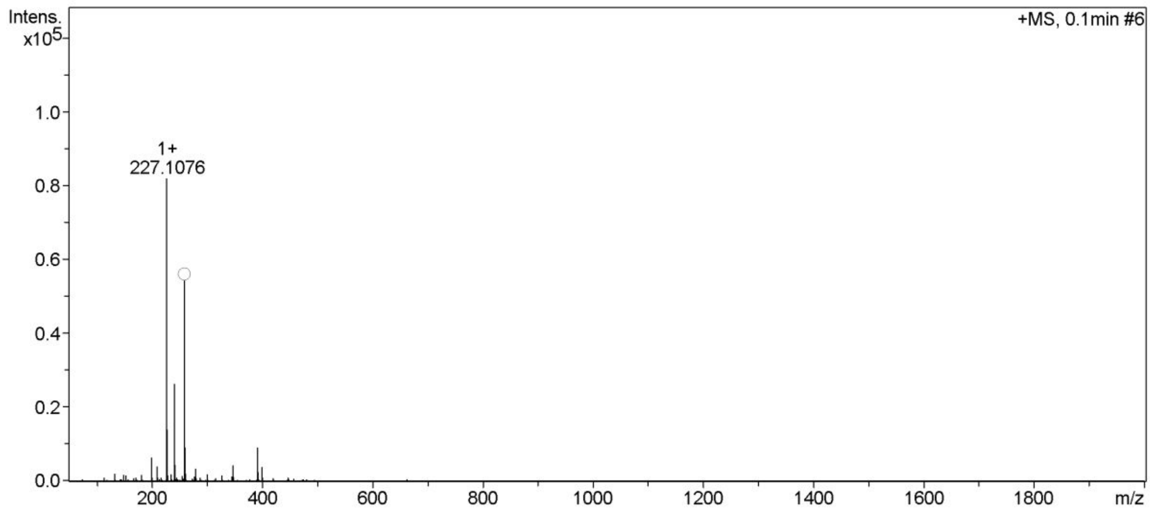

Meas. m/z	#	Ion Formula	m/z	err [mDa]	err [ppm]	rdb	N-Rule	e⁻ Conf	mSigma
259.132994	1	C16H19O3	259.132871	-0.1	-0.5	7.5	ok	even	10.8

Figure S122: HRMS (m/z)-APCI spectrum of ***6e****.*

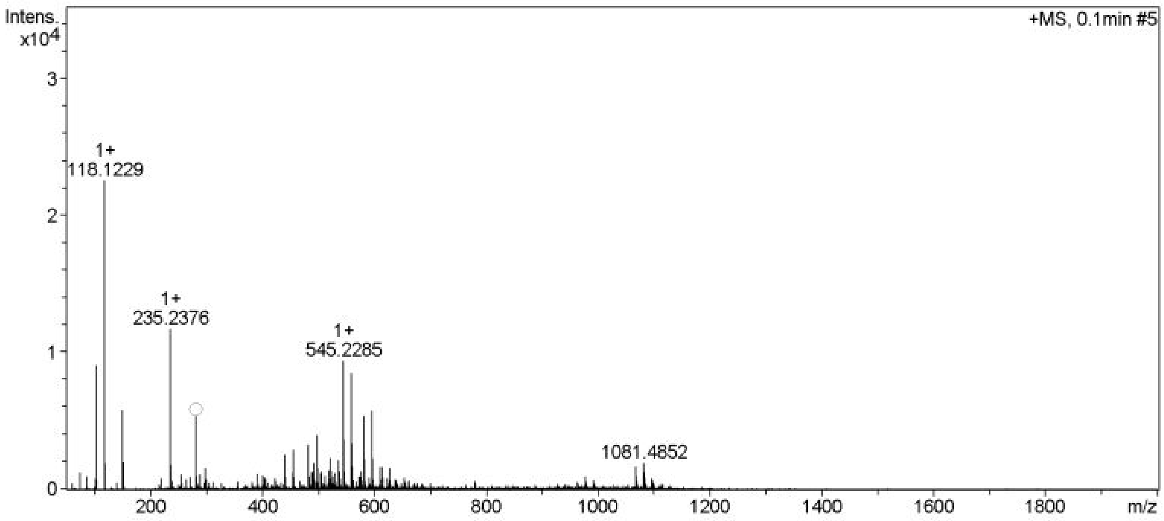

Meas. m/z	#	Ion Formula	m/z	err [mDa]	err [ppm]	rdb	N-Rule	e⁻ Conf	mSigma
281.114695	1	C16H18NaO3	281.114815	0.1	0.4	7.5	ok	even	10.0

*Figure S123: HRMS (m/z)- ESI spectrum of **6f**.*

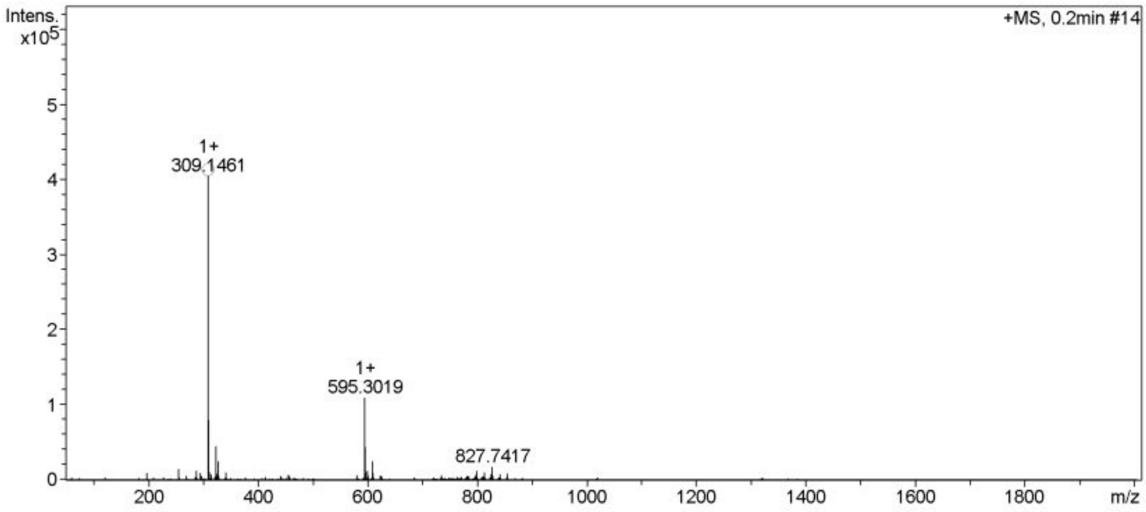

Meas. m/z	#	Ion Formula	m/z	err [mDa]	err [ppm]	rdb	N-Rule	e⁻ Conf	mSigma
309.146114	1	C18H22NaO3	309.146115	-0.0	-0.0	7.5	ok	even	0.4

*Figure S124: HRMS (m/z)- ESI spectrum of **6g**.*

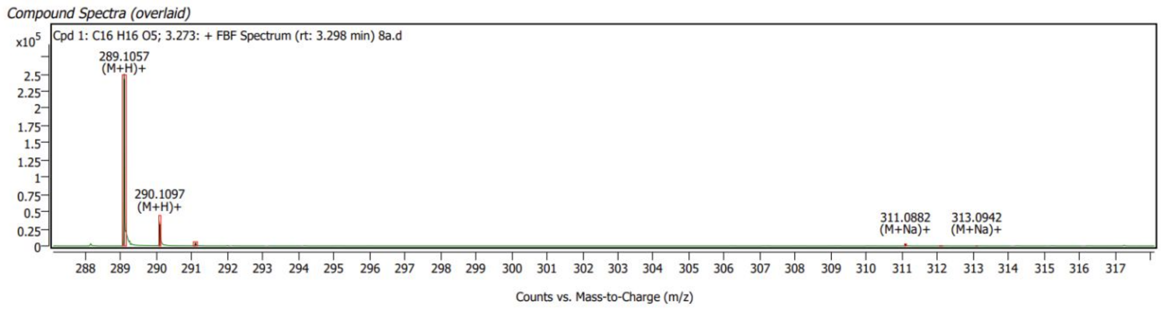

Compound ID Table

Name	Formula	Species	RT	RT Diff	Mass	CAS	ID Source	Score	Score (Lib)	Score (Tgt)
	C16 H16 O5	(M+H)+ (M+Na)+	3.273		288.0985		FBF	87.08		87.08

*Figure S125 HRMS (m/z)- ESI spectrum for **6h.***

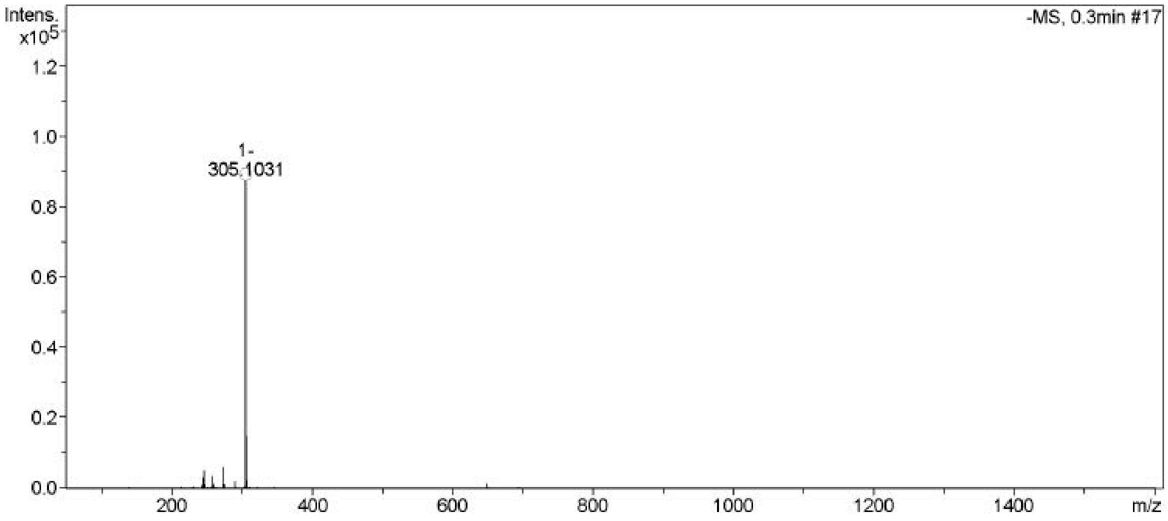

Meas. m/z	#	Ion Formula	m/z	err [mDa]	err [ppm]	rdb	N-Rule	e⁻ Conf	mSigma
305.103117	1	C16H17O6	305.103062	-0.1	-0.2	8.5	ok	even	3.9

Figure S126 HRMS (m/z)- APCI spectrum for ***6i.***

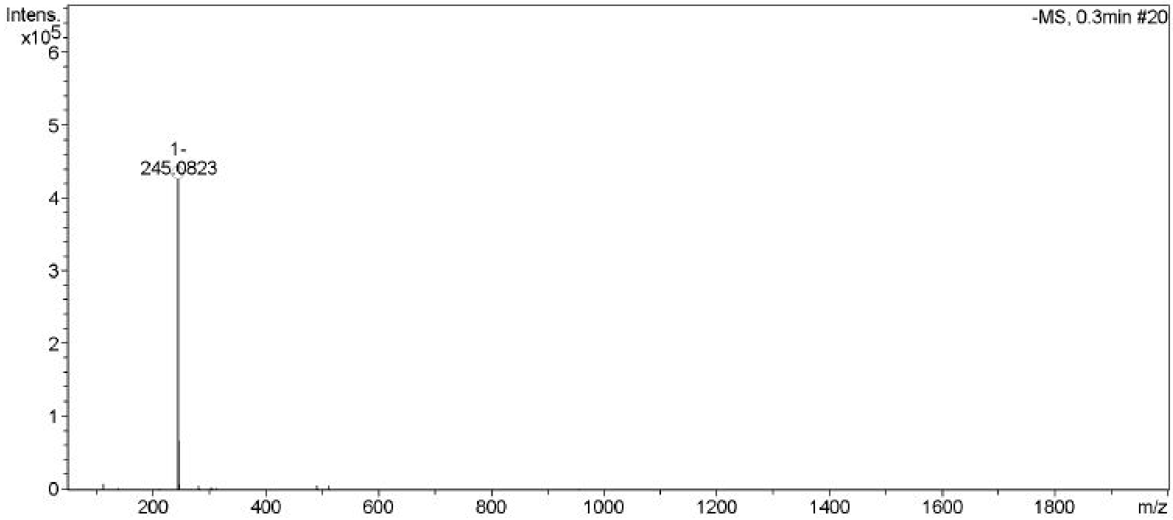

Meas. m/z	#	Ion Formula	m/z	err [mDa]	err [ppm]	rdb	N-Rule	e⁻ Conf	mSigma
245.082276	1	C14H13O4	245.081932	0.3	1.4	8.5	ok	even	1.7

Figure S127 HRMS (m/z)- ESI spectrum for ***6j.***

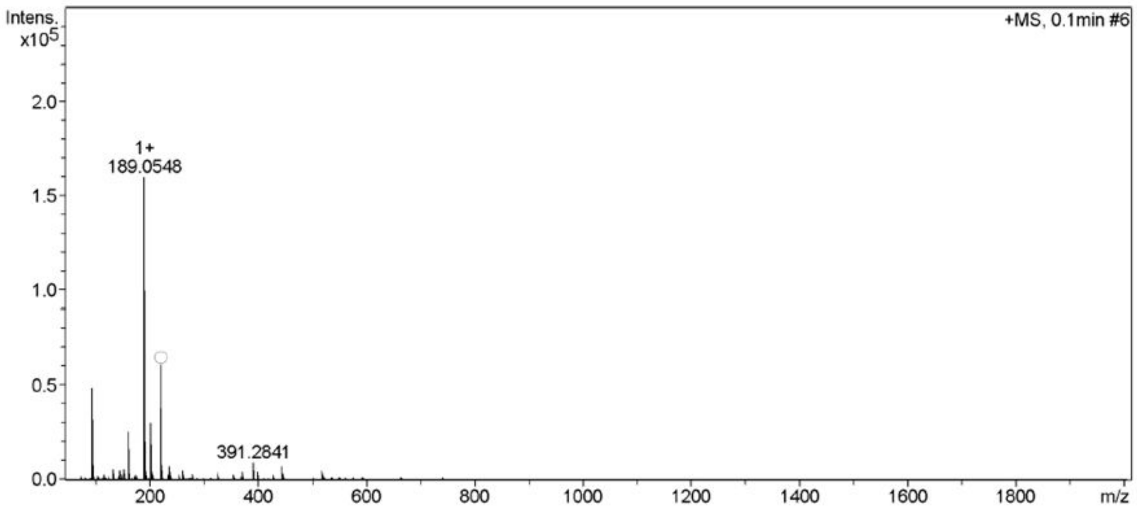

Meas. m/z	#	Ion Formula	m/z	err [mDa]	err [ppm]	rdb	N-Rule	e⁻ Conf	mSigma
221.080542	1	C12H13O4	221.080835	0.3	1.3	6.5	ok	even	5.3

Figure S128: HRMS (m/z)-APCI spectrum of ***6k****.*

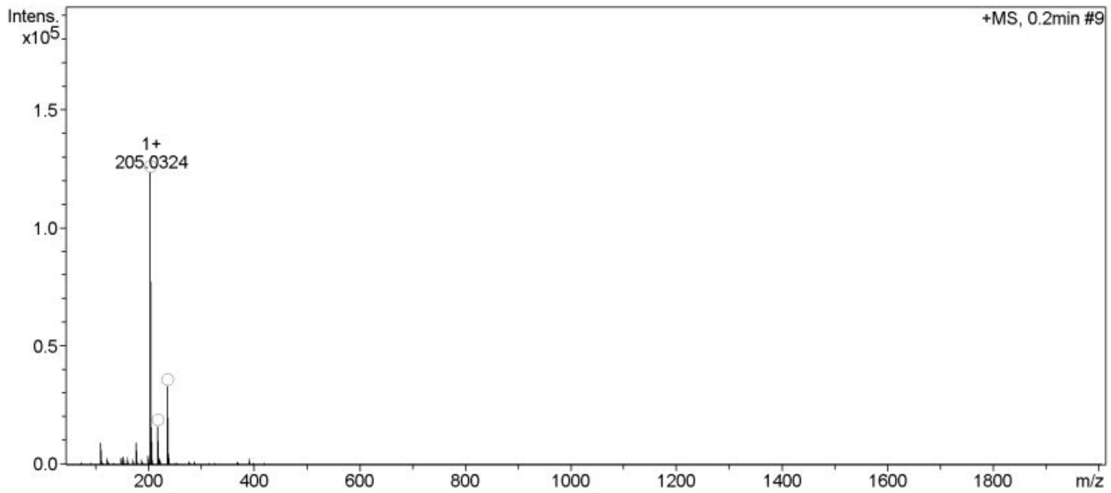

Meas. m/z	#	Ion Formula	m/z	err [mDa]	err [ppm]	rdb	N-Rule	e⁻ Conf	mSigma
205.032412	1	C11H9O2S	205.031777	-0.6	-3.1	7.5	ok	even	1.8
219.047641	1	C12H11O2S	219.047427	-0.2	-1.0	7.5	ok	even	8.8
237.058430	1	C12H13O3S	237.057992	0.4	1.8	6.5	ok	even	3.5

Figure S129: HRMS (m/z)-APCI spectrum of ***6l****.*

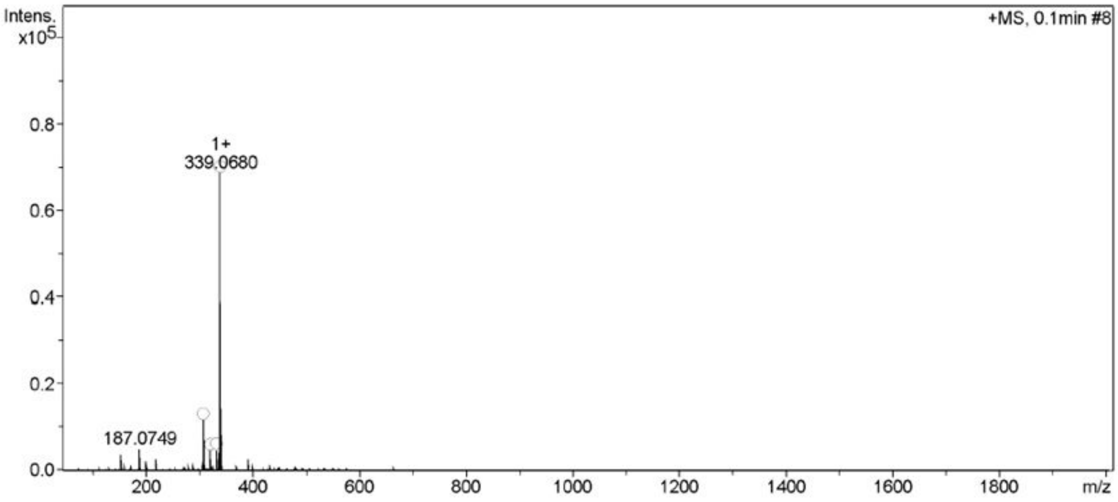

Meas. m/z	#	Ion Formula	m/z	err [mDa]	err [ppm]	rdb	N-Rule	e⁻ Conf	mSigma
307.041730	1	C16H3N8	307.047519	5.8	18.9	19.5	ok	even	5.6
	2	C13H11FeN6	307.038916	2.8	9.2	11.5	ok	even	6.0
	3	C17H3N6O	307.036285	-5.4	-17.7	19.5	ok	even	7.9
	4	C17H15FeO2	307.041608	-0.1	-0.4	10.5	ok	even	10.0
321.059236	1	C12H17FeN4O3	321.064464	5.2	16.3	6.5	ok	even	11.3
	2	C12H5N10O2	321.059146	0.1	0.3	15.5	ok	even	15.6
	3	C14H13FeN6	321.054567	4.7	14.5	11.5	ok	even	20.8
	4	C18H17FeO2	321.057259	2.0	6.2	10.5	ok	even	29.1
	5	C17H5N8	321.063169	-3.9	-12.2	19.5	ok	even	35.0
332.147301	1	C14H18N7O3	332.146564	0.7	2.2	9.5	ok	even	16.4
339.067981	1	C18H19FeO3	339.067825	-0.2	-0.5	9.5	ok	even	13.8

Figure S130: HRMS (m/z)-APCI spectrum of ***6m****.*

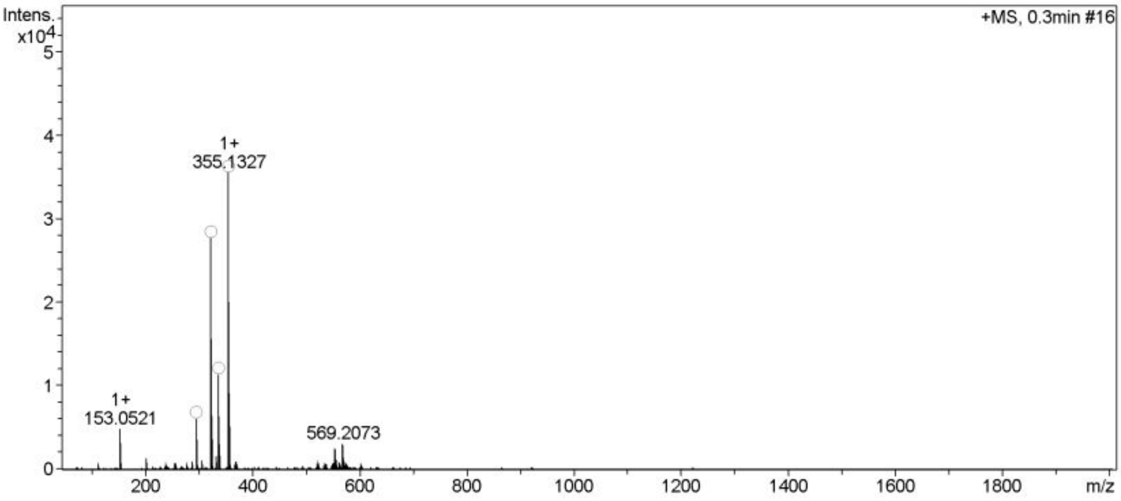

Meas. m/z	#	Ion Formula	m/z	err [mDa]	err [ppm]	rdb	N-Rule	e⁻ Conf	mSigma
295.110466	1	C22H15O	295.111742	-1.3	-4.3	15.5	ok	even	21.5
323.106276	1	C23H15O2	323.106656	-0.4	-1.2	16.5	ok	even	12.1
337.122478	1	C24H17O2	337.122306	-0.2	-0.5	16.5	ok	even	12.7
355.132720	1	C24H19O3	355.132871	-0.2	-0.4	15.5	ok	even	3.4

Figure S131: HRMS (m/z)-APCI spectrum of ***6n****.*

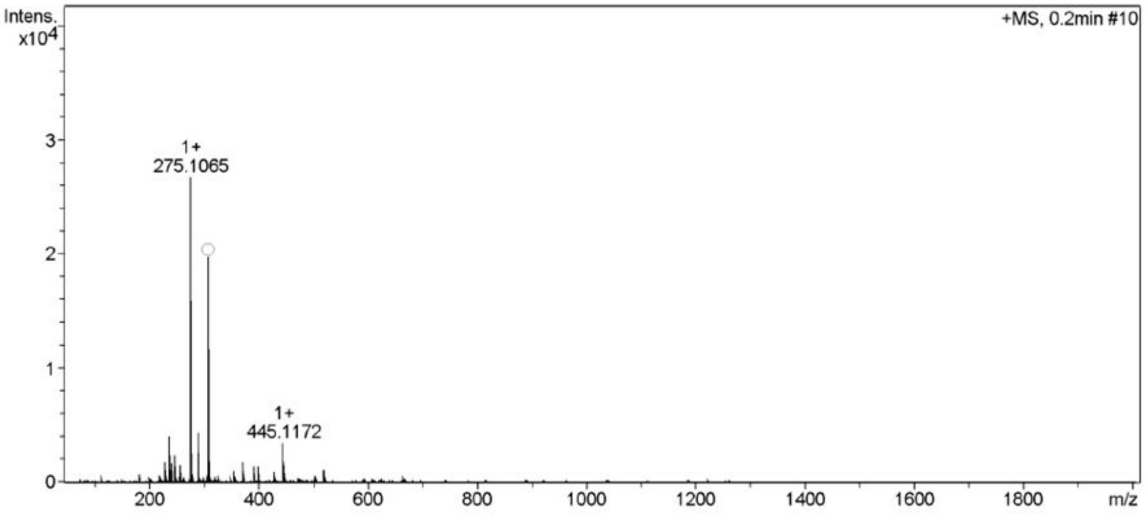

Meas. m/z	#	Ion Formula	m/z	err [mDa]	err [ppm]	rdb	N-Rule	e⁻ Conf	mSigma
307.133143	1	C20H19O3	307.132871	-0.3	-0.9	11.5	ok	even	2.8

*Figure S132: HRMS (m/z)-APCI spectrum of **6o.***

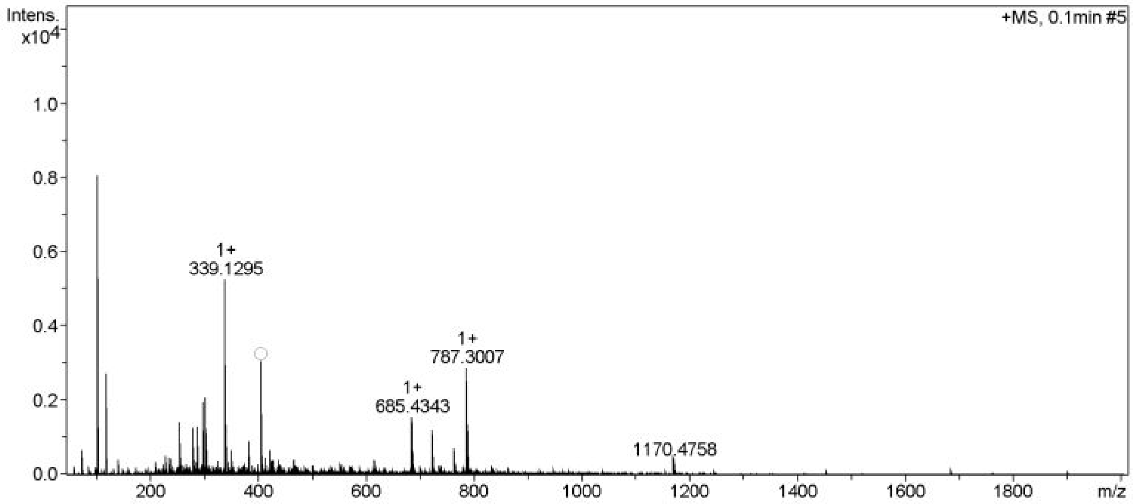

Meas. m/z	#	Ion Formula	m/z	err [mDa]	err [ppm]	rdb	N-Rule	e⁻ Conf	mSigma
405.144936	1	C26H22NaO3	405.146115	1.2	2.9	15.5	ok	even	62.0

*Figure S133: HRMS (m/z)- ESI spectrum of **6p**.*

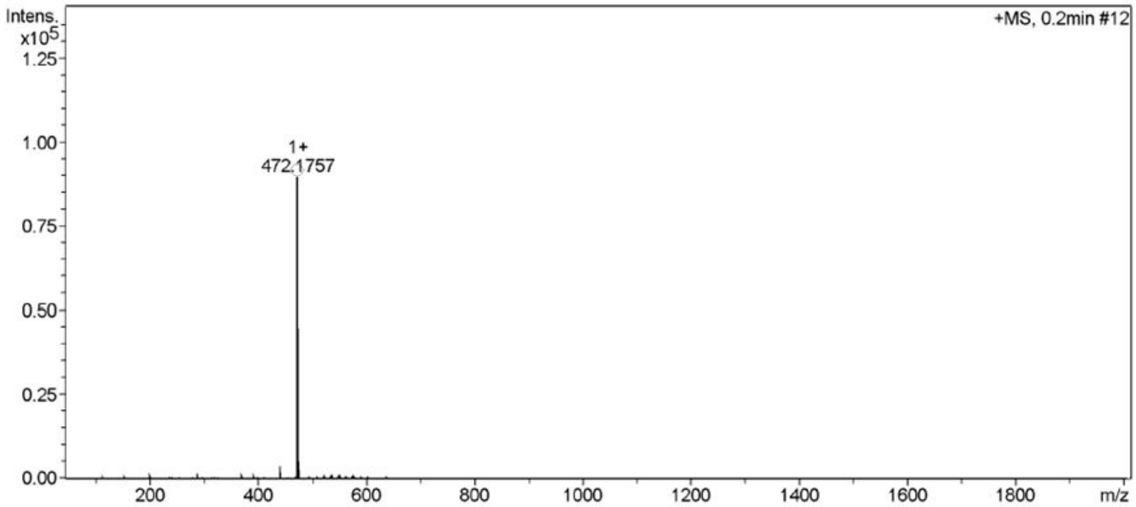

Meas. m/z	#	Ion Formula	m/z	err [mDa]	err [ppm]	rdb	N-Rule	e⁻ Conf	mSigma
472.175697	1	C28H26NO6	472.175464	0.2	0.5	16.5	ok	even	10.0

*Figure S134: HRMS (m/z)-APCI spectrum of **6q**.*

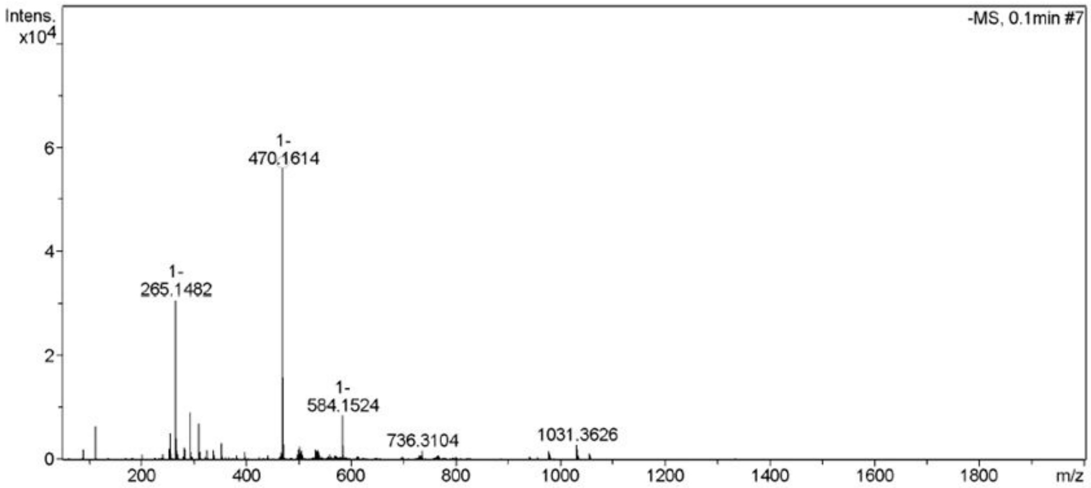

Meas. m/z	#	Ion Formula	m/z	err [mDa]	err [ppm]	rdb	N-Rule	e⁻ Conf	mSigma
470.161362	1	C28H24NO6	470.160911	-0.5	-1.0	17.5	ok	even	13.8

*Figure S135: HRMS (m/z)- ESI spectrum of **6r.***

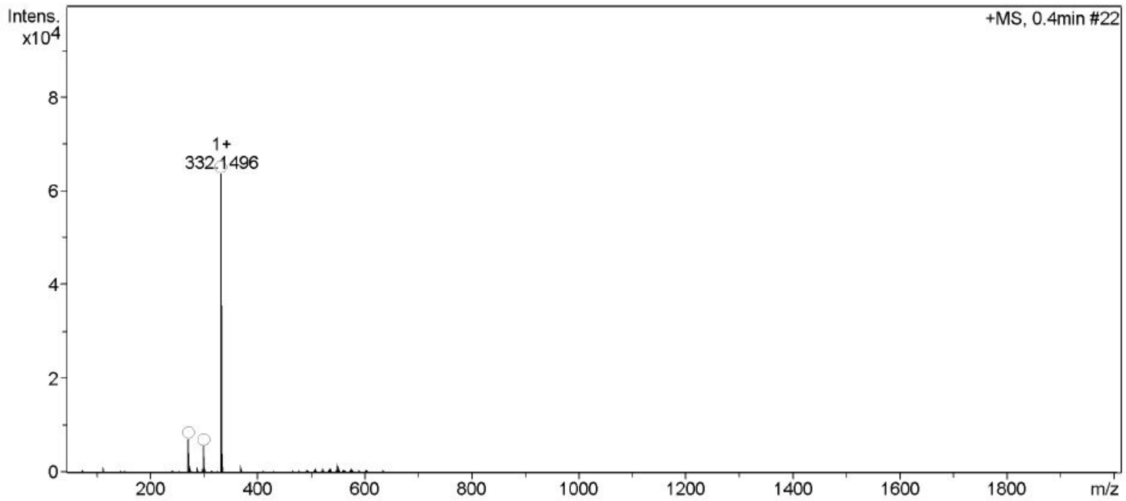

Meas. m/z	#	Ion Formula	m/z	err [mDa]	err [ppm]	rdb	N-Rule	e⁻ Conf	mSigma
272.127871	1	C16H18NO3	272.128120	-0.2	-0.9	8.5	ok	even	16.7
300.125013	1	C17H18NO4	300.123034	-2.0	-6.6	9.5	ok	even	13.9
332.149622	1	C18H22NO5	332.149249	-0.4	-1.1	8.5	ok	even	5.7

Figure S136: HRMS (m/z)- APCI spectrum of ***6s.***

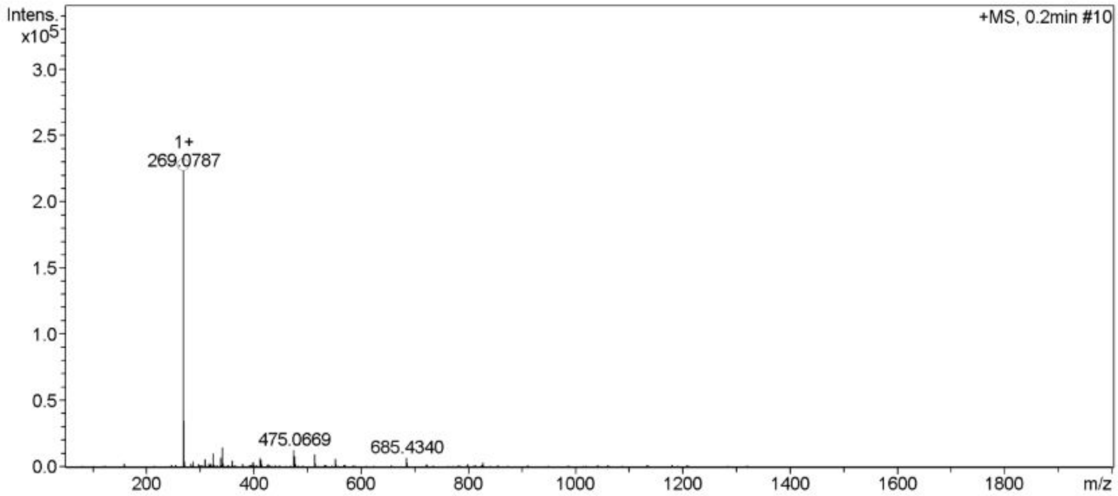

Meas. m/z	#	Ion Formula	m/z	err [mDa]	err [ppm]	rdb	N-Rule	e⁻ Conf	mSigma
269.078651	1	C14H14NaO4	269.078430	0.2	0.8	7.5	ok	even	0.4

Figure S137: HRMS (m/z)- APCI spectrum of ***6t.***

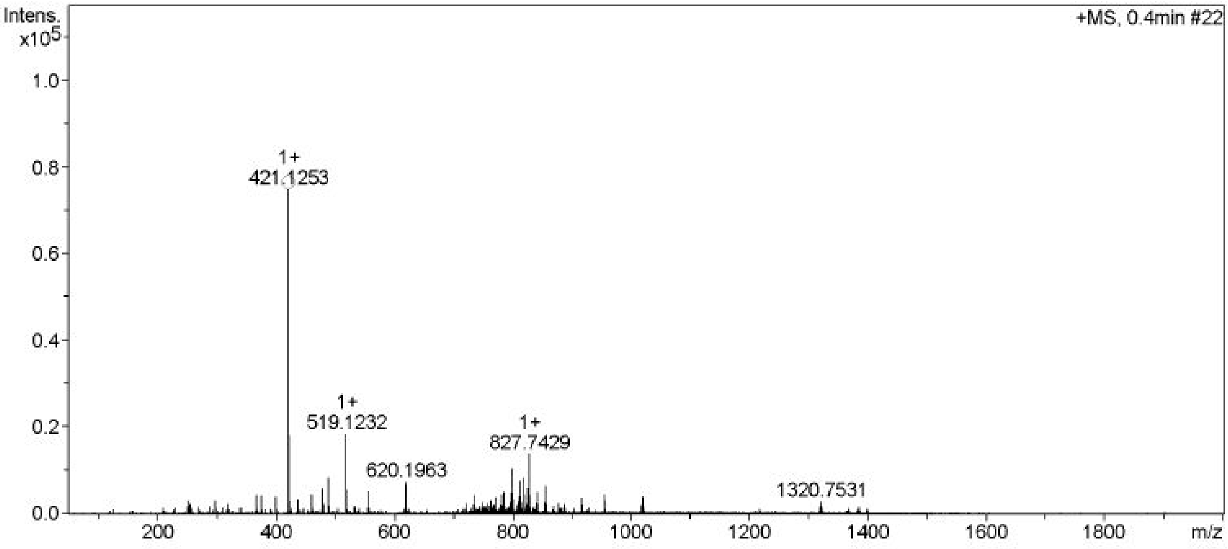

Meas. m/z	#	Ion Formula	m/z	err [mDa]	err [ppm]	rdb	N-Rule	e⁻ Conf	mSigma
421.125269	1	C22H22NaO7	421.125774	-0.5	-1.2	11.5	ok	even	2.5

*Figure S138: HRMS (m/z)- ESI spectrum of **6u.***

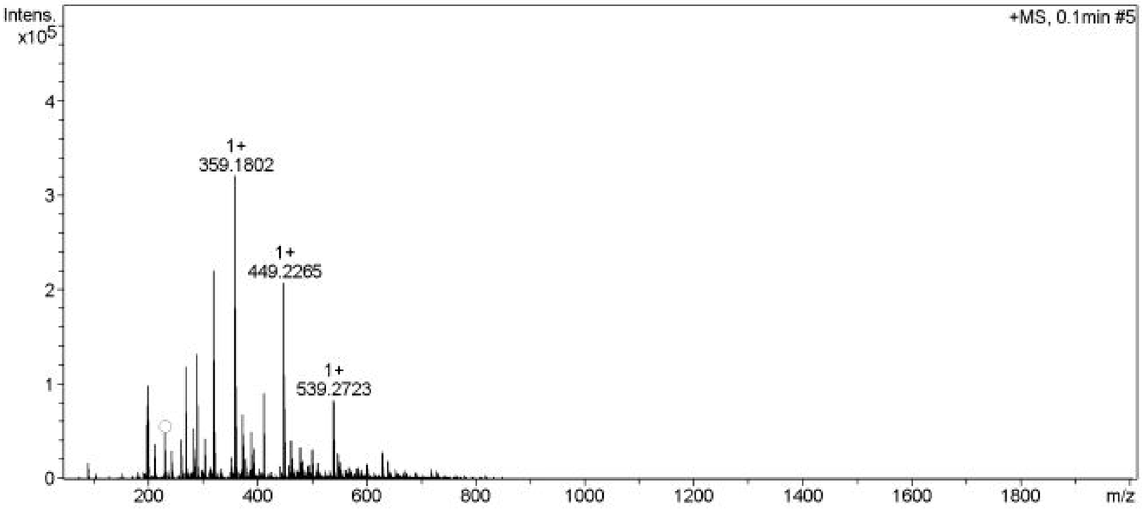

Meas. m/z	#	Ion Formula	m/z	err [mDa]	err [ppm]	rdb	N-Rule	e⁻ Conf	mSigma
231.102113	1	C14H15O3	231.101571	-0.5	-2.3	7.5	ok	even	16.2

*Figure S139: HRMS (m/z)- APCI spectrum of **6v**.*

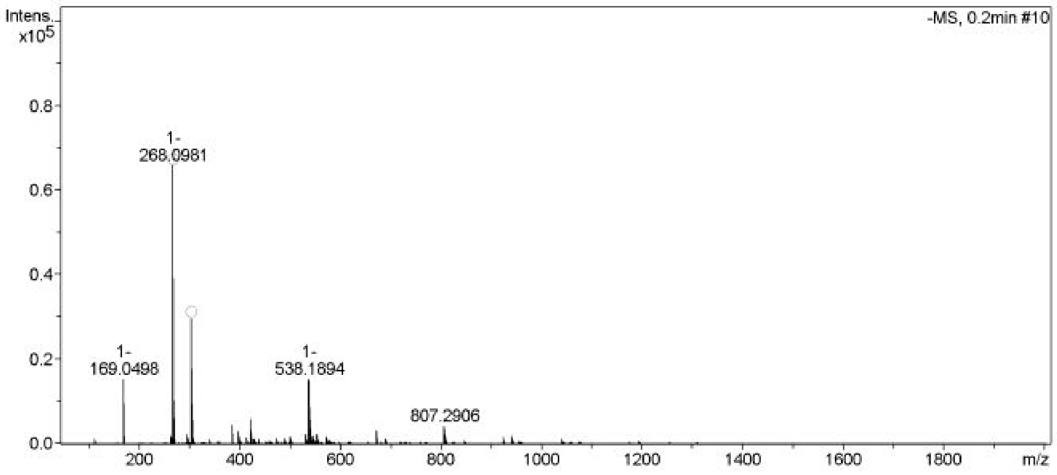

Meas. m/z	#	Ion Formula	m/z	err [mDa]	err [ppm]	rdb	N-Rule	e⁻ Conf	mSigma
268.098077	1	C16H14NO3	268.097917	0.2	0.6	10.5	ok	even	13.5
304.073944	1	C16H15ClNO3	304.074595	0.7	2.1	9.5	ok	even	11.5

*Figure S140: HRMS (m/z)- APCI spectrum of **6ab**.*

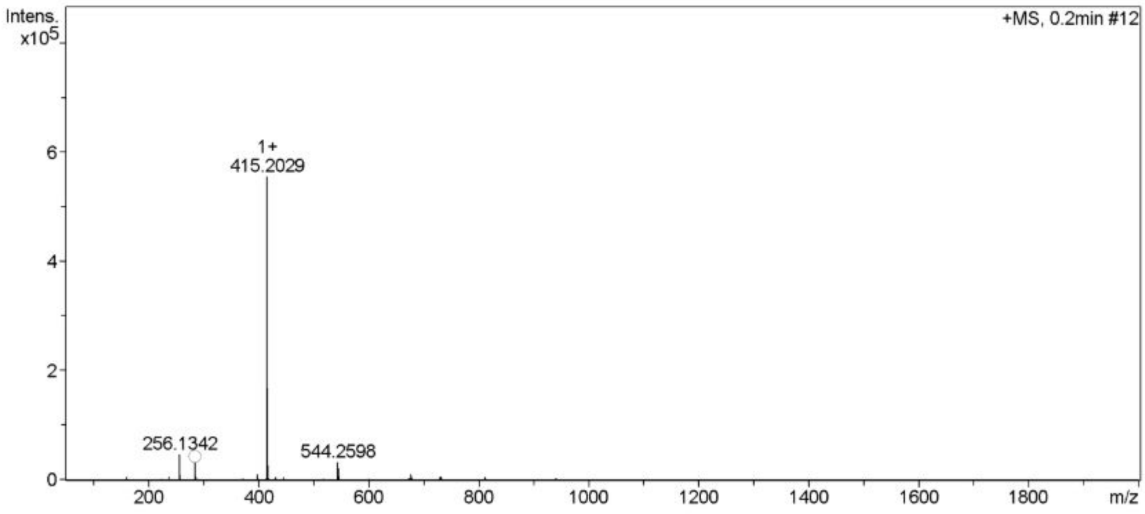

Meas. m/z	#	Ion Formula	m/z	err [mDa]	err [ppm]	rdb	N-Rule	e⁻ Conf	mSigma
284.128308	1	C17H18NO3	284.128120	-0.2	-0.7	9.5	ok	even	17.5

*Figure S141: HRMS (m/z)- APCI spectrum of **6ac**.*

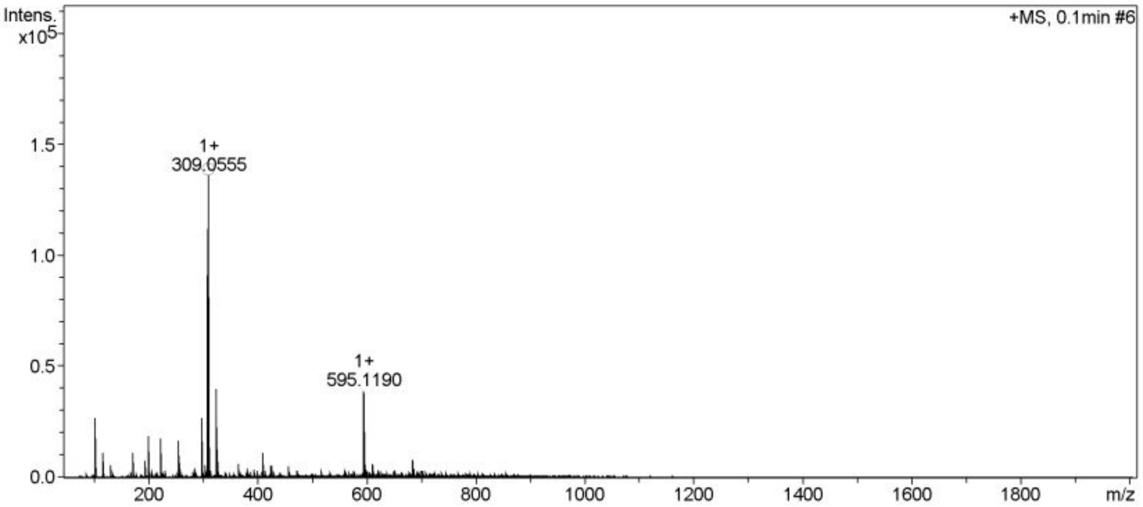

Meas. m/z	#	Ion Formula	m/z	err [mDa]	err [ppm]	rdb	N-Rule	e⁻ Conf	mSigma
309.055520	1	C16H14NaO3S	309.055586	0.1	0.2	9.5	ok	even	5.5

Figure S142: HRMS (m/z)- ESI spectrum of ***6ad****.*

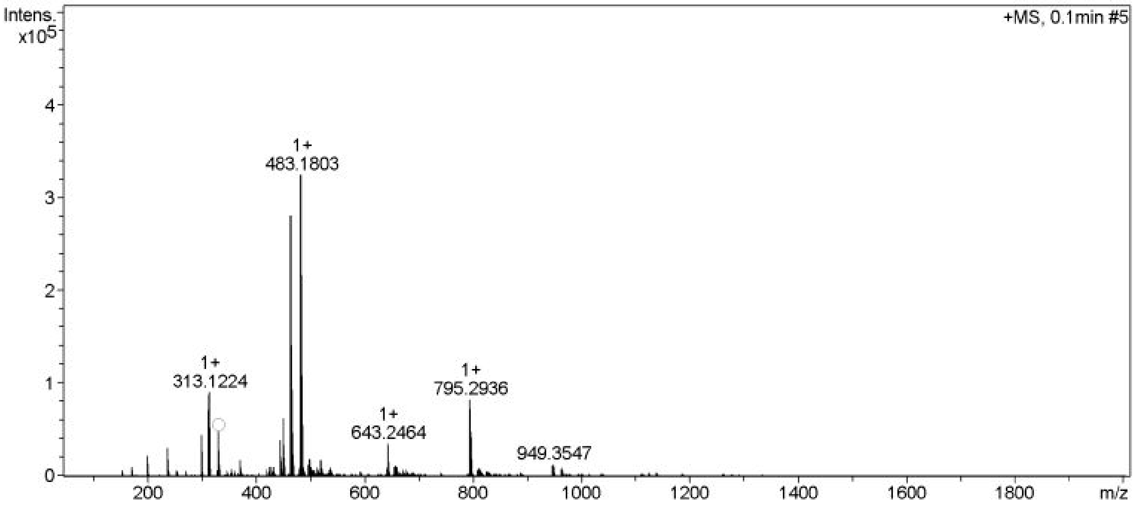

Meas. m/z	#	Ion Formula	m/z	err [mDa]	err [ppm]	rdb	N-Rule	e⁻ Conf	mSigma
331.132148	1	C22H19O3	331.132871	0.7	2.2	13.5	ok	even	14.0

Figure S143: HRMS (m/z)- APCI spectrum of ***6ae****.*

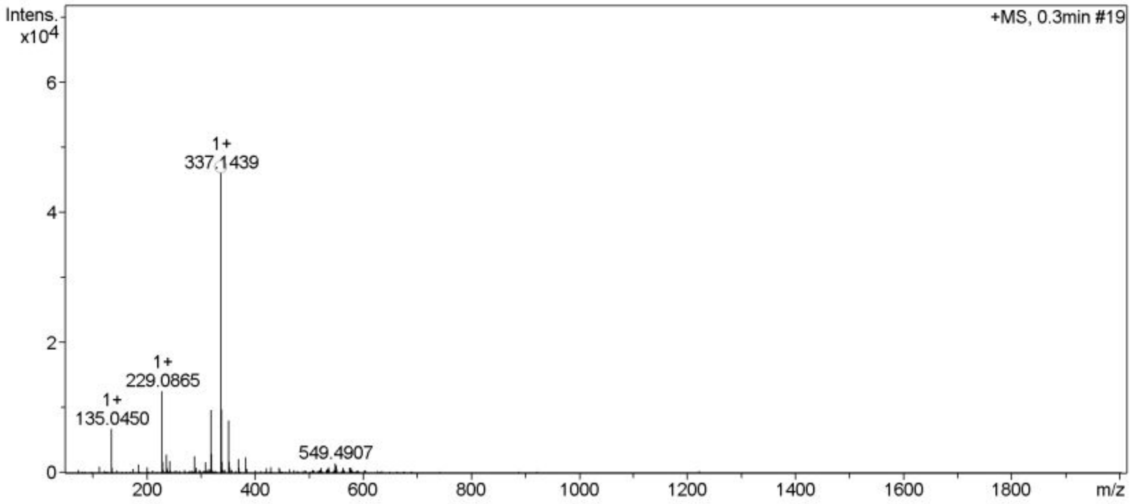

Meas. m/z	#	Ion Formula	m/z	err [mDa]	err [ppm]	rdb	N-Rule	e⁻ Conf	mSigma
337.143883	1	C21H21O4	337.143436	-0.4	-1.3	11.5	ok	even	9.3

*Figure S144: HRMS (m/z)- ESI spectrum of **7a**.*

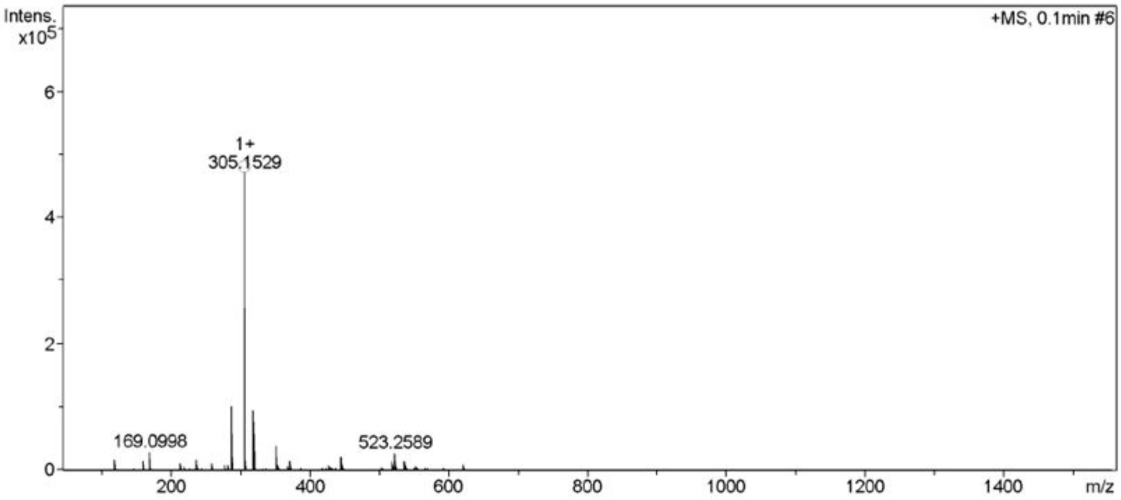

Meas. m/z	#	Ion Formula	m/z	err [mDa]	err [ppm]	rdb	N-Rule	e⁻ Conf	mSigma
305.152950	1	C21H21O2	305.153606	-0.7	-2.2	11.5	ok	even	5.6

*Figure S145: HRMS (m/z)-APCI spectrum of **7b**.*

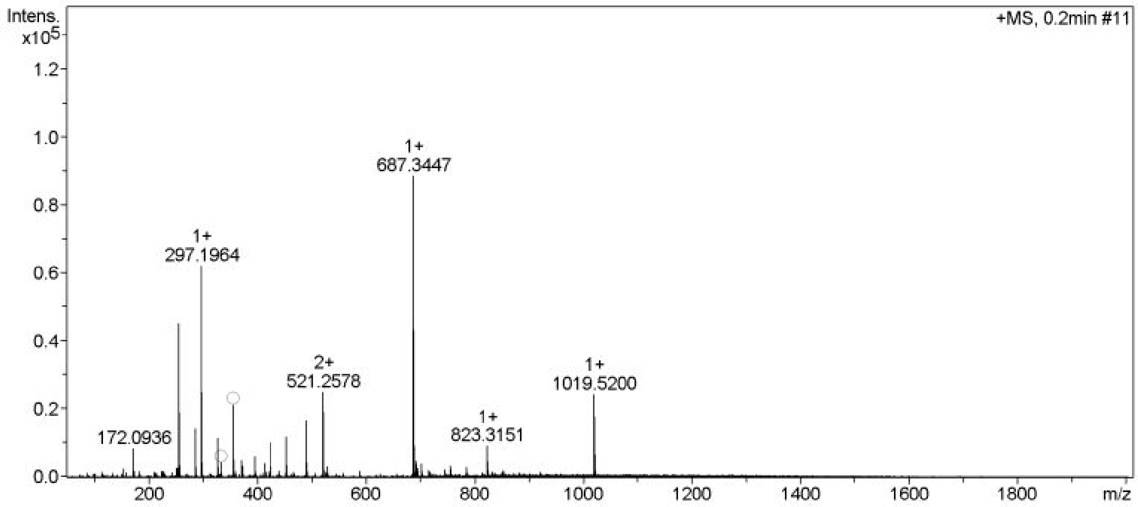

Meas. m/z	#	Ion Formula	m/z	err [mDa]	err [ppm]	rdb	N-Rule	e⁻ Conf	mSigma
333.184265	1	C23H25O2	333.184906	-0.6	-1.9	11.5	ok	even	23.0
355.166094	1	C23H24NaO2	355.166851	-0.8	-2.1	11.5	ok	even	10.2

Figure S146: HRMS (m/z)-APCI spectrum of ***7c****.*

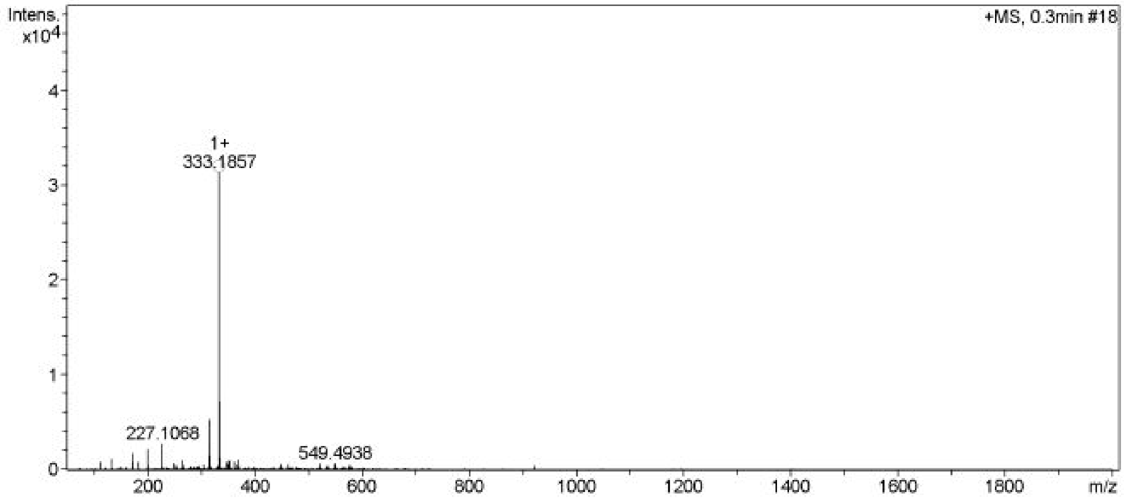

Meas. m/z	#	Ion Formula	m/z	err [mDa]	err [ppm]	rdb	N-Rule	e⁻ Conf	mSigma
333.185681	1	C23H25O2	333.184906	-0.8	-2.3	11.5	ok	even	12.5

Figure S147: HRMS (m/z)-APCI spectrum of ***7d****.*

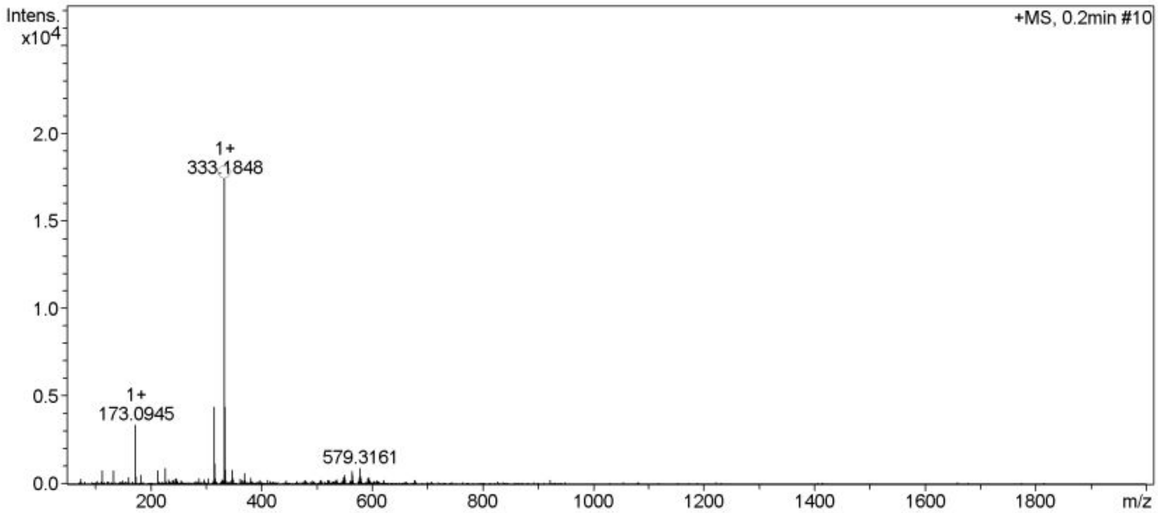

Meas. m/z	#	Ion Formula	m/z	err [mDa]	err [ppm]	rdb	N-Rule	e⁻ Conf	mSigma
333.184786	1	C23H25O2	333.184906	-0.1	-0.4	11.5	ok	even	7.1

*Figure S148: HRMS (m/z)-APCI spectrum of **7e**.*

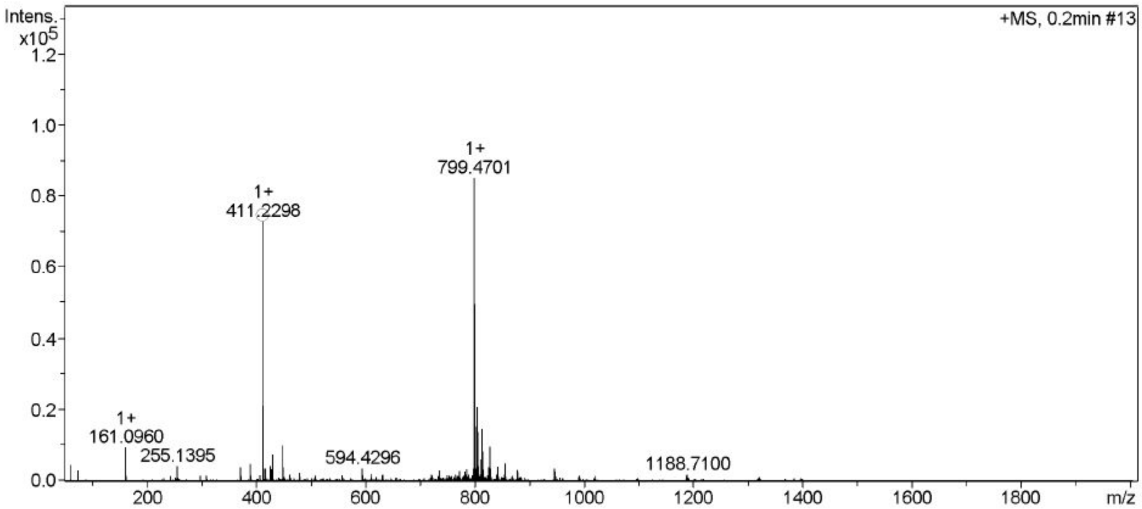

Meas. m/z	#	Ion Formula	m/z	err [mDa]	err [ppm]	rdb	N-Rule	e⁻ Conf	mSigma
411.229753	1	C27H32NaO2	411.229451	-0.3	-0.7	11.5	ok	even	3.2

*Figure S149: HRMS (m/z)-ESI spectrum of **7f**.*

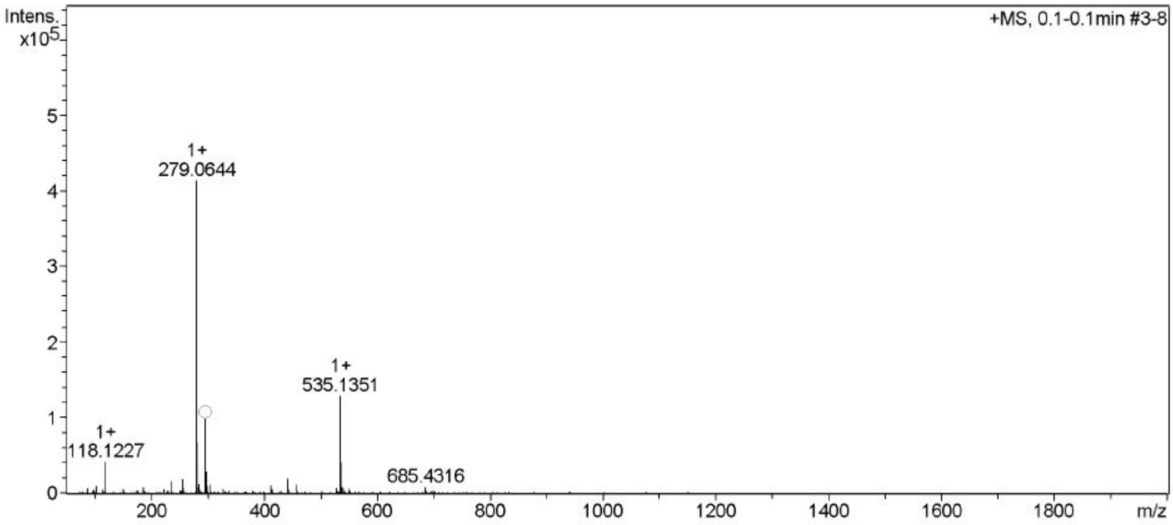

Meas. m/z	#	Ion Formula	m/z	err [mDa]	err [ppm]	rdb	N-Rule	e⁻ Conf	mSigma
295.037488	1	C15H12KO4	295.036717	0.8	2.6	9.5	ok	even	79.7

Figure S150: HRMS (m/z)-APCI spectrum of ***7g****.*

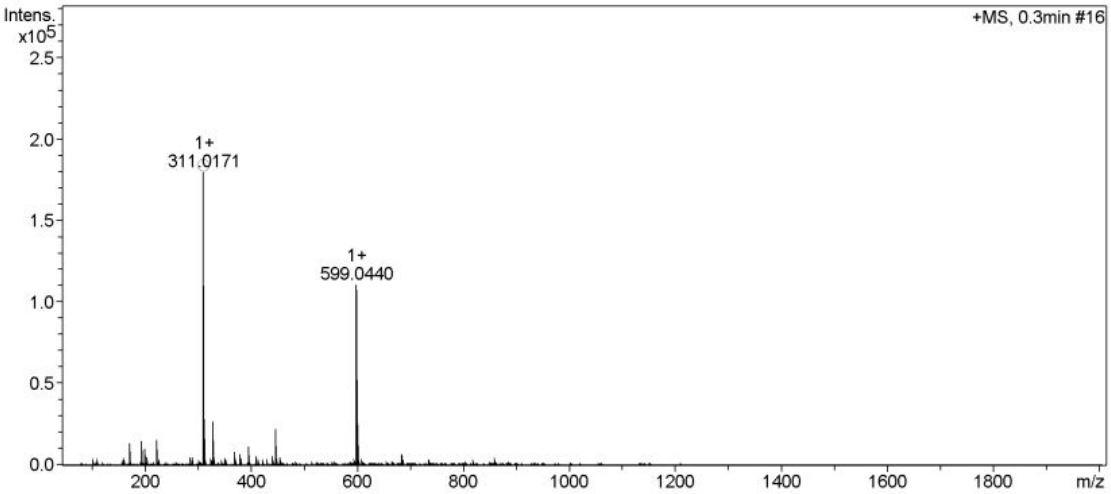

Meas. m/z	#	Ion Formula	m/z	err [mDa]	err [ppm]	rdb	N-Rule	e⁻ Conf	mSigma
311.017121	1	C15H12NaO2S2	311.017092	-0.0	-0.1	9.5	ok	even	10.8

Figure S151: HRMS (m/z)- ESI spectrum of ***7h****.*

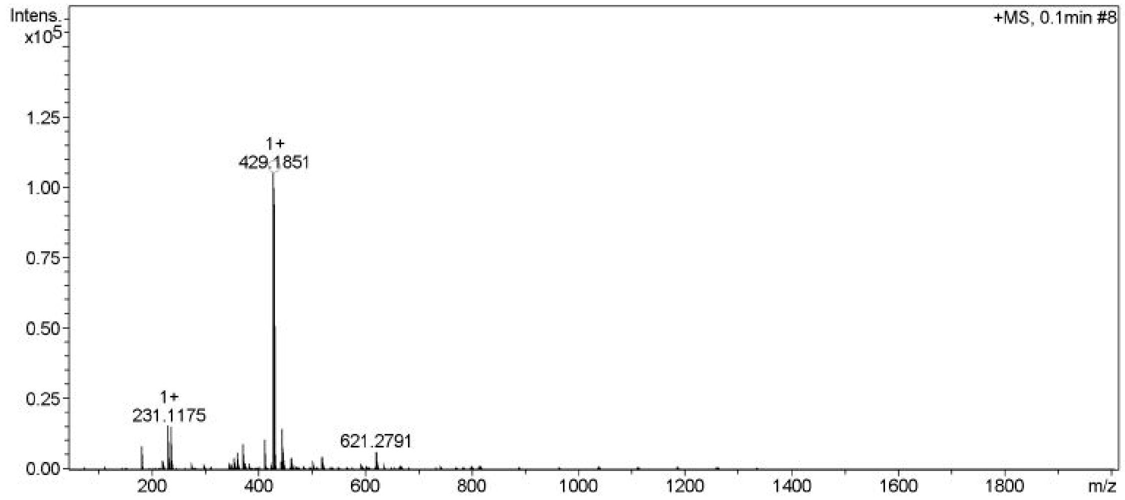

Meas. m/z	#	Ion Formula	m/z	err [mDa]	err [ppm]	rdb	N-Rule	e⁻ Conf	mSigma
429.185135	1	C31H25O2	429.184906	-0.2	-0.5	19.5	ok	even	5.6

*Figure S152: HRMS (m/z)-APCI spectrum of **7i**.*

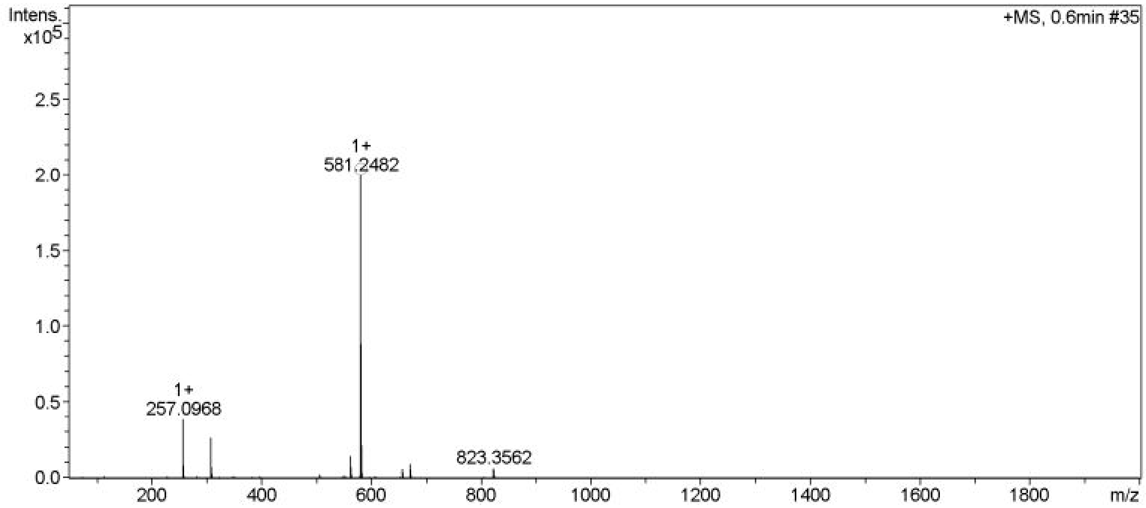

Meas. m/z	#	Ion Formula	m/z	err [mDa]	err [ppm]	rdb	N-Rule	e⁻ Conf	mSigma
581.248226	1	C43H33O2	581.247507	0.7	1.2	27.5	ok	even	14.3

*Figure S153: HRMS (m/z)-APCI spectrum of **7j**.*

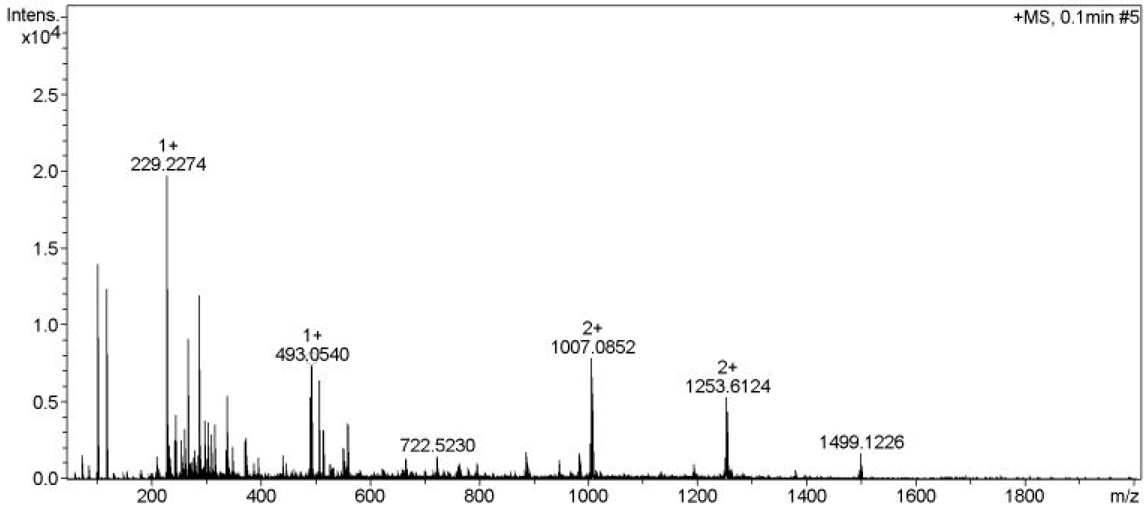

Meas. m/z	#	Ion Formula	m/z	err [mDa]	err [ppm]	rdb	N-Rule	e⁻ Conf	mSigma
493.053965	1	C27H25Fe2O2	493.054856	-0.9	-1.8	15.5	ok	even	258.8

*Figure S154: HRMS (m/z)- ESI spectrum of **7k**.*

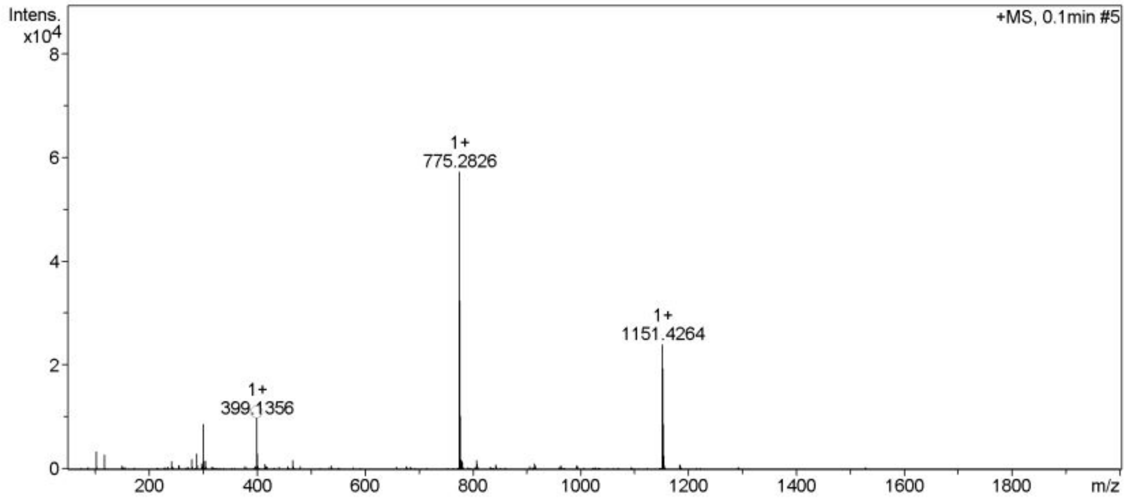

Meas. m/z	#	Ion Formula	m/z	err [mDa]	err [ppm]	rdb	N-Rule	e⁻ Conf	mSigma
399.135605	1	C27H20NaO2	399.135551	-0.1	-0.1	17.5	ok	even	10.0

*Figure S155: HRMS (m/z)-APCI spectrum of **7l.***

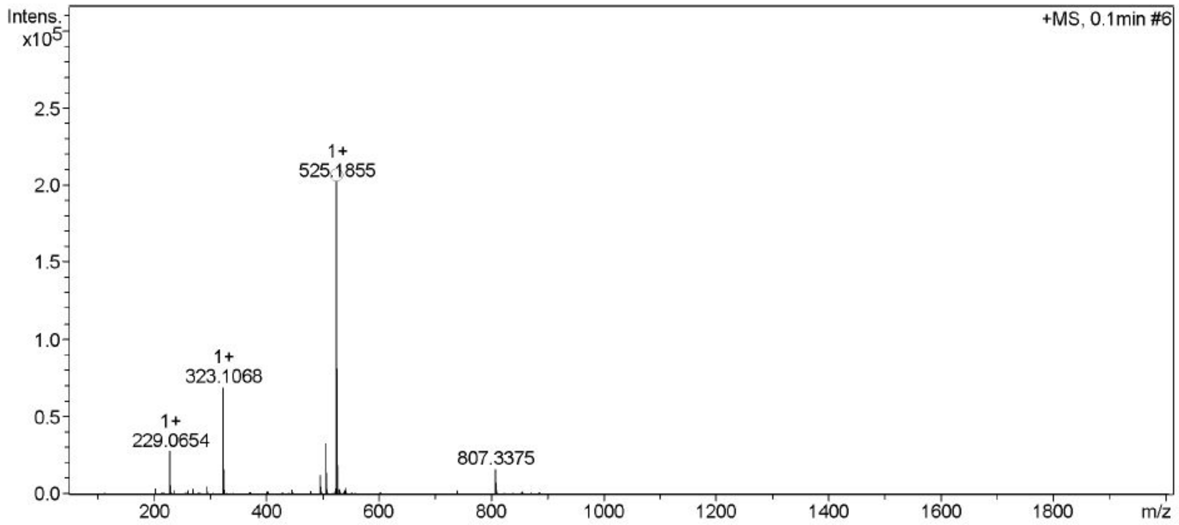

Meas. m/z	#	Ion Formula	m/z	err [mDa]	err [ppm]	rdb	N-Rule	e⁻ Conf	mSigma
525.185526	1	C39H25O2	525.184906	-0.6	-1.2	27.5	ok	even	10.5

Figure S156: HRMS (m/z)-APCI spectrum of ***7m.***

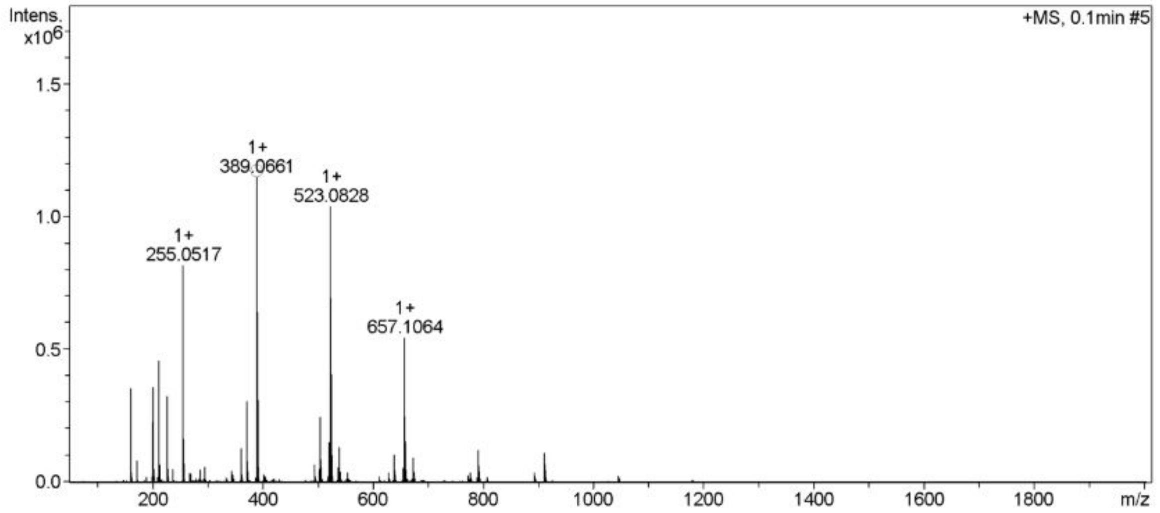

Meas. m/z	#	Ion Formula	m/z	err [mDa]	err [ppm]	rdb	N-Rule	e⁻ Conf	mSigma
389.066084	1	C23H17O2S2	389.066448	-0.4	-0.9	15.5	ok	even	145.1

Figure S157: HRMS (m/z)-ESI spectrum of ***7o.***

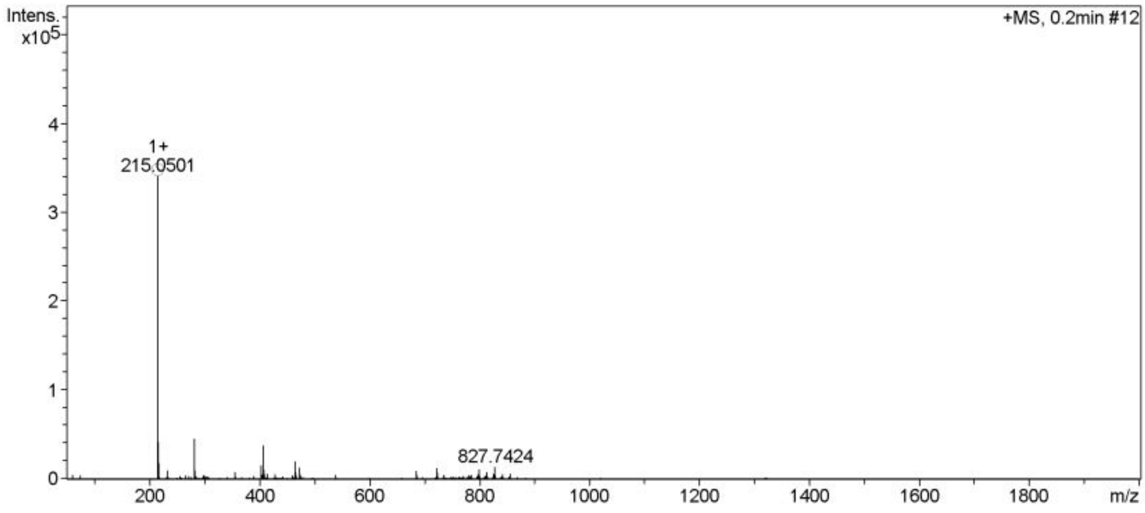

Meas. m/z	#	Ion Formula	m/z	err [mDa]	err [ppm]	rdb	N-Rule	e⁻ Conf	mSigma
215.050125	1	C11H12NaOS	215.050107	-0.0	-0.1	5.5	ok	even	3.9

Figure S158: HRMS (m/z)-ESI spectrum of ***9b.***

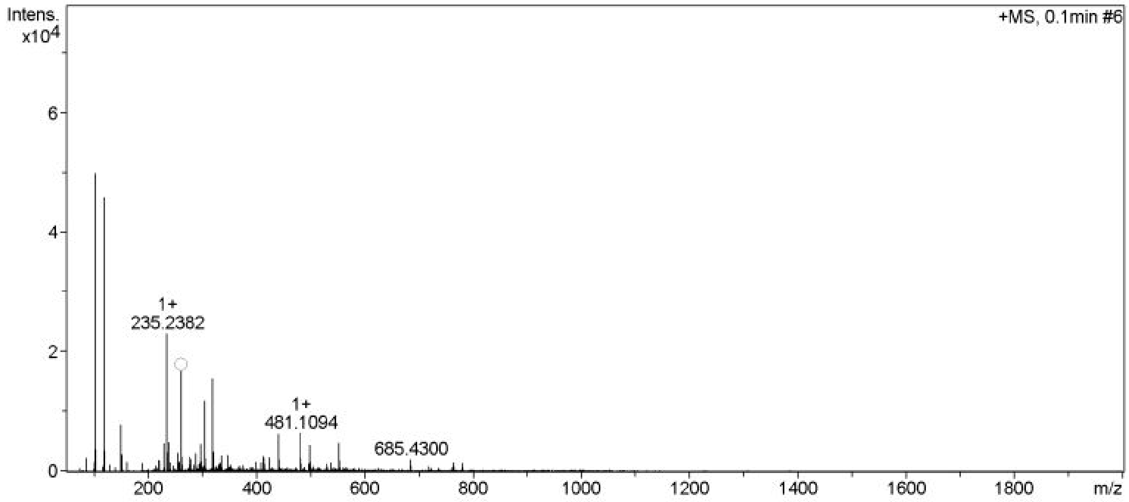

Meas. m/z	#	Ion Formula	m/z	err [mDa]	err [ppm]	rdb	N-Rule	e⁻ Conf	mSigma
261.055850	1	C12H14NaO3S	261.055586	0.3	1.0	5.5	ok	even	6.1

Figure S159: HRMS (m/z)- ESI spectrum of ***10.***

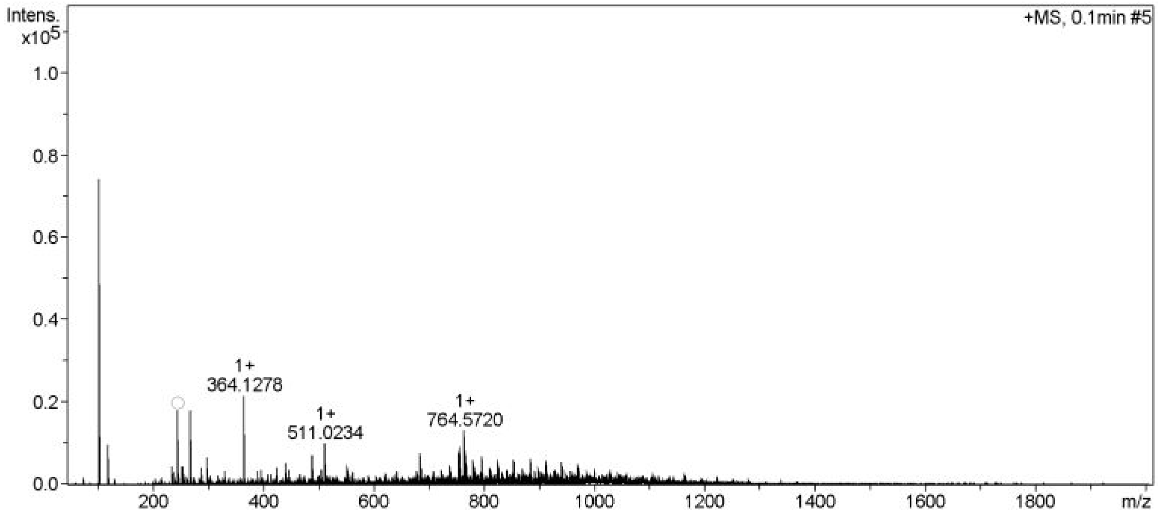

Meas. m/z	#	Ion Formula	m/z	err [mDa]	err [ppm]	rdb	N-Rule	e¯ Conf	mSigma
245.023889	1	C11H10NaO3S	245.024286	0.4	1.6	6.5	ok	even	6.2

*Figure S160: HRMS (m/z)- ESI spectrum of **11**.*

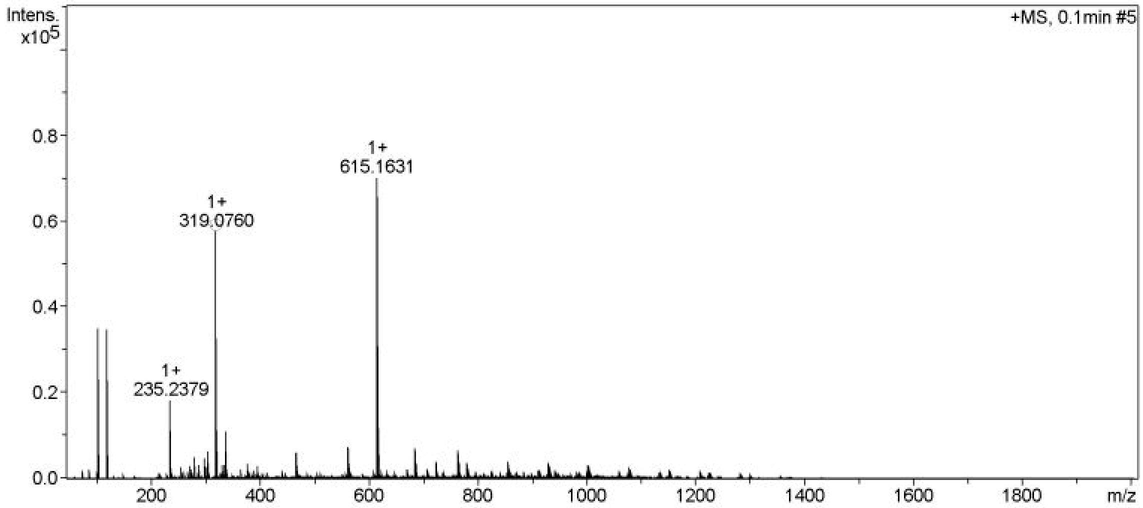

Meas. m/z	#	Ion Formula	m/z	err [mDa]	err [ppm]	rdb	N-Rule	e¯ Conf	mSigma
319.076047	1	C18H16NaO2S	319.076321	-0.3	-0.9	10.5	ok	even	4.4

*Figure S161: HRMS (m/z)- ESI spectrum of **13**.*

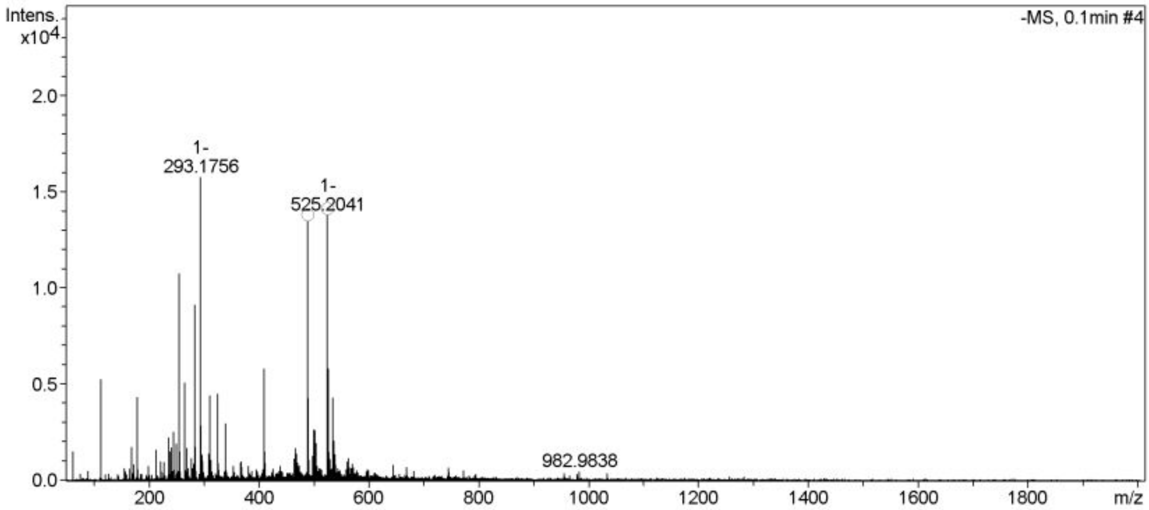

Meas. m/z	#	Ion Formula	m/z	err [mDa]	err [ppm]	rdb	N-Rule	e⁻ Conf	mSigma
489.228429	1	C30H33O6	489.228262	-0.2	-0.3	14.5	ok	even	9.1
525.204114	1	C30H34ClO6	525.204940	0.8	1.6	13.5	ok	even	18.1

*Figure S162: HRMS (m/z)-APCI spectrum of compound **14a**.*

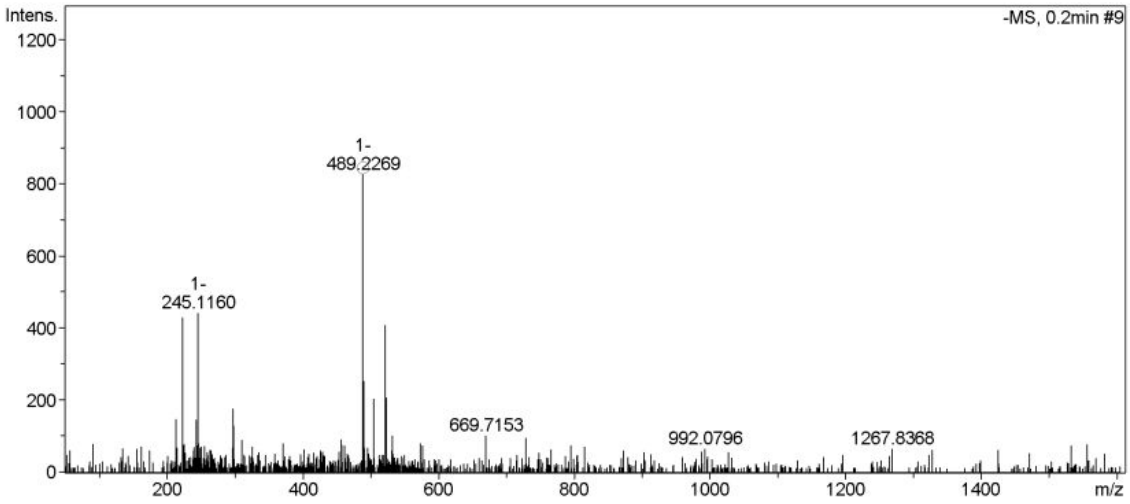

Meas. m/z	#	Ion Formula	m/z	err [mDa]	err [ppm]	rdb	N-Rule	e⁻ Conf	mSigma
489.226879	1	C30H33O6	489.228262	-1.4	-2.8	14.5	ok	even	24.1

*Figure S163: HRMS (m/z)- APCI spectrum of **14b**.*

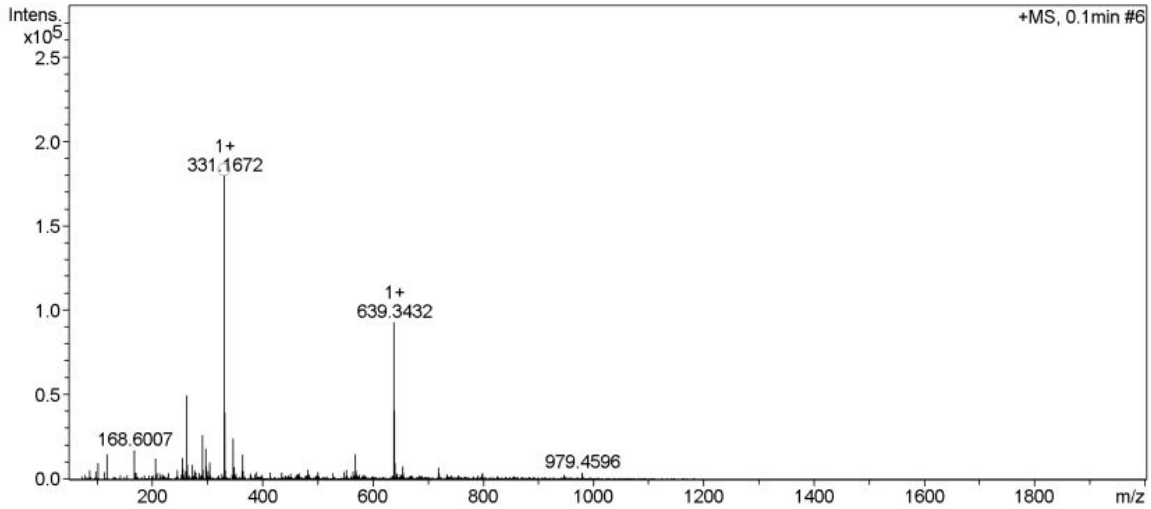

Meas. m/z	#	Ion Formula	m/z	err [mDa]	err [ppm]	rdb	N-Rule	e⁻ Conf	mSigma
331.167196	1	C21H24NaO2	331.166851	0.3	1.0	9.5	ok	even	6.2

*Figure S164: HRMS (m/z)- APCI spectrum of **15**.*

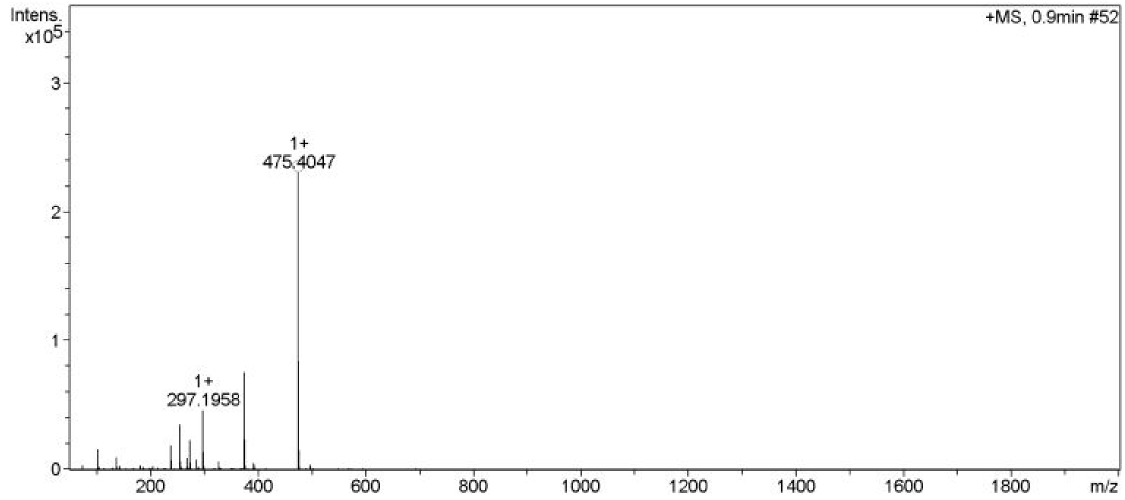

Meas. m/z	#	Ion Formula	m/z	err [mDa]	err [ppm]	rdb	N-Rule	e⁻ Conf	mSigma
475.404732	1	C33H51N2	475.404676	-0.1	-0.1	9.5	ok	even	2.8

*Figure S165: HRMS (m/z)- ESI spectrum of **16**.*

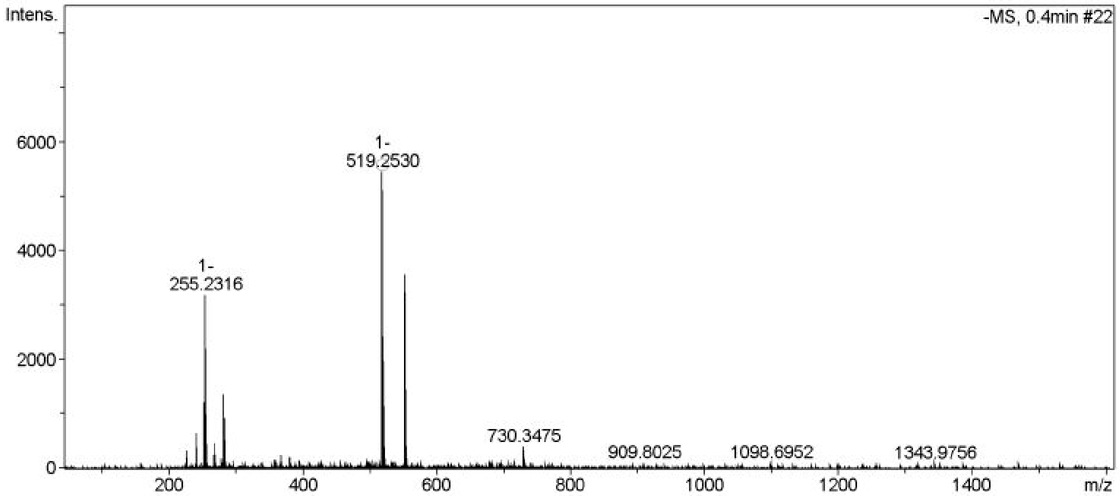

Meas. m/z	#	Ion Formula	m/z	err [mDa]	err [ppm]	rdb	N-Rule	e⁻ Conf	mSigma
519.253017	1	C35H35O4	519.254083	-1.1	-2.1	18.5	ok	even	1.5

*Figure S166: HRMS (m/z)- ESI spectrum of **17**.*

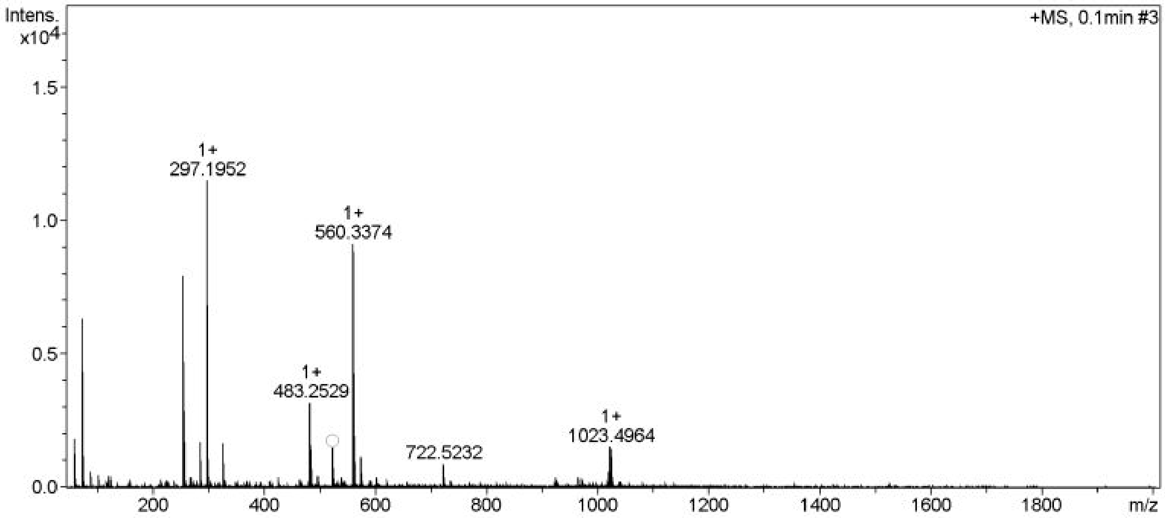

Meas. m/z	#	Ion Formula	m/z	err [mDa]	err [ppm]	rdb	N-Rule	e⁻ Conf	mSigma
523.245439	1	C31H40NaO2Si2	523.245904	0.5	0.9	13.5	ok	even	38.2

*Figure S167: HRMS (m/z)- ESI spectrum of **18**.*

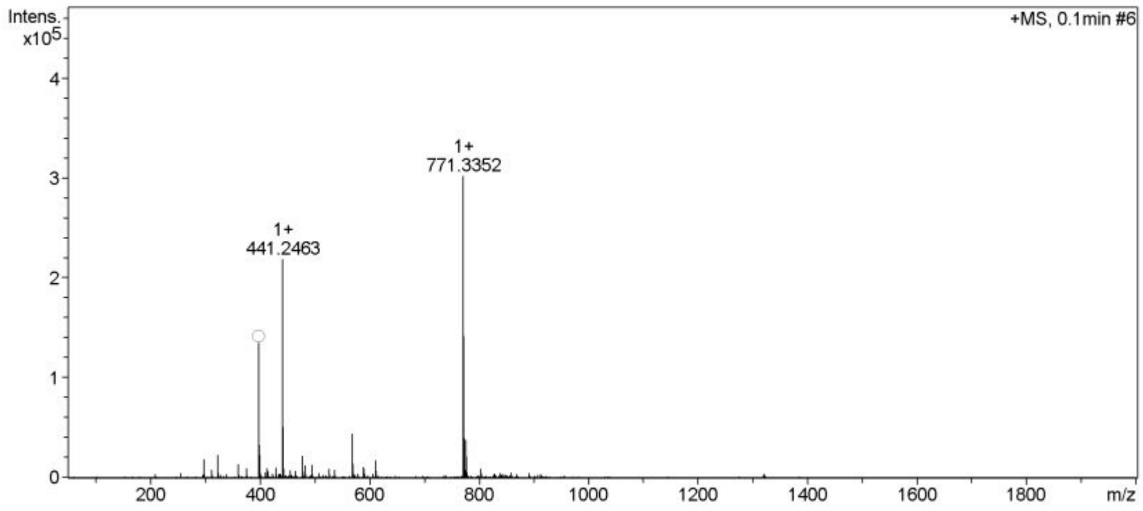

Meas. m/z	#	Ion Formula	m/z	err [mDa]	err [ppm]	rdb	N-Rule	e⁻ Conf	mSigma
397.162355	1	C21H26NaO6	397.162159	-0.2	-0.5	8.5	ok	even	67.7

Figure S168: HRMS (m/z)- ESI spectrum of ***20a****.*

Single X-ray crystallography data

Refinement details for 6b: For compound **6b** the error in the Flack parameter is very high. The Flack test results are ambiguous, and the Flack parameter cannot be determined reliably.

Refinement details for 6c: Compound **6c** was refined as a 2-component inversion twin with a Flack parameter of 0.1(2).

Refinement details for 6m: For compound **6m**, disorder in the BCP ring was refined over two locations with 89:11% occupancy. The solvent DCM partially occupied the lattice with 20% modelled over 4 locations (5.4; 5.4; 4.2 5% occupied). The disorder was modelled using geometric and displacement restraints and constraints (DFIX, DANG, SADI, SIMU, RIGU and EADP) as a rotational twin. The BASF is 0.3245(18) with a rotation angle of -93.319°. The rotation vector (laboratory) is -0.1086 -0.6024 0.7908; rotation vector (reciprocal cell) is 14.00 5.96 6.93; and the rotation vector (direct cell) is 1.00 -0.00 0.00.

Refinement details for 6r: For compound **6r** each terminal methyl ester is disordered over two locations with C21 being 51:49% occupied and C32 being 52:48% occupied. The DCM solvent is also disordered over two locations 54:46% occupied. The disorder was modelled with geometric and displacement restraints (SADI, SIMU, RIGU). The donor N-H hydrogen atoms were located on the difference map and refined with restraints (DFIX).

Refinement Details for 14a: For compound **14a** donor O-H hydrogen atoms were located on the difference map and refined.

Refinement Details for 14b: For compound **14b** disorder manifested in the BCP pinacol tolyl group. This disorder was modelled in two locations with 89:11% occupancy and with the full molecule generated by inversion (1-x, 2-y, 1-z). Geometric and displacement restraints (RIGU,

SIMU, FLAT, SADI, DFIX) used as well as displacement constraints (EADP; C6/C6a, C15A/C15, C9/C9A, C2/C2A, O1/O1A, C1A/C1, C12A/C12). After refinement, the minor disordered component was modelled as a rigid group. The donor O-H hydrogen atoms were located on the difference map and refined with restraints (DFIX).

Crystallographic data for the structures reported in this paper have been deposited with the Cambridge Crystallographic Data Centre. Deposition numbers 2468773 – 2468786 contain the supplementary crystallographic data for this paper. These data can be obtained free of charge from The Cambridge Crystallographic Data Centre via www.ccdc.cam.ac.uk/structures.

Crystallography and refinement details

*Table S1: Crystallography and refinement details for compounds **6a**, **6b**, **6c**, and **6k**.*

Compound numbers	**6a**	**6b**	**6c**	**6k**
Local TCD code	TCD2371	TCD2396	TCD2384	TCD2399
CCDC number	2468773	2468774	2468775	2468776
Empirical formula	$C_{15}H_{16}O_4$	$C_{15}H_{16}O_3$	$C_{16}H_{18}O_5$	$C_{12}H_{12}O_4$
Formula weight	260.28	244.28	290.3	220.22
Temperature [K]	100(2)	100(2)	100(2)	100(2)
Crystal system	monoclinic	orthorhombic	monoclinic	monoclinic
Space group (number)	$P2_1$/n (-14)	$P2_12_12_1$ (-19)	$P2_1$ (-4)	$P2_1$/n (-14)
a [Å]	5.93930(10)	6.1467(4)	5.6035(2)	11.8281(2)
b [Å]	7.9546(2)	6.9781(5)	11.7810(3)	5.97020(10)
c [Å]	27.4018(7)	28.978(2)	11.0103(3)	14.8362(3)
α [°]	90	90	90	90
β [°]	94.0542(12)	90	101.1743(14)	95.7357(13)
γ [°]	90	90	90	90
Volume [Å^3]	1291.35(5)	1242.92(15)	713.06(4)	1042.43(3)
Z	4	4	2	4
ρ_{calc} [gcm^{-3}]	1.339	1.305	1.352	1.403
μ [mm^{-1}]	0.798	0.731	0.833	0.884
F(000)	552	520	308	464
Crystal size [mm^3]	0.235×0.19×0.138	0.392×0.047×0.031	0.433×0.063×0.029	0.162×0.143×0.054
Crystal color	colorless	colorless	colorless	colorless
Crystal shape	block	needle	needle	block
Radiation	Cu K_α (λ = 1.54178 Å)	Cu K_α (λ = 1.54178 Å)	Cu K_α (λ = 1.54178 Å)	Cu K_α (λ = 1.54178 Å)
2θ range [°]	6.47 to 139.33 (0.82 Å)	6.10 to 140.15 (0.82 Å)	8.19 to 139.57 (0.82 Å)	9.13 to 139.53 (0.82 Å)
Index ranges	$-7 \le h \le 6$<br>$-9 \le k \le 9$<br>$-33 \le l \le 33$	$-7 \le h \le 7$<br>$-8 \le k \le 8$<br>$-34 \le l \le 30$	$-6 \le h \le 6$<br>$-14 \le k \le 14$<br>$-12 \le l \le 13$	$-14 \le h \le 14$<br>$-7 \le k \le 7$<br>$-18 \le l \le 18$
Reflections collected	10418	9266	14135	15509
Independent reflections	2427<br>R_{int} = 0.0425<br>R_{sigma} = 0.0318	2336<br>R_{int} = 0.0723<br>R_{sigma} = 0.0666	2670<br>R_{int} = 0.0423<br>R_{sigma} = 0.0286	1966<br>R_{int} = 0.0396<br>R_{sigma} = 0.0220
Completeness to θ = 67.679°	100.00%	100.00%	100.00%	99.90%
Data / Restraints / parameters	2427/0/174	2336/0/165	2670/1/194	1966/0/146
Absorption correction T_{min}/T_{max} (method)	0.5930/0.7533 (multi-scan)	0.5751/0.7533 (multi-scan)	0.5911/0.7533 (multi-scan)	0.6592/0.7533 (multi-scan)
Goodness-of-fit on F^2	1.024	1.049	1.074	1.062
Final R indexes [I≥2σ(I)]	R_1 = 0.0432<br>wR_2 = 0.1207	R_1 = 0.0647<br>wR_2 = 0.1696	R_1 = 0.0369<br>wR_2 = 0.1000	R_1 = 0.0404<br>wR_2 = 0.1079
Final R indexes [all data]	R_1 = 0.0451<br>wR_2 = 0.1234	R_1 = 0.0791<br>wR_2 = 0.1809	R_1 = 0.0375<br>wR_2 = 0.1007	R_1 = 0.0450<br>wR_2 = 0.1127
Largest peak/hole [eÅ^{-3}]	0.34/−0.26	0.45/−0.28	0.22/−0.19	0.28/−0.24
Flack X parameter		0.4(4)	0.1(2)	

*Table S2: Crystallography and refinement details for compounds **6l**, **6m**, **6n**, and **6r.***

Compound numbers	**6l**	**6n**	**6m**	**6o**
Local TCD code	TCD2428	TCD2405	TCD2433_5	TCD2408
CCDC number	2468777	2468778	2468779	2468780
Empirical formula	$C_{12}H_{12}O_3S$	$C_{24}H_{18}O_3$	$C_{18.10}H_{18.20}Cl_{0.20}FeO_3$	$C_{20}H_{18}O_3$
Formula weight	236.28	354.38	346.67	306.34
Temperature [K]	100(2)	100(2)	100(2)	100(2)
Crystal system	monoclinic	monoclinic	triclinic	monoclinic
Space group (number)	*C*2/*c* (-15)	*P*2$_1$/*c* (-14)	$P\bar{1}$ (-2)	*C*2/*c* (-15)
a [Å]	17.9825(5)	13.8430(4)	5.99010(10)	27.0605(7)
b [Å]	6.2550(2)	10.5275(3)	16.1354(4)	6.4466(2)
c [Å]	20.6526(6)	12.1074(3)	17.1314(4)	18.5067(5)
α [°]	90	90	87.3750(10)	90
β [°]	103.4731(13)	95.5150(8)	80.1120(10)	98.6817(8)
γ [°]	90	90	80.8760(10)	90
Volume [Å^3]	2259.08(12)	1756.27(8)	1610.27(6)	3191.47(16)
Z	8	4	4	8
ρ_{calc} [gcm^{-3}]	1.389	1.34	1.43	1.275
μ [mm^{-1}]	2.468	0.702	7.899	0.682
F(000)	992	744	721	1296
Crystal size [mm^3]	0.119×0.167×0.367	0.453×0.213×0.128	0.053×0.059×0.265	0.102×0.245×0.397
Crystal color	colorless	colorless	orange	clear colorless
Crystal shape	block	block	block	block
Radiation	Cu K_α (λ = 1.54178 Å)	Cu K_α (λ = 1.54178 Å)	Cu K_α (λ = 1.54178 Å)	Cu K_α (λ = 1.54178 Å)
2θ range [°]	8.81 to 139.16 (0.82 Å)	6.41 to 139.91 (0.82 Å)	5.24 to 140.31 (0.82 Å)	6.61 to 139.92 (0.82 Å)
Index ranges	$-21 \leq h \leq 21$<br>$-7 \leq k \leq 7$<br>$-25 \leq l \leq 25$	$-16 \leq h \leq 16$<br>$-12 \leq k \leq 12$<br>$-14 \leq l \leq 14$	$-7 \leq h \leq 7$<br>$-19 \leq k \leq 19$<br>$-20 \leq l \leq 20$	$-30 \leq h \leq 32$<br>$-7 \leq k \leq 7$<br>$-22 \leq l \leq 22$
Reflections collected	22201	16646	9550	24806
Independent reflections	2130<br>R_{int} = 0.0412<br>R_{sigma} = 0.0202	3310<br>R_{int} = 0.0313<br>R_{sigma} = 0.0203	9550<br>R_{int} = 0.0513<br>R_{sigma} = 0.0548	3011<br>R_{int} = 0.0344<br>R_{sigma} = 0.0177
Completeness to θ = 67.679°	100.00%	99.90%	99.30%	99.90%
Data / Restraints / Parameters	2130 / 0 / 146	3310/0/245	9550 / 109 / 450	3011 / 0 / 209
Absorption correction T_{min}/T_{max} (method)	0.5969 / 0.7532 (multi-scan)	0.6647/0.7533 (multi-scan)	0.4580 / 0.7533 (multi-scan)	0.6453 / 0.7533 (multi-scan)
Goodness-of-fit on F^2	1.15	1.017	1.06	1.049
Final *R* indexes [$I \geq 2\sigma(I)$]	R_1 = 0.0437<br>wR_2 = 0.1147	R_1 = 0.0394<br>wR_2 = 0.1082	R_1 = 0.0545<br>wR_2 = 0.1419	R_1 = 0.0347<br>wR_2 = 0.0906
Final *R* indexes [all data]	R_1 = 0.0439<br>wR_2 = 0.1149	R_1 = 0.0408<br>wR_2 = 0.1096	R_1 = 0.0659<br>wR_2 = 0.1507	R_1 = 0.0355<br>wR_2 = 0.0913
Largest peak/hole [eÅ^{-3}]	0.41/−0.21	0.27/−0.23	0.73/−0.42	0.23/−0.23

*Table S3: Crystallography and refinement details for compounds **6o**, **7a**, **7b**, and **7l**.*

Compound numbers	**6r**	**7a**	**7b**	**7l**
Local TCD code	TCD2415B	TCD2425	TCD2426	TCD2506
CCDC number	2468781	2468782	2468783	2468784
Empirical formula	$C_{29}H_{27}Cl_2NO_6$	C21H20O4	$C_{21}H_{20}O_2$	$C_{27}H_{20}O_2$
Formula weight	556.41	336.37	304.37	376.43
Temperature [K]	100(2)	100(2)	100(2)	100(2)
Crystal system	monoclinic	monoclinic	triclinic	triclinic
Space group (number)	$C2/c$ -15	$I2/a$ (15)	$P\bar{1}$ -2	$P\bar{1}$ (2)
a [Å]	35.6294(9)	12.3076(3)	9.0725(2)	5.7562(2)
b [Å]	13.5313(3)	7.7654(2)	9.2165(2)	10.7849(4)
c [Å]	10.8797(3)	17.5863(6)	11.8130(3)	45.1472(16)
α [°]	90	90	74.1164(11)	89.8540(14)
β [°]	94.203(2)	99.9790(8)	69.9940(11)	86.3860(13)
γ [°]	90	90	61.0422(10)	87.2895(14)
Volume [Å^3]	5231.1(2)	1655.36(8)	805.08(3)	2794.04(17)
Z	8	4	2	6
ρ_{calc} [gcm^{-3}]	1.413	1.350	1.256	1.342
μ [mm^{-1}]	2.615	0.753	0.623	0.655
F(000)	2320	712	324	1188
Crystal size [mm^3]	0.022×0.187×0.199	0.044×0.123×0.286	0.115×0.165×0.232	0.054×0.242×0.353
Crystal color	colorless	colorless	colorless	colorless
Crystal shape	plate	block	block	plate
Radiation	Cu K_α (λ = 1.54178 Å)	Cu K_α (λ=1.54178 Å)	Cu K_α (λ = 1.54178 Å)	Cu K_α (λ=1.54178 Å)
2θ range [°]	4.97 to 136.15 (0.83 Å)	10.21 to 139.83 (0.82 Å)	8.03 to 139.68 (0.82 Å)	1.96 to 139.92 (0.82 Å)
Index ranges	−42 ≤ h ≤ 42<br>−16 ≤ k ≤ 16<br>−13 ≤ l ≤ 12	−14 ≤ h ≤ 14<br>−9 ≤ k ≤ 9<br>−21 ≤ l ≤ 21	−11 ≤ h ≤ 11<br>−11 ≤ k ≤ 11<br>−14 ≤ l ≤ 14	−6 ≤ h ≤ 7<br>−12 ≤ k ≤ 11<br>−54 ≤ l ≤ 54
Reflections collected	31104	20709	11476	37861
Independent reflections	4758<br>R_{int} = 0.0576<br>R_{sigma} = 0.0351	1564<br>R_{int} = 0.0362<br>R_{sigma} = 0.0144	3016<br>R_{int} = 0.0306<br>R_{sigma} = 0.0239	10407<br>R_{int} = 0.0334<br>R_{sigma} = 0.0317
Completeness to θ = 67.679°	99.90%	100.0 %	99.90%	99.8 %
Data / Restraints / Parameters	4758 / 305 / 453	1564 / 0 / 115	3016 / 0 / 210	10407 / 0 / 785
Absorption correction T_{min}/T_{max} (method)	0.5824 / 0.7530 (multi-scan)	0.6716 / 0.7533 (multi-scan)	0.6789 / 0.7533 (multi-scan)	0.6665 / 0.7533 (multi-scan)
Goodness-of-fit on F^2	1.041	1.071	1.069	1.096
Final R indexes [I≥2σ(I)]	R_1 = 0.0654<br>wR_2 = 0.1850	R1 = 0.0371<br>wR2 = 0.1058	R_1 = 0.0415<br>wR_2 = 0.1169	R_1 = 0.0440<br>wR_2 = 0.1369
Final R indexes [all data]	R_1 = 0.0790<br>wR_2 = 0.1988	R1 = 0.0388<br>wR2 = 0.1077	R_1 = 0.0439<br>wR_2 = 0.1199	R_1 = 0.0630<br>wR_2 = 0.1545
Largest peak/hole [eÅ^{-3}]	0.79/−0.49	0.24/−0.18	0.26/−0.22	0.28/−0.21

*Table S4: Crystallography and refinement details for compounds **14a** and **14b**.*

Compound numbers	**14a**	**14b**
Local TCD code	TCD2483	TCD2479
CCDC number	2468785	2468786
Empirical formula	$C_{30}H_{34}O_6$	$C_{30}H_{34}O_6$
Formula weight	490.57	490.57
Temperature [K]	100(2)	100(2)
Crystal system	orthorhombic	triclinic
Space group (number)	*Pbca*<br>-61	$P\bar{1}$<br>-2
a [Å]	13.4602(5)	9.7107(3)
b [Å]	10.4994(4)	10.9472(3)
c [Å]	17.8978(6)	12.9197(4)
α [°]	90	70.8819(12)
β [°]	90	81.8108(13)
γ [°]	90	75.2020(13)
Volume [Å^3]	2529.39(16)	1252.04(7)
Z	4	2
ρ_{calc} [gcm^{-3}]	1.288	1.301
μ [mm^{-1}]	0.718	0.726
F(000)	1048	524
Crystal size [mm^3]	0.214×0.426×0.484	0.078×0.189×0.205
Crystal color	colorless	colorless
Crystal shape	block	block
Radiation	Cu K_α (λ = 1.54178 Å)	Cu K_α (λ = 1.54178 Å)
2θ range [°]	9.88 to 139.77 (0.82 Å)	7.26 to 140.24 (0.82 Å)
Index ranges	$-16 \leq h \leq 16$<br>$-10 \leq k \leq 12$<br>$-21 \leq l \leq 21$	$-11 \leq h \leq 11$<br>$-13 \leq k \leq 13$<br>$-15 \leq l \leq 15$
Reflections collected	25302	21066
Independent reflections	2394<br>R_{int} = 0.0339<br>R_{sigma} = 0.0189	4701<br>R_{int} = 0.0372<br>R_{sigma} = 0.0265
Completeness to<br>θ = 67.679°	100.00%	99.90%
Data / Restraints / Parameters	2394 / 284 / 218	4701 / 0 / 337
Absorption correction<br>T_{min}/T_{max} (method)	0.6638 / 0.7533<br>(multi-scan)	0.6719 / 0.7533<br>(multi-scan)
Goodness-of-fit on F^2	1.094	1.054
Final *R* indexes<br>[$I \geq 2\sigma(I)$]	R_1 = 0.0453<br>wR_2 = 0.1234	R_1 = 0.0488<br>wR_2 = 0.1400
Final *R* indexes<br>[all data]	R_1 = 0.0456<br>wR_2 = 0.1237	R_1 = 0.0520<br>wR_2 = 0.1444
Largest peak/hole [eÅ^{-3}]	0.31/−0.23	0.48/−0.24

Table S5. Torsion angles in BCP ketones. Using majority occupied moieties only for calculations.

Entry	Compound	O(ketone)-CH2 BCP	Torsion angle [°]	CH2 BCP-O(ester)	Torsion angle [°]
1	**6a**	O10–C9–C11–C12	6.62(19)	C12–C15–C16–O17	21.6(2)
2	**6b**	O1–C8–C9–C12	−2.1(5)	C12–C13–C14–O2	−5.1(6)
3	**6c**	O3–C9–C10–C11	-35.0(3)	C11-C14-C15-O4	-109.2(3)
4	**6k**	O2–C6–C7–C8	−16.0(2)	C8–C11–C12–O3	−32.8(2)
5	**6l**	O7–C6–C8–C9	−4.9(3)	C9–C12–C13–O14	−20.3(3)
6	**6m**	O12-C11-C13-C14	19.0(6)	C14-C17-C18-O19	23.5(7)
7	**6n**	O1–C17–C18–C21	-27.20(17)	C21-C22-C23-O2	82.70(17)
8	**6o**	O1–C13–C14–C15	-1.95(16)	C15–C18–C19–O2	119.56(13)
9	**6r**	O15-C14-C16-C17	-6.4(4)	C17-C20-C21A-O22A	-155.9(9)
10	**6r**	O26-C25-C27-C29	-22.6(5)	C29-C31-C32A-O33A	-9.5(14)
**Entry**	**Compound**	**O(ketone1)-CH2 BCP**	**Torsion angle [°]**	**O(ketone2)-CH2 BCP**	**Torsion angle [°]**
11	**7a**	O10-C9-C11-C13	-2.41(17)	O10-C9-C11-C13#1	-122.71(13)
12	**7b**	O9-C10-C8-C13	-15.40(19)	O16-C15-C14-C13	159.68(15)
13	**7l**	O1-C11-C12-C15	12.9(4)	O2-C17-C16-C15	89.6(4)

Table S6. Graph-set notation for crystal structures 6r, 14a, 14b.

Entry	Compound	Graph-set notation
1	**6r**	$C_1^1(8)$
2	**14a**	$C_1^1(8)$, $C_1^1(9)$, $C_2^2(18)$, $R_4^4(34)$, $R_6^6(52)$
3	**14b**	$R_2^2(16)$

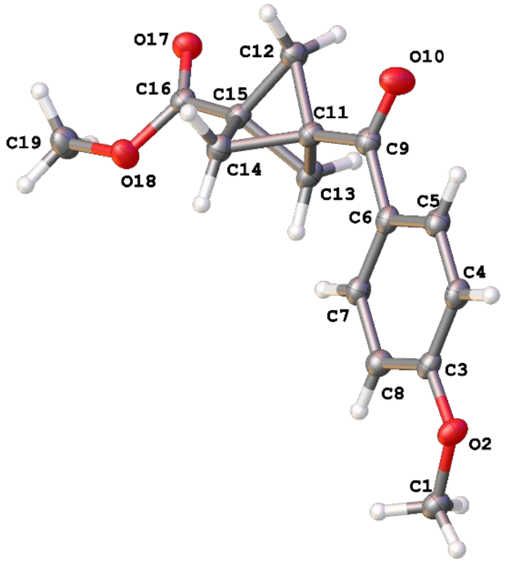

*Figure S169: View of the molecular structure of **6a** in the crystal. Displacement parameters shown at 50% probability.*

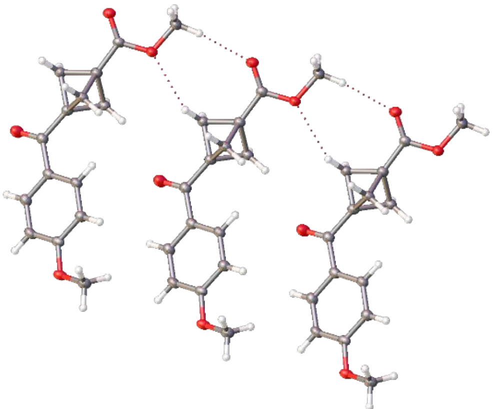

*Figure S170: View of the molecular structure of **6a** in the crystal showing hydrogen bonding between the hydrogens in the BCP ring bridgehead position and the methoxy-oxygen atom.*

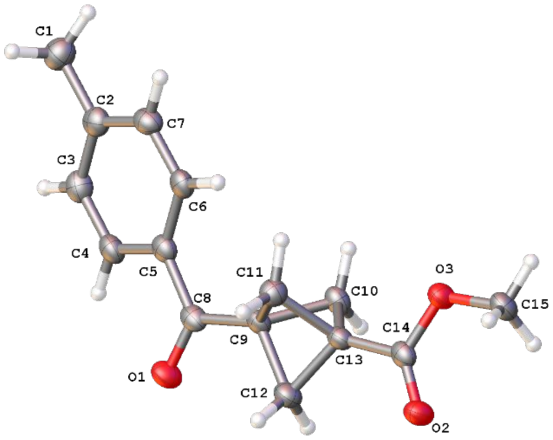

*Figure S171:View of the molecular structure of **6b** in the crystal. Displacement parameters shown at 50% probability.*

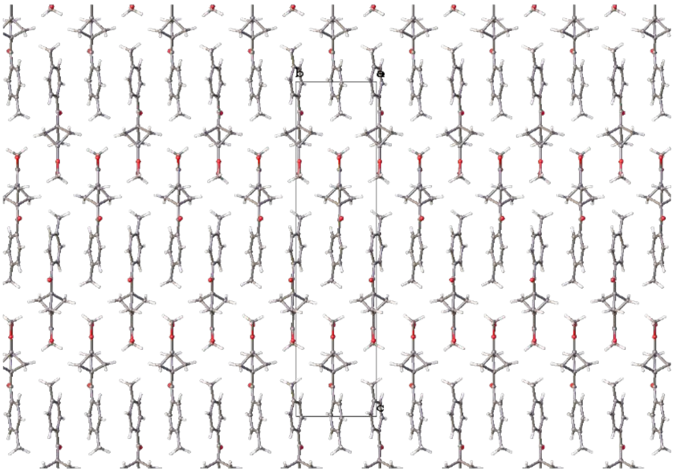

*Figure S172:Schematic packing diagram of **6b** viewed normal to the a-axis.*

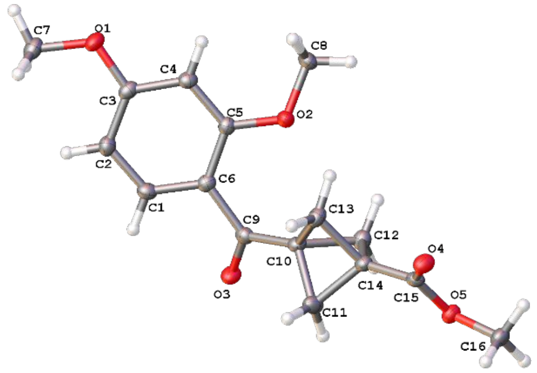

*Figure S173:View of the molecular structure of **6c** in the crystal. Displacement parameters shown at 50% probability.*

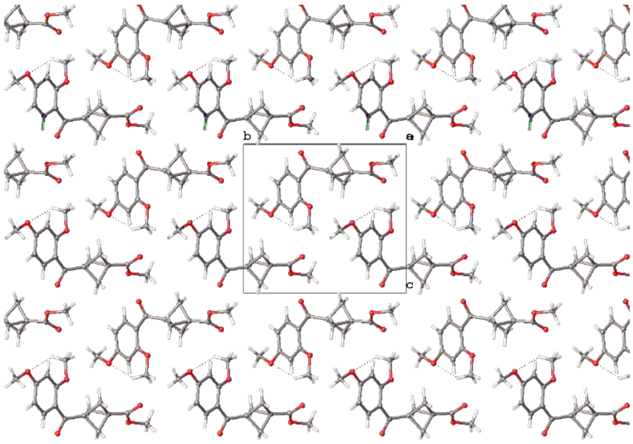

*Figure S174:Schematic packing diagram of **6c** viewed normal to the a-axis. Dotted lines indicate possible hydrogen bonding interactions.*

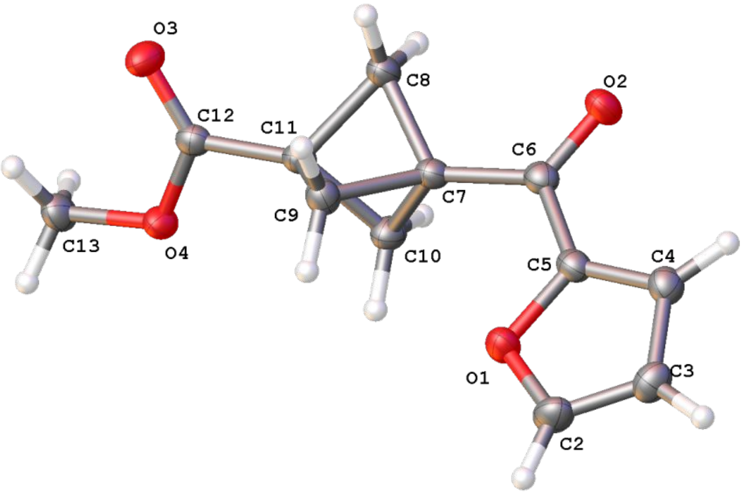

*Figure S175:View of the molecular structure of **6k** in the crystal. Displacement parameters shown at 50% probability.*

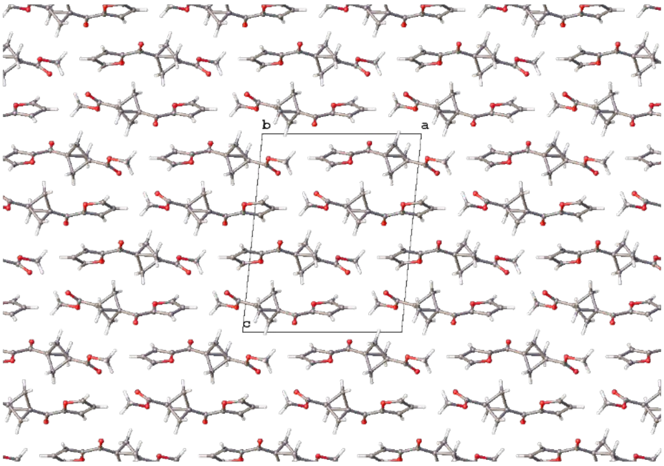

*Figure S176:Schematic packing diagram of **6k** viewed normal to the b-axis.*

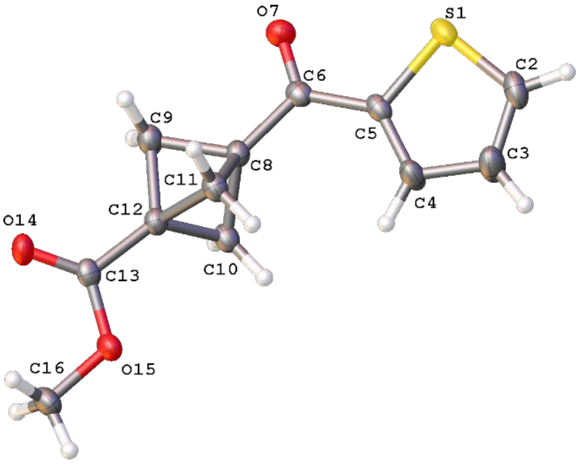

*Figure S177: View of the molecular structure of **6l** in the crystal with atomic displacement parameters shown at 50% probability.*

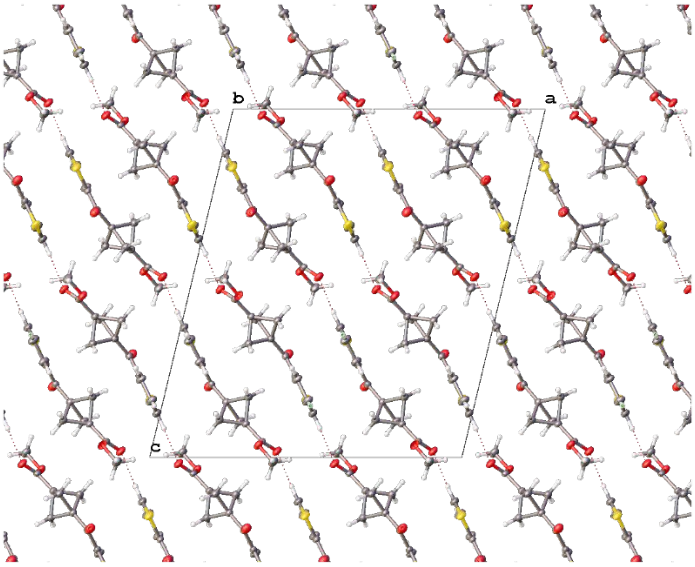

*Figure S178: Schematic packing representations of **6l** viewed normal to the b-axis. Dotted lines indicate hydrogen bonding interactions (see Table 6 below).*

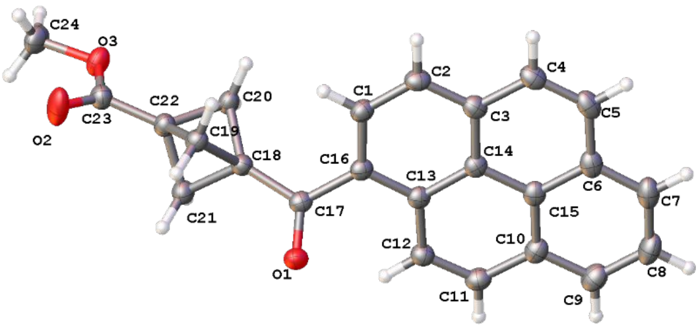

Figure S179:View of the molecular structure of ***6n*** *in the crystal. Displacement parameters shown at 50% probability.*

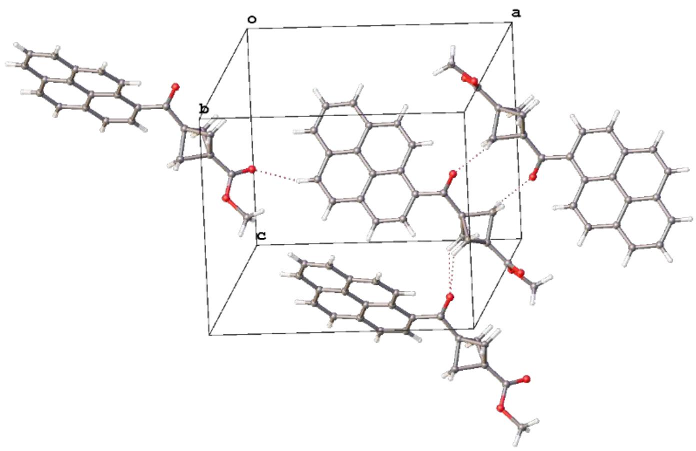

Figure S180: Potential hydrogen bonding pattern seen in ***6n****, exhibiting single and bifurcated bonds between BCP methylene hydrogens and carbonyl oxygen atoms. Bonding is also seen between pyrene hydrogens and carbonyl oxygens.*

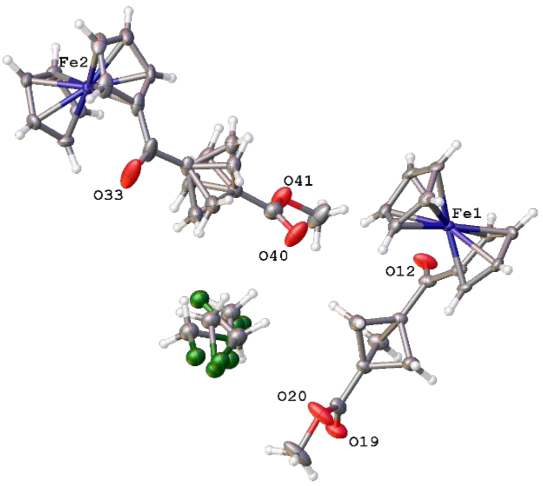

Figure S181: View of the molecular structure of ***6m*** *in the crystal, showing the disordered BCP and DCM exhibiting disorder and partial occupation of the crystal lattice. Displacement parameters shown at 50% probability and selected heteroatoms labelled only.*

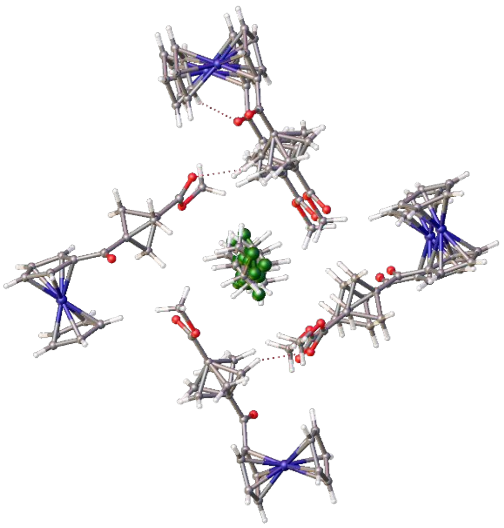

Figure S182: Detail of the hydrogen bonding between methoxy oxygen atoms and BCP methylene hydrogen in ***6m*** *represented by dotted lines.*

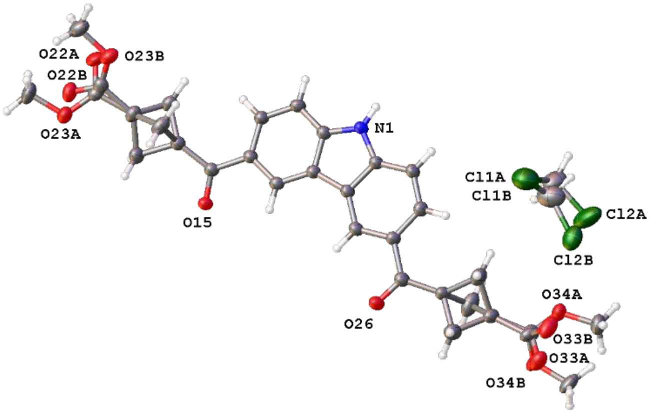

Figure S183: View of the molecular structure of ***6r*** *in the crystal with disorder in the terminal methyl ester moieties. DCM shown to occupy the crystal lattice and exhibit disorder. Displacement parameters shown at 50% probability.*

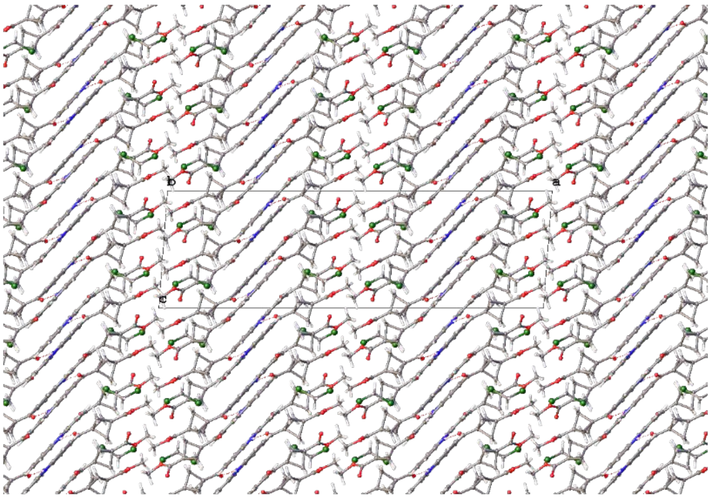

Figure S184: Schematic packing diagram of majority occupied moiety in ***6r*** *viewed normal to the b-axis. Dotted lines indicate hydrogen-bonding interactions.*

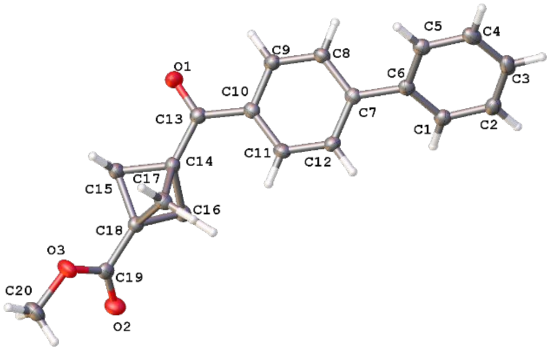

*Figure S185: View of the molecular structure of **6o** in the crystal with full atom labelling. Displacement parameters shown at 50% probability.*

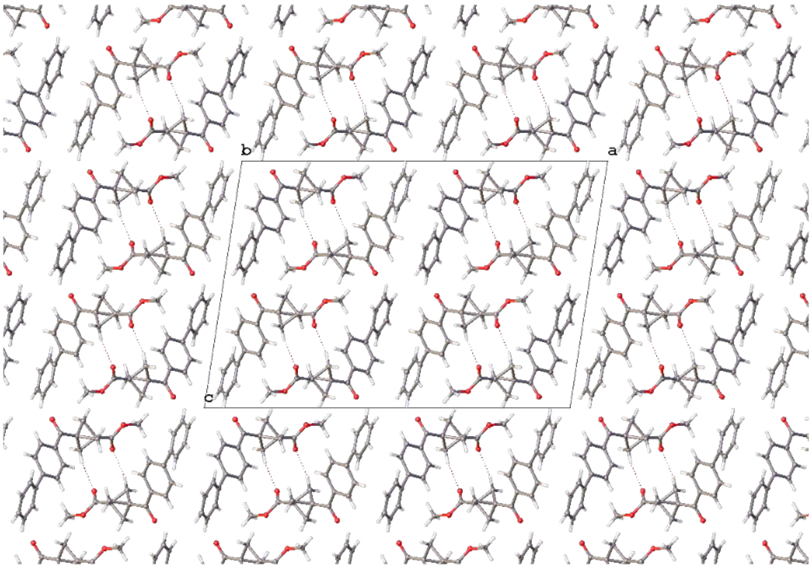

*Figure S186:Schematic packing diagram of **6o** viewed normal to the b-axis. Dotted lines indicate hydrogen-bonding interactions.*

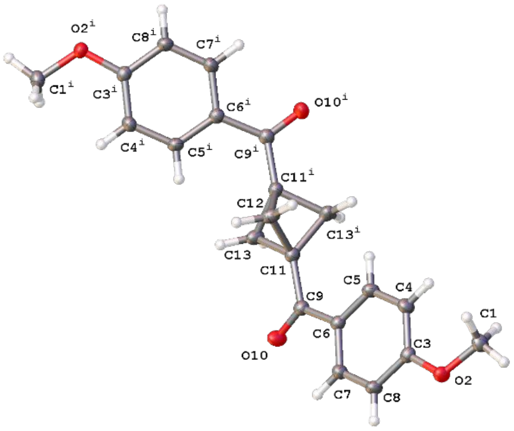

*Figure S187: View of molecular structure of **7a** in the crystal with full atom labelling generated with 2-fold axis symmetry. Symmetry transformation used i=1/2-X, +Y, 1-Z. Displacement parameters shown at 50%.*

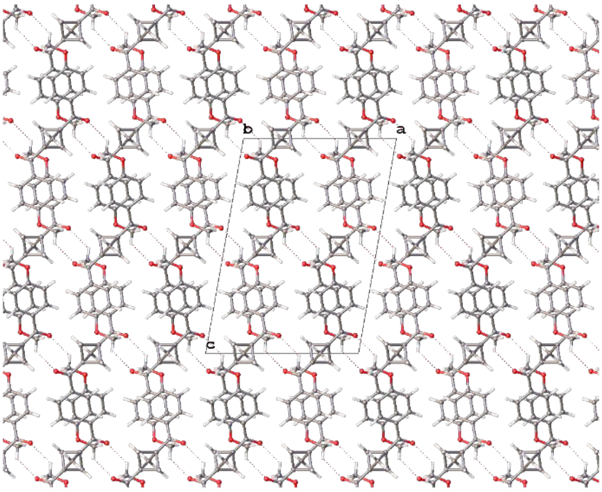

*Figure S188: Schematic packing diagram of **7a** viewed normal to the b-axis. Dotted lines indicate hydrogen-bonding interactions.*

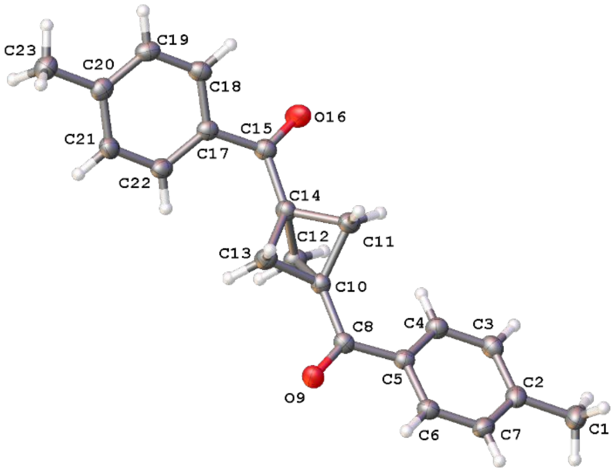

*Figure S189: View of the molecular structure of **7b** in the crystal with atomic displacement parameters shown at 50% probability.*

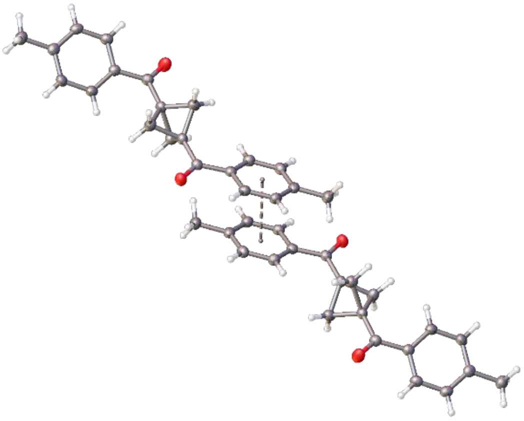

*Figure S190: Potential π-π interaction in **7b**. Displacement parameters shown at 50% probability.*

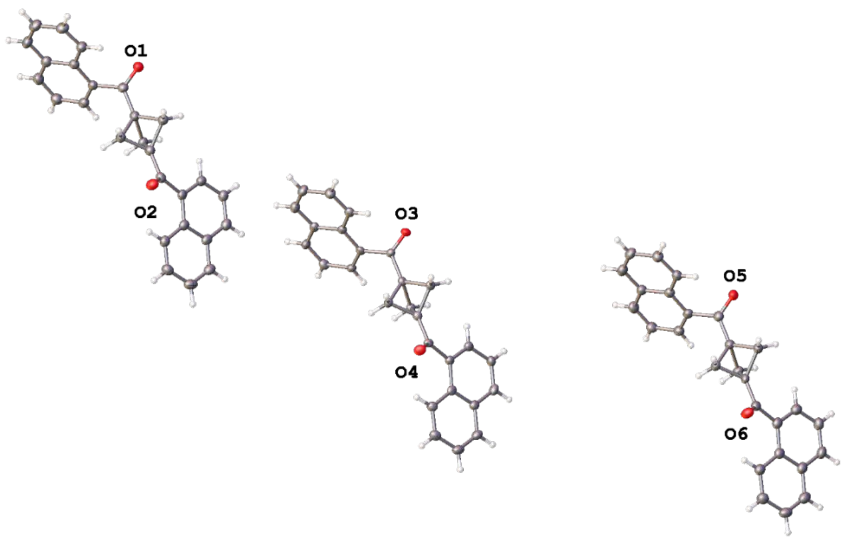

*Figure S191: View of the molecular structure of **7l** in the crystal with heteroatoms labelled. Three independent molecules are shown in the asymmetric unit. Displacement parameters are shown at 50% probability.*

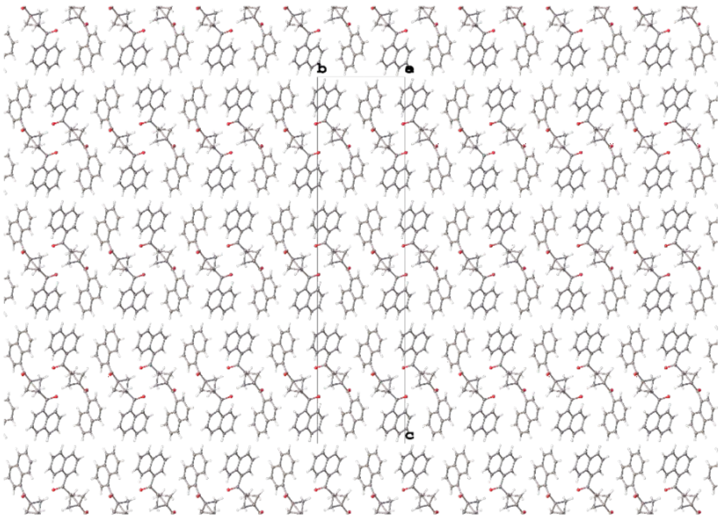

*Figure S192: Schematic packing diagram of **7l** viewed normal to the a-axis.*

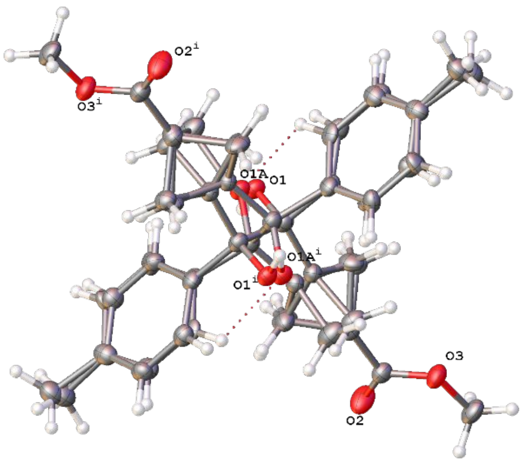

*Figure S193: View of the molecular structure of **14a** with labelling of heteroatoms only. The structure was symmetry generated through inversion and exhibits disorder. Displacement parameters shown at 50% probability. Intramolecular hydrogen bonding shown as dotted lines.*

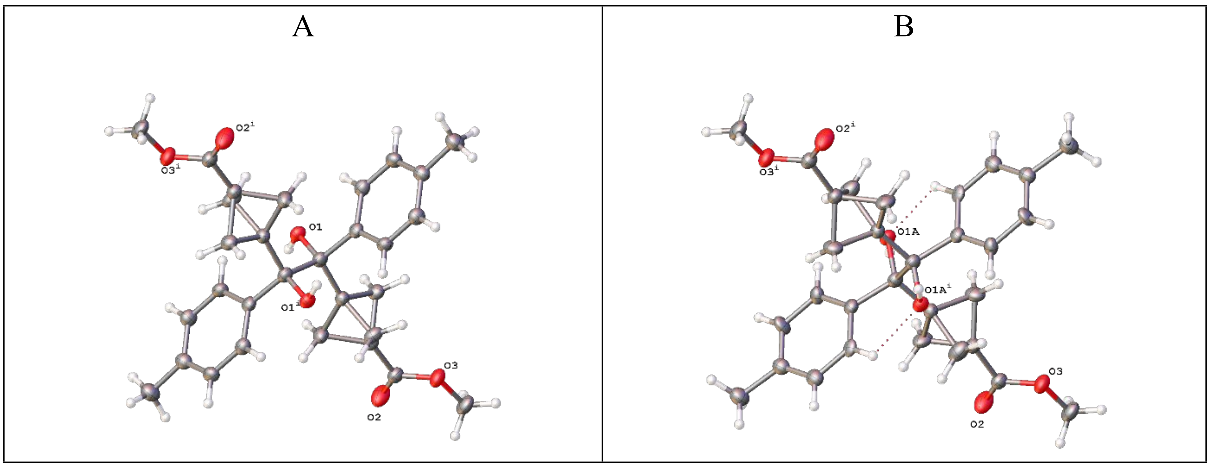

*Figure S194: Individual representations of each symmetry generated disordered moiety in **14a** with (A) 89% occupied and (B) 11% occupied. Donor O-H hydrogen located and refined and only heteroatoms are labelled. Displacement parameters shown at 50% probability. Intramolecular hydrogen bonding shown as dotted lines. Symmetry transformation i = 1-x, 2-y, 1-z used to generate the structure.*

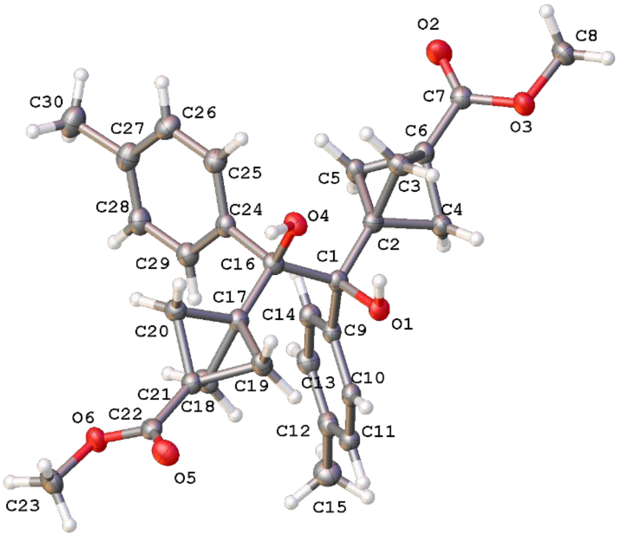

*Figure S195: View of the molecular structure **14b** in the crystal with all atoms labelled. Displacement parameters shown at 50% probability.*

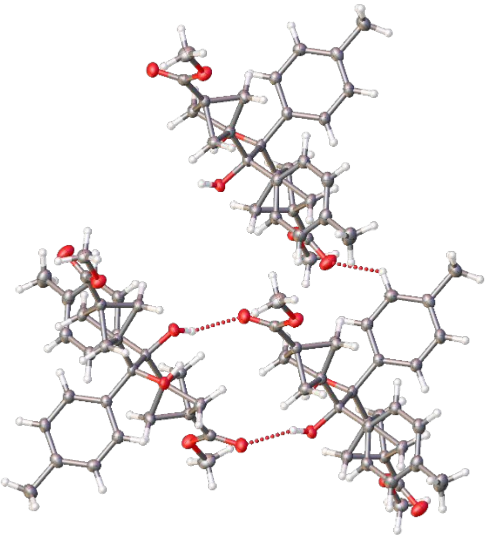

*Figure S196: Detail of intermolecular hydrogen bonding seen in the molecular structure of **14b** in the crystal represented by dotted lines.*

Hirshfeld Surface Analysis

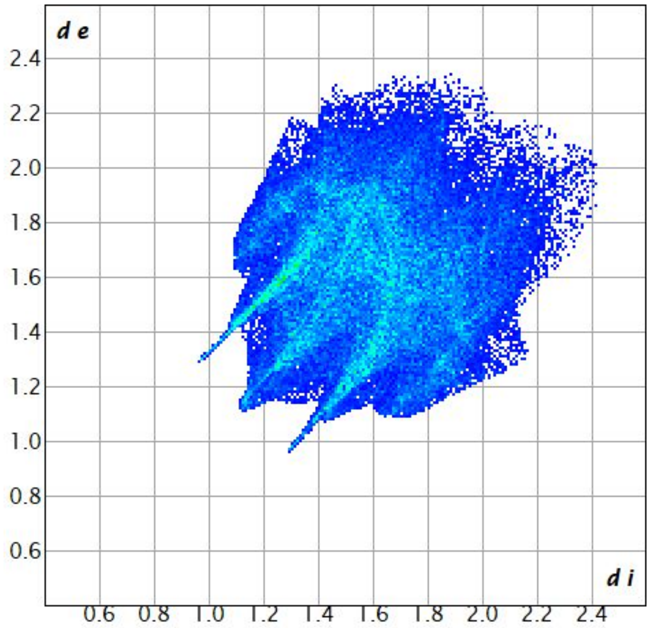

Figure S197: Two-dimensional fingerprint plot of compound ***6a****.*

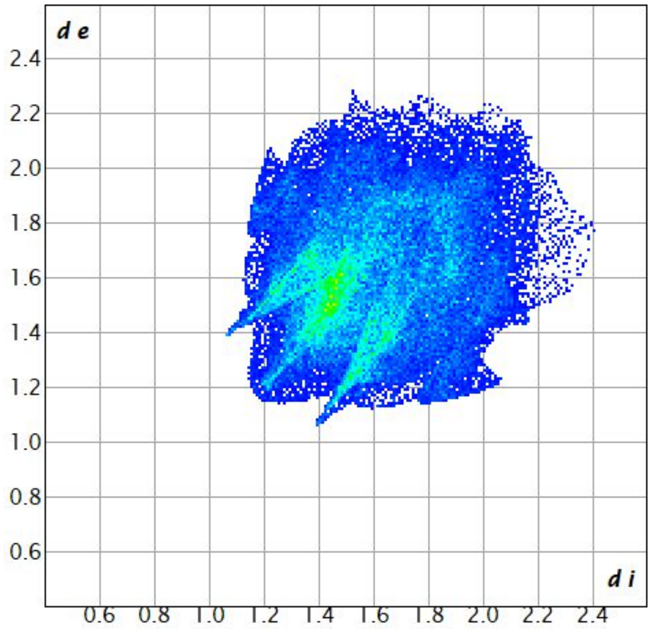

Figure S198: Two-dimensional fingerprint plot of compound ***6b****.*

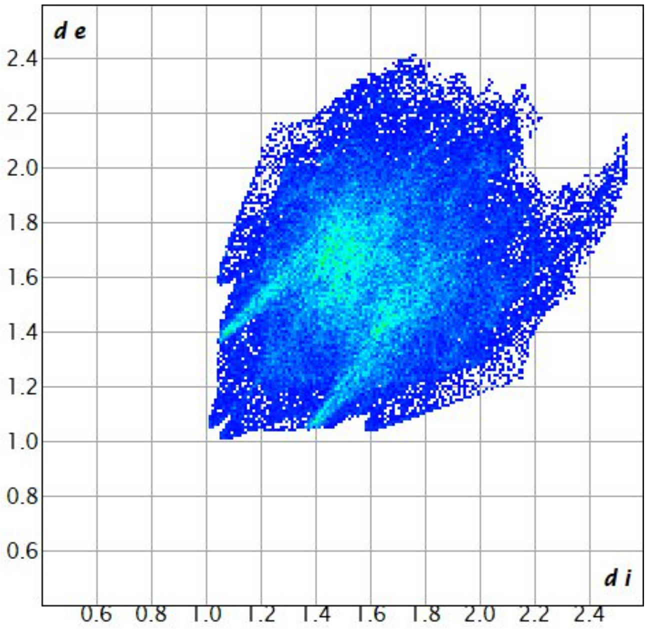

*Figure S199: Two-dimensional fingerprint plot of compound **6c**.*

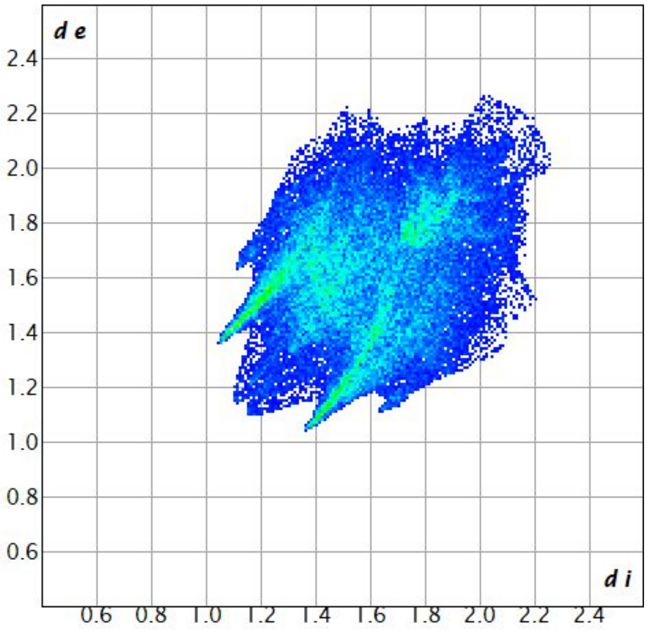

*Figure S200: Two-dimensional fingerprint plot of compound **6k**.*

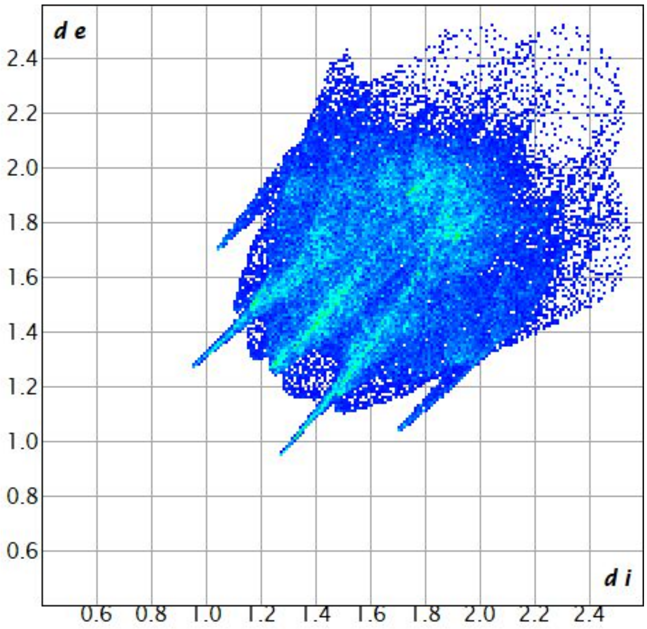

*Figure S201: Two-dimensional fingerprint plot of compound **6l**.*

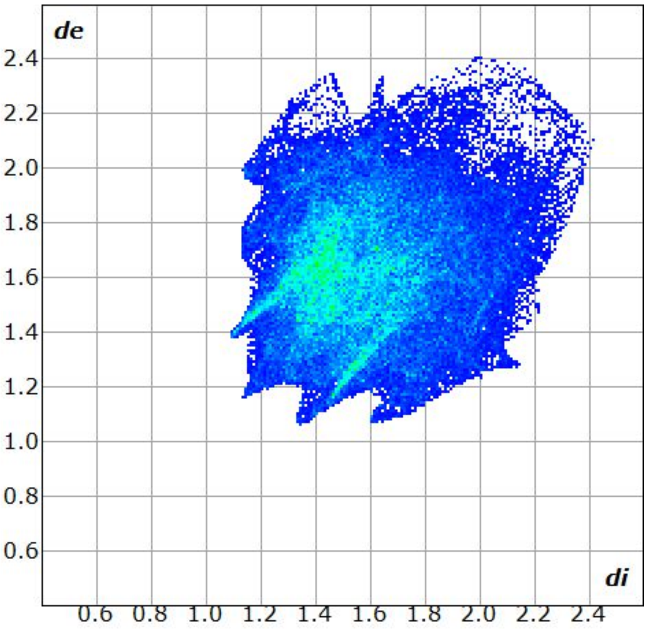

*Figure S202: Two-dimensional fingerprint plot of compound **6m**.*

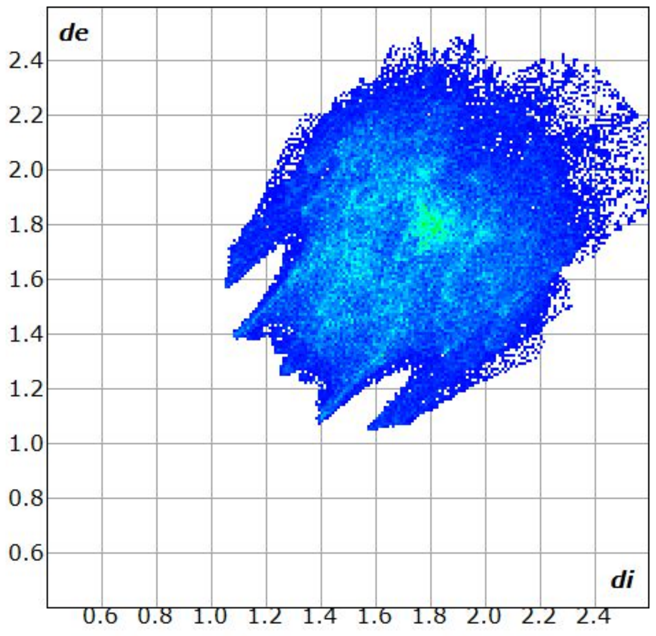

Figure S203: Two-dimensional fingerprint plot of compound ***6n****.*

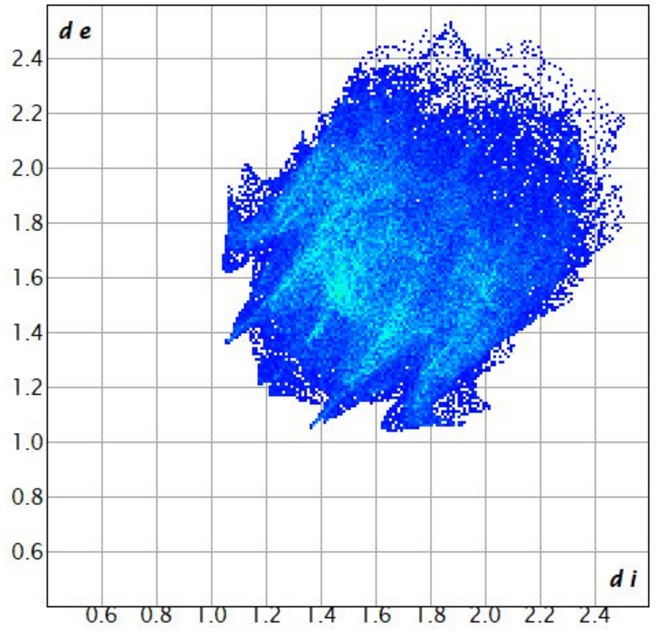

Figure S204: Two-dimensional fingerprint plot of compound ***6o****.*

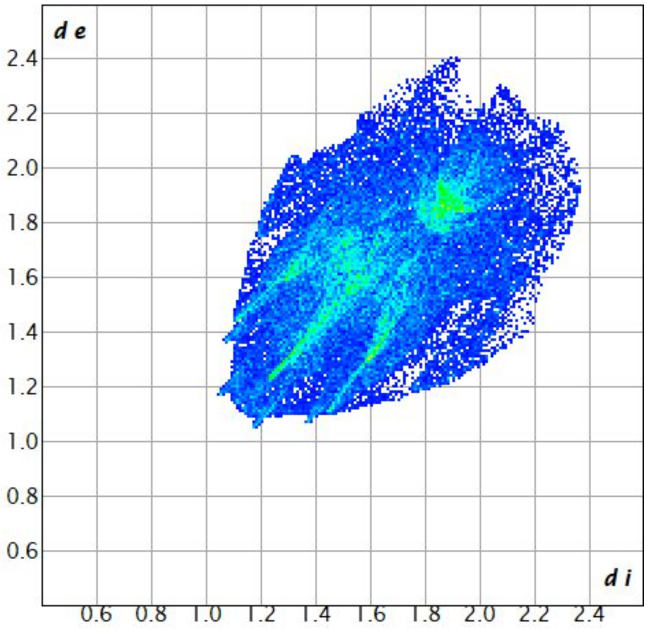

Figure S205: Two-dimensional fingerprint plot of compound ***7a.***

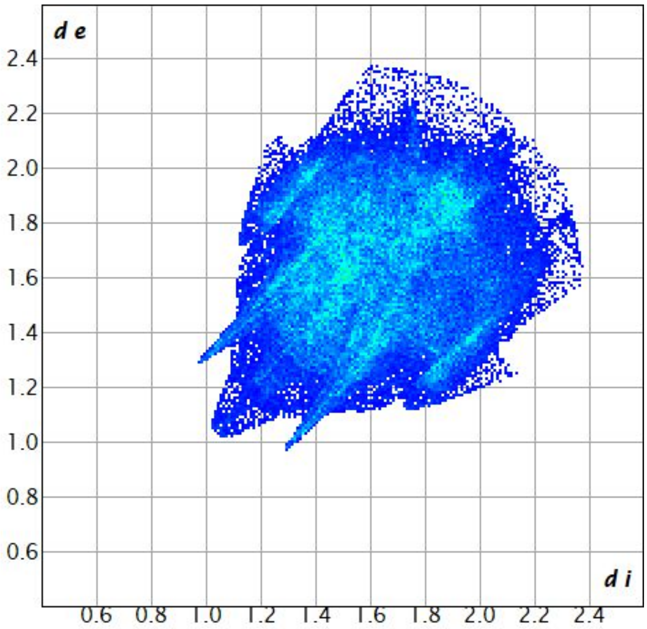

Figure S206: Two-dimensional fingerprint plot of compound ***7b.***

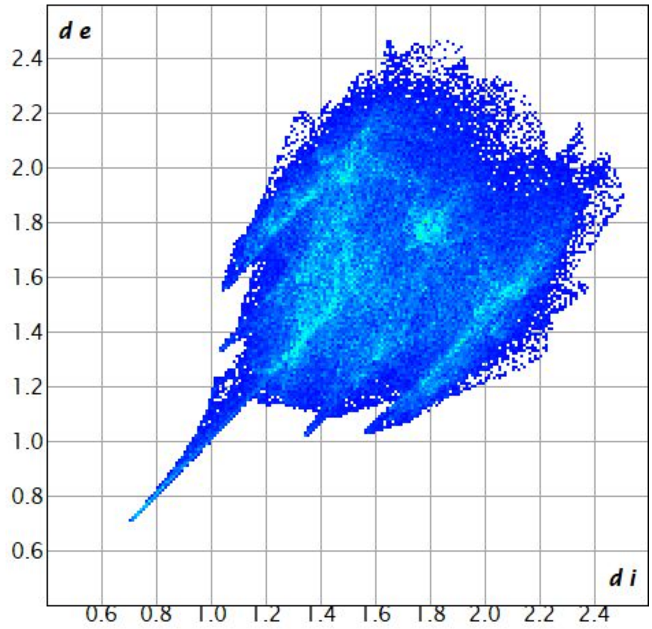

Figure S207: Two-dimensional fingerprint plot of compound ***7l.***

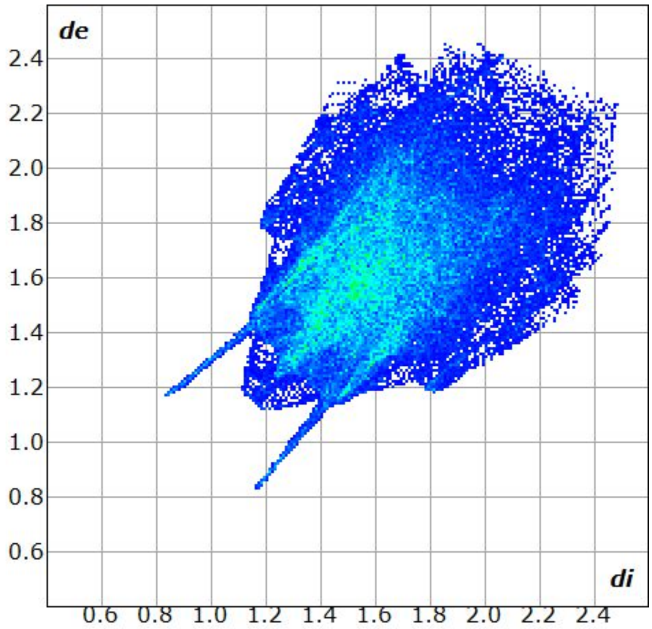

Figure S208: Two-dimensional fingerprint plot of compound ***14a.***

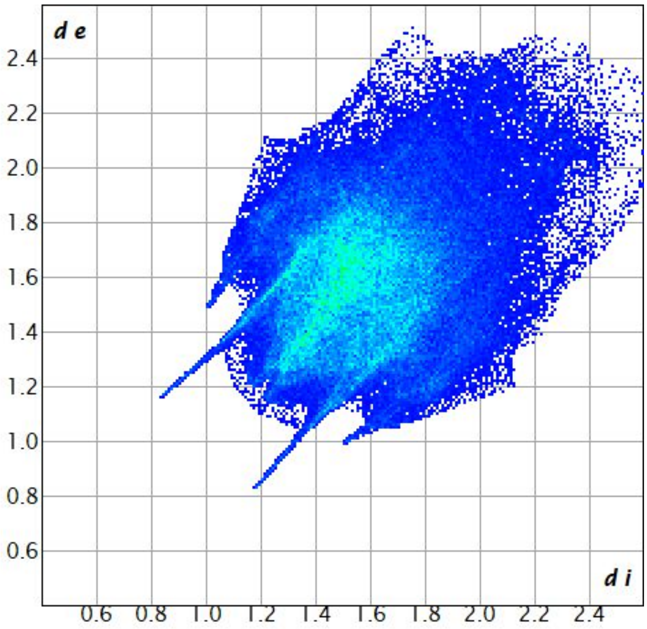

*Figure S209: Two-dimensional fingerprint plot of compound **14b.***
